# Functionalization and Hydrogenation of Carbon Chains Derived from CO**

**DOI:** 10.1002/anie.202202241

**Published:** 2022-03-16

**Authors:** Maria Batuecas, Richard Y. Kong, Andrew J. P. White, Mark R. Crimmin

**Affiliations:** ^1^ Department of Chemistry MSRH Imperial College London 82 Wood Lane, Shepherds Bush London W12 0BZ UK

**Keywords:** CO, Fischer–Tropsch, Homologation, Metallocarbenes, Reaction Mechanisms

## Abstract

Selective reactions that combine H_2_, CO and organic electrophiles (aldehyde, ketones, isocyanide) to form hydrogenated C_3_ and C_4_ carbon chains are reported. These reactions proceed by CO homologation mediated by [W(CO)_6_] and an aluminum(I) reductant, followed by functionalization and hydrogenation of the chain ends. A combination of kinetics (rates, KIEs) and DFT calculations has been used to gain insight into a key step which involves hydrogenation of a metallocarbene intermediate. These findings expand the extremely small scope of systems that combine H_2_ and CO to make well‐defined products with complete control over chain length and functionality.

## Graphical abstract

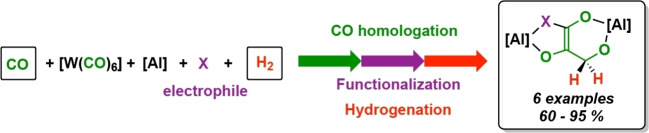

The controlled polymerization, hydrogenation and dehydration of CO/H_2_ mixtures to form hydrocarbons by the Fischer–Tropsch (F–T) process is an essential reaction for industry.[^1^, ^2^] There has long‐been interest in controlling the selectivity of this reaction.^[3]^ Many have advocated the potential of homogeneous catalysts to lead to reaction products with defined molecular weight and oxygen content.[^4^, ^5^]

Despite these ambitions, homogeneous reactions that lead to F–T products are incredibly rare.[^4^, ^6^, ^7^, ^8^, ^9^, ^10^, ^11^, ^12^, ^13^] Our fundamental understanding of this type of reactivity is limited; there are only a handful of well‐defined systems that combine CO and H_2_ in a single reaction sequence to form either hydrocarbon (C_*x*_H_*y*_) or oxygenate (C_*x*_H_*y*_O_*z*_) products. In 1991, Lippard and co‐workers reported the reductive coupling and hydrogenation of CO to form *cis*‐disiloxyethylene compounds, mediated by vanadium complexes.^[14]^ More recently, Peters and Suess reported a similar product from the hydrogenation of a CO derived iron dicarbyne.^[15]^ Hou and co‐workers have documented the hydrodeoxygenative cyclotetramerization of CO by a trinuclear titanium poly(hydride) complex to form a cyclobutanone product.^[16]^ Stephan and co‐workers have shown that a simple lithium amide base (LiNCy_2_) can react with CO/H_2_ mixtures to form small amounts (<10 % yield) of an α‐hydroxy amide derived from coupling and hydrogenation of two CO units.^[17]^

These systems represent the limit of knowledge in this field and have clear limitations. To date only C_2_ and C_4_ hydrogenated chain‐growth products have been isolated. There are no examples of generating more complex products by incorporating organic electrophiles (other than CO) within the carbon chain. There is also a lack of detailed mechanistic information on the hydrogenation step. A broader scope and deeper understanding of these types of transformations could be an important factor in ultimately achieving selective F–T catalysis.

Herein, we describe the direct hydrogenation of a series of CO homologation products, including for the first time, well‐defined reactivity of C_3_ carbon chains. We show that F–T products can be obtained by reaction with CO, organic electrophiles, a main group reductant and H_2_. We provide a mechanistic description of the key hydrogenation step, shedding light on a key C−H bond formation pathway of relevance to F–T catalysis. We have previously reported carbon‐chain growth reactions from **1**, [W(CO)_6_] and CO.[^18^, ^19^] Reaction of **2** with benzophenone at 100 °C in C_6_D_6_ led to the formation of **3 a** and **4 a** in 81 % yield, in a 4 : 1 ratio based on ^1^H NMR spectroscopy (Scheme 1).

**Scheme 1:**
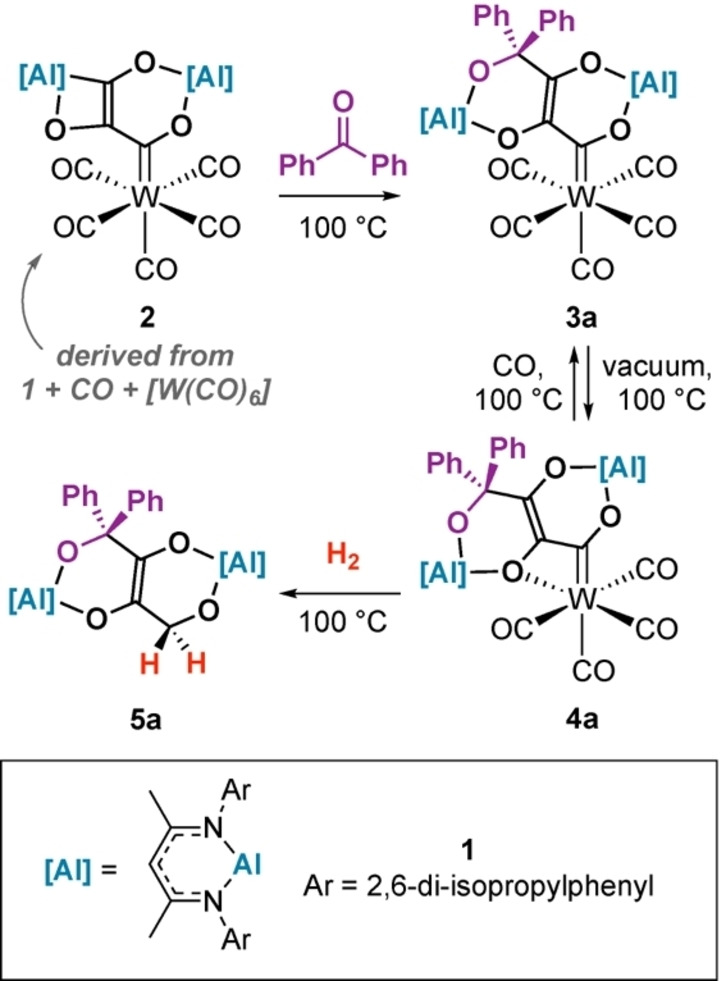
Reactions of **2** with benzophenone and H_2_.

The conversion of **3 a** to **4 a** is reversible. Heating mixtures of **3 a**+**4 a** under 1 atm. of CO for 12 h at 100 °C led to complete conversion to **3 a**. Upon heating under vacuum, **3 a** partially converts back to **4 a**. DFT calculations are consistent with the reversible reaction. Formation of **4 a** from **3 a** was calculated to be endergonic (Δ*G*°_298K_=+6.2 kcal mol^−1^) and occur via an interchange mechanism (Δ*G* ^≠^_298K_=+25.4 kcal mol^−1^) (Figure 1a).

**Figure 1:**
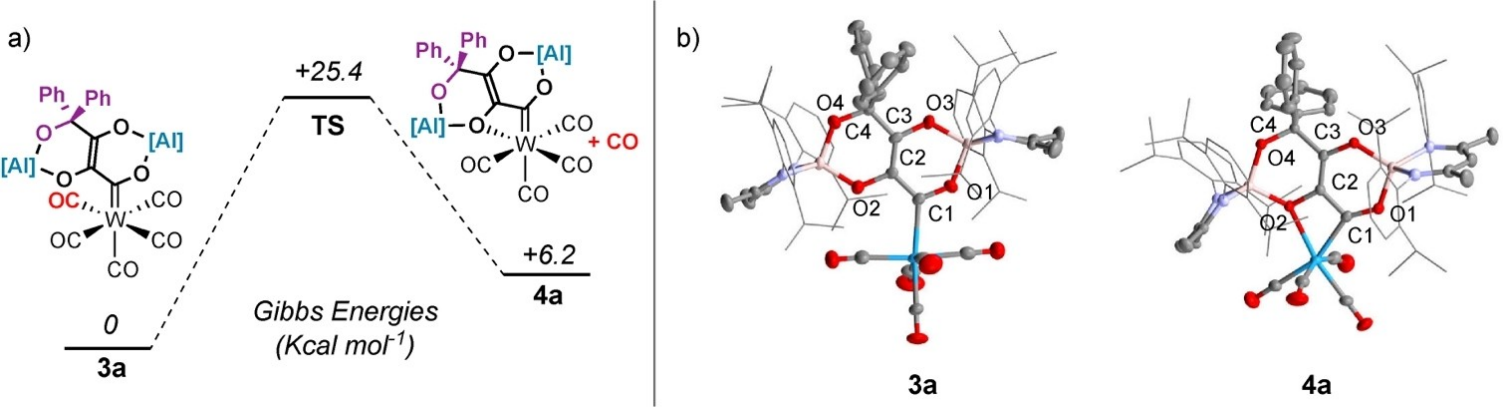
a) DFT calculated mechanism for transformation of **3 a** to **4 a**. b) Solid‐state structures of **3 a** and **4 a**.

Compounds **3 a** and **4 a** have been characterized by multinuclear NMR and IR spectroscopy. In C_6_D_6_ solution, **3 a** and **4 a** display ^13^C NMR resonances for the metallocarbene ligand at *δ*=315.6 and 310.9 ppm respectively. The equatorial and axial carbonyl ligands of **3 a** are magnetically inequivalent and appear at *δ*=203.3 and 205.1 ppm. For comparison, **4 a** shows three resonances for the CO ligands in the ^13^C NMR spectrum at *δ*=215.5, 218.8 and 221.4 ppm due to the reduction in symmetry. IR spectroscopy is consistent with a change in geometry around the metal center from **3 a** (ν (CO)=2050, 1897 and 1871 cm^−1^) to **4 a** (ν (CO)=1988, 1874, 1862 and 1825 cm^−1^) due to CO dissociation.

In the solid‐state, the W−C bond length of **3 a** of 2.269(4) Å is longer than that of 2.195(3) Å found in **2**. Formation of the κ^2^‐C,O coordination mode occurs with a large distortion away from an ideal octahedral geometry at W, an effect driven by the acute bite angle of 60.9(1) ° of the chelating ligand.^[20]^ This distortion also influences the geometry at the metallocarbene fragment. The W−C^1^−O^1^ and W−C^1^−C^2^ angles in **3 a** are 115.4(2) and 130.0(3)°, close to the expected value for a sp^2^‐hybridised carbon center. Upon chelation to form **4 a** these values become increasingly distorted away from an ideal geometry with the W−C^1^−O^1^ angle expanding to 141.3(2)° and W−C^1^−C^2^ angle contracting to 99.4(2)° (Figure 1b).

Complex **4 a** reacts with H_2_. Treatment of a benzene solution of **4 a** with H_2_ (1 atm.) at 100 °C for 2 h led to the corresponding F–T type product **5 a** in >95 % NMR yield (Scheme 1). Attempts to crystallise this complex were unsuccessful, however compound **5 a** was characterised by diagnostic resonances at *δ*=4.38 ppm and *δ*=67.9 ppm, in the ^1^H and ^13^C NMR spectra respectively, assigned to the new methylene group formed upon H_2_ addition. Further analysis of the reaction mixtures revealed [W(CO)_6_] and [W(CO)_3_(*η* ^6^‐C_6_D_6_)] as side products of hydrogenation. An isotope labelling experiment in which **4 a** was reacted with D_2_ provided clear evidence for the formation of **5 a**‐D_2_ with the methylene group resonating at *δ*=4.38 ppm in the ^2^H NMR spectrum. Hydrogenation of **3 a** also directly leads to **5 a**,^[21]^ as does the reaction of **2**, H_2_ and benzophenone at 100 °C.

The reaction scope was developed further (Figure 2). A series of C_4_ homologation complexes (**3 b**–**e**) were prepared from the reaction of **2** with CO,^[18]^ 3‐methylbenzaldehyde, 2‐butanone, and 2,6‐dimethylphenyl isocyanide. These reactions proceeded smoothly in all cases demonstrating that C_3_→C_4_ chain growth is possible with a range of electrophiles. Hydrogenation of **3 b**–**e** led to **5 b**–**e** in 60–95 % NMR yield. In the case of **3 e**, the expected hydrogenation product can be observed spectroscopically but isomerizes to a more stable enamine tautomer under the reaction conditions. The reaction is not limited to C_4_ homologation products, as direct hydrogenation of **2** was also possible leading to the formation of the C_3_ analogue **5 f**. Remarkably, **5 b** could also be obtained in 50 % NMR yield from a direct reaction of [W(CO)_6_], **1** and syngas (1 : 1 mixture of H_2_/CO, 1 atm.) after 10 days at 100 °C. While the reaction is slow, this experiment shows that there is self‐organisation in this system as F–T type products to be formed in a single step from simple starting materials.

**Figure 2:**
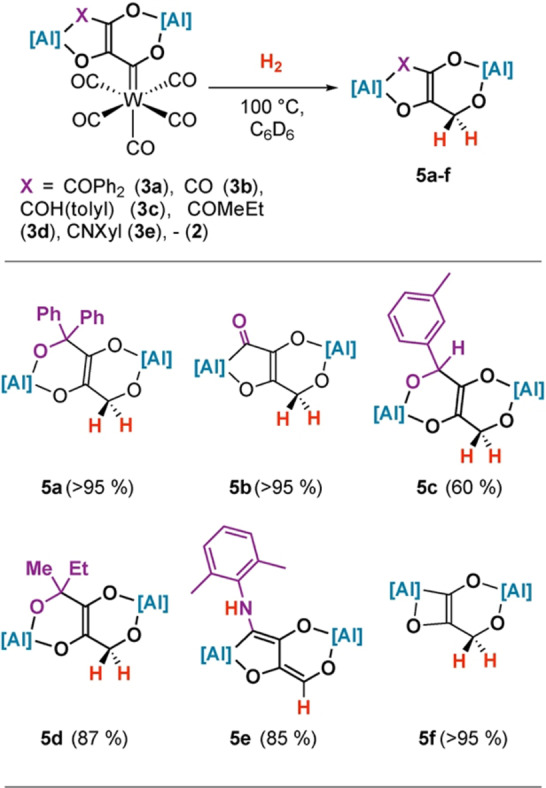
Scope of hydrogenation reaction. Yield determined by ^1^H NMR spectroscopy using 1,3,5‐trimethoxybenzene as external standard.

Further experiments and calculations were undertaken to gain insight into the key hydrogenation step. The reaction of **3 a** with H_2_ (1 atm.) in benzene‐*d_6_* at 100 °C was monitored as a function of time by in situ ^1^H NMR spectroscopy. Kinetic data show that hydrogenation of **3 a** occurs as consecutive reactions with **4 a** as intermediate (Supporting Information, Figure S6). Hydrogenation of **4 a** was also monitored by ^1^H NMR spectroscopy. Kinetic data could be fitted to pseudo‐1^st^ order decay of [**4 a**]. The rate constant for the H_2_ reaction was found to be *k*_obs_(H_2_)=6.28 (±0.06)×10^−4^ s^−1^ (Figure 3). Side‐by‐side kinetic runs with H_2_ and D_2_ gave a KIE of 1.02 (±0.01).

**Figure 3:**
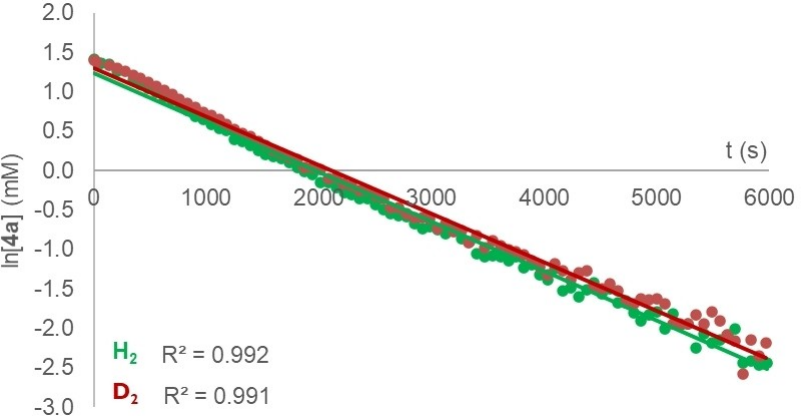
Ln[**4 a**] versus time plot for reaction of **4 a** with H_2_ (green) and D_2_ (red); [**4 a**]_0_=4.6 mM.

A series of plausible pathways for the hydrogenation reaction were calculated by DFT. The lowest energy pathway is depicted in Figure 4. The calculated mechanism is initiated by *η* ^2^‐dihydrogen coordination to complex **4 a** to give **Int‐1**.[^22^, ^23^, ^24^, ^25^, ^26^] Formation of this intermediate occurs via an interchange mechanism (Δ*G* ^≠^_298K_=+21.7 kcal mol^−1^). Oxidative addition of H_2_ to the W centre from **Int‐1** gives the dihydride intermediate **Int‐2** via a low energy barrier transition state **TS‐2** followed by a barrierless migration of one of the hydrides to the carbon atom of the metallocarbene to give **Int‐3**. There is precedent for this type of 1,2‐migration involving hydride and metallocarbene ligands.[^27^, ^28^, ^29^] Prior calculations are consistent with a low energy process.[^30^, ^31^, ^32^] **Int‐3** is stabilised by an agostic interaction of the newly formed C−H bond to W.^[33]^ After two consecutive rotations steps via **TS‐4** and **TS‐5**, **Int‐3** leads to **Int‐5** which is stabilised by coordination of an oxygen atom of the carbon chain. **Int‐5** dissociates a CO ligand through **TS‐6** to give **Int‐6. Int‐6** then rotates again through **Int‐7** to **Int‐8** which is preorganised for reductive elimination via **TS‐9** to afford the thermodynamically stabilised **Int‐9**, a precursor of the final products.^[34]^ The barrier for the reductive elimination step is low (Δ*G* ^≠^_298K_=+11.7 kcal mol^−1^).

**Figure 4:**
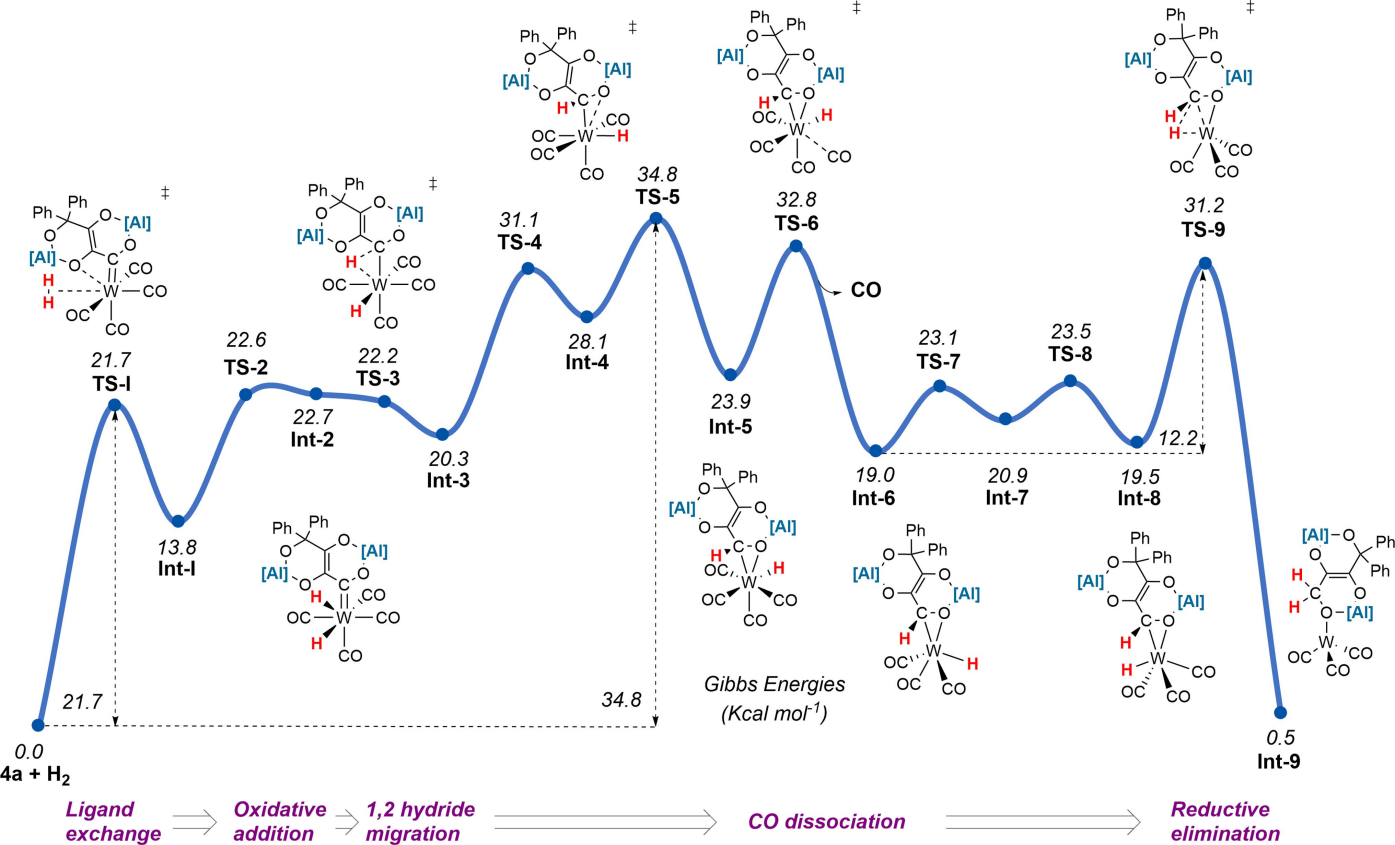
DFT‐calculated mechanism for hydrogenation of **4 a**.

Overall, this calculated mechanism proceeds via a series of established fundamental steps of organometallic compounds namely: i) ligand substitution, ii) oxidative addition, iii) migratory insertion, and iv) reductive elimination. The Gibbs activation energy corresponds to an energy span from **4 a** to **TS‐5** (Δ*G* ^≠^_298K_=+34.8 kcal mol^−1^).^[35]^ The rate‐limiting sequence involves coordination of H_2_, oxidative addition of H_2_, hydride migration from W to C and CO dissociation. The predicted pathway is consistent across a series of DFT functionals.

Consideration of the calculated reaction mechanism suggests that the assignment of a KIE in this system is complex. Although the experimentally determined KIE of 1.02 (±0.01) could be interpreted as a simple step not involving hydrogen atoms, based on the calculations it more likely arises from the combination of individual KIEs (or EIEs) from a series of steps. While oxidative addition of H_2_ to W is expected to show a normal primary KIE, H_2_ binding often occurs with an inverse IE.^[36]^ Similarly, based on the stretching vibrational modes, hydride migration from W to C might be expected to occur with an inverse KIE.^[37]^

In summary, we report the formation of F–T type products from the combination of H_2_, CO, organic electrophiles, and a main group reductant. The reaction scope allows the generation of both C_3_ and C_4_ chains with complete selectively. The hydrogenation step is mediated by the transition metal which likely plays a key role through activation of H_2_ at a site adjacent to a metallocarbene ligand. These findings greatly expand the scope and understanding of reactivity for homogeneous systems reported that combine H_2_ and CO to make hydrogenated carbon chains.

Crystal data is available through the CCDC.^[38]^ Primary data (.mnova, .txt and .xyz) are available from Imperial's Research Data Repository and available through the following link: 10.14469/hpc/10042.

## Author Contributions

M.B. and R.Y.K. carried out the experimental work. M.B. carried out the calculations. A.J.P.W. and R.Y.K. performed the crystallography. R.Y.K. designed the initial experiments and M.B. advanced the ideas. M.B. and M.R.C. wrote the manuscript with feedback from all authors. All authors have given approval to the final version of the manuscript.

## Conflict of interest

The authors declare no conflict of interest.

## Supporting information

As a service to our authors and readers, this journal provides supporting information supplied by the authors. Such materials are peer reviewed and may be re‐organized for online delivery, but are not copy‐edited or typeset. Technical support issues arising from supporting information (other than missing files) should be addressed to the authors.

Supporting Information

Supporting Information

Supporting Information

Supporting Information

## Data Availability

The data that support the findings of this study are openly available in Imperial′s Research Data Repository at 10.14469/hpc/10042, reference number 10042.
