# Supplementary material for: Functionalization and Hydrogenation of Carbon Chains Derived from CO
Source: Angew Chem Int Ed Engl. 2022 Mar 16;61(20):e202202241. doi: 10.1002/anie.202202241 (PMC9311202; doi:10.1002/anie.202202241)

checkCIF/PLATON report

Structure factors have been supplied for datablock(s) 3a, 4a, 5c, 5d, S1, S2

THIS REPORT IS FOR GUIDANCE ONLY. IF USED AS PART OF A REVIEW PROCEDURE FOR PUBLICATION, IT SHOULD NOT REPLACE THE EXPERTISE OF AN EXPERIENCED CRYSTALLOGRAPHIC REFEREE.

No syntax errors found. CIF dictionary Interpreting this report

Datablock: 3a

Bond precision: C-C = 0.0067 A Wavelength=0.71073

Cell:	a=13.1875(3)	b=16.3826(5)	c=18.6982(5)
	alpha=99.300(2)	beta=99.5985(19)	gamma=92.952(2)
Temperature:	173 K		

	Calculated	Reported
Volume	3917.96(19)	3917.95(17)
Space group	P -1	P -1
Hall group	-P 1	-P 1
Moiety formula	C79 H92 Al2 N4 O9 W, 0.739(C7 H8), 0.261(C5 H12)	C79 H92 Al2 N4 O9 W, 0.74(C7 H8), 0.26(C5 H12)
Sum formula	C85.48 H101.04 Al2 N4 O9 W	C85.48 H101.04 Al2 N4 O9 W
Mr	1566.29	1566.31
Dx,g cm-3	1.328	1.328
Z	2	2
Mu (mm-1)	1.556	1.556
F000	1627.8	1628.0
F000’	1626.93	
h,k,lmax	17,21,24	17,21,23
Nref	19389	15462
Tmin,Tmax	0.662,0.817	0.705,0.856
Tmin’	0.427	

Correction method= # Reported T Limits: Tmin=0.705 Tmax=0.856
AbsCorr = ANALYTICAL

Data completeness= 0.797 Theta(max)= 28.249

R(reflections)= 0.0429(12739) wR2(reflections)= 0.1025(15462)

S = 1.057 Npar= 981

The following ALERTS were generated. Each ALERT has the format
test-name_ALERT_alert-type_alert-level.
Click on the hyperlinks for more details of the test.

Alert level B

```
PLAT910_ALERT_3_B Missing # of FCF Reflection(s) Below Theta(Min).         13 Note
```

Alert level C

```
PLAT220_ALERT_2_C NonSolvent   Resd 1  C   Ueq(max)/Ueq(min) Range        3.5 Ratio
PLAT234_ALERT_4_C Large Hirshfeld Difference C81      --C87      .       0.16 Ang.
PLAT242_ALERT_2_C Low    'MainMol' Ueq as Compared to Neighbors of        C36 Check
PLAT260_ALERT_2_C Large Average Ueq of Residue Including       C81      0.107 Check
PLAT911_ALERT_3_C Missing FCF Refl Between Thmin & STh/L=    0.600        179 Report
PLAT971_ALERT_2_C Check Calcd Resid. Dens.  1.00A   From W1              2.04 eA-3
PLAT975_ALERT_2_C Check Calcd Resid. Dens.  0.96A   From O2              0.92 eA-3
PLAT975_ALERT_2_C Check Calcd Resid. Dens.  1.00A   From O3              0.89 eA-3
PLAT977_ALERT_2_C Check Negative Difference Density on H83              -0.35 eA-3
PLAT977_ALERT_2_C Check Negative Difference Density on H93B             -0.38 eA-3
```

Alert level G

```
PLAT002_ALERT_2_G Number of Distance or Angle Restraints on AtSite          5 Note
PLAT003_ALERT_2_G Number of Uiso or Uij Restrained non-H Atoms ...         22 Report
PLAT042_ALERT_1_G Calc. and Reported Moiety Formula Strings Differ     Please Check
PLAT068_ALERT_1_G Reported F000 Differs from Calcd (or Missing)...     Please Check
PLAT152_ALERT_1_G The Supplied and Calc. Volume s.u. Differ by ...          2 Units
PLAT172_ALERT_4_G The CIF-Embedded .res File Contains DFIX Records          1 Report
PLAT176_ALERT_4_G The CIF-Embedded .res File Contains SADI Records          2 Report
PLAT178_ALERT_4_G The CIF-Embedded .res File Contains SIMU Records          3 Report
PLAT301_ALERT_3_G Main Residue  Disorder .............(Resd  1  )          6% Note
PLAT302_ALERT_4_G Anion/Solvent/Minor-Residue Disorder (Resd  2  )       100% Note
PLAT302_ALERT_4_G Anion/Solvent/Minor-Residue Disorder (Resd  3  )       100% Note
PLAT304_ALERT_4_G Non-Integer Number of Atoms in ..... (Resd  2  )      11.09 Check
PLAT304_ALERT_4_G Non-Integer Number of Atoms in ..... (Resd  3  )       4.44 Check
PLAT411_ALERT_2_G Short Inter H...H Contact  H19      ..H92B     .       2.14 Ang.
                                                      x,y,z  =      1_555 Check
PLAT412_ALERT_2_G Short Intra XH3 .. XHn     H43A     ..H66C     .       2.11 Ang.
                                                      x,y,z  =      1_555 Check
PLAT413_ALERT_2_G Short Inter XH3 .. XHn     H64C     ..H93A     .       2.01 Ang.
                                                1-x,1-y,1-z  =      2_666 Check
PLAT773_ALERT_2_G Check long C-C Bond in CIF: C28      --C133            1.72 Ang.
PLAT860_ALERT_3_G Number of Least-Squares Restraints .............        149 Note
PLAT912_ALERT_4_G Missing # of FCF Reflections Above STh/L=  0.600       3652 Note
PLAT933_ALERT_2_G Number of OMIT Records in Embedded .res File ...          3 Note
PLAT941_ALERT_3_G Average HKL Measurement Multiplicity ...........        1.5 Low
PLAT978_ALERT_2_G Number C-C Bonds with Positive Residual Density.          2 Info
```

0 **ALERT level A** = Most likely a serious problem - resolve or explain
1 **ALERT level B** = A potentially serious problem, consider carefully
10 **ALERT level C** = Check. Ensure it is not caused by an omission or oversight
22 **ALERT level G** = General information/check it is not something unexpected

```
 3 ALERT type 1 CIF construction/syntax error, inconsistent or missing data
16 ALERT type 2 Indicator that the structure model may be wrong or deficient
 5 ALERT type 3 Indicator that the structure quality may be low
 9 ALERT type 4 Improvement, methodology, query or suggestion
 0 ALERT type 5 Informative message, check
```

Datablock: 4a

Bond precision: C-C = 0.0082 A Wavelength=1.54184

Cell:	a=13.5044(3)	b=42.6353(10)	c=14.7867(5)
	alpha=90	beta=103.943(3)	gamma=90
Temperature:	173 K		

	Calculated	Reported
Volume	8262.8(4)	8262.8(4)
Space group	P 21/n	P 1 21/n 1
Hall group	-P 2yn	-P 2yn
Moiety formula	4(C77 H89 Al2 N4 O8 W), 3(C7 H16), 4(C H3)	C78 H92 Al2 N4 O8 W, 0.75(C7 H16)
Sum formula	C333 H416 Al8 N16 O32 W4	C83.25 H104 Al2 N4 O8 W
Mr	6106.04	1526.51
Dx,g cm-3	1.227	1.227
Z	1	4
Mu (mm-1)	3.215	3.215
F000	3182.0	3182.0
F000’	3169.40	
h,k,lmax	16,53,18	16,52,18
Nref	16672	15844
Tmin,Tmax	0.679,0.786	0.472,0.851
Tmin’	0.105	

Correction method= # Reported T Limits: Tmin=0.472 Tmax=0.851
AbsCorr = ANALYTICAL

Data completeness= 0.950 Theta(max)= 73.596

R(reflections)= 0.0513(12208) wR2(reflections)= 0.1423(15844)

S = 1.080 Npar= 989

The following ALERTS were generated. Each ALERT has the format
test-name_ALERT_alert-type_alert-level.
Click on the hyperlinks for more details of the test.

Alert level A

```
PLAT308_ALERT_2_A Single Bonded Metal Atom in Structure  (Unusual)         W1' Check
```

Alert level C

```
PLAT220_ALERT_2_C NonSolvent   Resd 1  C   Ueq(max)/Ueq(min) Range        4.3 Ratio
PLAT220_ALERT_2_C NonSolvent   Resd 1  O   Ueq(max)/Ueq(min) Range        3.7 Ratio
PLAT234_ALERT_4_C Large Hirshfeld Difference N2       --C38      .       0.16 Ang.
PLAT234_ALERT_4_C Large Hirshfeld Difference C32      --C34      .       0.16 Ang.
PLAT234_ALERT_4_C Large Hirshfeld Difference C40      --C41      .       0.20 Ang.
PLAT234_ALERT_4_C Large Hirshfeld Difference C41      --C42      .       0.19 Ang.
PLAT242_ALERT_2_C Low    'MainMol' Ueq as Compared to Neighbors of        C35 Check
PLAT242_ALERT_2_C Low    'MainMol' Ueq as Compared to Neighbors of        C73 Check
PLAT242_ALERT_2_C Low    'MainMol' Ueq as Compared to Neighbors of        C76 Check
PLAT329_ALERT_4_C Carbon Atom Hybridisation Unclear for ..........       C44' Check
PLAT342_ALERT_3_C Low Bond Precision on  C-C Bonds ...............    0.00817 Ang.
PLAT906_ALERT_3_C Large K Value in the Analysis of Variance ......      7.019 Check
PLAT911_ALERT_3_C Missing FCF Refl Between Thmin & STh/L=    0.600        238 Report
PLAT934_ALERT_3_C Number of (Iobs-Icalc)/Sigma(W) > 10 Outliers ..          1 Check
PLAT972_ALERT_2_C Check Calcd Resid. Dens.  0.85A   From W1             -1.51 eA-3
```

Alert level G

```
PLAT002_ALERT_2_G Number of Distance or Angle Restraints on AtSite          9 Note
PLAT003_ALERT_2_G Number of Uiso or Uij Restrained non-H Atoms ...         24 Report
PLAT042_ALERT_1_G Calc. and Reported Moiety Formula Strings Differ     Please Check
PLAT045_ALERT_1_G Calculated and Reported Z Differ by a Factor ...       0.25 Check
PLAT063_ALERT_4_G Crystal Size Possibly too Large for Beam Size ..       0.69 mm
PLAT083_ALERT_2_G SHELXL Second Parameter in WGHT  Unusually Large       7.77 Why ?
PLAT172_ALERT_4_G The CIF-Embedded .res File Contains DFIX Records          2 Report
PLAT178_ALERT_4_G The CIF-Embedded .res File Contains SIMU Records          3 Report
PLAT232_ALERT_2_G Hirshfeld Test Diff (M-X)  W1       --C7       .        9.0 s.u.
PLAT300_ALERT_4_G Atom Site Occupancy of C79       Constrained at         0.5 Check
PLAT300_ALERT_4_G Atom Site Occupancy of C80       Constrained at         0.5 Check
PLAT300_ALERT_4_G Atom Site Occupancy of C81       Constrained at         0.5 Check
PLAT300_ALERT_4_G Atom Site Occupancy of C82       Constrained at         0.5 Check
PLAT300_ALERT_4_G Atom Site Occupancy of C83       Constrained at         0.5 Check
PLAT300_ALERT_4_G Atom Site Occupancy of C84       Constrained at         0.5 Check
PLAT300_ALERT_4_G Atom Site Occupancy of C85       Constrained at         0.5 Check
PLAT300_ALERT_4_G Atom Site Occupancy of H79A      Constrained at         0.5 Check
PLAT300_ALERT_4_G Atom Site Occupancy of H79B      Constrained at         0.5 Check
PLAT300_ALERT_4_G Atom Site Occupancy of H79C      Constrained at         0.5 Check
PLAT300_ALERT_4_G Atom Site Occupancy of H80A      Constrained at         0.5 Check
PLAT300_ALERT_4_G Atom Site Occupancy of H80B      Constrained at         0.5 Check
PLAT300_ALERT_4_G Atom Site Occupancy of H81A      Constrained at         0.5 Check
PLAT300_ALERT_4_G Atom Site Occupancy of H81B      Constrained at         0.5 Check
PLAT300_ALERT_4_G Atom Site Occupancy of H82A      Constrained at         0.5 Check
PLAT300_ALERT_4_G Atom Site Occupancy of H82B      Constrained at         0.5 Check
PLAT300_ALERT_4_G Atom Site Occupancy of H83A      Constrained at         0.5 Check
PLAT300_ALERT_4_G Atom Site Occupancy of H83B      Constrained at         0.5 Check
PLAT300_ALERT_4_G Atom Site Occupancy of H84A      Constrained at         0.5 Check
```

```
PLAT300_ALERT_4_G Atom Site Occupancy of H84B       Constrained at        0.5 Check
PLAT300_ALERT_4_G Atom Site Occupancy of H85A       Constrained at        0.5 Check
PLAT300_ALERT_4_G Atom Site Occupancy of H85B       Constrained at        0.5 Check
PLAT300_ALERT_4_G Atom Site Occupancy of H85C       Constrained at        0.5 Check
PLAT300_ALERT_4_G Atom Site Occupancy of C86        Constrained at       0.25 Check
PLAT300_ALERT_4_G Atom Site Occupancy of C87        Constrained at       0.25 Check
PLAT300_ALERT_4_G Atom Site Occupancy of C88        Constrained at       0.25 Check
PLAT300_ALERT_4_G Atom Site Occupancy of C89        Constrained at       0.25 Check
PLAT300_ALERT_4_G Atom Site Occupancy of C90        Constrained at       0.25 Check
PLAT300_ALERT_4_G Atom Site Occupancy of C91        Constrained at       0.25 Check
PLAT300_ALERT_4_G Atom Site Occupancy of C92        Constrained at       0.25 Check
PLAT300_ALERT_4_G Atom Site Occupancy of H86A       Constrained at       0.25 Check
PLAT300_ALERT_4_G Atom Site Occupancy of H86B       Constrained at       0.25 Check
PLAT300_ALERT_4_G Atom Site Occupancy of H86C       Constrained at       0.25 Check
PLAT300_ALERT_4_G Atom Site Occupancy of H87A       Constrained at       0.25 Check
PLAT300_ALERT_4_G Atom Site Occupancy of H87B       Constrained at       0.25 Check
PLAT300_ALERT_4_G Atom Site Occupancy of H88A       Constrained at       0.25 Check
PLAT300_ALERT_4_G Atom Site Occupancy of H88B       Constrained at       0.25 Check
PLAT300_ALERT_4_G Atom Site Occupancy of H89A       Constrained at       0.25 Check
PLAT300_ALERT_4_G Atom Site Occupancy of H89B       Constrained at       0.25 Check
PLAT300_ALERT_4_G Atom Site Occupancy of H90A       Constrained at       0.25 Check
PLAT300_ALERT_4_G Atom Site Occupancy of H90B       Constrained at       0.25 Check
PLAT300_ALERT_4_G Atom Site Occupancy of H91A       Constrained at       0.25 Check
PLAT300_ALERT_4_G Atom Site Occupancy of H91B       Constrained at       0.25 Check
PLAT300_ALERT_4_G Atom Site Occupancy of H92A       Constrained at       0.25 Check
PLAT300_ALERT_4_G Atom Site Occupancy of H92B       Constrained at       0.25 Check
PLAT300_ALERT_4_G Atom Site Occupancy of H92C       Constrained at       0.25 Check
PLAT301_ALERT_3_G Main Residue  Disorder .............(Resd  1  )         13% Note
PLAT302_ALERT_4_G Anion/Solvent/Minor-Residue Disorder (Resd  2  )       100% Note
PLAT302_ALERT_4_G Anion/Solvent/Minor-Residue Disorder (Resd  3  )       100% Note
PLAT302_ALERT_4_G Anion/Solvent/Minor-Residue Disorder (Resd  4  )       100% Note
PLAT302_ALERT_4_G Anion/Solvent/Minor-Residue Disorder (Resd  5  )       100% Note
PLAT304_ALERT_4_G Non-Integer Number of Atoms in ..... (Resd  2  )      11.50 Check
PLAT304_ALERT_4_G Non-Integer Number of Atoms in ..... (Resd  3  )       5.75 Check
PLAT304_ALERT_4_G Non-Integer Number of Atoms in ..... (Resd  4  )       2.33 Check
PLAT304_ALERT_4_G Non-Integer Number of Atoms in ..... (Resd  5  )       1.67 Check
PLAT412_ALERT_2_G Short Intra XH3 .. XHn     H28      ..H33A     .       1.97 Ang.
                                                     x,y,z  =      1_555 Check
PLAT412_ALERT_2_G Short Intra XH3 .. XHn     H45A     ..H75B     .       2.05 Ang.
                                                     x,y,z  =      1_555 Check
PLAT412_ALERT_2_G Short Intra XH3 .. XHn     H48C     ..H42'     .       2.04 Ang.
                                                     x,y,z  =      1_555 Check
PLAT413_ALERT_2_G Short Inter XH3 .. XHn     H21A     ..H46C     .       2.11 Ang.
                                                     x,y,z  =      1_555 Check
PLAT773_ALERT_2_G Check long C-C Bond in CIF: C44      --C46             1.76 Ang.
PLAT773_ALERT_2_G Check long C-C Bond in CIF: C44'     --C45'            1.91 Ang.
PLAT773_ALERT_2_G Check long C-C Bond in CIF: C44'     --C46'            1.73 Ang.
PLAT789_ALERT_4_G Atoms with Negative _atom_site_disorder_group  #         23 Check
PLAT790_ALERT_4_G Centre of Gravity not Within Unit Cell: Resd.  #          2 Note
              C7 H16
PLAT794_ALERT_5_G Tentative Bond Valency for Al2       (III)     .       3.01 Info
PLAT860_ALERT_3_G Number of Least-Squares Restraints ............          84 Note
PLAT883_ALERT_1_G No Info/Value for _atom_sites_solution_primary .     Please Do !
PLAT910_ALERT_3_G Missing # of FCF Reflection(s) Below Theta(Min).          3 Note
PLAT912_ALERT_4_G Missing # of FCF Reflections Above STh/L=  0.600        587 Note
PLAT913_ALERT_3_G Missing # of Very Strong Reflections in FCF ....          1 Note
PLAT933_ALERT_2_G Number of OMIT Records in Embedded .res File ...         20 Note
```

```
PLAT941_ALERT_3_G Average HKL Measurement Multiplicity ...........        1.8 Low
PLAT978_ALERT_2_G Number C-C Bonds with Positive Residual Density.          3 Info
```

```
   1 ALERT level A = Most likely a serious problem - resolve or explain
   0 ALERT level B = A potentially serious problem, consider carefully
  15 ALERT level C = Check. Ensure it is not caused by an omission or oversight
  82 ALERT level G = General information/check it is not something unexpected

   3 ALERT type 1 CIF construction/syntax error, inconsistent or missing data
  20 ALERT type 2 Indicator that the structure model may be wrong or deficient
   9 ALERT type 3 Indicator that the structure quality may be low
  65 ALERT type 4 Improvement, methodology, query or suggestion
   1 ALERT type 5 Informative message, check
```

Datablock: 5c

Bond precision: C-C = 0.0091 A Wavelength=1.54184

Cell:	a=30.6403(15)	b=21.7168(5)	c=23.4001(12)
	alpha=90	beta=98.775(6)	gamma=90
Temperature:	173 K		

	Calculated	Reported
Volume	15388.4(12)	15388.4(12)
Space group	I 2/a	I 2/a
Hall group	-I 2ya	-I 2ya
Moiety formula	C74 H90 Al2 N4 O9 W [+ solvent]	C74 H90 Al2 N4 O9 W, 0.75(C5 H12)
Sum formula	C74 H90 Al2 N4 O9 W [+ solvent]	C77.75 H99 Al2 N4 O9 W
Mr	1417.31	1471.41
Dx,g cm-3	1.224	1.270
Z	8	8
Mu (mm-1)	3.425	3.443
F000	5872.0	6124.0
F000'	5845.94	
h,k,lmax	38,27,29	37,26,28
Nref	15627	14796
Tmin,Tmax	0.413,0.880	0.528,0.889
Tmin'	0.237	

Correction method= # Reported T Limits: Tmin=0.528 Tmax=0.889
AbsCorr = ANALYTICAL

Data completeness= 0.947 Theta(max)= 74.085

R(reflections)= 0.0503(8502) wR2(reflections)= 0.1461(14796)

S = 0.985 Npar= 882

The following ALERTS were generated. Each ALERT has the format
test-name_ALERT_alert-type_alert-level.
Click on the hyperlinks for more details of the test.

Alert level C

```
PLAT213_ALERT_2_C Atom O16              has ADP max/min Ratio .....        3.1 prolat
PLAT220_ALERT_2_C NonSolvent   Resd 1   C   Ueq(max)/Ueq(min) Range        4.0 Ratio
PLAT230_ALERT_2_C Hirshfeld Test Diff for    O8        --C7       .        6.0 s.u.
PLAT234_ALERT_4_C Large Hirshfeld Difference W1        --C19      .       0.17 Ang.
PLAT242_ALERT_2_C Low    'MainMol' Ueq as Compared to Neighbors of          C7 Check
PLAT242_ALERT_2_C Low    'MainMol' Ueq as Compared to Neighbors of         C32 Check
PLAT242_ALERT_2_C Low    'MainMol' Ueq as Compared to Neighbors of         C35 Check
PLAT242_ALERT_2_C Low    'MainMol' Ueq as Compared to Neighbors of         C47 Check
PLAT242_ALERT_2_C Low    'MainMol' Ueq as Compared to Neighbors of         C77 Check
PLAT342_ALERT_3_C Low Bond Precision on  C-C Bonds ..............      0.00909 Ang.
PLAT905_ALERT_3_C Negative K value in the Analysis of Variance ...      -4.262 Report
PLAT911_ALERT_3_C Missing FCF Refl Between Thmin & STh/L=    0.600         146 Report
```

Alert level G

```
FORMU01_ALERT_2_G  There is a discrepancy between the atom counts in the
            _chemical_formula_sum and the formula from the _atom_site* data.
            Atom count from _chemical_formula_sum:C77.75 H99 Al2 N4 O9 W1
            Atom count from the _atom_site data:  C74 H90 Al2 N4 O9 W1
CELLZ01_ALERT_1_G Difference between formula and atom_site contents detected.
CELLZ01_ALERT_1_G ALERT: Large difference may be due to a
            symmetry error - see SYMMG tests
           From the CIF: _cell_formula_units_Z    8
           From the CIF: _chemical_formula_sum  C77.75 H99 Al2 N4 O9 W
           TEST: Compare cell contents of formula and atom_site data

           atom    Z*formula  cif sites diff
           C        622.00    592.00   30.00
           H        792.00    720.00   72.00
           Al        16.00     16.00    0.00
           N         32.00     32.00    0.00
           O         72.00     72.00    0.00
           W          8.00      8.00    0.00
PLAT002_ALERT_2_G Number of Distance or Angle Restraints on AtSite          23 Note
PLAT003_ALERT_2_G Number of Uiso or Uij Restrained non-H Atoms ...          22 Report
PLAT041_ALERT_1_G Calc. and Reported SumFormula    Strings  Differ     Please Check
PLAT128_ALERT_4_G Alternate Setting for Input Space Group  I2/a          I2/c Note
PLAT175_ALERT_4_G The CIF-Embedded .res File Contains SAME Records           1 Report
PLAT176_ALERT_4_G The CIF-Embedded .res File Contains SADI Records           1 Report
PLAT178_ALERT_4_G The CIF-Embedded .res File Contains SIMU Records           1 Report
PLAT301_ALERT_3_G Main Residue  Disorder .............(Resd  1  )          12% Note
PLAT606_ALERT_4_G Solvent Accessible VOID(S) in Structure ........           ! Info
```

```
PLAT793_ALERT_4_G Model has Chirality at C2          (Centro SPGR)          R Verify
PLAT794_ALERT_5_G Tentative Bond Valency for Al1       (III)     .       3.04 Info
PLAT794_ALERT_5_G Tentative Bond Valency for Al2       (III)     .       3.13 Info
PLAT860_ALERT_3_G Number of Least-Squares Restraints ............         110 Note
PLAT869_ALERT_4_G ALERTS Related to the Use of SQUEEZE Suppressed           ! Info
PLAT883_ALERT_1_G No Info/Value for _atom_sites_solution_primary .     Please Do !
PLAT910_ALERT_3_G Missing # of FCF Reflection(s) Below Theta(Min).           3 Note
PLAT912_ALERT_4_G Missing # of FCF Reflections Above STh/L=  0.600         659 Note
PLAT941_ALERT_3_G Average HKL Measurement Multiplicity ...........         1.6 Low
PLAT978_ALERT_2_G Number C-C Bonds with Positive Residual Density.           0 Info
```

```
   0 ALERT level A = Most likely a serious problem - resolve or explain
   0 ALERT level B = A potentially serious problem, consider carefully
  12 ALERT level C = Check. Ensure it is not caused by an omission or oversight
  22 ALERT level G = General information/check it is not something unexpected

   4 ALERT type 1 CIF construction/syntax error, inconsistent or missing data
  12 ALERT type 2 Indicator that the structure model may be wrong or deficient
   7 ALERT type 3 Indicator that the structure quality may be low
   9 ALERT type 4 Improvement, methodology, query or suggestion
   2 ALERT type 5 Informative message, check
```

Datablock: 5d

```
Bond precision:   C-C = 0.0123 A          Wavelength=1.54184

Cell:             a=20.4694(5)     b=17.5544(4)       c=20.6800(4)
                  alpha=90         beta=92.606(2)     gamma=90
Temperature:      173 K
```

	Calculated	Reported
Volume	7423.2(3)	7423.2(3)
Space group	P 21/n	P 21/n
Hall group	-P 2yn	-P 2yn
Moiety formula	C70 H90 Al2 N4 O9 W [+ solvent]	C70 H90 Al2 N4 O9 W, C5 H12
Sum formula	C70 H90 Al2 N4 O9 W [+ solvent]	C75 H102 Al2 N4 O9 W
Mr	1369.27	1441.41
Dx,g cm-3	1.225	1.290
Z	4	4
Mu (mm-1)	3.531	3.555
F000	2840.0	3008.0
F000'	2826.70	
h,k,lmax	25,21,25	25,21,25
Nref	14949	20698
Tmin,Tmax	0.445,0.488	0.530,0.635
Tmin'	0.337	

Correction method= # Reported T Limits: Tmin=0.530 Tmax=0.635
AbsCorr = ANALYTICAL

Data completeness= 1.385 Theta(max)= 73.454

R(reflections)= 0.0593(15250) wR2(reflections)= 0.1749(20698)

S = 0.975 Npar= 813

The following ALERTS were generated. Each ALERT has the format
test-name_ALERT_alert-type_alert-level.
Click on the hyperlinks for more details of the test.

Alert level C

```
PLAT220_ALERT_2_C NonSolvent   Resd 1  C   Ueq(max)/Ueq(min) Range        4.8 Ratio
PLAT220_ALERT_2_C NonSolvent   Resd 1  O   Ueq(max)/Ueq(min) Range        3.4 Ratio
PLAT222_ALERT_3_C NonSolvent Resd 1  H   Uiso(max)/Uiso(min) Range        4.4 Ratio
PLAT242_ALERT_2_C Low    'MainMol' Ueq as Compared to Neighbors of        C12 Check
PLAT242_ALERT_2_C Low    'MainMol' Ueq as Compared to Neighbors of        C42 Check
PLAT242_ALERT_2_C Low    'MainMol' Ueq as Compared to Neighbors of        C45 Check
PLAT342_ALERT_3_C Low Bond Precision on  C-C Bonds ...............    0.01226 Ang.
PLAT911_ALERT_3_C Missing FCF Refl Between Thmin & STh/L=    0.600        145 Report
```

Alert level G

```
FORMU01_ALERT_2_G  There is a discrepancy between the atom counts in the
            _chemical_formula_sum and the formula from the _atom_site* data.
            Atom count from _chemical_formula_sum:C75 H102 Al2 N4 O9 W1
```

```
           Atom count from the _atom_site data:  C70 H90 Al2 N4 O9 W1
CELLZ01_ALERT_1_G Difference between formula and atom_site contents detected.
CELLZ01_ALERT_1_G ALERT: Large difference may be due to a
           symmetry error - see SYMMG tests
          From the CIF: _cell_formula_units_Z    4
          From the CIF: _chemical_formula_sum  C75 H102 Al2 N4 O9 W
          TEST: Compare cell contents of formula and atom_site data
```

atom	Z*formula	cif sites	diff
C	300.00	280.00	20.00
H	408.00	360.00	48.00
Al	8.00	8.00	0.00
N	16.00	16.00	0.00
O	36.00	36.00	0.00
W	4.00	4.00	0.00

```
PLAT002_ALERT_2_G Number of Distance or Angle Restraints on AtSite         10 Note
PLAT003_ALERT_2_G Number of Uiso or Uij Restrained non-H Atoms ...          8 Report
PLAT041_ALERT_1_G Calc. and Reported SumFormula    Strings  Differ     Please Check
PLAT176_ALERT_4_G The CIF-Embedded .res File Contains SADI Records          8 Report
PLAT178_ALERT_4_G The CIF-Embedded .res File Contains SIMU Records          1 Report
PLAT232_ALERT_2_G Hirshfeld Test Diff (M-X)  W1       --C69      .        5.5 s.u.
PLAT301_ALERT_3_G Main Residue  Disorder .............(Resd  1  )          5% Note
PLAT343_ALERT_2_G Unusual sp?      Angle Range in Main Residue for        C71 Check
PLAT412_ALERT_2_G Short Intra XH3 .. XHn     H14B     ..H73C     .       1.75 Ang.
                                                       x,y,z  =      1_555 Check
PLAT412_ALERT_2_G Short Intra XH3 .. XHn     H28C     ..H74A     .       2.14 Ang.
                                                       x,y,z  =      1_555 Check
PLAT412_ALERT_2_G Short Intra XH3 .. XHn     H28C     ..H75D     .       2.09 Ang.
                                                       x,y,z  =      1_555 Check
PLAT412_ALERT_2_G Short Intra XH3 .. XHn     H46C     ..H75E     .       2.04 Ang.
                                                       x,y,z  =      1_555 Check
PLAT606_ALERT_4_G Solvent Accessible VOID(S) in Structure ........          ! Info
PLAT793_ALERT_4_G Model has Chirality at C65         (Centro SPGR)          R Verify
PLAT793_ALERT_4_G Model has Chirality at C65'        (Centro SPGR)          S Verify
PLAT794_ALERT_5_G Tentative Bond Valency for Al1       (III)     .       3.02 Info
PLAT860_ALERT_3_G Number of Least-Squares Restraints ............          59 Note
PLAT869_ALERT_4_G ALERTS Related to the Use of SQUEEZE Suppressed           ! Info
PLAT870_ALERT_4_G ALERTS Related to Twinning Effects Suppressed ..          ! Info
PLAT883_ALERT_1_G No Info/Value for _atom_sites_solution_primary .     Please Do !
PLAT910_ALERT_3_G Missing # of FCF Reflection(s) Below Theta(Min).          3 Note
PLAT912_ALERT_4_G Missing # of FCF Reflections Above STh/L=  0.600        413 Note
PLAT931_ALERT_5_G CIFcalcFCF  Twin Law ( 0 0 1)         Est.d BASF       0.41 Check
PLAT941_ALERT_3_G Average HKL Measurement Multiplicity ..........         1.4 Low
```

---

0 **ALERT level A** = Most likely a serious problem - resolve or explain
0 **ALERT level B** = A potentially serious problem, consider carefully
8 **ALERT level C** = Check. Ensure it is not caused by an omission or oversight
27 **ALERT level G** = General information/check it is not something unexpected

4 ALERT type 1 CIF construction/syntax error, inconsistent or missing data
14 ALERT type 2 Indicator that the structure model may be wrong or deficient
7 ALERT type 3 Indicator that the structure quality may be low
8 ALERT type 4 Improvement, methodology, query or suggestion
2 ALERT type 5 Informative message, check

Datablock: S1

Bond precision:	C-C = 0.0099 A	Wavelength=1.54184	
Cell:	a=14.9640(6)	b=21.4758(5)	c=24.2362(8)
	alpha=90	beta=107.909(4)	gamma=90
Temperature:	173 K		

	Calculated	Reported
Volume	7411.3(5)	7411.2(4)
Space group	P 21/c	P 21/c
Hall group	-P 2ybc	-P 2ybc
Moiety formula	C74 H90 Al2 N4 O9 W, C4 H8 O	C74 H90 Al2 N4 O9 W, C4 H8 O
Sum formula	C78 H98 Al2 N4 O10 W	C78 H98 Al2 N4 O10 W
Mr	1489.34	1489.41
Dx,g cm-3	1.335	1.335
Z	4	4
Mu (mm-1)	3.592	3.592
F000	3095.8	3096.0
F000'	3083.27	
h,k,lmax	18,26,30	18,26,29
Nref	14993	14495
Tmin,Tmax	0.410,0.827	0.465,0.837
Tmin'	0.262	

Correction method= # Reported T Limits: Tmin=0.465 Tmax=0.837
AbsCorr = ANALYTICAL

Data completeness= 0.967 Theta(max)= 73.683

R(reflections)= 0.0647(11508) wR2(reflections)= 0.1700(14495)

S = 1.110 Npar= 935

The following ALERTS were generated. Each ALERT has the format
test-name_ALERT_alert-type_alert-level.
Click on the hyperlinks for more details of the test.

Alert level A

PLAT308_ALERT_2_A Single Bonded Metal Atom in Structure (Unusual) W1' Check

Author Response: Difference electron density maps for the structure of S1 suggested the presence of a second orientation of the whole molecule (of ca. 13% occupancy) overlaying the main occupancy orientation in a manner corresponding to a ca. 180 deg rotation about an axis passing through the middle of the complex (along the b axis direction, approximately coincident with the C66-C62 bond). Unsurprisingly, the only atom of this second orientation that could be reliably located was the minor occupancy tungsten atom, W1', which was refined anisotropically.

Alert level C

```
PLAT234_ALERT_4_C Large Hirshfeld Difference C73      --C78      .        0.18 Ang.
PLAT242_ALERT_2_C Low    'MainMol' Ueq as Compared to Neighbors of        C12 Check
PLAT242_ALERT_2_C Low    'MainMol' Ueq as Compared to Neighbors of        C68 Check
PLAT260_ALERT_2_C Large Average Ueq of Residue Including       O80      0.149 Check
PLAT342_ALERT_3_C Low Bond Precision on  C-C Bonds ...............     0.0099 Ang.
PLAT906_ALERT_3_C Large K Value in the Analysis of Variance ......      5.256 Check
PLAT911_ALERT_3_C Missing FCF Refl Between Thmin & STh/L=    0.600         28 Report
PLAT975_ALERT_2_C Check Calcd Resid. Dens.  0.96A   From O60          0.61 eA-3
```

Alert level G

```
CELLZ01_ALERT_1_G Difference between formula and atom_site contents detected.
CELLZ01_ALERT_1_G ALERT: check formula stoichiometry or atom site occupancies.
           From the CIF: _cell_formula_units_Z    4
           From the CIF: _chemical_formula_sum  C78 H98 Al2 N4 O10 W
           TEST: Compare cell contents of formula and atom_site data

           atom    Z*formula  cif sites diff
           C         312.00    311.98    0.02
           H         392.00    391.97    0.03
           Al          8.00      8.00    0.00
           N          16.00     16.00    0.00
           O          40.00     40.00    0.00
           W           4.00      4.00    0.00
PLAT002_ALERT_2_G Number of Distance or Angle Restraints on AtSite         26 Note
PLAT003_ALERT_2_G Number of Uiso or Uij Restrained non-H Atoms ...         31 Report
PLAT068_ALERT_1_G Reported F000 Differs from Calcd (or Missing)...     Please Check
PLAT083_ALERT_2_G SHELXL Second Parameter in WGHT  Unusually Large      20.12 Why ?
PLAT176_ALERT_4_G The CIF-Embedded .res File Contains SADI Records          7 Report
PLAT178_ALERT_4_G The CIF-Embedded .res File Contains SIMU Records          3 Report
PLAT232_ALERT_2_G Hirshfeld Test Diff (M-X)  W1       --C69      .        5.7 s.u.
PLAT232_ALERT_2_G Hirshfeld Test Diff (M-X)  Al1      --O67      .        6.0 s.u.
PLAT232_ALERT_2_G Hirshfeld Test Diff (M-X)  Al2      --C66      .        5.7 s.u.
PLAT301_ALERT_3_G Main Residue  Disorder ..............(Resd  1  )         9% Note
PLAT302_ALERT_4_G Anion/Solvent/Minor-Residue Disorder (Resd  2  )       100% Note
PLAT302_ALERT_4_G Anion/Solvent/Minor-Residue Disorder (Resd  3  )       100% Note
PLAT302_ALERT_4_G Anion/Solvent/Minor-Residue Disorder (Resd  4  )       100% Note
PLAT304_ALERT_4_G Non-Integer Number of Atoms in ..... (Resd  2  )       6.76 Check
PLAT304_ALERT_4_G Non-Integer Number of Atoms in ..... (Resd  3  )       3.93 Check
PLAT304_ALERT_4_G Non-Integer Number of Atoms in ..... (Resd  4  )       2.30 Check
PLAT380_ALERT_4_G Incorrectly? Oriented X(sp2)-Methyl Moiety .....        C79 Check
PLAT398_ALERT_2_G Deviating  C-O-C    Angle From 120 for O80          92.3 Degree
```

```
PLAT398_ALERT_2_G Deviating  C-O-C    Angle From 120 for O80'           94.4 Degree
PLAT398_ALERT_2_G Deviating  C-O-C    Angle From 120 for O80"           91.5 Degree
PLAT411_ALERT_2_G Short Inter H...H Contact  H9A      ..H81C     .       1.48 Ang.
                                                      x,y,z  =     1_555 Check
PLAT411_ALERT_2_G Short Inter H...H Contact  H10A     ..H81E     .       1.75 Ang.
                                                      x,y,z  =     1_555 Check
PLAT412_ALERT_2_G Short Intra XH3 .. XHn     H26C     ..H64A     .       1.93 Ang.
                                                      x,y,z  =     1_555 Check
PLAT412_ALERT_2_G Short Intra XH3 .. XHn     H26C     ..H64B     .       2.00 Ang.
                                                      x,y,z  =     1_555 Check
PLAT413_ALERT_2_G Short Inter XH3 .. XHn     H16A     ..H81F     .       2.04 Ang.
                                                      x,y,z  =     1_555 Check
PLAT413_ALERT_2_G Short Inter XH3 .. XHn     H34B     ..H82B     .       1.95 Ang.
                                              x,3/2-y,1/2+z  =     4_576 Check
PLAT413_ALERT_2_G Short Inter XH3 .. XHn     H58C     ..H84E     .       2.02 Ang.
                                           1-x,-1/2+y,3/2-z  =     2_646 Check
PLAT413_ALERT_2_G Short Inter XH3 .. XHn     H59C     ..H84F     .       1.42 Ang.
                                              x,3/2-y,1/2+z  =     4_576 Check
PLAT432_ALERT_2_G Short Inter X...Y Contact  C9       ..C81'             3.18 Ang.
                                                      x,y,z  =     1_555 Check
PLAT432_ALERT_2_G Short Inter X...Y Contact  C10      ..C81"             3.20 Ang.
                                                      x,y,z  =     1_555 Check
PLAT794_ALERT_5_G Tentative Bond Valency for Al1       (III)     .       3.05 Info
PLAT860_ALERT_3_G Number of Least-Squares Restraints ............         269 Note
PLAT883_ALERT_1_G No Info/Value for _atom_sites_solution_primary .    Please Do !
PLAT910_ALERT_3_G Missing # of FCF Reflection(s) Below Theta(Min).          2 Note
PLAT912_ALERT_4_G Missing # of FCF Reflections Above STh/L=  0.600        457 Note
PLAT933_ALERT_2_G Number of OMIT Records in Embedded .res File ...          2 Note
PLAT941_ALERT_3_G Average HKL Measurement Multiplicity ..........         2.2 Low
PLAT965_ALERT_2_G The SHELXL WEIGHT Optimisation has not Converged    Please Check
PLAT978_ALERT_2_G Number C-C Bonds with Positive Residual Density.          0 Info
```

```
   1 ALERT level A = Most likely a serious problem - resolve or explain
   0 ALERT level B = A potentially serious problem, consider carefully
   8 ALERT level C = Check. Ensure it is not caused by an omission or oversight
  41 ALERT level G = General information/check it is not something unexpected

   4 ALERT type 1 CIF construction/syntax error, inconsistent or missing data
  27 ALERT type 2 Indicator that the structure model may be wrong or deficient
   7 ALERT type 3 Indicator that the structure quality may be low
  11 ALERT type 4 Improvement, methodology, query or suggestion
   1 ALERT type 5 Informative message, check
```

Datablock: S2

```
Bond precision:   C-C = 0.0071 A           Wavelength=0.71073

Cell:           a=13.3538(2)      b=13.8568(4)       c=26.7693(6)
                alpha=75.930(2)   beta=77.1228(16)   gamma=83.7504(18)
Temperature:    173 K
```

	Calculated	Reported
Volume	4676.13(19)	4676.11(19)
Space group	P -1	P -1
Hall group	-P 1	-P 1
Moiety formula	2(C83 H100 Al2 N6 O7 W), C7 H8 [+ solvent]	C83 H100 Al2 N6 O7 W, 0.5(C7 H8)
Sum formula	C173 H208 Al4 N12 O14 W2 [+ solvent]	C86.50 H104 Al2 N6 O7 W
Mr	3155.13	1577.56
Dx,g cm-3	1.120	1.120
Z	1	2
Mu (mm-1)	1.303	1.303
F000	1642.0	1642.0
F000'	1641.07	
h,k,lmax	17,18,35	17,17,35
Nref	23248	18515
Tmin,Tmax	0.459,0.585	0.566,0.648
Tmin'	0.443	

Correction method= # Reported T Limits: Tmin=0.566 Tmax=0.648
AbsCorr = ANALYTICAL

Data completeness= 0.796 Theta(max)= 28.297

R(reflections)= 0.0435(15316) wR2(reflections)= 0.1110(18515)

S = 1.043 Npar= 987

The following ALERTS were generated. Each ALERT has the format
test-name_ALERT_alert-type_alert-level.
Click on the hyperlinks for more details of the test.

Alert level B

```
PLAT220_ALERT_2_B NonSolvent   Resd 1  C   Ueq(max)/Ueq(min) Range        7.0 Ratio
PLAT910_ALERT_3_B Missing # of FCF Reflection(s) Below Theta(Min).         14 Note
```

Alert level C

```
PLAT220_ALERT_2_C NonSolvent   Resd 1  O   Ueq(max)/Ueq(min) Range        4.0 Ratio
PLAT222_ALERT_3_C NonSolvent Resd 1  H   Uiso(max)/Uiso(min) Range        5.1 Ratio
PLAT241_ALERT_2_C High   'MainMol' Ueq as Compared to Neighbors of        C42 Check
PLAT242_ALERT_2_C Low    'MainMol' Ueq as Compared to Neighbors of        C33 Check
PLAT242_ALERT_2_C Low    'MainMol' Ueq as Compared to Neighbors of        C65 Check
PLAT242_ALERT_2_C Low    'MainMol' Ueq as Compared to Neighbors of        C74 Check
PLAT242_ALERT_2_C Low    'MainMol' Ueq as Compared to Neighbors of        C77 Check
PLAT911_ALERT_3_C Missing FCF Refl Between Thmin & STh/L=    0.600        197 Report
PLAT934_ALERT_3_C Number of (Iobs-Icalc)/Sigma(W) > 10 Outliers ..          1 Check
```

Alert level G

```
PLAT002_ALERT_2_G Number of Distance or Angle Restraints on AtSite         10 Note
PLAT003_ALERT_2_G Number of Uiso or Uij Restrained non-H Atoms ...         36 Report
PLAT042_ALERT_1_G Calc. and Reported Moiety Formula Strings Differ     Please Check
PLAT045_ALERT_1_G Calculated and Reported Z Differ by a Factor ...       0.50 Check
PLAT063_ALERT_4_G Crystal Size Possibly too Large for Beam Size ..       0.62 mm
PLAT172_ALERT_4_G The CIF-Embedded .res File Contains DFIX Records          2 Report
PLAT174_ALERT_4_G The CIF-Embedded .res File Contains FLAT Records          1 Report
PLAT176_ALERT_4_G The CIF-Embedded .res File Contains SADI Records          2 Report
PLAT178_ALERT_4_G The CIF-Embedded .res File Contains SIMU Records          3 Report
PLAT301_ALERT_3_G Main Residue  Disorder .............(Resd  1  )         11% Note
PLAT302_ALERT_4_G Anion/Solvent/Minor-Residue Disorder (Resd  2  )       100% Note
PLAT302_ALERT_4_G Anion/Solvent/Minor-Residue Disorder (Resd  3  )       100% Note
PLAT304_ALERT_4_G Non-Integer Number of Atoms in ..... (Resd  2  )       5.26 Check
PLAT304_ALERT_4_G Non-Integer Number of Atoms in ..... (Resd  3  )       2.24 Check
PLAT412_ALERT_2_G Short Intra XH3 .. XHn     H58      ..H63F     .       2.03 Ang.
                                                      x,y,z  =      1_555 Check
PLAT606_ALERT_4_G Solvent Accessible VOID(S) in Structure ........          ! Info
PLAT721_ALERT_1_G Bond    Calc     0.97000, Rep     0.96000 Dev...       0.01 Ang.
              C86'     -H86F            1_555   1_555 ........     #  266 Check
PLAT790_ALERT_4_G Centre of Gravity not Within Unit Cell: Resd.  #          2 Note
              C7 H8
PLAT790_ALERT_4_G Centre of Gravity not Within Unit Cell: Resd.  #          3 Note
              C7 H8
PLAT794_ALERT_5_G Tentative Bond Valency for Al1       (III)     .       3.01 Info
PLAT860_ALERT_3_G Number of Least-Squares Restraints ............         145 Note
PLAT869_ALERT_4_G ALERTS Related to the Use of SQUEEZE Suppressed           ! Info
PLAT912_ALERT_4_G Missing # of FCF Reflections Above STh/L=  0.600       4424 Note
PLAT913_ALERT_3_G Missing # of Very Strong Reflections in FCF ....          1 Note
PLAT933_ALERT_2_G Number of OMIT Records in Embedded .res File ...          6 Note
PLAT941_ALERT_3_G Average HKL Measurement Multiplicity ..........         1.5 Low
PLAT978_ALERT_2_G Number C-C Bonds with Positive Residual Density.          4 Info
```

---

0 **ALERT level A** = Most likely a serious problem - resolve or explain
2 **ALERT level B** = A potentially serious problem, consider carefully
9 **ALERT level C** = Check. Ensure it is not caused by an omission or oversight
27 **ALERT level G** = General information/check it is not something unexpected

3 ALERT type 1 CIF construction/syntax error, inconsistent or missing data
12 ALERT type 2 Indicator that the structure model may be wrong or deficient
8 ALERT type 3 Indicator that the structure quality may be low
14 ALERT type 4 Improvement, methodology, query or suggestion
1 ALERT type 5 Informative message, check

It is advisable to attempt to resolve as many as possible of the alerts in all categories. Often the minor alerts point to easily fixed oversights, errors and omissions in your CIF or refinement strategy, so attention to these fine details can be worthwhile. In order to resolve some of the more serious problems it may be necessary to carry out additional measurements or structure refinements. However, the purpose of your study may justify the reported deviations and the more serious of these should normally be commented upon in the discussion or experimental section of a paper or in the "special_details" fields of the CIF. checkCIF was carefully designed to identify outliers and unusual parameters, but every test has its limitations and alerts that are not important in a particular case may appear. Conversely, the absence of alerts does not guarantee there are no aspects of the results needing attention. It is up to the individual to critically assess their own results and, if necessary, seek expert advice.

Publication of your CIF in IUCr journals

A basic structural check has been run on your CIF. These basic checks will be run on all CIFs submitted for publication in IUCr journals (*Acta Crystallographica*, *Journal of Applied Crystallography*, *Journal of Synchrotron Radiation*); however, if you intend to submit to *Acta Crystallographica Section C* or *E* or *IUCrData*, you should make sure that full publication checks are run on the final version of your CIF prior to submission.

Publication of your CIF in other journals

Please refer to the *Notes for Authors* of the relevant journal for any special instructions relating to CIF submission.

PLATON version of 13/07/2021; check.def file version of 13/07/2021

Datablock 3a - ellipsoid plot

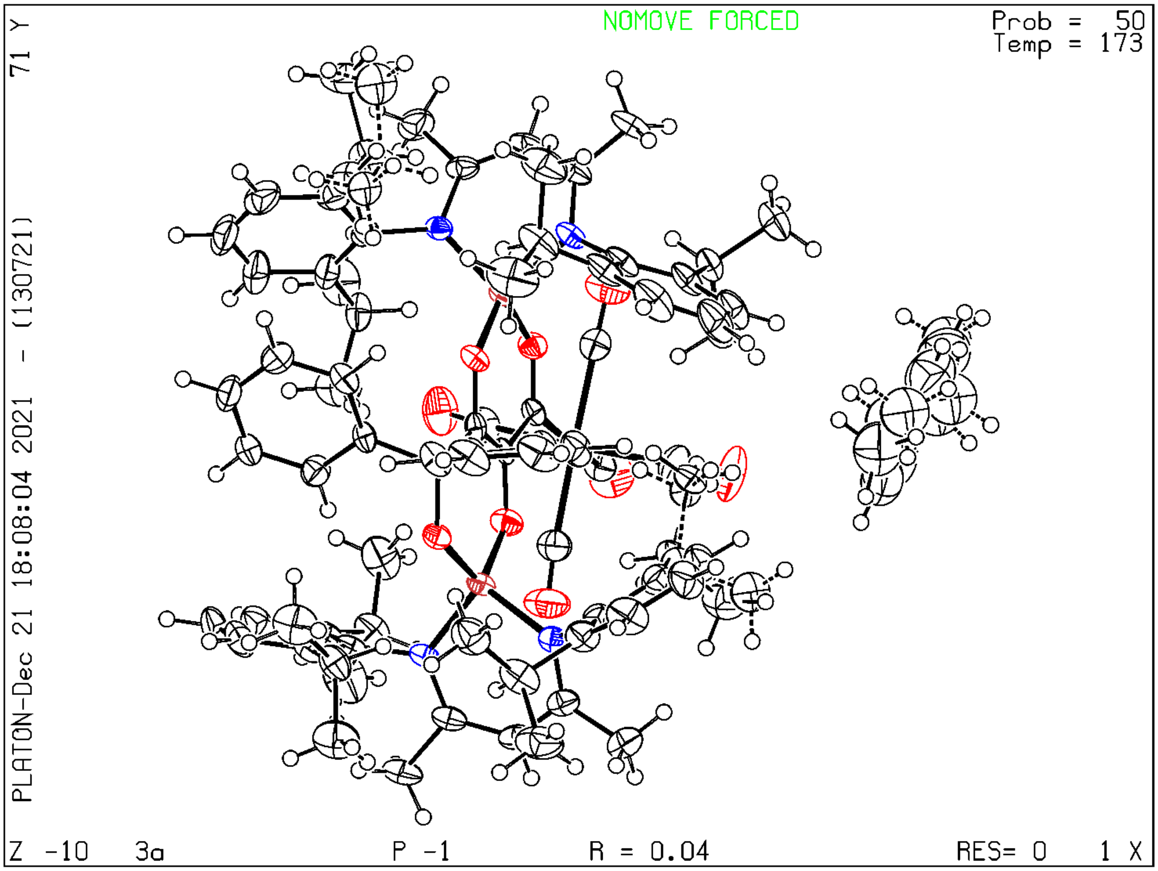

Datablock 4a - ellipsoid plot

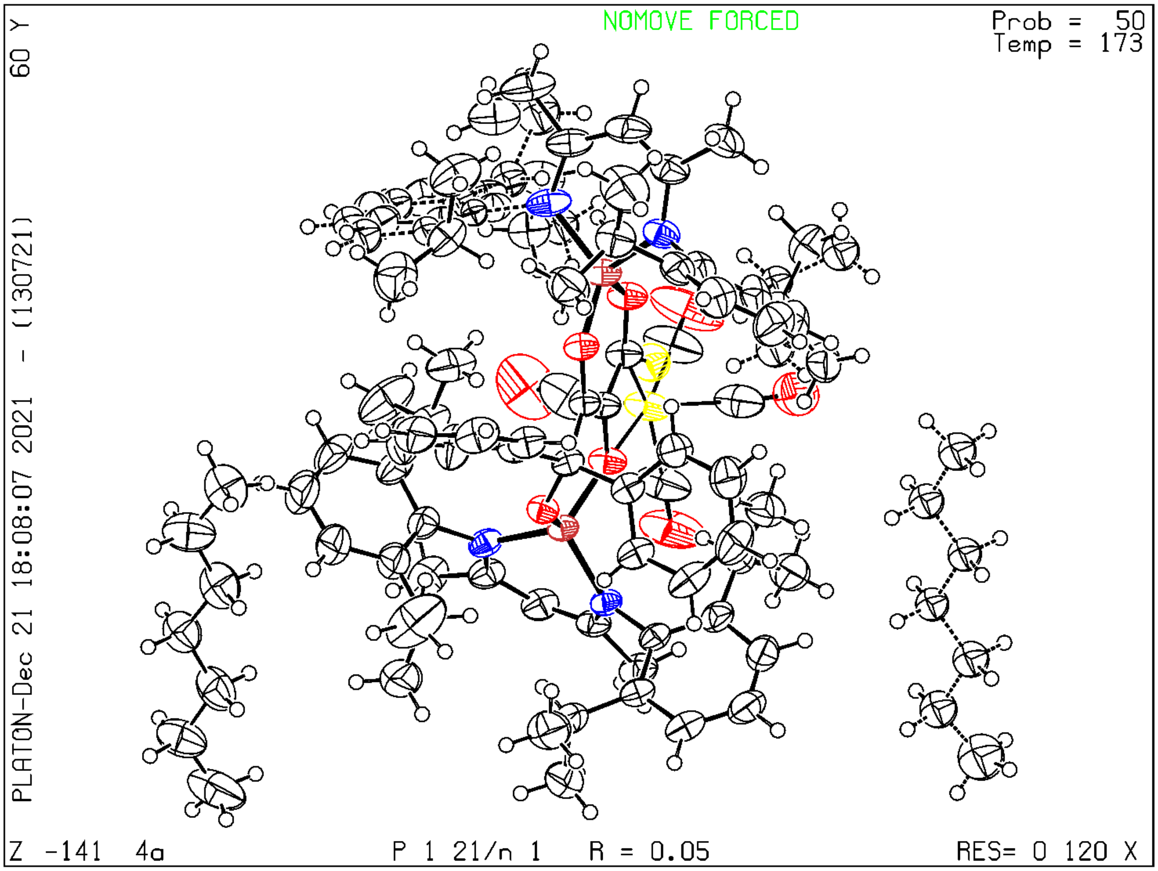

Datablock 5c - ellipsoid plot

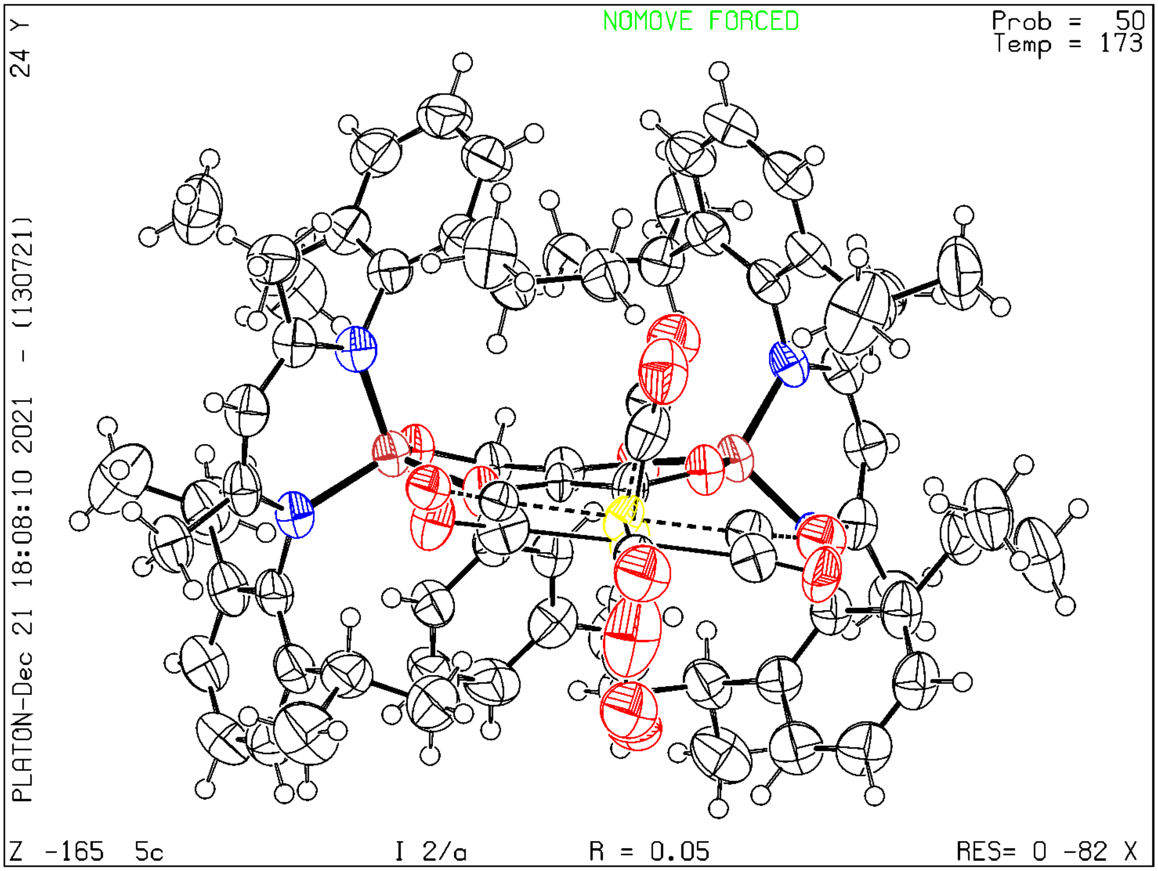

Datablock 5d - ellipsoid plot

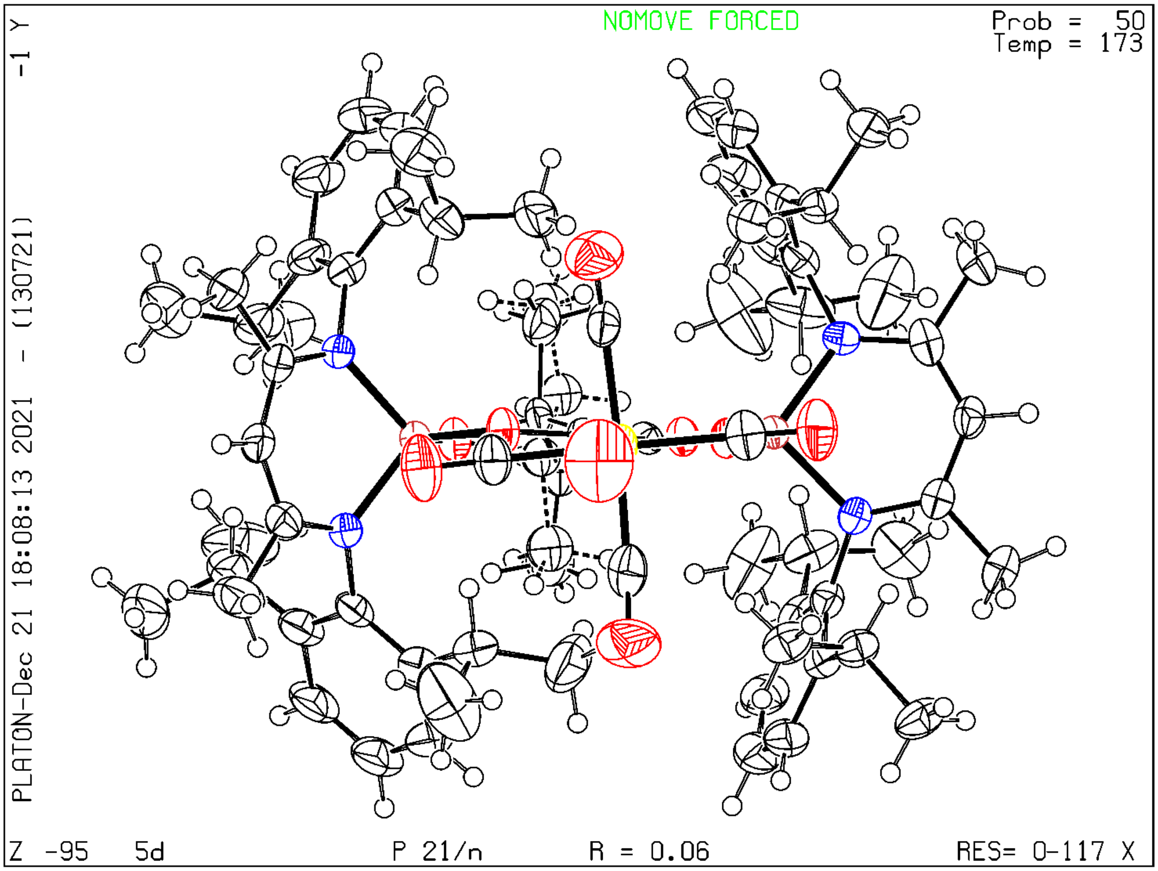

Datablock S1 - ellipsoid plot

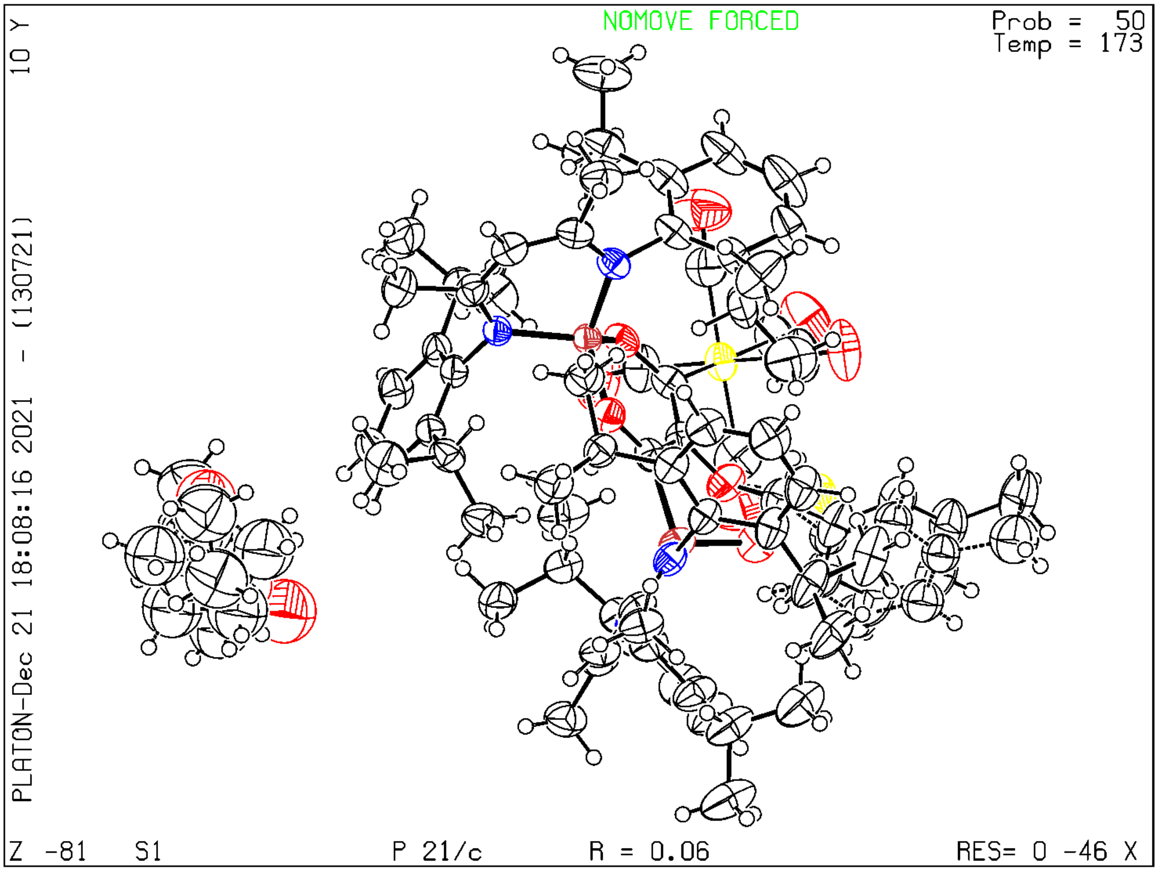

Datablock S2 - ellipsoid plot

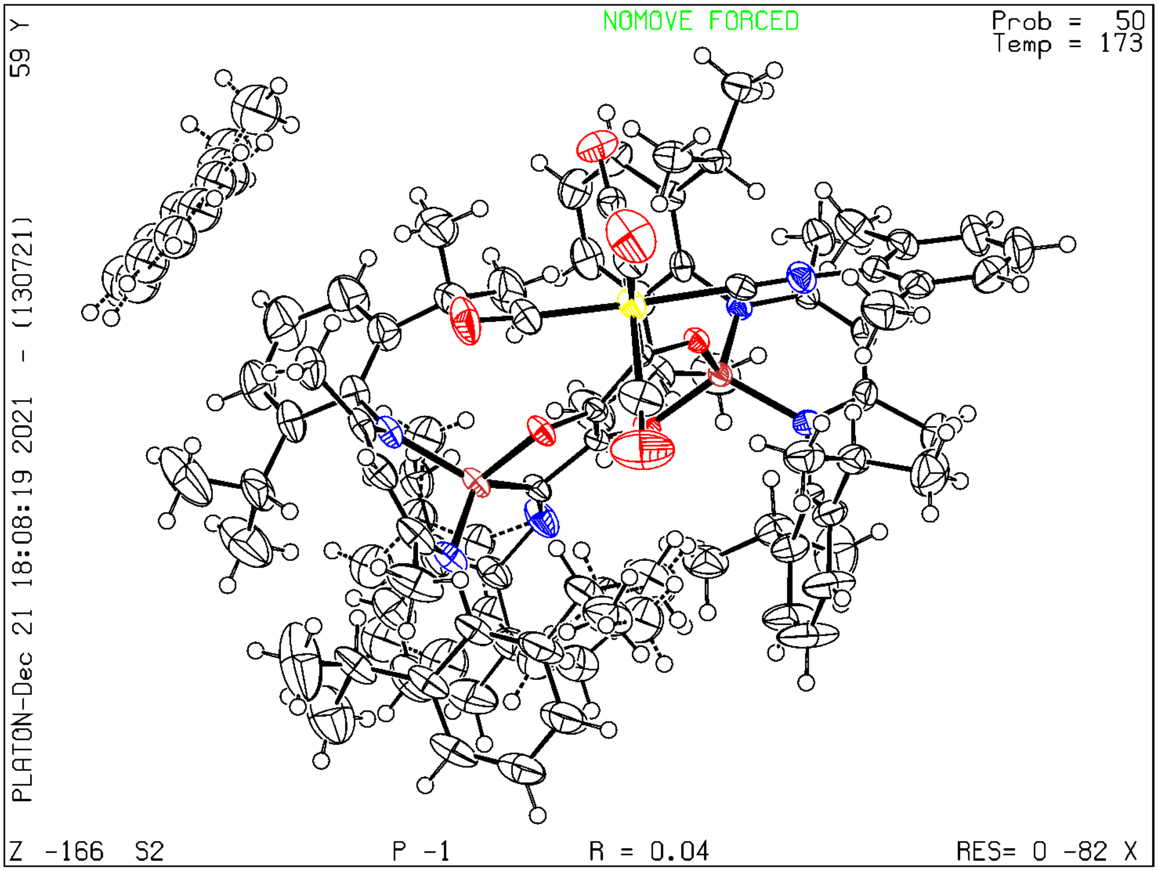

Supplement: Supplementary file 1 — Supporting Information [file ANIE-61-0-s004.pdf]
