# Supplementary material for: Functionalization and Hydrogenation of Carbon Chains Derived from CO
Source: Angew Chem Int Ed Engl. 2022 Mar 16;61(20):e202202241. doi: 10.1002/anie.202202241 (PMC9311202; doi:10.1002/anie.202202241)
Supplement: Supplementary file 4 — Supporting Information [file ANIE-61-0-s002.pdf]

Angewandte Chemie
Eine Zeitschrift der Gesellschaft Deutscher Chemiker

Supporting Information

Functionalization and Hydrogenation of Carbon Chains Derived from CO

*M. Batuecas, R. Y. Kong, A. J. P. White, M. R. Crimmin**

Table of Contents

1 GENERAL EXPERIMENTAL S3

2 EXPERIMENTAL METHODS S4

2.1 – Preparation of Compounds S4

2.2 – Reversible conversion of **3a** to **4a** S31

2.3 – Direct reaction of **2**, H_2 and benzophenone S34

2.4 – Direct reaction of [W(CO$_6$)], **1** and syngas S35

2.5 – Kinetic experiments S36

2.6 – Monitoring of reaction of **3a** with H_2 S38

3 X-RAY DATA S39

4 DENSITY FUNCTIONAL THEORY CALCULATIONS S51

4.1 – Computational methods S51

4.2 –Calculated stationary points S52

4.2.1 – Transformation of **3a** to **4a** S52

4.2.2 – Calculated mechanism for hydrogenation of **4** S54

4.2.3 – Rotation barriers for **Int-1** S66

4.3 – Functional testing on key stationary points S67

5 NMR SPECTRA S68

6 COMPUTATIONAL COORDINATES S82

7 REFERENCES S240

1 GENERAL EXPERIMENTAL

All manipulations were carried out using standard Schlenk-line and glovebox techniques under an inert atmosphere of argon or dinitrogen. A MBraun Labmaster glovebox was employed, operating at <0.1 ppm O_2 and <0.1 ppm H_2O. A Polar Bear Cub reactor located inside this MBraun Labmaster glovebox was used as the low-temperature reactor. Solvents were dried over activated alumina from a SPS (solvent purification system) based upon the Grubbs design and degassed before use. Glassware was dried for 12 h at 120 °C prior to use. C_6D_6 was dried over 3 Å molecular sieves and freeze-pump-thaw degassed thrice before use.

NMR Spectra were recorded on Bruker 400 MHz at 25 °C unless otherwise stated and values recorded in ppm. Data were processed in MestReNova software. Where needed, chemical shifts were assigned with the assistance of 2D NMR (HSQC, HMBC, COSY) spectra. **1**[1] and **2**[2] and **3b**[2] were synthesized according to literature procedures. IR spectra were recorded on an Agilent Cary630 ATR FTIR spectrometer located inside an MBraun glovebox operating at <0.1 ppm O_2 and <0.1 ppm H_2O. Chemicals were purchased from Sigma Aldrich, Fluorochem, Alfa Aesar, or VWR and used as received. CO was purchased from BOC Ltd and used as received. Elemental analyses were performed by Elemental Labs (https://www.elementallab.co.uk/).

2 EXPERIMENTAL METHODS

2.1 – Preparation of Compounds

*Preparation of **3a***

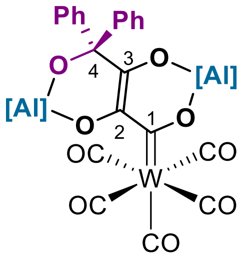

In a glovebox, **2** (15 mg, 0.012 mmol, 1 equiv) and benzophenone (2.6 mg, 0.014 mmol, 1.2 equiv) were dissolved in C_6D_6 (0.6 mL) and transferred to a J-Young NMR tube. The mixture was heated for 24 h at 100 °C. The reaction was monitored by ^{1}H NMR spectroscopy and was deemed to be complete upon consumption of **2**. The formation of both **3a** and **4a** was observed at this point in an approximate 4:1 ratio, respectively. The J-Young NMR tube was returned to the glovebox, and the headspace of the NMR tube was evacuated. CO gas (~1 atm) was introduced to the NMR tube and the reaction mixture was subsequently heated for a further 1 h at 100 °C. A ^{1}H NMR spectrum was taken at this time point and showed the full conversion of **4a** to **3a**. The J-Young NMR tube was returned to the glovebox, the reaction mixture was diluted with toluene (~0.5 mL), decanted into a 20 mL scintillation vial, and concentrated *in vacuo* until approx. 0.5 mL of solution remained. The solution was then filtered into a 4 mL vial, and further concentrated until approximately 0.2 mL of solution remained. n-Pentane (~2.5 mL) was layered on top, the vial was placed in the glovebox freezer (–35 °C), and **3a** was allowed to recrystallize as bright orange crystals. The supernatant was decanted, and the resultant crystals were washed with cold n-pentane (3 x 1mL) before the crystals were dried *in vacuo*. Yield: 10 mg, 0.0068 mmol, 56%.

^{1}H NMR (400 MHz, C_6D_6, 298 K) δ 0.60 (d, $^3J_{HH}$ = 6.8 Hz, 6H, (CH_3)$_2$CH), 0.74 (d, $^3J_{HH}$ = 6.8 Hz, 6H, (CH_3)$_2$CH), 0.93 (d, $^3J_{HH}$ = 6.8 Hz, 6H, (CH_3)$_2$CH), 1.02 (d, $^3J_{HH}$ = 6.8 Hz, 6H, (CH_3)$_2$CH), 1.05 (d, $^3J_{HH}$ = 6.8 Hz, 6H, (CH_3)$_2$CH), 1.23 (d, $^3J_{HH}$ = 6.7 Hz, 6H, (CH_3)$_2$CH), 1.27 (s, 6H, {(CH_3)$_2$C}$_2$CH), 1.37 (d, $^3J_{HH}$ = 6.8 Hz, 6H, (CH_3)$_2$CH), 1.44 (s, 6H, {(CH_3)$_2$C}$_2$CH), 1.57 (d, $^3J_{HH}$ = 6.7 Hz, 6H, (CH_3)$_2$CH), 2.54 (hept, $^3J_{HH}$ = 6.8 Hz, 2H, (CH$_3$)$_2$CH), 3.02 (hept, $^3J_{HH}$ = 6.9 Hz, 2H, (CH$_3$)$_2$CH), 3.23 (hept, $^3J_{HH}$ = 6.8 Hz, 2H, (CH$_3$)$_2$CH), 3.58 (hept, $^3J_{HH}$ = 6.6 Hz, 2H, (CH$_3$)$_2$CH), 4.86 (s, 1H, {(CH$_3$)C}$_2$CH), 4.99 (s, 1H, {(CH$_3$)C}$_2$CH), 6.40 – 6.47

(overlapping signals, 4H, Ar-***H***), 6.51 – 6.59 (overlapping signals, 4H, Ar-***H***), 6.80 (m, 2H, Ar-***H***), 6.90 (m, 2H, Ar-***H***), 6.96 (m, 2H, Ar-***H***), 7.08 – 7.22 (overlapping signals, 8H, Ar-***H***).

$^{13}C\{^1H\}$ NMR (101 MHz, C_6D_6, 298 K) δ 23.4 (2x (***C***$H_3)_2$CH), 24.0 (2x {(***C***$H_3)_2$C}$_2$CH), 24.1 (2x {(***C***$H_3)_2$C}$_2$CH), 24.2 (2x (***C***$H_3)_2$CH), 24.4 (2x (***C***$H_3)_2$CH), 24.6 (4x (***C***$H_3)_2$CH), 24.9 (2x (***C***$H_3)_2$CH), 27.3 (2x (***C***$H_3)_2$CH), 27.9 (2x (***C***$H_3)_2$CH), 28.4 (2x $(CH_3)_2$***C***H), 28.8 (4x $(CH_3)_2$***C***H), 29.1 (2x $(CH_3)_2$***C***H), 85.4 (***C***^{4}), 100.2 ({$(CH_3)_2C\}_2$***C***H), 100.6 ({$(CH_3)_2C\}_2$***C***H), 124.2 (Ar***C***), 124.7 (Ar***C***), 125.0 (Ar***C***), 125.6 (Ar***C***), 127.3 (Ar***C***), 127.4 (Ar***C***), 139.8 (Ar***C***), 140.6 (Ar***C***), 142.8 (Ar***C***), 143.5 (Ar***C***), 144.7 (Ar***C***), 146.0 (Ar***C***), 148.3 (Ar***C***), 152.9 (***C***$^{2/3}$), 155.9 (***C***$^{2/3}$), 172.7 ({$(CH_3)_2$***C***}$_2$CH), 174.5 ({$(CH_3)_2$***C***}$_2$CH), 203.2 (W(***C***O)$_4$), 205.1 (W***C***O), 315.6 (***C***^{1}). Some Ar***C*** resonances are overlapping and cannot be observed.

IR (ATR), ν_{CO} (cm^{-1}): 2050, 1897, 1871.

Anal. Calc. ($C_{79}H_{92}Al_2N_4O_9W$): C, 64.14; H, 6.44; N, 3.79. Found: C, 64.63; H, 6.47; N, 3.88.

Preparation of ***4a***

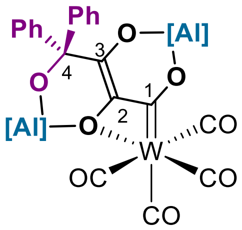

In a glovebox, **3a** (40 mg, 0.027 mmol) was dissolved in toluene (~5 mL) and transferred to a J-Young ampoule with an approximate headspace of 100 mL. The headspace of the ampoule was evacuated and the ampoule was removed from the glovebox. The reaction mixture was heated at 100 °C. After one hour, the ampoule was returned to the glovebox vacuum was applied to the headspace of the ampoule, and the ampoule was returned to heat at 100 °C. This process was repeated 5 times, for a total of 6 hours of heating, and a colour change from the characteristic yellow-orange of **3a** to deep red of **4a** was observed over the course of the reaction. After 6 hours, the reaction mixture was returned to the glovebox, decanted into a 20 mL scintillation vial, and the mixture was concentrated to a red oil. Pentane (ca. 2 mL) was added to the reaction mixture and the resultant solution was placed in the glovebox freezer to crystallise at –35 °C. **4a** crystallised as deep red blocks from this solution. The supernatant was decanted, and the crystals were washed thrice with pentane (3 x 1mL) before the crystals were dried *in vacuo*. Yield: 26 mg, 0.018 mmol, 66%.

^{1}H NMR (400 MHz, C_6D_6, 298 K) δ 0.41 (d, $^{3}J_{HH}$ = 6.6 Hz, 6H, (C***H***$_3$)$_2$CH), 0.68 (d, $^{3}J_{HH}$ = 6.8 Hz, 6H, (C***H***$_3$)$_2$CH), 0.80 (d, $^{3}J_{HH}$ = 6.8 Hz, 6H, (C***H***$_3$)$_2$CH), 0.95 (d, $^{3}J_{HH}$ = 6.7 Hz, 6H, (C***H***$_3$)$_2$CH), 1.07 (d, $^{3}J_{HH}$ = 6.7 Hz, 6H, (C***H***$_3$)$_2$CH), 1.15 (d, $^{3}J_{HH}$ = 6.7 Hz, 6H, (C***H***$_3$)$_2$CH), 1.32 (s, 6H, {(C***H***$_3$)$_2$C}$_2$CH), 1.49 (d, $^{3}J_{HH}$ = 6.6 Hz, 6H, (C***H***$_3$)$_2$CH), 1.66 (s, 6H, {(C***H***$_3$)$_2$C}$_2$CH), 1.78 (d, $^{3}J_{HH}$ = 6.7 Hz, 6H, (C***H***$_3$)$_2$CH), 2.62 (hept, $^{3}J_{HH}$ = 6.8 Hz, 2H, (CH$_3$)$_2$C***H***), 3.11 (hept, $^{3}J_{HH}$ = 6.7 Hz, 2H, (CH$_3$)$_2$C***H***), 3.24 (hept, $^{3}J_{HH}$ = 6.7 Hz, 2H, (CH$_3$)$_2$C***H***), 3.40 (hept, $^{3}J_{HH}$ = 6.6 Hz, 2H, (CH$_3$)$_2$C***H***), 5.11 (s, 1H, {(CH$_3$)C}$_2$C***H***), 5.33 (s, 1H, {(CH$_3$)C}$_2$C***H***), 6.43 (m, 4H, Ar-***H***), 6.60 (m, 4H, Ar-***H***), 6.83 (m, 2H, Ar-***H***), 6.99 (m, 2H, Ar-***H***), 7.08 (m, 4H, Ar-***H***), 7.16 – 7.26 (overlapping signals, 6H, Ar-***H***).

$^{13}C\{^{1}H\}$ NMR (101 MHz, C_6D_6, 298 K) δ 24.0 (2x ($\boldsymbol{C}H_3)_2CH$), 24.2 (2x ($\boldsymbol{C}H_3)_2CH$), 24.2 (2x ($\boldsymbol{C}H_3)_2CH$), 24.3 (2x $\{(\boldsymbol{C}H_3)_2C\}_2CH$), 24.6 (2x ($\boldsymbol{C}H_3)_2CH$), 24.6 (2x ($\boldsymbol{C}H_3)_2CH$), 24.9 (2x $\{(\boldsymbol{C}H_3)_2C\}_2CH$), 24.9 (2x ($\boldsymbol{C}H_3)_2CH$), 25.1 (2x ($\boldsymbol{C}H_3)_2CH$), 27.8 (2x ($\boldsymbol{C}H_3)_2CH$), 28.2 (2x $(CH_3)_2\boldsymbol{C}H$), 28.7 (2x $(CH_3)_2\boldsymbol{C}H$), 29.0 (2x $(CH_3)_2\boldsymbol{C}H$), 29.3 (2x $(CH_3)_2\boldsymbol{C}H$), 82.3 ($\boldsymbol{C}^{4}$), 100.7 ($\{(CH_3)_2C\}_2\boldsymbol{C}H$), 101.0 ($\{(CH_3)_2C\}_2\boldsymbol{C}H$), 124.0 (Ar***C***), 124.5 (Ar***C***), 125.4 (Ar***C***), 126.0(Ar***C***), 126.1 (Ar***C***), 127.4 (Ar***C***), 127.6 (Ar***C***), 128.8 (Ar***C***), 139.9 (Ar***C***), 140.0 (Ar***C***), 142.8 ($\boldsymbol{C}^{2/3}$), 143.2 (Ar***C***), 143.4 (Ar***C***), 146.1 (Ar***C***), 146.9 (Ar***C***), 147.5 (Ar***C***), 161.8 ($\boldsymbol{C}^{2/3}$), 174.6 ($\{(CH_3)_2\boldsymbol{C}\}_2CH$), 174.6 ($\{(CH_3)_2\boldsymbol{C}\}_2CH$), 215.5 (2 x $W(\boldsymbol{C}O)_4$), 218.8 ($W(\boldsymbol{C}O)_4$), 221.4 ($W(\boldsymbol{C}O)_4$), 310.6 ($\boldsymbol{C}^{1}$). Some Ar***C*** resonances are overlapping and cannot be observed.

IR (ATR), ν_{CO} (cm^{-1}): 1988, 1874, 1862, 1825.

Anal. Calc. ($C_{78}H_{92}Al_2N_4O_8W$): C, 64.55; H, 6.39; N, 3.86. Found: C, 64.61; H, 6.33; N, 3.61.

Preparation of ***5a***

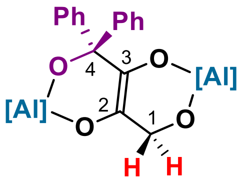

In a glovebox, **4a** (10 mg, 0.007 mmol) was dissolved in C_6D_6 (0.6 mL) and transferred to a J-Young NMR tube. The headspace of the NMR tube was evacuated, H_2 gas (~1 bar) was introduced into the NMR tube and the reaction mixture was heated for 2 h at 100 °C. After this time, conversion >95 % of **4a** to **5a** was observed. All attempts to separate **5a** from reaction mixture were unsuccessful. Compound **5a** was characterized by NMR spectroscopy.
5b can be also obtained in similar yield by reaction of **3a** (8 mg, 0.0055 mmol) with H_2, following the former procedure (see section 2.6 below).

^{1}H NMR (400 MHz, C_6D_6, 298 K) δ 0.37 (d, $^3J_{HH}$ = 6.8 Hz, 6H, (C***H***$_3$)$_2$CH), 0.96 (d, $^3J_{HH}$ = 6.8 Hz, 12H, (C***H***$_3$)$_2$CH), 1.12 (d, $^3J_{HH}$ = 6.8 Hz, 6H, (C***H***$_3$)$_2$CH), 1.13 (d, $^3J_{HH}$ = 6.8 Hz, 6H, (C***H***$_3$)$_2$CH), 1.18 (d, $^3J_{HH}$ = 6.7 Hz, 12H, (C***H***$_3$)$_2$CH), 1.42 (s, 6H, {(C***H***$_3$)$_2$C}$_2$CH), 1.44 (s, 6H, {(C***H***$_3$)$_2$C}$_2$CH), 1.69 (d, $^3J_{HH}$ = 6.7 Hz, 6H, (C***H***$_3$)$_2$CH), 2.84 (hept, $^3J_{HH}$ = 6.8 Hz, 2H, (CH$_3$)$_2$C***H***), 2.96 (hept, $^3J_{HH}$ = 6.8 Hz, 2H, (CH$_3$)$_2$C***H***), 3.34 (hept, $^3J_{HH}$ = 6.8 Hz, 2H, (CH$_3$)$_2$C***H***), 3.49 (hept, $^3J_{HH}$ = 6.7 Hz, 2H, (CH$_3$)$_2$C***H***), 4.38 (s, 2H, C***H***$_2$), 4.84 (s, 1H, {(CH$_3$)C}$_2$C***H***), 4.86 (s, 1H, {(CH$_3$)C}$_2$C***H***), 6.72 – 6.84 (overlapping signals, 8H, Ar-***H***), 6.94 (m, 2H, Ar-***H***), 6.98 (m, 2H, Ar-***H***), 7.03 (m, 2H, Ar-***H***), 7.11 – 7.21 (overlapping signals, 4H, Ar-***H***), 7.26 (m, 4H, Ar-***H***).

^{13}C{^{1}H} NMR (101 MHz, C_6D_6, 298 K) δ 23.2 (2x (***C***H$_3$)$_2$CH), 23.5 (2x (***C***H$_3$)$_2$CH), 24.0 (2x {(***C***H$_3$)$_2$C}$_2$CH), 24.1 (2x {(***C***H$_3$)$_2$C}$_2$CH), 24.4 (2x (***C***H$_3$)$_2$CH), 24.7 (2x (***C***H$_3$)$_2$CH), 24.9 (2x (***C***H$_3$)$_2$CH), 25.0 (2x (***C***H$_3$)$_2$CH), 25.5 (2x (***C***H$_3$)$_2$CH), 26.0 (2x (***C***H$_3$)$_2$CH), 28.0 (2x (CH$_3$)$_2$***C***H), 28.7 (2x (CH$_3$)$_2$***C***H), 28.8 (2x (CH$_3$)$_2$***C***H), 28.9 (2x (CH$_3$)$_2$***C***H), 69.2 (C***H***$_2$), 84.5 (***C***^{4}), 97.6 ({(CH$_3$)$_2$C}$_2$***C***H), 97.8 ({(CH$_3$)$_2$C}$_2$***C***H), 124.2 (Ar***C***), 124.4 (Ar***C***), 124.6 (Ar***C***), 124.8 (Ar***C***), 125.2 (Ar***C***), 126.9 (Ar***C***), 127.3 (Ar***C***), 127.5 (Ar***C***), 129.4 (Ar***C***), 135.9 (***C***$^{2/3}$), 138.8 (***C***$^{2/3}$), 140.1 (Ar***C***), 140.5 (Ar***C***), 143.0 (Ar***C***), 143.9 (Ar***C***), 144.8 (Ar***C***), 145.0 (Ar***C***),

150.1 (Ar***C***), 150.4 (Ar***C***), 171.1 ({$(CH_3)_2$***C***}$_2$CH), 172.2 ({$(CH_3)_2$***C***}$_2$CH). Some Ar***C*** resonances are overlapping and cannot be observed.

Also observed resonances corresponding to $W(CO)_6$: δ 191.2 ($^1J_{WC}$ = 126.7 Hz),[3] and $(C_6D_6)W(CO)_3$: δ 209.3.[4]

Attempts to obtain mass spectra data of **5a** by ES and APCI (both +ve and -ve mode) were unsuccessful. Submission of CH_3CN/H_2O (20:80) solutions of **5a** to LC-MS allowed detection of hydrolyzed species: m/z calcd for $C_{16}H_{17}O_4$ $[M+H]^+$ 273.1, found 273.4; calcd for $C_{16}H_{14}O_3$ $[M-H_2O]^+$ 254.1, found 254.3.

Preparation of ***5a-d₂***

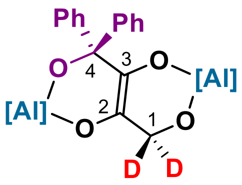

In a glovebox, **4a** (10 mg, 0.007 mmol) was dissolved in C_6D_6 (0.6 mL) and transferred to a J-Young NMR tube. The headspace of the NMR tube was evacuated, D_2 gas (~1 bar) was introduced into the NMR tube and the reaction mixture was heated for 2 h at 100 °C. After this time, an ^{1}H NMR spectrum was recorded to check that reaction was completed and expected product was formed. Data for ***5a-d$_2$*** are consistent with those reported for **5a** but the resonance at δ = 4.38 ppm corresponding to CH_2 fragment was not observed. The J-Young NMR tube was returned to the glovebox, the solvent was removed under reduced pressure and the residue dissolved in C_6H_6. A ^{2}H NMR spectra was recorded.
^{2}H NMR (61 MHz, C_6H_6, 298 K) δ 4.21 (bs).

Attempts to obtain mass spectra data of ***5a-d$_2$*** by ES and APCI (both +ve and -ve mode) were unsuccessful. Submission of CH_3CN/H_2O (20:80) solutions of ***5a-d$_2$*** to LC-MS allowed detection of hydrolyzed species: m/z calcd for $C_{16}H_{13}D_2O_4$ $[M-H]^-$ 273.1, found 273.2; calcd for $C_{16}H_{11}D_2O_3$ $[M-H_2O-H]^-$ 255.1, found 255.1.

Preparation of ***5b***

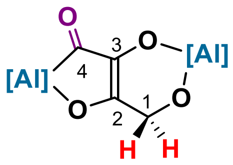

In a glovebox, **3b** (10 mg, 0.007 mmol) was dissolved in C_6D_6 (0.6 mL) and transferred to a J-Young NMR tube. The headspace of the NMR tube was evacuated, H_2 gas (~1 bar) was introduced into the NMR tube and the reaction mixture was heated for 12 h at 100 °C. After this time, conversion >95 % of **3b** to **5b** was observed. All attempts to separate **5b** from reaction mixture were unsuccessful. Compound **5b** was characterized by NMR spectroscopy.

^{1}H NMR (400 MHz, C_6D_6, 298 K) δ 0.56 (d, $^3J_{HH}$ = 6.8 Hz, 6H, (C***H***$_3$)$_2$CH), 0.87 (d, $^3J_{HH}$ = 6.8 Hz, 6H, (C***H***$_3$)$_2$CH), 0.89 (d, $^3J_{HH}$ = 6.8 Hz, 6H, (C***H***$_3$)$_2$CH), 1.01 (d, $^3J_{HH}$ = 6.8 Hz, 6H, (C***H***$_3$)$_2$CH)), 1.10 (d, $^3J_{HH}$ = 6.7 Hz, 6H, (C***H***$_3$)$_2$CH)), 1.14 (d, $^3J_{HH}$ = 6.7 Hz, 6H, (C***H***$_3$)$_2$CH)), 1.44 (s, 6H, {(C***H***$_3$)$_2$C}$_2$CH), 1.52 (d, $^3J_{HH}$ = 6.8 Hz, 6H, (C***H***$_3$)$_2$CH)), 1.54 (s, 6H, {(C***H***$_3$)$_2$C}$_2$CH), 1.71 (d, $^3J_{HH}$ = 6.7 Hz, 6H, (C***H***$_3$)$_2$CH)), 3.13 (hept, $^3J_{HH}$ = 6.8 Hz, 2H, (CH$_3$)$_2$C***H***), 3.21 (hept, $^3J_{HH}$ = 6.8 Hz, 2H, (CH$_3$)$_2$C***H***), 3.27 (hept, $^3J_{HH}$ = 6.8 Hz, 2H, (CH$_3$)$_2$C***H***), 3.32 (hept, $^3J_{HH}$ = 6.8 Hz, 2H, (CH$_3$)$_2$C***H***), 4.28 (s, 2H, C***H***$_2$), 4.85 (s, 1H, {(CH$_3$)C}$_2$C***H***), 4.94 (s, 1H, {(CH$_3$)C}$_2$C***H***), 6.97– 7.28 (overlapping signals, 12H, Ar-***H***).

^{13}C NMR (101 MHz, C_6D_6, 298 K) δ 22.9 (2x {(***C***H$_3$)$_2$C}$_2$CH), 23.0 (2x {(***C***H$_3$)$_2$C}$_2$CH), 24.3 (2x (***C***H$_3$)$_2$CH), 24.6 (2x (***C***H$_3$)$_2$CH), 24.7 (2x (***C***H$_3$)$_2$CH), 24.8 (2x (***C***H$_3$)$_2$CH), 24.9 (2x (***C***H$_3$)$_2$CH), 24.9 (2x (***C***H$_3$)$_2$CH), 25.1 (2x (***C***H$_3$)$_2$CH), 27.8 (2x (CH$_3$)$_2$***C***H), 28.3 (2x (CH$_3$)$_2$***C***H), 28.9 (4x (CH$_3$)$_2$***C***H), 29.6 (2x (***C***H$_3$)$_2$CH), 67.5 (C***H***$_2$), 96.8 ({(CH$_3$)$_2$C}$_2$***C***H), 98.2 ({(CH$_3$)$_2$C}$_2$***C***H), 123.8 (Ar***C***), 124.4 (Ar***C***), 124.6 (Ar***C***), 124.9 (Ar***C***), 125.4 (Ar***C***), 127.3 (Ar***C***), 137.8 (Ar***C***), 138.5 (Ar***C***), 138.7 (Ar***C***), 143.8 (Ar***C***), 144.4 (Ar***C***), 145.0 (Ar***C***), 146.0 (Ar***C***), 151.8 (***C***^{3}), 168.4 (***C***^{2}), 171.2 ({(CH$_3$)$_2$***C***}$_2$CH), 171.9 ({(CH$_3$)$_2$***C***}$_2$CH). Some Ar***C*** resonances are overlapping and cannot be observed. The Al–***C***^{4} resonance could not be observed in the ^{13}C NMR spectrum due to coupling to the quadrupolar ^{27}Al (I = 5/2) nucleus.

Preparation of ***3c***

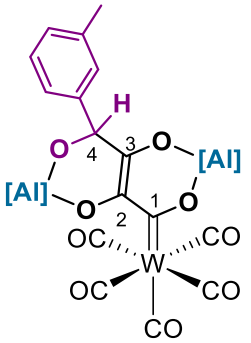

3c was prepared following the reaction sequence shown in Scheme S1. Reaction of **2** at 25 °C gives the kinetic product **S1** by C=O insertion into Al–O bond.[2] **S1** evolves to the thermodynamic product **3c** after heating at 60 °C for 48 h.

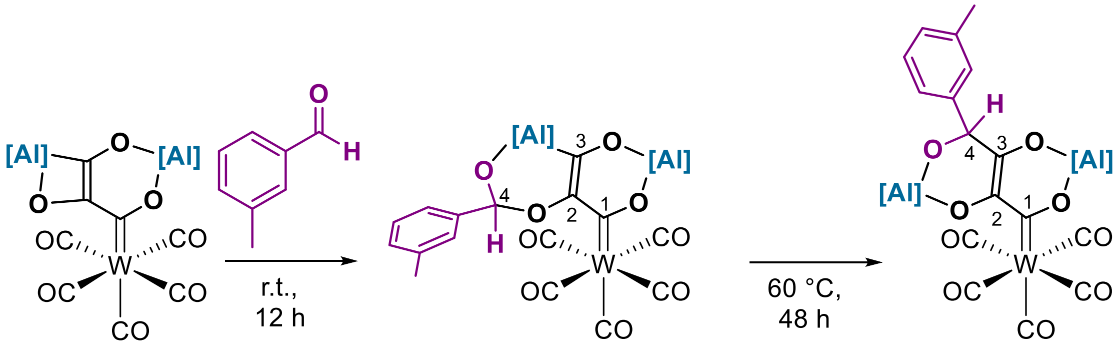

2 **S1** **3c**

Scheme S1: Reaction of **2** with 3-tolualdehyde.

Preparation of **S1**: **2** was prepared *in-situ* in an NMR tube from **1** (17.8 mg, 0.04 mmol), $[W(CO)_6]$ (8 mg, 0.023 mmol) and CO gas (~1 bar) following the reported procedure.[2] Once formation of **2** was confirmed by ^1H NMR spectroscopy, the NMR tube was returned to the glovebox, the reaction mixture was cooled to –35 °C using a low temperature reactor and the headspace of the NMR tube was evacuated to remove the remaining CO gas and 3-tolualdehyde (4.8 μL, 0.04 mmol) was added via microsyringe. After 12 h at 25 °C the reaction mixture was returned to the glovebox, diluted with ~0.5 mL of toluene and decanted into a 20 mL scintillation vial. The resultant solution was concentrated *in vacuo* and residue dissolved in THF (~0.2 mL), filtered into a 4 mL vial, and carefully layered with n-pentane (~2 mL). The vial was placed in the glovebox freezer (–35 °C) and **S1** crystallised as orange blocks. The supernatant was decanted, and the resultant crystals were washed with cold n-pentane thrice (3 x 0.5mL) before being dried *in vacuo.* Yield: 15.3 mg, 0.011 mmol, 53%.

^{1}H NMR (400 MHz, THF-d_8, 298 K) δ 0.49 ((d, $^{3}J_{HH}$ = 6.7 Hz, 3H, (C***H*₃**)₂CH)), 0.51 (d, $^{3}J_{HH}$ = 6.7 Hz, 3H, ($C\boldsymbol{H_3}$)$_2$CH)), 0.61 (d, $^{3}J_{HH}$ = 6.7 Hz, 3H, ($C\boldsymbol{H_3}$)$_2$CH)), 0.85 (d, $^{3}J_{HH}$ = 6.7 Hz, 3H, ($C\boldsymbol{H_3}$)$_2$CH)), 0.86 (d, $^{3}J_{HH}$ = 6.7 Hz, 3H, ($C\boldsymbol{H_3}$)$_2$CH)), 0.94 (d, $^{3}J_{HH}$ = 6.9 Hz, 3H, ($C\boldsymbol{H_3}$)$_2$CH)), 0.95 (d, $^{3}J_{HH}$ = 6.8 Hz, 3H, ($C\boldsymbol{H_3}$)$_2$CH)), 1.07 (d, $^{3}J_{HH}$ = 6.9 Hz, 3H, ($C\boldsymbol{H_3}$)$_2$CH)), 1.10 (d, $^{3}J_{HH}$ = 6.6 Hz, 3H, ($C\boldsymbol{H_3}$)$_2$CH)), 1.18 (d, $^{3}J_{HH}$ = 6.8 Hz, 3H, ($C\boldsymbol{H_3}$)$_2$CH)), 1.25 (d, $^{3}J_{HH}$ = 6.7 Hz, 3H, ($C\boldsymbol{H_3}$)$_2$CH)), 1.33 (d, $^{3}J_{HH}$ = 6.9 Hz, 3H, ($C\boldsymbol{H_3}$)$_2$CH)), 1.38 (d, $^{3}J_{HH}$ = 6.7 Hz, 3H, ($C\boldsymbol{H_3}$)$_2$CH)), 1.43 (d, $^{3}J_{HH}$ = 6.8 Hz, 3H, ($C\boldsymbol{H_3}$)$_2$CH)), 1.47 (d, $^{3}J_{HH}$ = 6.7 Hz, 3H, ($C\boldsymbol{H_3}$)$_2$CH)), 1.53 (d, $^{3}J_{HH}$ = 6.7 Hz, 3H, ($C\boldsymbol{H_3}$)$_2$CH)), 1.65 (s, 3H, {($C\boldsymbol{H_3}$)$_2$C}$_2$CH), 1.82 (s, 3H, {($C\boldsymbol{H_3}$)$_2$C}$_2$CH), 1.84 (s, 3H, {($C\boldsymbol{H_3}$)$_2$C}$_2$CH), 1.96 (s, 3H, {($C\boldsymbol{H_3}$)$_2$C}$_2$CH), 2.09 (s, 3H, $C\boldsymbol{H}_3$-C_6H_4), 2.60 – 2.80 (hept overlapping, 3H, $(CH_3)_2C\boldsymbol{H}$), 2.87 (hept, $^{3}J_{HH}$ = 6.8 Hz, 1H, $(CH_3)_2C\boldsymbol{H}$), 3.13 (hept overlapping, 2H, $(CH_3)_2C\boldsymbol{H}$), 3.24 (hept overlapping, 2H, $(CH_3)_2C\boldsymbol{H}$), 5.40 (s, 1H, {$(CH_3)C$}$_2C\boldsymbol{H}$), 5.43 (s, 1H, $\boldsymbol{H}C^4$), 5.75 (s, 1H, {$(CH_3)C$}$_2C\boldsymbol{H}$), 6.20 (s, 1H, Ar-***H***), 6.23 (m, 1H, Ar-***H***), 6.72 – 6.77 (m, 2H, Ar-***H***), 6.93 – 7.01 (overlapping signals, 2H, Ar-***H***), 7.03 – 7.12 (overlapping signals, 4H, Ar-***H***), 7.21 (m, 1H, Ar-***H***), 7.25 – 7.41 (overlapping signals, 5H, Ar-***H***).

^{13}C NMR (101 MHz, THF-d_8, 298 K) δ 21.7 ($\boldsymbol{C}H_3$-C_6H_4), 23.1 (($\boldsymbol{C}H_3$)$_2$CH), 23.6 (($\boldsymbol{C}H_3$)$_2$CH), 23.6 (($\boldsymbol{C}H_3$)$_2$CH), 24.1 ({($\boldsymbol{C}H_3$)$_2$C}$_2$CH), 24.6 – 26.1 (overlapping signals, 3 x {($\boldsymbol{C}H_3$)$_2$C}$_2$CH and 8 x ($\boldsymbol{C}H_3$)$_2$CH), 26.2 (($\boldsymbol{C}H_3$)$_2$CH), 26.3 (($\boldsymbol{C}H_3$)$_2$CH), 26.6 (($\boldsymbol{C}H_3$)$_2$CH), 26.6 (($\boldsymbol{C}H_3$)$_2$CH), 27.2 (($\boldsymbol{C}H_3$)$_2$CH), 28.8 ($(CH_3)_2\boldsymbol{C}H$), 29.1 ($(CH_3)_2\boldsymbol{C}H$), 29.2 ($(CH_3)_2\boldsymbol{C}H$), 29.2 ($(CH_3)_2\boldsymbol{C}H$), 29.4 ($(CH_3)_2\boldsymbol{C}H$), 29.4 ($(CH_3)_2\boldsymbol{C}H$), 29.4 ($(CH_3)_2\boldsymbol{C}H$), 29.5 ($(CH_3)_2\boldsymbol{C}H$), 100.1 ({$(CH_3)_2C$}$_2\boldsymbol{C}H$), 101.6 ({$(CH_3)_2C$}$_2\boldsymbol{C}H$), 103.5 ($\boldsymbol{C^4}$), 123.9 (Ar***C***), 124.7 (Ar***C***), 125.3 (Ar***C***), 125.4 (Ar***C***), 125.5 (Ar***C***), 125.7 (Ar***C***), 125.8 (Ar***C***), 126.2 (Ar***C***), 126.7 (Ar***C***), 127.1 (Ar***C***), 128.1 (Ar***C***), 128.2 (Ar***C***), 128.6 (Ar***C***), 128.7 (Ar***C***), 129.3 (Ar***C***), 136.4 (Ar***C***), 139.1 (Ar***C***), 139.9 (Ar***C***), 140.8 (Ar***C***), 141.1 (Ar***C***), 141.8 (Ar***C***), 143.3 (Ar***C***), 144.2 (Ar***C***), 144.5 (Ar***C***), 145.2 (Ar***C***), 145.4 (Ar***C***), 145.6 (Ar***C***), 145.7 (Ar***C***), 146.6 (Ar***C***), 162.1 (Ar***C***), 169.7 ($\boldsymbol{C^2}$), 173.0 ({$(CH_3)_2\boldsymbol{C}$}$_2$CH), 173.1 ({$(CH_3)_2\boldsymbol{C}$}$_2$CH), 174.8 ({$(CH_3)_2\boldsymbol{C}$}$_2$CH), 174.8 ({$(CH_3)_2\boldsymbol{C}$}$_2$CH), 200.5 (W($\boldsymbol{C}$O)$_4$), 206.6 (W($\boldsymbol{C}$O)), 300.4 ($\boldsymbol{C^1}$). The Al–$\boldsymbol{C^3}$ resonance could not be observed in the ^{13}C NMR spectrum due to coupling to the quadrupolar ^{27}Al (I = 5/2) nucleus.

IR (ATR), ν_{CO} (cm^{-1}): 2050, 1905, 1871.

Preparation of **3c**: In a glovebox, **S1** (8 mg, 0.0056 mmol) was dissolved in C_6D_6 (0.6 mL) and transferred to a J-Young NMR tube. The solution was heated for 48 h at 60 °C. The reaction was monitored by ^{1}H NMR spectroscopy and was deemed to be complete upon consumption of **S1**. Then the NMR tube was returned to the glovebox, diluted with ~0.5 mL of toluene and decanted into a 20 mL scintillation vial. The resultant solution was concentrated *in vacuo* and residue was extracted with pentane (3 x 0.5mL). The resultant yellow solution was concentrated (~0.7 mL) and placed in the glovebox freezer (–35 °C) for 2 days to allow crystallization of **3c** as brown blocks. The mother liquor was decantated and the crystals washed with cold n-pentane thrice (3 x 0.5mL) before being dried *in vacuo*. Yield: 4.4 mg, 0.0031 mmol, 55%.

^{1}H NMR (400 MHz, C_6D_6, 298 K) δ 0.43 (d, $^3J_{HH}$ = 6.8 Hz, 3H, $(CH_3)_2CH$), 0.43 (d, $^3J_{HH}$ = 6.7 Hz, 3H, $(CH_3)_2CH$), 0.68 (d, $^3J_{HH}$ = 6.7 Hz, 3H, $(CH_3)_2CH$), 0.76 (d, $^3J_{HH}$ = 6.7 Hz, 3H, $(CH_3)_2CH$), 0.96 (d, $^3J_{HH}$ = 6.9 Hz, 3H, $(CH_3)_2CH$), 0.97 (d, $^3J_{HH}$ = 6.7 Hz, 3H, $(CH_3)_2CH$), 1.07 (d, $^3J_{HH}$ = 6.8 Hz, 3H, $(CH_3)_2CH$), 1.08 (d, $^3J_{HH}$ = 6.9 Hz, 3H, $(CH_3)_2CH$), 1.09 (d, $^3J_{HH}$ = 6.7 Hz, 3H, $(CH_3)_2CH$), 1.13 (d, $^3J_{HH}$ = 6.7 Hz, 3H, $(CH_3)_2CH$), 1.31 (s, 3H, $\{(CH_3)_2C\}_2CH$), 1.32 (d, $^3J_{HH}$ = 6.8 Hz, 3H, $(CH_3)_2CH$), 1.33 (d, $^3J_{HH}$ = 6.7 Hz, 3H, $(CH_3)_2CH$), 1.33 (d, $^3J_{HH}$ = 6.8 Hz, 3H, $(CH_3)_2CH$), 1.44 (s, 3H, $\{(CH_3)_2C\}_2CH$), 1.40 (s, 3H, $\{(CH_3)_2C\}_2CH$), 1.38 (s, 3H, $\{(CH_3)_2C\}_2CH$), 1.55 (d, $^3J_{HH}$ = 6.8 Hz, 3H, $(CH_3)_2CH$), 1.56 (d, $^3J_{HH}$ = 6.7 Hz, 3H, $(CH_3)_2CH$), 1.65 (d, $^3J_{HH}$ = 6.8 Hz, 3H, $(CH_3)_2CH$), 2.05 (s, 3H, $CH_3C_6H_4$), 2.40 (hept, $^3J_{HH}$ = 6.7 Hz, 1H, $(CH_3)_2CH$), 2.99 - 3.11 (hept overlapping, 2H, $(CH_3)_2CH$), 3.11 - 3.25 (hept overlapping, 3H, $(CH_3)_2CH$), 3.49 (hept, $^3J_{HH}$ = 6.7 Hz, 1H, $(CH_3)_2CH$), 3.74 (hept, $^3J_{HH}$ = 6.7 Hz, 1H, $(CH_3)_2CH$), 4.81 (s, 1H, $\{(CH_3)C\}_2CH$), 4.95 (s, 1H, $\{(CH_3)C\}_2CH$), 5.14 (s, 1H, C^4H), 6.46 (d, $^3J_{HH}$ = 7.5, 1H, Ar-*H*), 6.71 (t, $^3J_{HH}$ = 7.5 Hz, 1H, Ar-*H*), 6.78 - 6.86 (overlapping signals, 3H, Ar-*H*), 6.96 - 7.00 (m, 1H, Ar-*H*), 7.03 - 7.12 (overlapping signals, 6H, Ar-*H*), 7.17 - 7.30 (overlapping signals, 4H, Ar-*H*).

$^{13}C\{^1H\}$ NMR (101 MHz, C_6D_6, 298 K) δ 21.3 ($CH_3C_6H_4$), 23.5 ($\{(CH_3)_2C\}_2CH$), 23.8 ($\{(CH_3)_2C\}_2CH$), 23.9 ($(CH_3)_2CH$), 23.9 ($(CH_3)_2CH$), 24.3 (2x $(CH_3)_2CH$), 24.5 (2x $(CH_3)_2CH$), 24.6 (2x $(CH_3)_2CH$), 24.7 (2x $(CH_3)_2CH$), 25.4 (2x $(CH_3)_2CH$), 25.9 (2x $(CH_3)_2CH$), 26.0 (2x $(CH_3)_2CH$), 27.7 ($(CH_3)_2CH$), 27.8 ($(CH_3)_2CH$), 28.0 ($(CH_3)_2CH$), 28.1 ($(CH_3)_2CH$), 28.7 ($(CH_3)_2CH$), 28.8 ($(CH_3)_2CH$), 28.8 ($(CH_3)_2CH$), 28.9 ($(CH_3)_2CH$), 80.3 (C^4H), 99.7 ($\{(CH_3)_2C\}_2CH$), 99.8 ($\{(CH_3)_2C\}_2CH$), 124.2 (Ar*C*), 124.7 (Ar*C*), 124.8 (Ar*C*),

124.9 (Ar***C***), 125.1 (Ar***C***), 125.4 (Ar***C***), 125.5 (Ar***C***), 136.2 (Ar***C***), 138.1 (Ar***C***), 138.6 (Ar***C***), 140.4 (Ar***C***), 140.6 (Ar***C***), 142.8 (Ar***C***), 143.4 (Ar***C***), 143.8 (Ar***C***), 143.9 (Ar***C***), 144.3 (Ar***C***), 144.4 (Ar***C***), 146.0 (Ar***C***), 146.2 (Ar***C***), 151.2 ($\boldsymbol{C}^3$), 154.6 ($\boldsymbol{C}^2$), 173.8 ({$(CH_3)_2$***C***}$_2$CH), 173.3 ({$(CH_3)_2$***C***}$_2$CH), 172.6 ({$(CH_3)_2$***C***}$_2$CH), 172,5 ({$(CH_3)_2$***C***}$_2$CH), 202.1 (W(***C***O)$_4$), 211.7 (W(***C***O)), 314.1 ($\boldsymbol{C}^1$). Some Ar***C*** resonances are overlapping and cannot be observed.

IR (ATR), ν_{CO} (cm^{-1}): 2052, 1927, 1901, 1867.

Anal. Calc. ($C_{74}H_{90}Al_2N_4O_9W$): C, 62.71; H, 6.40; N, 3.95. Found: C, 60.64; H, 6.29; N, 4.28. The low C content, but accurate H and N content likely reflect limitations of the technique (e.g. incomplete C combustion).

Preparation of ***5c***

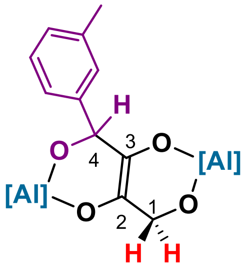

In a glovebox, **3c** (5 mg, 0.0036 mmol) was dissolved in C_6D_6 (0.6 mL) and transferred to a J-Young NMR tube containing a capillary with 1,3,5-trimethoxybenzene as external standard. The headspace of the NMR tube was evacuated, H_2 gas (~1 bar) was introduced into the NMR tube and the reaction mixture was heated for 48 h at 100 °C. After this time, **3c** was not observed by ^{1}H NMR spectroscopy in the reaction mixture. NMR yield: 60 %. Compound **5c** was characterized by NMR spectroscopy.

^{1}H NMR (400 MHz, C_6D_6, 298 K) δ 0.57 (d, $^3J_{HH}$ = 6.7 Hz, 3H, (C***H***$_3$)$_2$CH), 0.87 (d, $^3J_{HH}$ = 6.8 Hz, 3H, (C***H***$_3$)$_2$CH), 0.93 (d, $^3J_{HH}$ = 6.7 Hz, 3H, (C***H***$_3$)$_2$CH), 0.97 (d, $^3J_{HH}$ = 6.8 Hz, 3H, (C***H***$_3$)$_2$CH), 0.99 (d, $^3J_{HH}$ = 6.6 Hz, 3H, (C***H***$_3$)$_2$CH), 1.05 (d, $^3J_{HH}$ = 6.8 Hz, 3H, (C***H***$_3$)$_2$CH), 1.07 (d, $^3J_{HH}$ = 6.7 Hz, 3H, (C***H***$_3$)$_2$CH), 1.09 (d, $^3J_{HH}$ = 6.9 Hz, 3H, (C***H***$_3$)$_2$CH), 1.10 (d, $^3J_{HH}$ = 6.7 Hz, 3H, (C***H***$_3$)$_2$CH), 1.13 (d, $^3J_{HH}$ = 6.8 Hz, 3H, (C***H***$_3$)$_2$CH), 1.16 (d, $^3J_{HH}$ = 6.8 Hz, 6H, (C***H***$_3$)$_2$CH), 1.22 (d, $^3J_{HH}$ = 6.9 Hz, 3H, (C***H***$_3$)$_2$CH), 1.34 (d, $^3J_{HH}$ = 6.7 Hz, 3H, (C***H***$_3$)$_2$CH), 1.42 (s, 3H, {(C***H***$_3$)$_2$C}$_2$CH), 1.44 (s, 3H, {(C***H***$_3$)$_2$C}$_2$CH), 1.45 (s, 3H, {(C***H***$_3$)$_2$C}$_2$CH), 1.48 (s, 3H, {(C***H***$_3$)$_2$C}$_2$CH), 1.50 (d, $^3J_{HH}$ = 6.7 Hz, 3H, (C***H***$_3$)$_2$CH), 1.51 (d, $^3J_{HH}$ = 6.7 Hz, 3H, (C***H***$_3$)$_2$CH), 2.14 (s, 3H, C***H***$_3$C$_6$H$_4$), 2.78 (hept, $^3J_{HH}$ = 6.8 Hz, 1H, (CH$_3$)$_2$C***H***), 3.01 (hept, $^3J_{HH}$ = 6.9 Hz, 1H, (CH$_3$)$_2$C***H***), 3.21 – 3.37 (hept overlapping, 4H, (CH$_3$)$_2$C***H***), 3.42 (hept, $^3J_{HH}$ = 6.9 Hz, 1H, (CH$_3$)$_2$C***H***), 3.53 (hept, $^3J_{HH}$ = 6.8 Hz, 1H, (CH$_3$)$_2$C***H***), 4.42 (AB spin system, Δν = 83.2 Hz, J_{AB} = 13.8 Hz, 2H, C***H***$_2$), 4.83 (s, 1H, {(CH$_3$)C}$_2$C***H***), 4.88 (s, 1H, {(CH$_3$)C}$_2$C***H***), 5.01 (s, 1H, C^4***H***), 6.46 (m, 1H, Ar-***H***), 6.70 (s, 1H, Ar-***H***), 6.78 (m, 1H, Ar-***H***), 6.83 – 6.89 (overlapping signals, 2H, Ar-***H***), 6.94 (m, 1H, Ar-***H***), 7.04 – 7.09 (overlapping signals, 2H, Ar-***H***), 7.13 – 7.23 (overlapping signals, 6H, Ar-***H***), 7.32 (m, 2H, Ar-***H***).

^{13}C NMR (101 MHz, C_6D_6, 298 K) δ 22.2 (***C***H$_3$C$_6$H$_4$), 23.3 ({(***C***H$_3$)$_2$C}$_2$CH), 23.5 ({(***C***H$_3$)$_2$C}$_2$CH), 23.5 ({(***C***H$_3$)$_2$C}$_2$CH), 23.6 ({(***C***H$_3$)$_2$C}$_2$CH), 24.4 ((***C***H$_3$)$_2$CH), 24.5 ((***C***H$_3$)$_2$CH), 24.5 ((***C***H$_3$)$_2$CH), 24.5 ((***C***H$_3$)$_2$CH), 24.6 ((***C***H$_3$)$_2$CH), 24.7 ((***C***H$_3$)$_2$CH), 24.7

(($\mathit{C}H_3)_2CH$), 24.7 (($\mathit{C}H_3)_2CH$), 24.8 (($\mathit{C}H_3)_2CH$), 24.8 (($\mathit{C}H_3)_2CH$), 24.8 (($\mathit{C}H_3)_2CH$), 24.9 (($\mathit{C}H_3)_2CH$), 25.0 (($\mathit{C}H_3)_2CH$), 25.3 (($\mathit{C}H_3)_2CH$), 25.4 (($\mathit{C}H_3)_2CH$), 25.6 (($\mathit{C}H_3)_2CH$), 28.1 (($CH_3)_2\mathit{C}H$), 28.2 (($CH_3)_2\mathit{C}H$), 28.3 (($CH_3)_2\mathit{C}H$), 28.6 (($CH_3)_2\mathit{C}H$), 28.7 (($CH_3)_2\mathit{C}H$), 28.7 (($CH_3)_2\mathit{C}H$), 28.8 (($CH_3)_2\mathit{C}H$), 29.0 (($CH_3)_2\mathit{C}H$), 68.6 ($\mathit{C}H_2$), 79.8 (C^4H), 97.6 ($\{(CH_3)_2C\}_2\mathit{C}H$), 97.9 ($\{(CH_3)_2C\}_2\mathit{C}H$), 123.6 (Ar*C*), 123.7 (Ar*C*), 123.9 (Ar*C*), 124.2 (Ar*C*), 124.4 (Ar*C*), 124.5 (Ar*C*), 124.7 (Ar*C*), 124.8 (Ar*C*), 124.9 (Ar*C*), 125.4 (Ar*C*), 125.9 (Ar*C*), 126.1 (Ar*C*), 127.4 (Ar*C*), 129.1 (Ar*C*), 129.7 (Ar*C*), 135.7 (C^2), 136.0 (C^3), 136.8 (Ar*C*), 139.4 (Ar*C*), 139.6 (Ar*C*), 139.7 (Ar*C*), 140.3 (Ar*C*), 141.3 (Ar*C*), 142.8 (Ar*C*), 144.0 (Ar*C*), 144.2 (Ar*C*), 144.3 (Ar*C*), 144.5 (Ar*C*), 144.7 (Ar*C*), 144.8 (Ar*C*), 145.2 (Ar*C*), 148.0 (Ar*C*), 171.3 ($\{(CH_3)_2\mathit{C}\}_2CH$), 171.4 ($\{(CH_3)_2\mathit{C}\}_2CH$), 171.5 ($\{(CH_3)_2\mathit{C}\}_2CH$), 171.6 ($\{(CH_3)_2\mathit{C}\}_2CH$).

Preparation of ***3d***

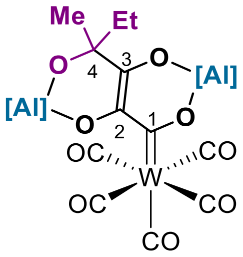

2 was prepared *in-situ* in an NMR tube from **1** (8.9 mg, 0.02 mmol), [W(CO)$_6$] (4 mg, 0.011 mmol) and CO gas (~1 bar) following the reported procedure.[2] Once formation of **2** was confirmed by ^{1}H NMR spectroscopy, the NMR tube was returned to the glovebox, the reaction mixture was cooled to –35 °C using a low temperature reactor and the headspace of the NMR tube was evacuated to remove the remaining CO gas and 2-butanone (1.8 μL, 0.02 mmol) was added via microsyringe. After 24 h at 25 °C reaction mixture was returned to the glovebox, diluted with ~0.5 mL of toluene and decanted into a 20 mL scintillation vial. The resultant solution was concentrated *in vacuo* to ~0.2 mL, filtered into a 4 mL vial, and carefully layered with n-pentane (~2 mL). The vial was placed in the glovebox freezer (–35 °C) and **3d** crystallised as yellow blocks. The supernatant was decanted, and the resultant crystals were washed with cold n-pentane thrice (3 x 0.5mL) before being dried briefly *in vacuo* (~ 2 min). Yield: 7.0 mg, 0.0051 mmol, 51%.

^{1}H NMR (400 MHz, C$_6$D$_6$, 298 K) δ -0.03 (t, $^3J_{HH}$ = 7.3 Hz, 3H, CH$_2$C***H***$_3$), 0.49 (s, 3H, C***H***$_3$C^4), 0.68 (d, $^3J_{HH}$ = 6.7 Hz, 3H, (C***H***$_3$)$_2$CH), 0.77 (d, $^3J_{HH}$ = 6.8 Hz, 3H, (C***H***$_3$)$_2$CH), 0.81 (d, $^3J_{HH}$ = 6.8 Hz, 3H, (C***H***$_3$)$_2$CH), 0.90 (m, 2H, C***H***$_2$CH$_3$), 1.03 (d, $^3J_{HH}$ = 6.8 Hz, 3H, (C***H***$_3$)$_2$CH), 1.06 (d, $^3J_{HH}$ = 6.8 Hz, 3H, (C***H***$_3$)$_2$CH), 1.13 (d, $^3J_{HH}$ = 6.7 Hz, 3H, (C***H***$_3$)$_2$CH), 1.16 (d, $^3J_{HH}$ = 6.8 Hz, 3H, (C***H***$_3$)$_2$CH), 1.19 (d, $^3J_{HH}$ = 6.9 Hz, 6H, (C***H***$_3$)$_2$CH), 1.25 (overlapping signals, 3H, (C***H***$_3$)$_2$CH), 1.27 (d, $^3J_{HH}$ = 6.6 Hz, 3H, (C***H***$_3$)$_2$CH), 1.34 (overlapping signals, 3H, (C***H***$_3$)$_2$CH), 1.35 (s, 3H, {(C***H***$_3$)$_2$C}$_2$CH), 1.35 (d, $^3J_{HH}$ = 6.8 Hz, 3H, (C***H***$_3$)$_2$CH), 1.38 (s, 3H, {(C***H***$_3$)$_2$C}$_2$CH), 1.41 (d, $^3J_{HH}$ = 6.8 Hz, 3H, (C***H***$_3$)$_2$CH), 1.48 (s, 3H, {(C***H***$_3$)$_2$C}$_2$CH), 1.50 (s, 3H, {(C***H***$_3$)$_2$C}$_2$CH), 1.64 (d, $^3J_{HH}$ = 6.8 Hz, 3H, (C***H***$_3$)$_2$CH), 1.77 (d, $^3J_{HH}$ = 6.8 Hz, 3H, (C***H***$_3$)$_2$CH), 2.86 (hept, $^3J_{HH}$ = 6.8 Hz, 1H, (CH$_3$)$_2$C***H***), 3.01 - 3.20 (hept overlapping, 5H, (CH$_3$)$_2$C***H***), 3.62 (hept, $^3J_{HH}$ = 6.6 Hz, 1H, (CH$_3$)$_2$C***H***), 3.73 (hept, $^3J_{HH}$ = 6.7 Hz, 1H, (CH$_3$)$_2$C***H***), 4.77 (s, 1H, {(CH$_3$)C}$_2$C***H***), 4.98 (s, 1H, {(CH$_3$)C}$_2$C***H***), 6.88 (m, 1H, Ar-***H***), 6.99 (m, 4H, Ar-***H***), 7.07 (m, 2H, Ar-***H***), 7.10 - 7.20 (overlapping signals, 5H, Ar-***H***).

$^{13}C\{^1H\}$ NMR (101 MHz, C_6D_6, 298 K) δ 7.5 ($CH_2\boldsymbol{C}H_3$), 23.1 ($(\boldsymbol{C}H_3)_2CH$), 23.3 ($(\boldsymbol{C}H_3)_2CH$), 23,4 ($(\boldsymbol{C}H_3)_2CH$), 23.6 ($\{(\boldsymbol{C}H_3)_2C\}_2CH$), 23.8 ($\{(\boldsymbol{C}H_3)_2C\}_2CH$), 24.1 ($(\boldsymbol{C}H_3)_2CH$), 24.1 ($(\boldsymbol{C}H_3)_2CH$), 24.2 ($(\boldsymbol{C}H_3)_2CH$), 24.4 ($(\boldsymbol{C}H_3)_2CH$), 24.5 ($\boldsymbol{C}H_3)_2CH$), 24.6 ($\{(\boldsymbol{C}H_3)_2C\}_2CH$), 24.6 ($\{(\boldsymbol{C}H_3)_2C\}_2CH$), 24.7 ($\boldsymbol{C}H_3)_2CH$), 24.9 ($\boldsymbol{C}H_3)_2CH$), 25.0 ($\boldsymbol{C}H_3)_2CH$), 25.3 ($\boldsymbol{C}H_3)_2CH$), 25.3 ($\boldsymbol{C}H_3)_2CH$), 26.8 ($\boldsymbol{C}H_3)_2CH$), 27.7 ($\boldsymbol{C}H_3)_2CH$), 27.9 ($(\boldsymbol{C}H_3)_2CH$), 28.3 ($(CH_3)_2\boldsymbol{C}H$), 28.4 ($(CH_3)_2\boldsymbol{C}H$), 28.7 ($(CH_3)_2\boldsymbol{C}H$), 28.7 ($(CH_3)_2\boldsymbol{C}H$), 28.7 ($\boldsymbol{C}H_3C^4$), 28.9 ($(CH_3)_2\boldsymbol{C}H$), 29.0 ($(CH_3)_2\boldsymbol{C}H$), 29.1 ($(CH_3)_2\boldsymbol{C}H$), 29.5 ($(CH_3)_2\boldsymbol{C}H$), 35.4 ($\boldsymbol{C}H_2CH_3$), 78.4 ($\boldsymbol{C}^4$), 99.8 ($\{(CH_3)_2C\}_2\boldsymbol{C}H$), 99.9 ($\{(CH_3)_2C\}_2\boldsymbol{C}H$), 123.6 (Ar$\boldsymbol{C}$), 123.9 (Ar$\boldsymbol{C}$), 124.0 (Ar$\boldsymbol{C}$), 124.1 (Ar$\boldsymbol{C}$), 124.6 (Ar$\boldsymbol{C}$), 124.8 (Ar$\boldsymbol{C}$), 125.4 (Ar$\boldsymbol{C}$), 125.9 (Ar$\boldsymbol{C}$), 127.2 (Ar$\boldsymbol{C}$), 127.5 (Ar$\boldsymbol{C}$), 139.0 (Ar$\boldsymbol{C}$), 139.3 (Ar$\boldsymbol{C}$), 140.3 (Ar$\boldsymbol{C}$), 140.4 (Ar$\boldsymbol{C}$), 142.1 (Ar$\boldsymbol{C}$), 142.6 (Ar$\boldsymbol{C}$), 143.8 (Ar$\boldsymbol{C}$), 144.9 (Ar$\boldsymbol{C}$), 144.8 (Ar$\boldsymbol{C}$), 145.4 (Ar$\boldsymbol{C}$), 146.3 (Ar$\boldsymbol{C}$), 146.4 (Ar$\boldsymbol{C}$), 154.9 ($\boldsymbol{C}^3$), 157.2 ($\boldsymbol{C}^2$), 172.1 ($\{(CH_3)_2\boldsymbol{C}\}_2CH$), 172.3 ($\{(CH_3)_2\boldsymbol{C}\}_2CH$), 173.8 ($\{(CH_3)_2\boldsymbol{C}\}_2CH$), 174,3 ($\{(CH_3)_2\boldsymbol{C}\}_2CH$), 203.6 ($W(\boldsymbol{C}O)_4$), 205.2 ($W(\boldsymbol{C}O)$), 311.3 ($\boldsymbol{C}^1$). Some Ar$\boldsymbol{C}$ resonances are overlapping and cannot be observed.

IR (ATR), ν_{CO} (cm^{-1}): 2043, 1923, 1893, 1871.

Anal. Calc. ($C_{71}H_{92}Al_2N_4O_9W$): C, 61.65; H, 6.70; N, 4.05. Found: C, 61.79; H, 6.65; N, 4.49.

Preparation of ***5d***

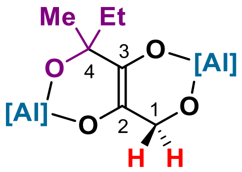

In a glovebox, **3d** (5 mg, 0.0036 mmol) was dissolved in C_6D_6 (0.6 mL) and transferred to a J-Young NMR tube containing a capillary with 1,3,5-trimethoxybenzene as external standard. The headspace of the NMR tube was evacuated, H_2 gas (~1 bar) was introduced into the NMR tube and the reaction mixture was heated for 4 h at 100 °C. After this time, **3d** was not observed by ^{1}H NMR spectroscopy in the reaction mixture. NMR yield: 87%. Compound **5d** was characterized by NMR spectroscopy.

^{1}H NMR (400 MHz, C_6D_6, 298 K) δ -0.37 (t, $^3J_{HH}$ = 7.2 Hz, 3H, $CH_2C\boldsymbol{H}_3$), 0.56 (s, 3H, $C\boldsymbol{H}_3C^4$), 0.76 (d, $^3J_{HH}$ = 6.7 Hz, 3H, $(C\boldsymbol{H}_3)_2CH)$)), 0.81 (m, 1H, $C\boldsymbol{H}_2CH_3$), 0.98 (d, $^3J_{HH}$ = 6.8 Hz, 3H, $(C\boldsymbol{H}_3)_2CH$), 1.03 (d, $^3J_{HH}$ = 6.8 Hz, 6H, $(C\boldsymbol{H}_3)_2CH$), 1.03 (overlapping signals, 1H $C\boldsymbol{H}_2CH_3$), 1.12 (d, $^3J_{HH}$ = 6.8 Hz, 6H, $(C\boldsymbol{H}_3)_2CH$), 1.14 (d, $^3J_{HH}$ = 6.8 Hz, 6H, $(C\boldsymbol{H}_3)_2CH$), 1.17 (d, $^3J_{HH}$ = 6.8 Hz, 6H, $(C\boldsymbol{H}_3)_2CH$), 1.21 (d, $^3J_{HH}$ = 6.9 Hz, 3H, $(C\boldsymbol{H}_3)_2CH$), 1.30 (d, $^3J_{HH}$ = 6.7 Hz, 3H, $(C\boldsymbol{H}_3)_2CH$), 1.46 (d, $^3J_{HH}$ = 6.7 Hz, 6H, $(C\boldsymbol{H}_3)_2CH$), 1.48 (s, 6H, $\{(C\boldsymbol{H}_3)_2C\}_2CH$), 1.52 (d, $^3J_{HH}$ = 6.7 Hz, 3H, $(C\boldsymbol{H}_3)_2CH$), 1.56 (s, 6H, $\{(C\boldsymbol{H}_3)_2C\}_2CH$), 1.59 (d, $^3J_{HH}$ = 6.6 Hz, 3H, $(C\boldsymbol{H}_3)_2CH$), 2.98 (hept, $^3J_{HH}$ = 6.9 Hz, 2H, $(CH_3)_2C\boldsymbol{H}$), 3.08 (hept, $^3J_{HH}$ = 6.7 Hz, 2H, $(CH_3)_2C\boldsymbol{H}$), 3.24 – 3.58 (hept overlapping, 4H, $(CH_3)_2C\boldsymbol{H}$), 4.67 (s, 2H, $C\boldsymbol{H}_2$), 4.90 (s, 1H, $\{(CH_3)C\}_2C\boldsymbol{H}$), 4.93 (s, 1H, $\{(CH_3)C\}_2C\boldsymbol{H}$), 6.94 (m, 1H, Ar-***H***), 7.02 (m, 2H, Ar-***H***), 7.10 - 7.20 (overlapping signals, 9H, Ar-***H***).

^{13}C NMR (101 MHz, C_6D_6, 298 K) δ 6.9 ($CH_2\boldsymbol{C}H_3$), 22.7 ($(\boldsymbol{C}H_3)_2CH$), 23.4 ($(\boldsymbol{C}H_3)_2CH$), 23.4 ($(\boldsymbol{C}H_3)_2CH$), 23.5 ($\{(\boldsymbol{C}H_3)_2C\}_2CH$), 23.5 ($\{(\boldsymbol{C}H_3)_2C\}_2CH$), 23.8 ($(\boldsymbol{C}H_3)_2CH$), 23.9 ($(\boldsymbol{C}H_3)_2CH$), 24.0 ($(\boldsymbol{C}H_3)_2CH$), 24.1 ($(\boldsymbol{C}H_3)_2CH$), 24.4 ($(\boldsymbol{C}H_3)_2CH$), 24.5 ($\{(\boldsymbol{C}H_3)_2C\}_2CH$), 24.5 ($\{(\boldsymbol{C}H_3)_2C\}_2CH$), 24.7 ($(\boldsymbol{C}H_3)_2CH$), 24.8 ($(\boldsymbol{C}H_3)_2CH$), 24.8 ($(\boldsymbol{C}H_3)_2CH$), 24.9 ($(\boldsymbol{C}H_3)_2CH$), 25.3 ($(\boldsymbol{C}H_3)_2CH$), 25.5 ($(\boldsymbol{C}H_3)_2CH$), 26.2 ($(\boldsymbol{C}H_3)_2CH$), 26.7 ($(\boldsymbol{C}H_3)_2CH$), 27.9 ($(CH_3)_2\boldsymbol{C}H$), 28.0 ($(CH_3)_2\boldsymbol{C}H$), 28.2 ($(CH_3)_2\boldsymbol{C}H$), 28.5 ($(CH_3)_2\boldsymbol{C}H$), 28.7 ($(CH_3)_2\boldsymbol{C}H$), 28.8 ($(CH_3)_2\boldsymbol{C}H$), 28.8 ($(CH_3)_2\boldsymbol{C}H$) 28.9 ($(CH_3)_2\boldsymbol{C}H$), 30.3 ($\boldsymbol{C}H_3C^4$), 35.1 ($\boldsymbol{C}H_2CH_3$), 69.1 ($C\boldsymbol{H}_2$), 78.4 ($\boldsymbol{C}^4$), 97.3 ($\{(CH_3)_2C\}_2\boldsymbol{C}H$), 97.8 ($\{(CH_3)_2C\}_2\boldsymbol{C}H$), 123.6 (Ar***C***), 123.7 (Ar***C***), 124.0 (Ar***C***), 124.1 (Ar***C***), 124.2 (Ar***C***), 124.5 (Ar***C***), 124.8 (Ar***C***), 125.5 (Ar***C***), 125.9 (Ar***C***), 135.3 ($\boldsymbol{C}^2$), 138.3 ($\boldsymbol{C}^3$),

140.0 (Ar***C***), 140.5 (Ar***C***), 140.6 (Ar***C***), 141.1 (Ar***C***), 142.8 (Ar***C***), 143.3 (Ar***C***), 143.4 (Ar***C***), 144.1 (Ar***C***), 144.2 (Ar***C***), 144.5 (Ar***C***), 144.9 (Ar***C***), 145.4 (Ar***C***), 171.0 ($\{(CH_3)_2$***C***$\}_2$CH), 171.3 ($\{(CH_3)_2$***C***$\}_2$CH), 171.7 ($\{(CH_3)_2$***C***$\}_2$CH), 172.2 ($\{(CH_3)_2$***C***$\}_2$CH). Some Ar***C*** resonances are overlapping and cannot be observed.

*Preparation of **3e***

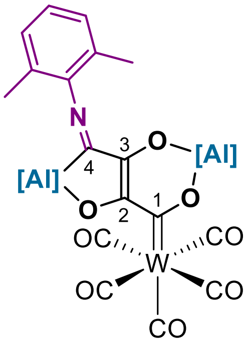

2 was prepared *in-situ* in an NMR tube from **1** (8.9 mg, 0.02 mmol), [W(CO)$_6$] (4 mg, 0.011 mmol) and CO gas (~1 bar) following the reported procedure.[2] Once formation of **2** was confirmed by ^{1}H NMR spectroscopy, the NMR tube was returned to the glovebox, reaction mixture was cooled to −35 °C using a low temperature reactor and the headspace of the NMR tube was evacuated to remove the remaining CO gas. 2,6-Dimethylphenyl isocyanide (1.3 mg, 0.01 mmol) was dissolved in 0.3 mL of C_6D_6 and added to the reaction mixture. After 24 h at 60 °C reaction mixture was returned to the glovebox, diluted with ~0.5 mL of toluene and decanted into a 20 mL scintillation vial. The resultant solution was concentrated *in vacuo* and the residue dissolved in ~0.2 mL of THF. A dark purple solid precipitated after addition of n-pentane (~0.5 mL) to the solution. The supernatant was decanted, and the solid washed with n-pentane thrice (3 x 0.5mL) before being dried *in vacuo*. Yield: 8.5 mg, 0.006 mmol, 60%.

^{1}H NMR (400 MHz, THF-*d*$_8$, 298 K) δ 0.14 (d, $^3J_{HH}$ = 6.7 Hz, 6H, (C***H***$_3$)$_2$CH), 0.60 (s, 6H, Xyl-C***H***$_3$), 0.74 (d, $^3J_{HH}$ = 6.7 Hz, 6H, (C***H***$_3$)$_2$CH), 0.87 (d, $^3J_{HH}$ = 6.7 Hz, 6H, (C***H***$_3$)$_2$CH), 0.92 (d, $^3J_{HH}$ = 6.8 Hz, 6H, (C***H***$_3$)$_2$CH), 1.00 (d, $^3J_{HH}$ = 6.6 Hz, 6H, (C***H***$_3$)$_2$CH), 1.18 (d, $^3J_{HH}$ = 6.7 Hz, 6H, (C***H***$_3$)$_2$CH), 1.26 (d, $^3J_{HH}$ = 6.7 Hz, 6H, (C***H***$_3$)$_2$CH), 1.54 (d, $^3J_{HH}$ = 6.9 Hz, 6H, (C***H***$_3$)$_2$CH), 1.64 (s, 6H, {(C***H***$_3$)$_2$C}$_2$CH), 1.84 (s, 6H, {(C***H***$_3$)$_2$C}$_2$CH), 2.37 (hept, $^3J_{HH}$ = 6.9 Hz, 2H, (CH$_3$)$_2$C***H***), 2.98 (hept, $^3J_{HH}$ = 6.8 Hz, 2H, (CH$_3$)$_2$C***H***), 3.25 (hept, $^3J_{HH}$ = 6.7 Hz, 2H, (CH$_3$)$_2$C***H***), 3.33 (hept, $^3J_{HH}$ = 6.9 Hz, 2H, (CH$_3$)$_2$C***H***), 5.48 (s, 1H, {(CH$_3$)C}$_2$C***H***), 5.66 (s, 1H, {(CH$_3$)C}$_2$C***H***), 6.41 (m, 3H, Ar-***H***), 6.96 (m, 2H, Ar-***H***), 7.02 – 7.10 (overlapping signals, 4H, Ar-***H***), 7.15 (m, 3H, Ar-***H***), 7.24 (m, 3H, Ar-***H***).

^{13}C NMR (101 MHz, THF, 298 K) δ 17.1 (Xyl-***C**H₃*), 24.2 (2x (***C***$H_3)_2$CH), 24.5 (2x {(***C***$H_3)_2$C}$_2$CH), 24.5 (2x {(***C***$H_3)_2$C}$_2$CH), 26.3 (2x (***C***$H_3)_2$CH), 26.4 (2x (***C***$H_3)_2$CH), 28.1 (2x (***C***$H_3)_2$CH), 28.6 (2x (***C***$H_3)_2$CH), 29.3 (2x (***C***$H_3)_2$CH), 29.4 (2x $(CH_3)_2$***C***H), 30.0 (2x $(CH_3)_2$***C***H), 100.5 ({$(CH_3)_2$C}$_2$***C***H), 102.2 ({$(CH_3)_2$C}$_2$***C***H), 121.9 (Ar***C***), 124.0 (Ar***C***), 124.9 (Ar***C***), 125.3 (Ar***C***), 125.5 (Ar***C***), 125.8 (Ar***C***), 128.2 (Ar***C***), 128.6 (Ar***C***), 129.0 (Ar***C***), 139.3 (Ar***C***), 140.8 (Ar***C***), 144.2 (Ar***C***), 145.0 (Ar***C***), 145.0 (Ar***C***), 145.8 (***C***$^{2/3}$), 146.5 (Ar***C***), 156.5 (Ar***C***), 161.2 (***C***$^{2/3}$) 173.8 ({$(CH_3)_2$***C***}$_2$CH), 175.0 ({$(CH_3)_2$***C***}$_2$CH), 202.5 (W(***C***O)$_4$), 206.5 (W(***C***O)$_4$), 329.2 (***C***^{1}). Some Ar***C*** resonances are overlapping and cannot be observed. The Al–***C***^{4} resonance could not be observed in the ^{13}C NMR spectrum due to coupling to the quadrupolar ^{27}Al (I = 5/2) nucleus.

IR (ATR), ν_{CO} (cm^{-1}): 2051, 1907, 1879.

Anal. Calc. ($C_{75}H_{91}Al_2N_5O_8W$): C, 63.07; H, 6.42; N, 4.90. Found: C, 51.79; H, 4.95; N, 4.50.[1]

[1] Accurate CHN analysis could not be obtained likely due to the air-sensitive nature of the compound.

Preparation of ***S2***

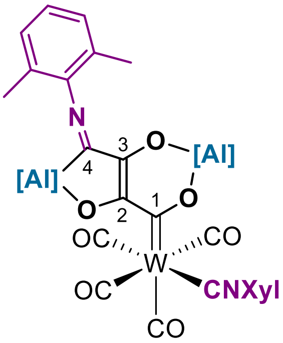

In a glovebox, **3f** (24 mg, 0.019 mmol) and 2,6-dimethylphenyl isocyanide (7 mg, 0.053 mmol) were dissolved in C_6D_6 (0.600 mL) and transferred to a J-Young NMR tube. The mixture was heated at 100°C for 6 h. At 2 hr intervals, the headspace of the NMR tube was removed under vacuum and refreshed with dinitrogen. The J-Young NMR tube was returned to the glovebox, diluted with toluene (~0.5 mL), decanted into a 20 mL scintillation vial, and concentrated *in vacuo* until approx 0.3 mL of solution remains. The solution was then filtered into 10 mL of n-pentane. **S2** directly crystallises from this mixture as dark purple-black needles. The vial was placed in the freezer at –35°C for 18 h. The supernatant was decanted, and the resultant crystals were washed with cold n-pentane thrice (3x 1mL) before the crystals were dried briefly *in vacuo* (~2 minutes). Yield: 14 mg, 0.0091 mmol, 48%.

^{1}H NMR (400 MHz, C_6D_6, 298 K) δ 0.46 (d, $^3J_{HH}$ = 6.7 Hz, 6H, (C***H***$_3$)$_2$CH), 0.87 (d overlapping, $^3J_{HH}$= 6.7 Hz, 12H, 2x (C***H***$_3$)$_2$CH), 0.92 (d, $^3J_{HH}$ = 6.8 Hz, 6H, (C***H***$_3$)$_2$CH), 0.95 (d, $^3J_{HH}$ = 6.8 Hz, 6H, (C***H***$_3$)$_2$CH), 1.03 (s, 6H, 2x Xyl-C***H***$_3$), 1.37 (s, 6H, {(C***H***$_3$)$_2$C}$_2$CH), 1.40 (d, $^3J_{HH}$ = 6.8 Hz, 6H, (C***H***$_3$)$_2$CH), 1.44 (d, $^3J_{HH}$ = 6.5 Hz, 6H, (C***H***$_3$)$_2$CH), 1.49 (s, 6H, {(C***H***$_3$)$_2$C}$_2$CH), 1.78 (d, $^3J_{HH}$ = 6.8 Hz, 6H, (C***H***$_3$)$_2$CH), 2.51 (hept, $^3J_{HH}$ = 6.7 Hz, 2H, 2x (CH$_3$)$_2$C***H***), 2.53 (s, 6H, 2x Xyl-C***H***$_3$), 3.06 (hept, $^3J_{HH}$ = 6.8 Hz, 2H, 2x (CH$_3$)$_2$C***H***), 3.18 (hept, $^3J_{HH}$ = 6.8 Hz, 2H, 2x (CH$_3$)$_2$C***H***), 3.74 (hept, $^3J_{HH}$ = 6.6 Hz, 2H, 2x (CH$_3$)$_2$C***H***), 4.95 (s, 1H, {(CH$_3$)C}$_2$C***H***), 5.21 (s, 1H, {(CH$_3$)C}$_2$C***H***), 6.67 – 7.23 (m, overlapping signals, 24H, Ar-***H***).

^{13}C NMR (101 MHz, C_6D_6, 298 K) δ 17.0 (2x Xyl-CH$_3$), 19.1 (2x Xyl-***C***H$_3$), 23.9 ({(***C***H$_3$)$_2$C}$_2$CH), 24.0 ({(***C***H$_3$)$_2$C}$_2$CH), 24.1 ({(***C***H$_3$)$_2$C}$_2$CH), 24.8 (2x (***C***H$_3$)$_2$CH), 25.3 ((***C***H$_3$)$_2$CH), 25.3 ((***C***H$_3$)$_2$CH), 25.5 ((***C***H$_3$)$_2$CH), 26.0 ((***C***H$_3$)$_2$CH), 27.7 ((***C***H$_3$)$_2$CH), 28.5 (2x (CH$_3$)$_2$***C***H), 28.7 (4x (CH$_3$)$_2$***C***H), 29.4 (2x (CH$_3$)$_2$***C***H), 99.3 ({(CH$_3$)$_2$C}$_2$***C***H), 102.0

($\{(CH_3)_2C\}_2\boldsymbol{C}H$), 121.5 (Ar***C***), 123.7 (Ar***C***), 124.2 (2x Ar***C***), 124.7 (2x Ar***C***), 125.1 (2x Ar***C***), 125.1 (2x Ar***C***), 126.8 (Ar***C***), 127.6 (Ar***C***), 129.9 (Ar***C***), 135.0 (Ar***C***), 138.7 (Ar***C***), 140.6 (Ar***C***), 143.6 (Ar***C***), 144.4 (Ar***C***), 144.4 (Ar***C***), 146.5 (Ar***C***), 156.5 ($\boldsymbol{C}^{2/3}$), 160.9 ($\boldsymbol{C}^{2/3}$), 173.1 ($\{(CH_3)_2\boldsymbol{C}\}_2CH$), 173.3 ($\{(CH_3)_2\boldsymbol{C}\}_2CH$), 205.8 ($W(\boldsymbol{C}O)_2$), 211.4 ($W(\boldsymbol{C}O)_2$), 319.4 ($\boldsymbol{C}^{1}$). Some Ar***C*** resonances are overlapping and cannot be observed. The Al–***C*** resonance could not be observed due to quadrupolar broadening to the ^{27}Al (I = 5/2) nucleus.

IR (ATR), ν_{CO} (cm^{-1}): 1976, 1911, 1889, 1859.

Anal. Calc. ($C_{83}H_{100}Al_2N_6O_7W$): C, 65.09;H, 6.58; N, 5.49. Found: C, 65.22; H, 6.73; N, 5.44.

*Preparation of **S3***

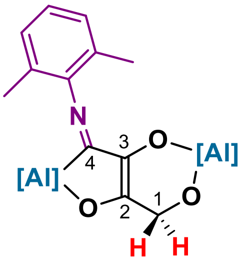

In a glovebox, **3e** (5 mg, 0.0035 mmol) was dissolved in C_6D_6 (0.6 mL) and transferred to a J-Young NMR tube containing a capillary with 1,3,5-trimethoxybenzene as external standard. The headspace of the NMR tube was evacuated, H_2 gas (~1 bar) was introduced into the NMR tube and the reaction mixture was heated for 16 h at 100 °C. After this time, reaction mixture contained **3e**, **S3** and **5e** in 7, 60 and 20 % NMR yield, respectively. Compound **S3** was characterized by NMR spectroscopy from the mixture.

^{1}H NMR (400 MHz, C_6D_6, 298 K) δ 0.71 (d, $^3J_{HH}$ = 6.8 Hz, 3H, (C***H**3*)2CH)), 0.88 (d, $^3J_{HH}$ = 6.7 Hz, 6H, (C***H**3*)2CH)), 0.96 (d, $^3J_{HH}$ = 6.6 Hz, 6H, (C***H**3*)2CH)), 1.12 (d, $^3J_{HH}$ = 6.6 Hz, 6H, (C***H**3*)2CH)), 1.21 (d, $^3J_{HH}$ = 6.7 Hz, 6H, (C***H**3*)2CH)), 1.39 (s, 6H, {(C***H**3*)2C}2CH), 1.51 (s, 6H, {(C***H**3*)2C}2CH), 1.53 (d, $^3J_{HH}$ = 6.7 Hz, 6H, (C***H**3*)2CH)), 1.56 (d, $^3J_{HH}$ = 6.7 Hz, 6H, (C***H**3*)2CH)), 1.61 (d, $^3J_{HH}$ = 6.7 Hz, 6H, (C***H**3*)2CH)), 1.86 (s, 6H, Xyl-C***H3***), 2.04 (m, 3H, (C***H**3*)2CH)), 2.54 (hept, $^3J_{HH}$ = 6.6 Hz, 2H, (CH3)2C***H***), 3.19 (hept, $^3J_{HH}$ = 6.7 Hz, 2H, (CH3)2C***H***), 3.58 (hept, $^3J_{HH}$ = 6.7 Hz, 2H, (CH3)2C***H***), 3.63 (hept, $^3J_{HH}$ = 6.8 Hz, 2H, (CH3)2C***H***), 4.86 (s, 2H, C***H***2), 4.90 (s, 1H, {(CH3)C}2C***H***), 4.99 (s, 1H, {(CH3)C}2C***H***), 6.44-7.34 (overlapping signals, 15H, Ar-***H***).

^{13}C NMR (101 MHz, C_6D_6, 298 K) δ 68.7 (C***H***2), 97.2 ({(CH3)2C}2***C***H), 98.6 ({(CH3)2C}2***C***H), 146.3 (***C***^{3}), 156.2 (***C***^{2}), 171.2 ({(CH3)2***C***}2CH), 171.8 ({(CH3)2***C***}2CH). Resonances corresponding to Xyl-***C***H3, (***C***H3)2CH, {(***C***H3)2C}2CH, (CH3)2***C***H and Ar***C*** are overlapping and cannot be assigned. The Al–***C***^{4} resonance could not be observed in the ^{13}C NMR spectrum due to coupling to the quadrupolar ^{27}Al (I = 5/2) nucleus.

Preparation of ***5e***

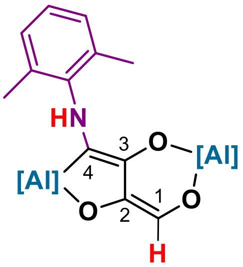

In a glovebox, **3e** (5 mg, 0.0035 mmol) was dissolved in C_6D_6 (0.6 mL) and transferred to a J-Young NMR tube containing a capillary with 1,3,5-trimethoxybenzene as external standard. The headspace of the NMR tube was evacuated, H_2 gas (~1 bar) was introduced into the NMR tube and the reaction mixture was heated for 4 days at 100 °C. After this time, no signals corresponding to **3e** or **S2** were observed by NMR spectroscopy from the mixture. NMR yield: 85%. Compound **5e** was characterized by NMR spectroscopy.

^{1}H NMR (400 MHz, C_6D_6, 298 K) δ 0.62 (d, $^3J_{HH}$ = 6.6 Hz, 3H, (C***H**3*)2CH)), 1.02 (d, $^3J_{HH}$ = 6.9 Hz, 6H, (C***H**3*)2CH)), 1.04 (d, $^3J_{HH}$ = 6.8 Hz, 6H, (C***H**3*)2CH)), 1.07 (d, $^3J_{HH}$ = 6.8 Hz, 6H, (C***H**3*)2CH)),), 1.11 (d, $^3J_{HH}$ = 6.7 Hz, 6H, (C***H**3*)2CH)),), 1.20 (d, $^3J_{HH}$ = 6.9 Hz, 6H, (C***H**3*)2CH)),), 1.30 (d, $^3J_{HH}$ = 6.8 Hz, 6H, (C***H**3*)2CH)), 1.36 (d, $^3J_{HH}$ = 6.7 Hz, 6H, (C***H**3*)2CH)), 1.47 (s, 6H, {(C***H**3*)2C}2CH), 1.48 (s, 6H, {(C***H**3*)2C}2CH), 1.86 (s, 6H, Xyl-C***H3***), 2.09 (m, 3H, (C***H**3*)2CH)), 3.06 (hept, $^3J_{HH}$ = 6.8 Hz, 2H, (CH3)2C***H***), 3.08 (hept, $^3J_{HH}$ = 6.8 Hz, 2H, (CH3)2C***H***), 3.31 (hept, $^3J_{HH}$ = 6.9 Hz, 2H, (CH3)2C***H***),), 3.68 (hept, $^3J_{HH}$ = 6.8 Hz, 2H, (CH3)2C***H***),), 4.90 (s, 1H, {(CH3)C}2C***H***), 4.94 (s, 1H, {(CH3)C}2C***H***), 5.61 (s, 1H, N***H***), 6.47 (s, 1H, C^1***H***), 6.50 (m, 2H, Ar-***H***), 6.68 (m, 1H, Ar-***H***), 6.79 (m, 1H, Ar-***H***), 6.98 (m, 2H, Ar-***H***), 7.12 – 7.22 (overlapping signals, 9H, Ar-***H***).

^{13}C NMR (101 MHz, C_6D_6, 298 K) δ 18.3 (Xyl-***C***H3), 19.8 (2x (***C***H3)2CH), 23.3 (2x {(***C***H3)2C}2CH), 23.6 (2x {(***C***H3)2C}2CH), 24.1 (2x (***C***H3)2CH), 24.4 (2x (***C***H3)2CH), 24.7 (2x (***C***H3)2CH), 24.9 (2x (***C***H3)2CH), 25.2 (2x (***C***H3)2CH), 25.3 (2x (***C***H3)2CH), 25.7 (2x (***C***H3)2CH), 28.3 (2x (CH3)2***C***H), 28.6 (2x (CH3)2***C***H), 28.6 (2x (CH3)2***C***H), 28.7 (2x (CH3)2***C***H), 98.6 ({(CH3)2C}2***C***H), 99.1 ({(CH3)2C}2***C***H), 119.3 (Ar***C***), 124.2 (Ar***C***), 124.3 (Ar***C***), 124.4 (Ar***C***), 125.1 (Ar***C***), 127.1 (Ar***C***), 127.4 (Ar***C***), 128.6 (***C***^{1}H), 129.2 (Ar***C***), 135.3 (Ar***C***), 138.6 (***C***^{2}), 139.5 (Ar***C***), 140.4 (Ar***C***), 143.2 (Ar***C***), 144.7 (Ar***C***), 145.1 (Ar***C***), 145.5 (Ar***C***), 146.3 (Ar***C***), 151.2 (***C***^{3}), 171.0 ({(CH3)2***C***}2CH), 171.8 ({(CH3)2***C***}2CH). Some Ar***C*** resonances are overlapping and cannot be observed. The Al–***C***^{4} resonance could not be

observed in the ^{13}C NMR spectrum due to coupling to the quadrupolar ^{27}Al (I = 5/2) nucleus.

NH group was confirmed by ^{15}N-^{1}H HSQC experiment. ^{15}N NMR (41 MHz, 298 K) δ 79.5.

Preparation of ***5f***

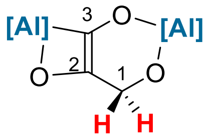

In a glovebox, **2** (10 mg, 0.008 mmol) was dissolved in C_6D_6 (0.6 mL) and transferred to a J-Young NMR tube. The headspace of the NMR tube was evacuated, H_2 gas (~1 bar) was introduced into the NMR tube and the reaction mixture was heated for 72 h at 100 °C. After this time, conversion >95% of **2** to **5f** was observed. All attempts to separate **5f** from reaction mixture were unsuccessful. Compound **5f** was characterized by NMR spectroscopy.

^{1}H NMR (400 MHz, C_6D_6, 298 K) δ 1.00 (d, $^3J_{HH}$ = 6.8 Hz, 6H, (C***H₃***)₂CH), 1.07 (d, $^3J_{HH}$ = 6.8 Hz, 6H, $(C\boldsymbol{H_3})_2CH$), 1.08 (d, $^3J_{HH}$ = 6.8 Hz, 6H, $(C\boldsymbol{H_3})_2CH$), 1.11 (d, $^3J_{HH}$ = 6.8 Hz, 6H, $(C\boldsymbol{H_3})_2CH$), 1.13 (d, $^3J_{HH}$ = 6.8 Hz, 6H, $(C\boldsymbol{H_3})_2CH$), 1.15 (d, $^3J_{HH}$ = 6.8 Hz, 6H, $(C\boldsymbol{H_3})_2CH$), 1.30 (d, $^3J_{HH}$ = 6.8 Hz, 6H, $(C\boldsymbol{H_3})_2CH$), 1.36 (d, $^3J_{HH}$ = 6.7 Hz, 6H, $(C\boldsymbol{H_3})_2CH$), 1.48 (s, 6H, $\{(C\boldsymbol{H_3})_2C\}_2CH$), 1.50 (s, 6H, $\{(C\boldsymbol{H_3})_2C\}_2CH$), 2.98 (hept, $^3J_{HH}$ = 6.8 Hz, 2H, $(CH_3)_2C\boldsymbol{H}$), 2.98 (hept, $^3J_{HH}$ = 6.8 Hz, 2H, $(CH_3)_2C\boldsymbol{H}$), 3.32 (hept, $^3J_{HH}$ = 6.8 Hz, 2H, $(CH_3)_2C\boldsymbol{H}$), 3.67 (hept, $^3J_{HH}$ = 6.8 Hz, 2H, $(CH_3)_2C\boldsymbol{H}$), 4.69 (s, 2H, $C\boldsymbol{H_2}$), 4.90 (s, 1H, $\{(CH_3)C\}_2C\boldsymbol{H}$), 4.94 (s, 1H, $\{(CH_3)C\}_2C\boldsymbol{H}$), 6.93 (m, 2H, Ar-***H***), 7.05 (m, 2H, Ar-***H***), 7.09 (m, 2H, Ar-***H***), 7.11 (m, 2H, Ar-***H***), 7.16 (m, 1H, Ar-***H***), 7.19 (m, 1H, Ar-***H***), 7.21 (m, 1H, Ar-***H***), 7.23 (m, 1H, Ar-***H***).

^{13}C NMR (101 MHz, C_6D_6, 298 K) δ 23.3 (2x $\{(\boldsymbol{C}H_3)_2C\}_2CH$), 23.4 (2x $\{(\boldsymbol{C}H_3)_2C\}_2CH$), 24.6 (4x $(\boldsymbol{C}H_3)_2CH$), 24.7 (4x $(\boldsymbol{C}H_3)_2CH$), 24.9 (2x $(\boldsymbol{C}H_3)_2CH$), 25.0 (2x $(\boldsymbol{C}H_3)_2CH$), 25.2 (2x $(\boldsymbol{C}H_3)_2CH$), 25.6 (2x $(\boldsymbol{C}H_3)_2CH$), 28.2 (4x $(CH_3)_2\boldsymbol{C}H$), 28.6 (2x $(CH_3)_2\boldsymbol{C}H$), 29.1 (2x $(CH_3)_2\boldsymbol{C}H$), 67.5 ($C\boldsymbol{H_2}$), 97.7 ($\{(CH_3)_2C\}_2\boldsymbol{C}H$), 97.9 ($\{(CH_3)_2C\}_2\boldsymbol{C}H$), 124.1 (Ar***C***), 124.1 (Ar***C***), 124.8 (Ar***C***), 124.9 (Ar***C***), 126.9 (Ar***C***), 127.4 (Ar***C***), 139.3 (Ar***C***), 140.2 (Ar***C***), 143.2 (Ar***C***), 143.4 (Ar***C***), 144.9 (Ar***C***), 145.1 (Ar***C***), 155.9 ($\boldsymbol{C^2}$), 171.0 ($\{(CH_3)_2\boldsymbol{C}\}_2CH$), 171.1 ($\{(CH_3)_2\boldsymbol{C}\}_2CH$). Some Ar***C*** resonances are overlapping and cannot be observed. The Al–$\boldsymbol{C^3}$ resonance could not be observed in the ^{13}C NMR spectrum due to coupling to the quadrupolar ^{27}Al (I = 5/2) nucleus.

Preparation of ^{13}***C**$_3$**-5f***

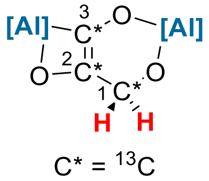

^{13}C labelled **^{13}C$_3$-3f** was prepared according to literature procedure.[2] In a glovebox, **^{13}C$_3$-3f** (18 mg) was dissolved in C_6D_6 (0.6 mL) and transferred to an NMR tube with a capillary of pyridine in C_6D_6 as an internal standard. The headspace of the NMR tube was evacuated *in vacuo*, and the NMR tube was removed from the glovebox. An atmosphere of H_2 (~ 1 atm) was introduced to the NMR tube. The NMR tube was heated at 100°C for 6 days. The reaction was monitored using ^{1}H NMR spectroscopy and deemed completed upon the total consumption of **^{13}C$_3$-3f** and formation of **^{13}C$_3$-5f**. Yield 39% (relative to internal pyridine standard).

^{1}H NMR (400 MHz, C_6D_6, 298 K) δ 1.00 (d, $^3J_{HH}$ = 6.8 Hz, 6H, (C$\boldsymbol{H_3}$)$_2$CH), 1.07 (d, $^3J_{HH}$ = 6.8 Hz, 6H, (C$\boldsymbol{H_3}$)$_2$CH), 1.08 (d, $^3J_{HH}$ = 6.8 Hz, 6H, (C$\boldsymbol{H_3}$)$_2$CH), 1.08 (d, $^3J_{HH}$ = 6.8 Hz, 6H, (C$\boldsymbol{H_3}$)$_2$CH), 1.10 (d, $^3J_{HH}$ = 6.8 Hz, 6H, (C$\boldsymbol{H_3}$)$_2$CH), 1.13 (d, $^3J_{HH}$ = 6.8 Hz, 6H, (C$\boldsymbol{H_3}$)$_2$CH), 1.15 (d, $^3J_{HH}$ = 6.8 Hz, 6H, (C$\boldsymbol{H_3}$)$_2$CH), 1.30 (d, $^3J_{HH}$ = 6.8 Hz, 6H, (C$\boldsymbol{H_3}$)$_2$CH), 1.48 (s, 6H, {(C$\boldsymbol{H_3}$)$_2$C}$_2$CH), 1.50 (s, 6H, {(C$\boldsymbol{H_3}$)$_2$C}$_2$CH), 2.98 (hept, $^3J_{HH}$ = 6.8 Hz, 2H, (CH$_3$)$_2$C***H***), 2.98 (hept, $^3J_{HH}$ = 6.8 Hz, 2H, (CH$_3$)$_2$C***H***), 3.32 (hept, $^3J_{HH}$ = 6.8 Hz, 2H, (CH$_3$)$_2$C***H***), 3.67 (hept, $^3J_{HH}$ = 6.8 Hz, 2H, (CH$_3$)$_2$C***H***), 4.70 (ddd, $^1J_{HC}$ = 140.7 Hz, $^2J_{HC}$ = 6.6 Hz, $^3J_{HC}$ 3.2 Hz, 2H, ^{13}C$\boldsymbol{H_2}$), 4.90 (s, 1H, {(CH$_3$)C}$_2$C***H***), 4.94 (s, 1H, {(CH$_3$)C}$_2$C***H***), 6.93 (m, 2H, Ar-***H***), 7.05 (m, 2H, Ar-***H***), 7.09 (m, 2H, Ar-***H***), 7.11 (m, 2H, Ar-***H***), 7.16 (m, 1H, Ar-***H***), 7.19 (m, 1H, Ar-***H***), 7.21 (m, 1H, Ar-***H***), 7.23 (m, 1H, Ar-***H***).

^{13}C NMR (101 MHz, C_6D_6, 298 K) δ 23.3 (2x {(***C***H$_3$)$_2$C}$_2$CH), 23.4 (2x {(***C***H$_3$)$_2$C}$_2$CH), 24.6 (4x (***C***H$_3$)$_2$CH), 24.7 (4x (***C***H$_3$)$_2$CH), 24.9 (2x (***C***H$_3$)$_2$CH), 25.0 (2x (***C***H$_3$)$_2$CH), 25.2 (2x (***C***H$_3$)$_2$CH), 25.6 (2x (***C***H$_3$)$_2$CH), 28.2 (4x (CH$_3$)$_2$***C***H), 28.6 (2x (CH$_3$)$_2$***C***H), 29.1 (2x (CH$_3$)$_2$***C***H), 67.48 (d, $^1J_{CC}$ = 48.6 Hz, ***^{13}C^1***H$_2$), 97.7 ({(CH$_3$)$_2$C}$_2$***C***H), 97.9 ({(CH$_3$)$_2$C}$_2$***C***H), 124.1 (Ar***C***), 124.1 (Ar***C***), 124.8 (Ar***C***), 124.9 (Ar***C***), 126.9 (Ar***C***), 127.4 (Ar***C***), 139.3 (Ar***C***), 140.2 (Ar***C***), 143.2 (Ar***C***), 143.4 (Ar***C***), 144.9 (Ar***C***), 145.1 (Ar***C***), 146.9 (d, $^1J_{CC}$ = 51.6 Hz, ***^{13}C^3***), 155.9 (dd, $^1J_{CC}$ = 53.1, 48.7 Hz, ***^{13}C^2***), 171.0 ({(CH$_3$)$_2$***C***}$_2$CH), 171.1 ({(CH$_3$)$_2$***C***}$_2$CH).

A proton coupled ^{13}C spectrum was also recorded (Figure S2) and key ^{13}C-labelled resonances were identified:

^{13}C NMR (101 MHz, C_6D_6, 298 K) δ 67.48 (td, $^{1}J_{CH}$ = 140.5, $^{1}J_{CC}$ = 48.7 Hz, $\boldsymbol{^{13}C^{1}}$H$_2$), 146.9 (d, $^{1}J_{CC}$ = 52.4 Hz, $\boldsymbol{^{13}C^{3}}$), 155.9 (ddt, $^{1}J_{CC}$ = 53.1, 48.6 Hz, $^{2}J_{CH}$ = 6.6 Hz, $\boldsymbol{^{13}C^{2}}$).

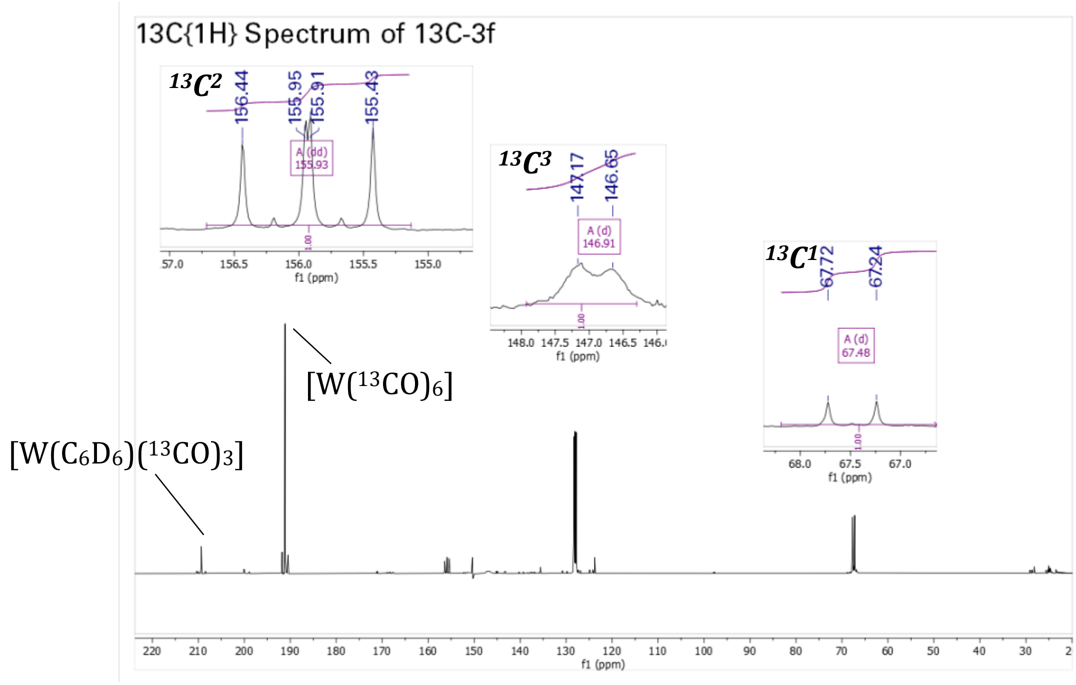

Figure S1: ^{13}C{^{1}H} spectrum of **5f-^{13}C** with key ^{13}C resonances of the carbon chain expanded. [W(^{13}CO)$_6$] and [W(C_6D_6)(^{13}CO)$_3$] are also observed in the spectrum.

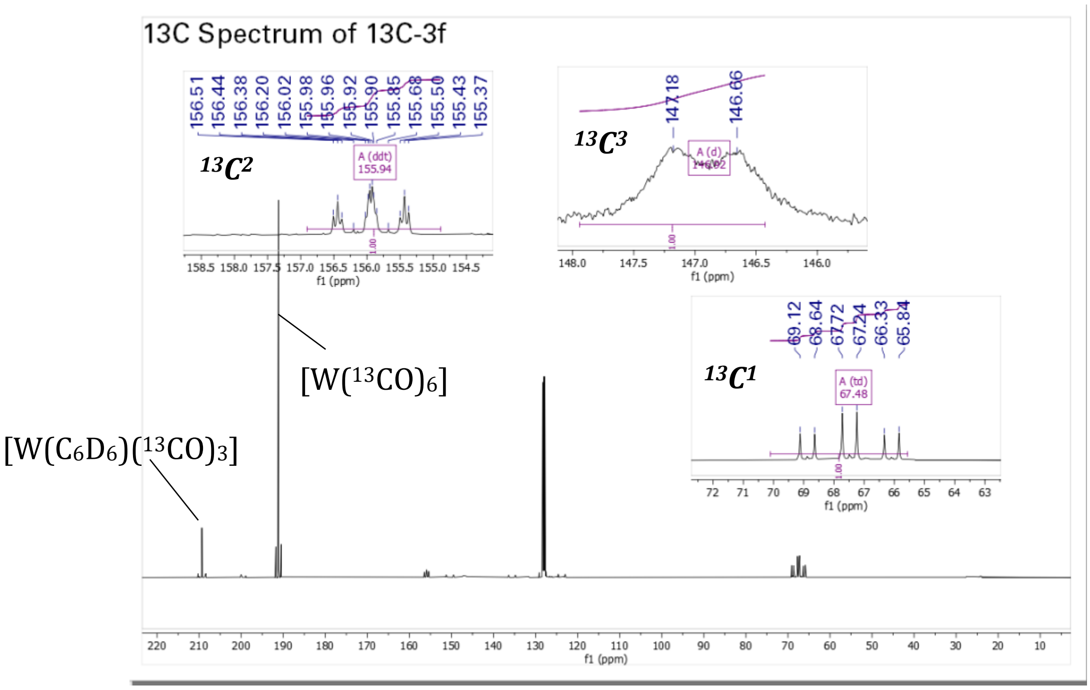

Figure S2: ^{13}C proton-coupled spectrum of **5f-^{13}C** with key ^{13}C resonances of the carbon chain expanded. [W(^{13}CO)$_6$] and [W(C_6D_6)(^{13}CO)$_3$] are also observed in the spectrum.

2.2 – Reversible conversion of **3a** to **4a**

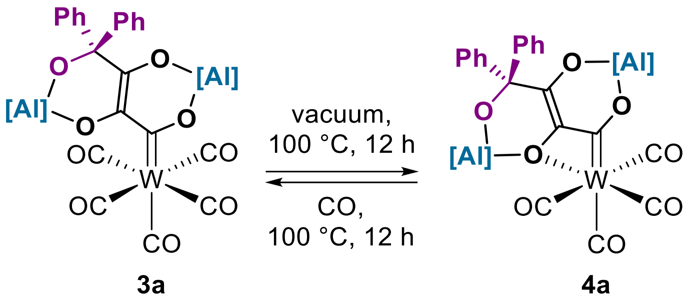

In a glovebox, **2** (15 mg, 0.012 mmol) and benzophenone (2.6 mg, 0.014 mmol) were dissolved in C_6D_6 (0.6 mL) and transferred to a J-Young NMR tube containing a capillary with ferrocene dissolved in C_6D_6 as external standard. The mixture was heated for 24 h at 100 °C. The reaction mixture was monitored by ^1H NMR spectroscopy and was deemed to be complete upon consumption of **2**. After this time, a ^1H NMR spectrum was recorded to determine the initial ratio between **3a** and **4a** (4:1). 81 % NMR yield.[2]

Conversion to 3a: The reaction mixture was frozen by placing it into a liquid-nitrogen bath (–196 °C), the headspace of the NMR tube was evacuated, and CO gas (~1 bar) was introduced into the NMR tube at 25 °C, the mixture was heated for 12h at 100 °C and a second ^1H NMR spectrum was recorded showing only resonances corresponding to **3a**.

Conversion back to a mixture of 3a/4a: The reaction mixture was frozen at –196 °C again, and CO removed under reduced pressure. The NMR tube was heated under static vacuum for 12h at 100 °C and a ^1H NMR spectrum was recorded showing the presence of **3a** and **4a** in a ratio ca. 1:1. This mixture was converted to **3a** again by introducing CO gas (~1 bar) at 25 °C into the NMR tube and heating for 12h at 100 °C. A Final ^1H NMR spectrum was recorder and signals corresponding to **4a** were not detected in the reaction mixture.

[2] The ratio of the formation of **3a** to **4a** under these reaction conditions is dependent on the concentration of the solution. The conversion of **3a** to **4a** involves the release of one CO molecule. The released CO molecule equilibrates between the headspace of the NMR tube (ca. 2mL) and solution. Hence, a more dilute sample will result in a larger relative proportion of **4a** in solution, whereas a more concentrated solution would result in a smaller relative proportion of **4a** in solution.

	Conditions	*Ratio 3a:4a*
t = 0	Initial reaction mixture	4:1
t = 1	100 °C for 12h under CO	99:1
t = 2	100 °C for 12h under vacuum	1:1
t = 3	100 °C for 12h under CO	99:1

Table S1: Ratio between **3a** and **4a** calculated by integration of ^{1}H NMR signals against an external standard of ferrocene.

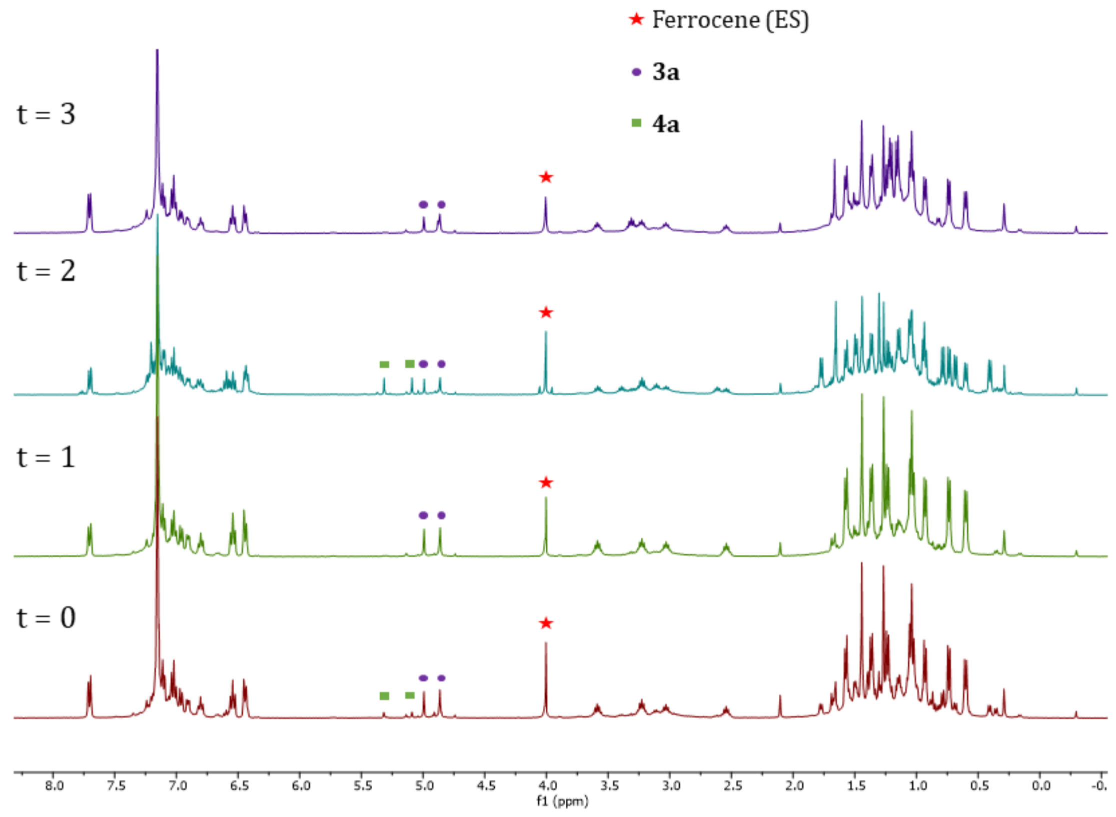

Figure S3: ^{1}H NMR experiments showing the reversible interconversion of **3** and **4**. Descriptions of conditions at each time detailed in Table S1.

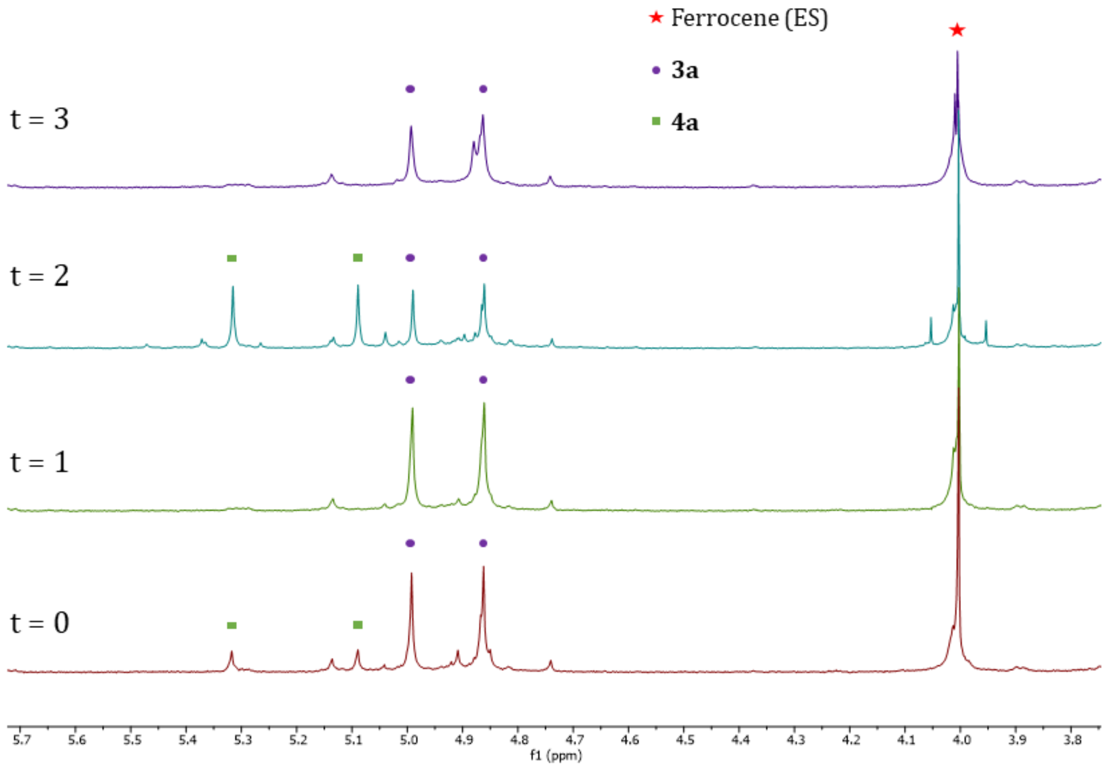

Figure S4: Detail of ^{1}H NMR experiments showing the reversible interconversion of **3a** and **4a**. Descriptions of conditions at each time detailed in Table S1.

2.3 – Direct reaction of **2**, H_2 and benzophenone

2 was prepared *in-situ* in an NMR tube from **1** (8.9 mg, 0.02 mmol), [W(CO)$_6$] (4 mg, 0.011 mmol) and CO gas (~1 bar) following the reported procedure.[2] Once formation of **2** was confirmed by ^{1}H NMR spectroscopy, the NMR tube was returned to the glovebox, the reaction mixture was cooled to –35 °C using a low temperature reactor and the headspace of the NMR tube was evacuated to remove the remaining CO gas. A capillary containing a solution of 1,3,5-trimethoxybenzene in C_6D_6 as external standard and a solution of Ph_2CO (1.82 mg, 0.01 mmol) in C_6D_6 (0.3 mL) were added to the NMR tube and the mixture cooled to –35 °C. The headspace of the NMR tube was evacuated, the NMR tube was removed from the glovebox and H_2 gas (~1 bar) was introduced into the headspace of the NMR. The resultant mixture was heated at 100 °C and conversion to **5a** was complete after 24 h, as monitored by ^{1}H NMR spectroscopy. NMR yield: 42%.

2.4 – Direct reaction of [W(CO$_6$)], **1** and syngas (1:1 H_2 : CO)

In a glovebox, an NMR tube was charged with a suspension of **1** (8.9 mg, 0.02 mmol) in C_6D_6 (0.3 mL) was cooled to –35 °C using a low temperature reactor. [W(CO)$_6$] (4 mg, 0.011 mmol)) was added slowly as a slurry in C_6D_6 (0.3 mL) *via* Pasteur pipette. Care was taken to ensure that the reaction mixture remains frozen and a capillary containing a solution of 1,3,5-trimethoxybenzene in C_6D_6 as external standard was introduced. The headspace of the NMR tube was evacuated, and the NMR tube was removed from the glovebox quickly and placed into a liquid-nitrogen bath (–196 °C). The tube was removed from the liquid nitrogen bath, and syngas (1:1 mixture of H_2/CO, ~1 bar) was introduced at room temperature into the headspace of the NMR tube while the mixture was still frozen. Upon addition of syngas, the mixture was allowed to thaw, and during this process the tube was shaken vigorously to ensure incorporation of gas into solution. A ^{1}H NMR spectrum was taken at this point to show formation of **2**. Reaction mixture was heated at 100 °C and monitored by ^{1}H NMR spectroscopy until conversion to **5b** was completed after 10 days. NMR yield: 50%.[3]

During the progress of the reaction only signals corresponding to **2**, **3b** and **5b** were observed. **5f** was not detected at any time.

[3] Calculated against 1,3,5-trimethoxybenzene external standard in relation to initial concentration of **2**.

2.5 – Kinetic experiments

In a glovebox, **4a** (8 mg, 0.0055 mmol) and ferrocene (0.2 mg, 0.0011 mmol) as internal standard were placed in a vial and dissolved in 1.2 mL of C_6D_6. Then, a portion 0.55 mL was transferred to a J-Young NMR tube. The headspace of the NMR tube was evacuated and H_2 (or D_2) gas (~1 bar) was introduced. The tube was transferred to an NMR spectrometer preheated at 80 °C and allowed to warm to 100 °C. The reaction mixture was monitored as a function of time over 8000 sec (133 min) with data points acquired every 68 sec. The concentration of **5a** was constant after 6000 sec (100 min) in both cases.

A plot of Ln[**4a**] (determined from initial concentration and integration against internal standard) vs time for both reactions using H_2 or D_2 gave a linear fit with high R-factor (Figure S5) indicating the reaction is first order in [**4a**]. Standard errors were calculated by use of the regression analysis calculation in Microsoft Excel software. The rate constant for the H_2 reaction was found to be $k_{obs}(H_2)$ = 6.28 x 10^{-4} (± 6 x 10^{-6}) s^{-1} and $k_{obs}(D_2)$ = 6.16 x 10^{-4} (± 6 x 10^{-6}) s^{-1} for D_2. This gave a $k_{obs}(H_2)$ / $k_{obs}(D_2)$ of 1.02 (±0.01) for the reaction.

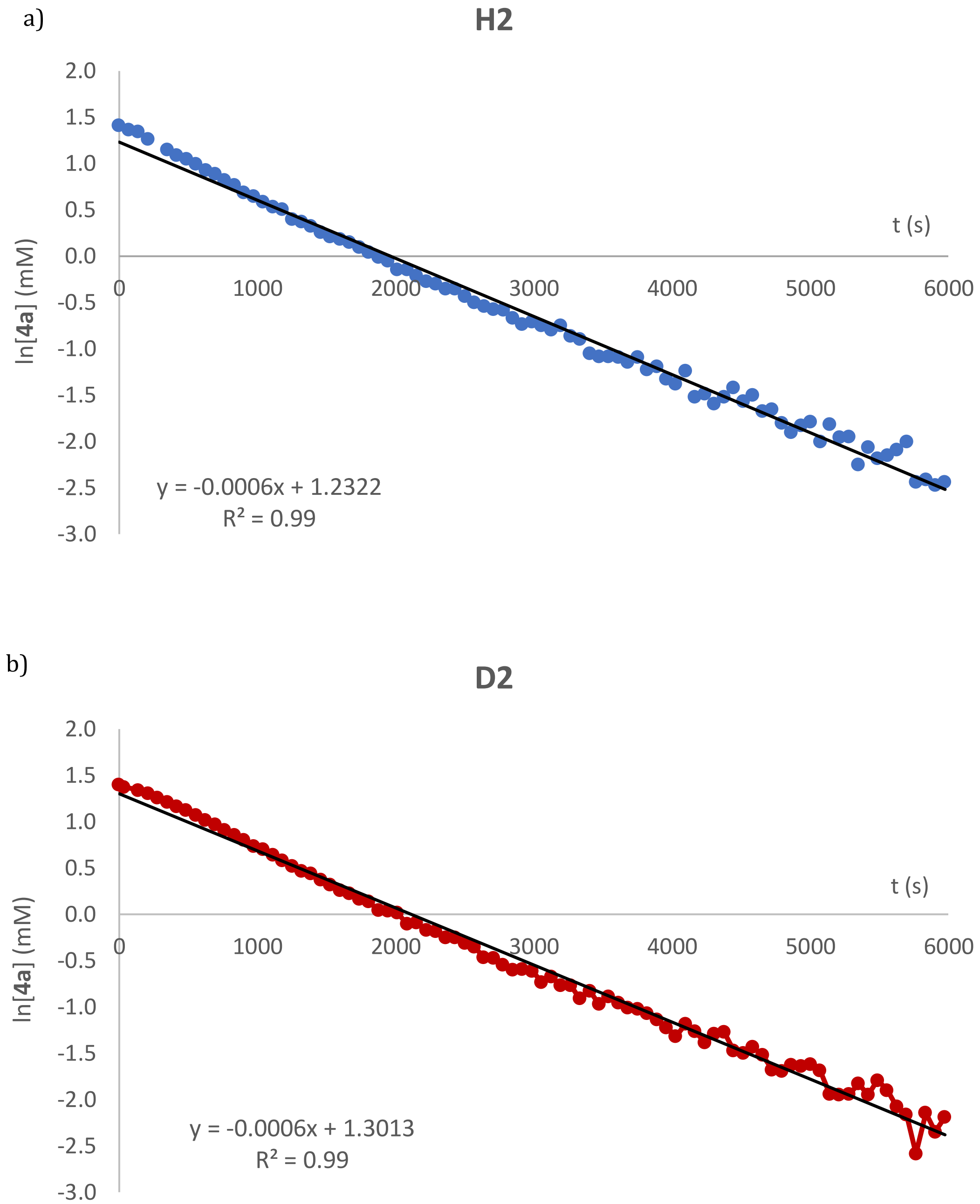

Figure S5: Ln[**4a**] versus time plot for reaction of **4a** with H_2 (a) and D_2 (b).

2.6 – Monitoring of reaction of **3a** with H_2

In a glovebox, **3a** (8 mg, 0.0055 mmol) and ferrocene (0.4 mg, 0.0021 mmol) as internal standard were placed in a vial and dissolved in 1.2 mL of C_6D_6. Then, a portion 0.45 mL was transferred to a J-Young NMR tube. The headspace of the NMR tube was evacuated and H_2 (or D_2) gas (~1 bar) was introduced. The tube was transferred to an NMR spectrometer preheated at 100 °C. The reaction mixture was monitored as a function of time over 8400 sec (140 min) with data points acquired every 68 sec. First spectrum was taken after 300 sec (5 min) and signals corresponding to **4a** and **5a** were detected. Complex **3a** was consumed after 5000 sec (83 min) and concentration of **5a** was constant after 8000 sec (133 min).

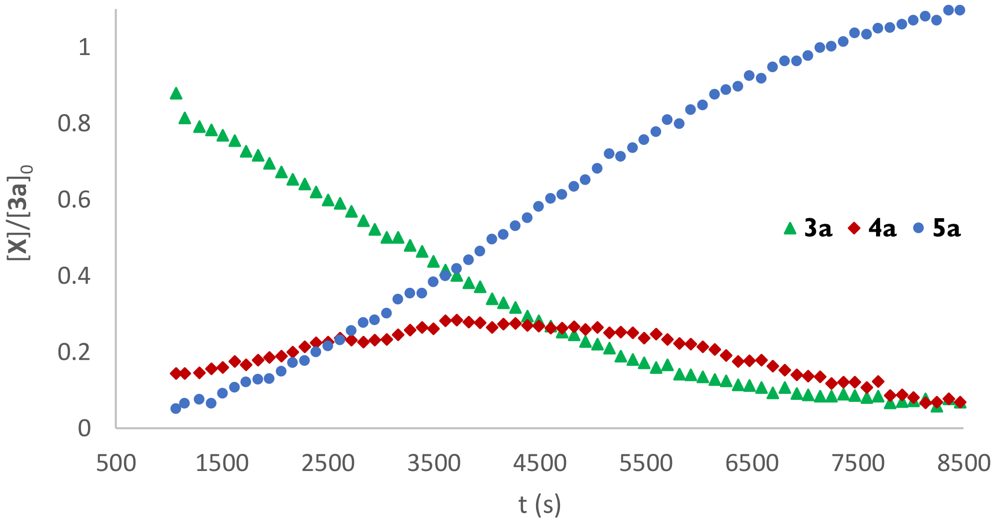

Figure S6: Plot of variation of concentration with time of compounds **3a**, **4a** and **5a** for hydrogenation of **3a**.

3 X-RAY DATA

The X-ray structure of **3a**

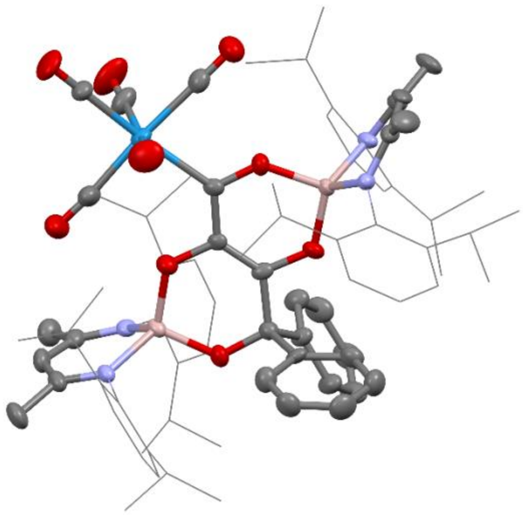

Figure S7: The X-ray structure of **3a**. All hydrogen atoms are omitted for clarity.

3a was found to crystallise in the P-1 space group with an included toluene and pentane molecule in the asymmetric unit.

The isopropyl group C33>C35 was found to be disordered over two sites in a ca. 58:42 ratio for the major and minor components respectively. The thermal parameters of both orietnations were restrained to be similar, and only the non-hydrogen atoms of the major component were refined anisotropically (those in the minor component were refined isotropically).

The included toluene molecule (C81>C87) was found to be disordered. No convincing model of a second orientation of a toluene molecule could be found and the residual electron density most resembled a pentane molecule. As a result, the toluene molecule was modelled as the major component of the disordered fragment with ca. 74% occupancy, while the pentane molecule (C91>C95) was modelled as the minor component of the disordered fragment with ca. 26% occupancy. The geometries of both major and minor components were optimized, their thermal parameters restrained to be similar and only the non-hydrogen atoms of the major component were refined anisotropically (those in the minor component were refined isotropically).

Crystal Data for $C_{85.48}H_{101.04}Al_2N_4O_9W$, M =1566.31, triclinic, space group P-1 (no. 2), a = 13.1875(3) Å, b = 16.3826(5) Å, c = 18.6982(5) Å, α = 99.300(2)°, β = 99.5985(19)°, γ = 92.952(2)°, V = 3917.95(17) Å^3, Z = 2, ρ_{calc}g/cm^3 = 1.328, μ(MoKα) = 1.556 mm^{-1}, T = 173.05(10), yellow needles, F^2 refinement,[5,6] R_1(obs) = 0.0429, wR_2(all) = 0.1025, 15462 independent observed reflections (R_{int} = 0.0286), 12739 independent measured reflections [$|F_o| > 4\sigma(|F_o|)$, $2\theta_{full}$ = 56.498], 981 parameters. CCDC 2130349.

The X-ray structure of **4a**

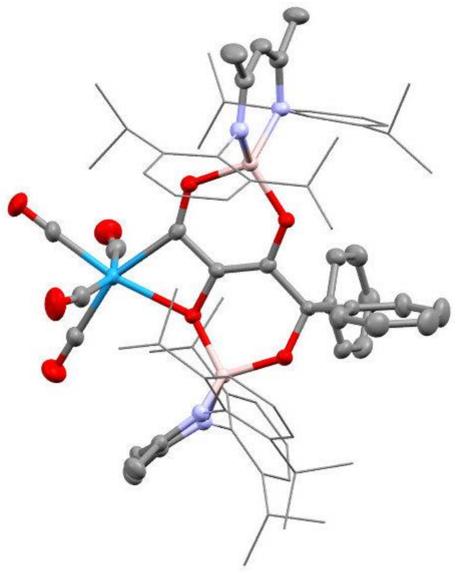

Figure S8: The X-ray structure of **4a**. All hydrogen atoms are omitted for clarity.

4a was found to crystallise in the $P2_1/n$ space group with two included hexane molecules with respective occupancies of 0.25 and 0.5 for a total of 0.75 molecules in the asymmetric unit, for a total of 3 within the unit cell.

A significant amount of electron density (ca $4e^-$) was observed approximately 1 Å from W1. This unresolved electron density was assigned to be a minor component of co-crystallised material. Accordingly, the tungsten centre was split over these two sites resulting in a ca 96:4 occupancy for the major and minor components respectively. The major component corresponds to the structure of **4a**, while the minor component appears to be trace amounts of **3a**, based on inspection of the W1'–C1–C2 angle and W1'–O2 distance. No other electron density associated with **3a** was observed in the Fourier difference map. This is not unexpected for two reasons: i) it is apparent from the geometry of the C1–W1 and C1–W1' orientations that it is likely that the ligands at the tungsten centres (both CO and the C1 to C4 carbon chain) overlap to a significant degree ii) the comparatively small percentage of the minor component (4%) preclude observation of the C and O carbonyl atoms bound to the W1' centre.

The isopropyl group C32>C34 was found to be disordered over two sites in a ca. 54:46 ratio for the major and minor components respectively. The thermal parameters of both

orietnations were restrained to be similar, and only the non-hydrogen atoms of the major component were refined anisotropically (those in the minor component were refined isotropically).

The arene ring C38>C43 was found to be disordered over two sites in a ca. 62:38 for the major and minor components respectively. The geometries of both were optimised using AFIX 66, the thermal parameters were restrained to be similar, and only the non-hydrogen atoms of the major component were refined anisotropically (those in the minor component were refined isotropically).

The isopropyl group C44>C46 was found to be disordered over two sites in a ca. 58:42 ratio for the major and minor components respectively. Their geometries were optimized, the thermal parameters were restrained to be similar, and only the non-hydrogen atoms of the major component were refined anisotropically (those in the minor component were refined isotropically).

The hexane molecule C79>C85 was found to be a 0.5 occupancy molecule by inspection of the thermal ellipsoids.

The hexane molecule C86>C92 was found to be disordered about a special position. The molecule was modelled in the Part -1. The occupancy of the molecule was set at 0.25 by inspection of the thermal ellipsoids.

Crystal Data for $C_{83.25}H_{104}Al_2N_4O_8W$, M =1526.51, monoclinic, space group $P2_1/n$ (no. 14), a = 13.5044(3) Å, b = 42.6353(10) Å, c = 14.7867(5) Å, β = 103.943(3)°, V = 8262.8(4) Å^3, Z = 4, ρ_{calc}g/cm^3 = 1.227, μ(CuKα) = 3.215 mm^{-1}, T = 173.00(10), red needles, F^2 refinement,[5,6] R_1(obs) = 0.0513, wR_2(all) = 0.1423, 15844 independent observed reflections (R_{int} = 0.0440), 12208 independent measured reflections [$|F_o| > 4\sigma(|F_o|)$, $2\theta_{full}$ = 147.192], 989 parameters. CCDC 2130349.

The X-ray structure of **S1**

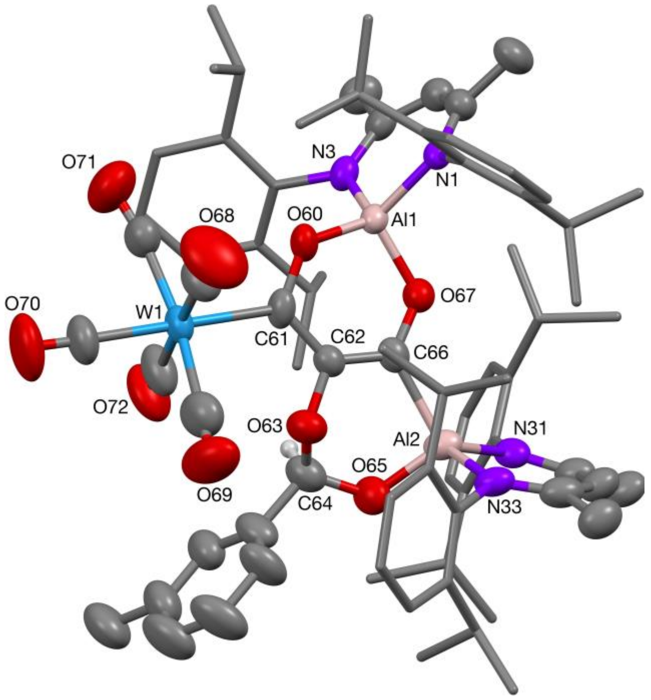

Figure S9: The X-ray structure of **S1**. All hydrogen atoms are omitted for clarity.

Difference electron density maps for the structure of **S1** suggested the presence of a second orientation of the whole molecule (of *ca.* 13% occupancy) overlaying the main occupancy orientation in a manner corresponding to a *ca.* 180° rotation about an axis passing through the middle of the complex (along the *b* axis direction, approximately coincident with the C66–C62 bond). Unsurprisingly, the only atom of this second orientation that could be reliably located was the minor occupancy tungsten atom, W1', which was refined anisotropically. The C64-bound *m*-tolyl group and the O80-based included tetrahydrofuran solvent molecule were both found to be disordered. For the former, two orientations were identified of *ca*. 67 and 33% occupancy, whilst for the latter three orientations were identified of *ca*. 52, 30 and 18% occupancy. The geometries of each set of orientations were optimised, the thermal parameters of adjacent atoms were restrained to be similar, and only the non-hydrogen atoms of the major occupancy orientations were refined anisotropically (those of the minor occupancy orientations were refined isotropically).

Crystal data for **S1**: $C_{74}H_{90}Al_2N_4O_9W{\cdot}C_4H_8O$, M = 1489.41, monoclinic, $P2_1/c$ (no. 14), a = 14.9640(6), b = 21.4758(5), c = 24.2362(8) Å, β = 107.909(4)°, V = 7411.2(4) Å^3, Z =

4, D_c = 1.335 g cm^{-3}, μ(Cu-Kα) = 3.592 mm^{-1}, T = 173 K, orange tablets, Agilent Xcalibur PX Ultra A diffractometer; 14495 independent measured reflections (R_{int} = 0.0404), F^2 refinement,[5,6] R_1(obs) = 0.0647, wR_2(all) = 0.1700, 11508 independent observed absorption-corrected reflections [$|F_o| > 4\sigma(|F_o|)$, completeness to θ_{full}(67.7°) = 99.8%], 935 parameters. CCDC 2129492.

The X-ray structure of **3c**

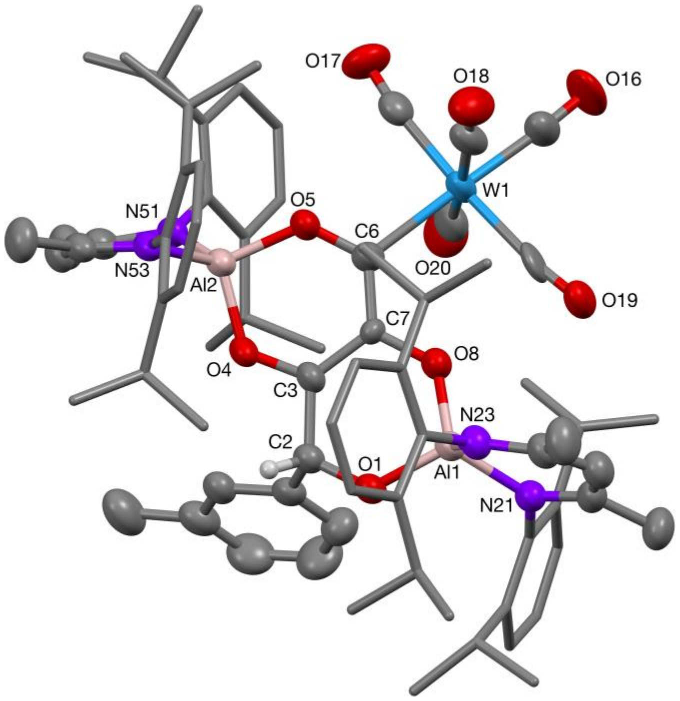

Figure S10: The X-ray structure of **3c**. All hydrogen atoms are omitted for clarity.

The W1-based $W(CO)_5$ unit in the structure of **5c** was found to be disordered. Two orientations were identified of *ca*. 70 and 30% occupancy, their geometries were optimised, and the thermal parameters of adjacent atoms were restrained to be similar. All of the atoms of the major occupancy orientation, and the tungsten centre of the minor occupancy orientation, were refined anisotropically (the oxygen and carbon atoms of the minor occupancy orientation were refined isotropically).

The included solvent was found to be highly disordered, and the best approach to handling this diffuse electron density was found to be the SQUEEZE routine of PLATON.[7] This suggested a total of 231 electrons per unit cell, equivalent to 28.9 electrons per asymmetric unit. Before the use of SQUEEZE the solvent most resembled pentane (C_5H_{12}, 42 electrons), and 0.75 pentane molecules corresponds to 31.5 electrons, so this was used as the solvent present. As a result, the atom list for the asymmetric unit is low by $0.75(C_5H_{12}) = C_{3.75}H_9$ (and that for the unit cell low by $C_{30}H_{72}$) compared to what is actually presumed to be present.

Crystal data for **3c**: $C_{74}H_{90}Al_2N_4O_9W·0.75(C_5H_{12})$, M = 1471.41, monoclinic, $I2_1/a$ (no. 15), a = 30.6403(15), b = 21.7168(5), c = 23.4001(12) Å, β = 98.775(6)°, V = 15388.4(12) Å^3, Z = 8, D_c = 1.270 g cm^{-3}, μ(Cu-Kα) = 3.443 mm^{-1}, T = 173 K, orange plates, Agilent Xcalibur PX Ultra A diffractometer; 14796 independent measured reflections (R_{int} = 0.0484), F^2 refinement,[5,6] R_1(obs) = 0.0503, wR_2(all) = 0.1461, 8502 independent observed absorption-corrected reflections [$|F_o| > 4\sigma(|F_o|)$, completeness to θ_{full}(67.7°) = 98.9%], 882 parameters. CCDC 2129490.

The X-ray structure of **3d**

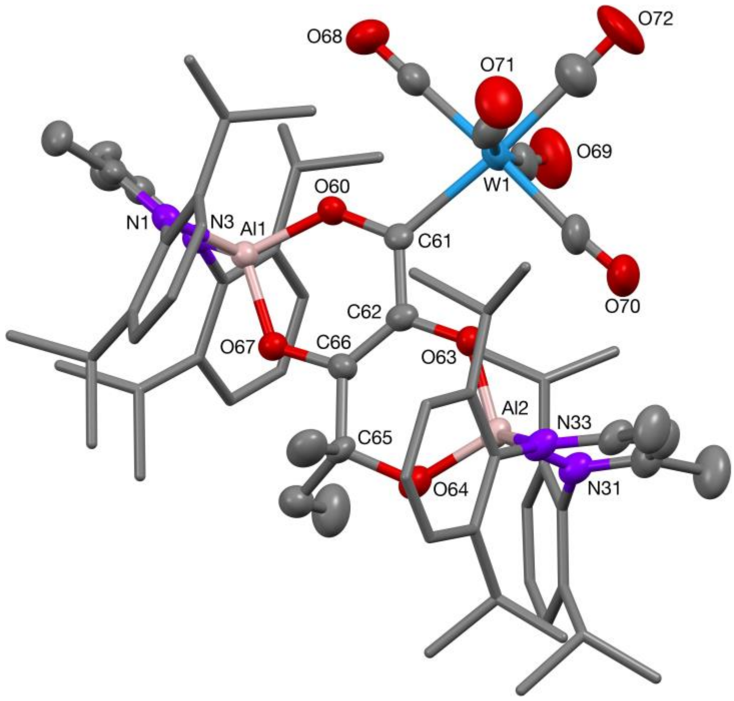

Figure S11: The X-ray structure of **3d**. All hydrogen atoms are omitted for clarity.

The crystal of **3d** that was studied was found to be a two component twin in a *ca*. 56:44 ratio, with the two lattices related by the approximate twin law [1.00 0.00 0.00 0.00 –1.00 0.00 –0.09 0.00 –1.00]. The C65-based C(Et)(Me) unit was found to be disordered. Two orientations were identified of *ca*. 56 and 44% occupancy, their geometries were optimised, the thermal parameters of adjacent atoms were restrained to be similar, and only the non-hydrogen atoms of the major occupancy orientation were refined anisotropically (those of the minor occupancy orientation were refined isotropically). The included solvent was found to be highly disordered, and the best approach to handling this diffuse electron density was found to be the SQUEEZE routine of PLATON. This suggested a total of 159 electrons per unit cell, equivalent to 39.8 electrons per asymmetric unit. Before the use of SQUEEZE the solvent most resembled pentane (C_5H_{12}, 42 electrons), and one pentane molecule corresponds to 42 electrons, so this was used as the solvent present. As a result, the atom list for the asymmetric unit is low by C_5H_{12} (and that for the unit cell low by $C_{20}H_{48}$) compared to what is actually presumed to be present.

Crystal data for **S11**: $C_{70}H_{90}Al_2N_4O_9W{\cdot}C_5H_{12}$, M = 1441.41, monoclinic, $P2_1/n$ (no. 14), a = 20.4694(5), b = 17.5544(4), c = 20.6800(4) Å, β = 92.606(2)°, V = 7423.2(3) Å^3, Z = 4, D_c = 1.290 g cm^{-3}, μ(Cu-Kα) = 3.555 mm^{-1}, T = 173 K, yellow blocks, Agilent Xcalibur PX Ultra A diffractometer; 20698 independent measured reflections (R_{int} = 0.0772), F^2 refinement,[5,6] R_1(obs) = 0.0593, wR_2(all) = 0.1749, 15250 independent observed absorption-corrected reflections [$|F_o| > 4\sigma(|F_o|)$, completeness to θ_{full}(67.7°) = 98.3%], 813 parameters. CCDC 2129491.

The X-ray structure of **S2**

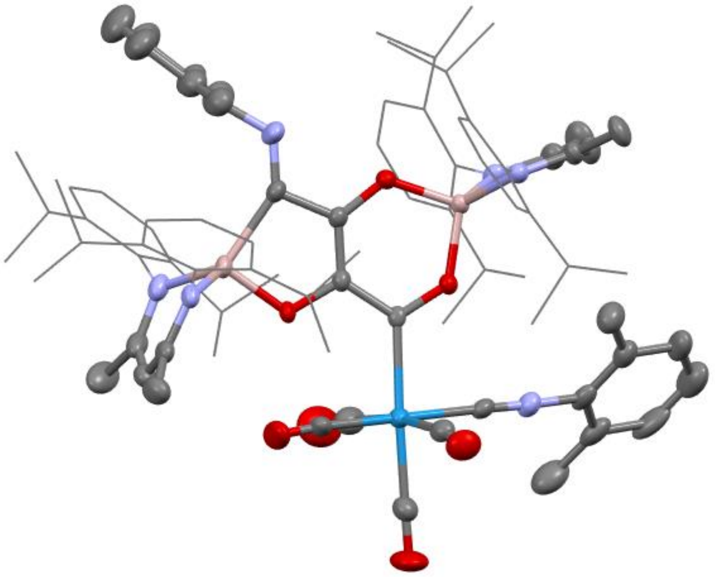

Figure S12: The X-ray structure of **S2**. All hydrogen atoms are omitted for clarity.

S2 was found to crystallise in the P-1 space group with one included pentane molecule and half of a toluene molecule for a total of two pentane molecules and one toluene molecule within the unit cell.

The included toluene molecule (C84>C90) was found to be disordered across two positions. Inspections of the thermal parameters of the ellipsoids suggest that the molecule has a total of one half occupancy across the two orientations. Their geometries were optimized, the thermal parameters were restrained to be similar, and only the non-hydrogen atoms of the major component were refined isotropically.

The included solvent was found to be highly disordered, and the best approach to handling this diffuse electron density was found to be the SQUEEZE routine of PLATON. This suggested a total of 144 electrons per unit cell, equivalent to 72 electrons per asymmetric unit. Before the use of SQUEEZE the solvent most resembled two pentane molecules (C_5H_{12}, 84 electrons), and two pentane molecule corresponds to 84 electrons, so this was used as the solvent present. As a result, the atom list for the asymmetric unit is low by $C_{10}H_{24}$ (and that for the unit cell low by $C_{20}H_{48}$) compared to what is actually presumed to be present.

Crystal Data for $C_{86.5}H_{104}Al_2N_6O_7W$, M =1577.56, triclinic, space group P-1 (no. 2), a = 13.3538(2) Å, b = 13.8568(4) Å, c = 26.7693(6) Å, α = 75.930(2)°, β = 77.1228(16)°, γ = 83.7504(18)°, V = 4676.11(19) Å^3, Z = 2, ρ_{calc}g/cm^3 = 1.120, μ(Mo Kα) = 1.303 mm^{-1}, T = 172.95(10), violet blocks, F^2 refinement, R_1(obs) = 0.0435, wR_2(all) = 0.1110, 18515 independent observed reflections (R_{int} = 0.0261), 15316 independent measured reflections [$|F_o| > 4\sigma(|F_o|)$, $2\theta_{full}$ = 56.594], 987 parameters. CCDC 2130351.

4 DENSITY FUNCTIONAL THEORY CALCULATIONS

4.1 – Computational methods

DFT calculations were performed using Gaussian 09 (Revision D.01) using an ultrafine integration grid (int=ultrafine).[8] Geometry optimisations and frequency calculations were performed using the ωB97X functional[9] with SDDAll (W, Al) and 6-31G** (C, H, N, O) basis set. Frequency analyses for all stationary points were performed using the enhanced criteria to confirm the nature of the structures as either minima (no imaginary frequency) or transition states (only one imaginary frequency). The electronic energies of the optimised geometries were calculated using the ωB97XD functional with def2tzvp (W, Al) and 6-311+G** (C, H, N, O) basis sets with solvent corrections (PCM, benzene, ε = 2.2706). The Gibbs free energy correction from the frequency calculation was added to this electronic energy to generate Gibbs free energy values for the calculated stationary points.

Intrinsic reaction coordinate (IRC) calculations were used to connect transition states and minima located on the potential energy surface allowing a full energy profile (calculated at 298.15 K, 1 atm) of the reaction to be constructed.[10] Natural Bond Orbital analysis was carried out using NBO 6.0 with the ωB97x functional.[11]

Functional testing was performed with the B3LYP,[12] and B3PW91[13] in addition to the M06L[14,15] and ωB97X functionals with SDDAll (W, Al) and 6-31G** (C, H, N, O) basis set. The electronic energies of the optimised geometries were calculated using the corresponding functional with the same basis set with solvent corrections (PCM, benzene, ε = 2.2706) and an empirical dispersion correction (Grimme, D3: B3LYP, B3PW91, M06L; Grimme D2: ωB97X).

4.2 – Calculated stationary points

4.2.1 – Transformation of **3a** to **4a** (Interchange mechanism).

Calculated stationary points for transformation of **3a** to **4a**.

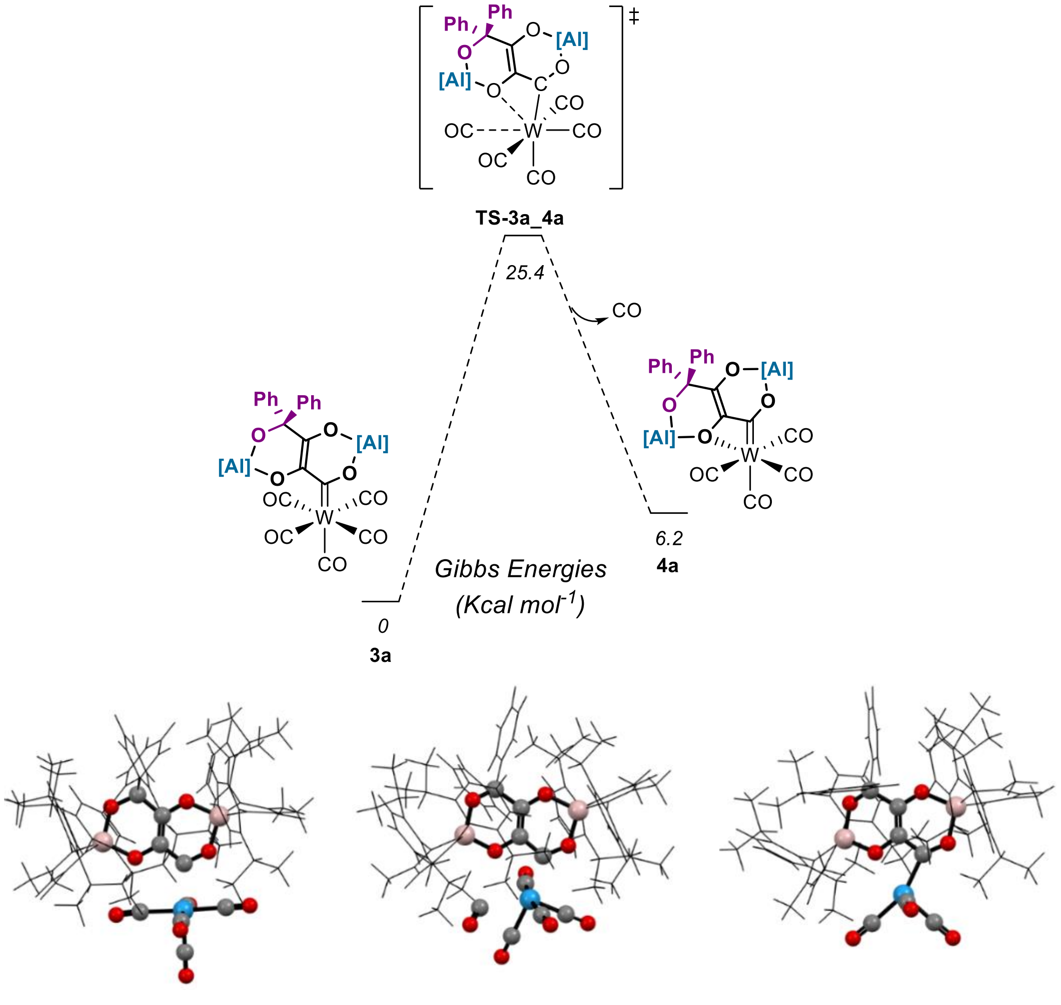

3a **TS-3a_4a** **4a**

Figure S13: Calculated pathway for transformation of **3a** to **4a**. All energies in kcal mol^{-1}.

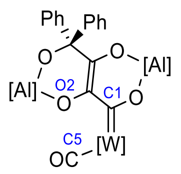

		3a	***TS-3a_4a***	***4a***
WBI	W-C1	0.51	0.56	0.61
	W-C5	0.78	0.14	-
	W-O2	0.003	0.05	0.17
NPA	W	-0.86	-0.51	-0.43
	C1	0.31	0.28	0.26
	O2	-1.00	-1.00	-0.96
Bond distance (Å)	W-C1	2.270	2.197	2.167
	W-C5	2.067	2.876	-
	W-O2	3.463	2.903	2.423

Table S2: Selected calculated NBO data of **3a**, **TS-3a_4a**, and **4a**.

4.2.2 - Calculated mechanism for hydrogenation of **4a**.

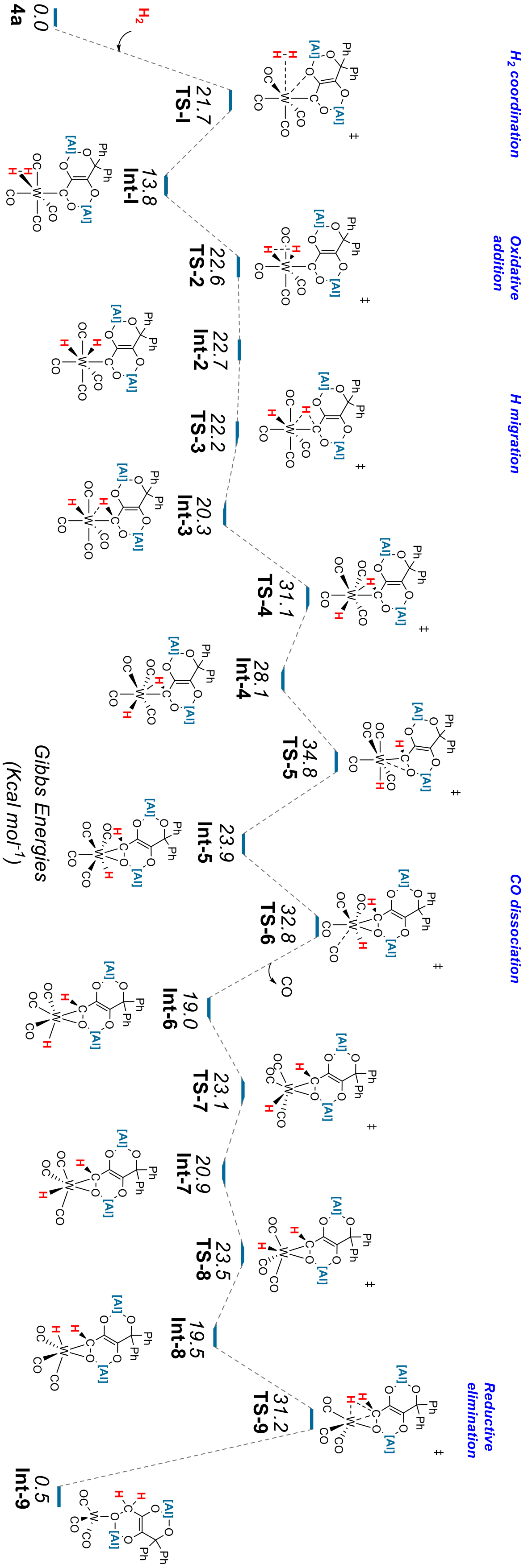

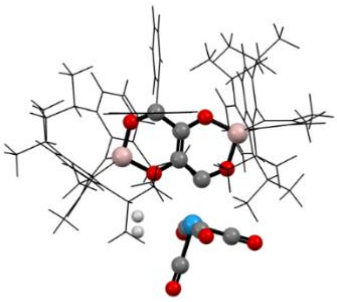

TS-1

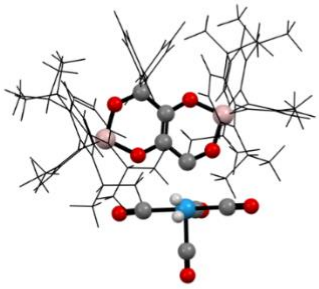

Int-1

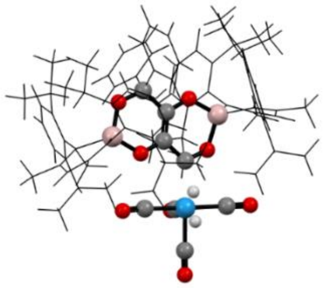

TS-2

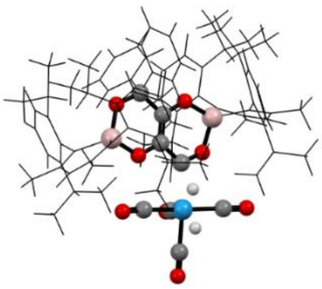

Int-2

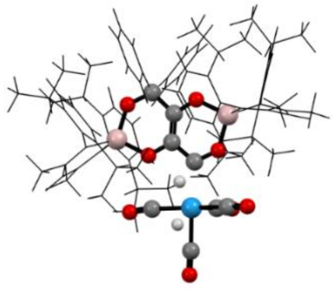

TS-3

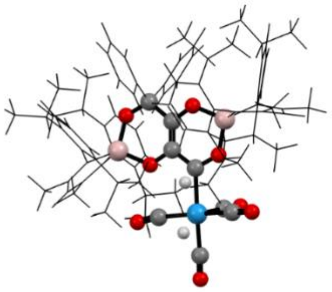

Int-3

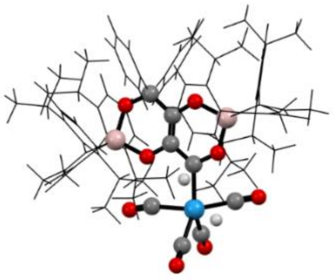

TS-4

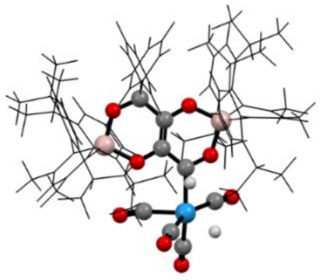

Int-4

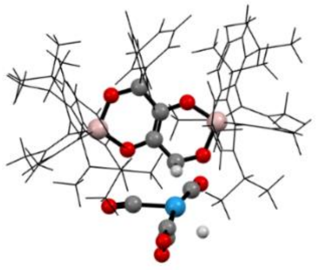

TS-5

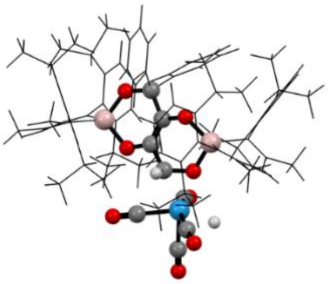

Int-5

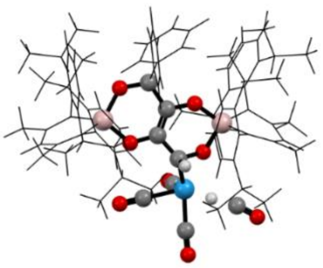

TS-6

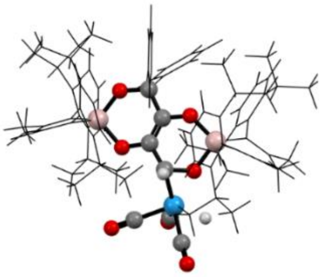

Int-6

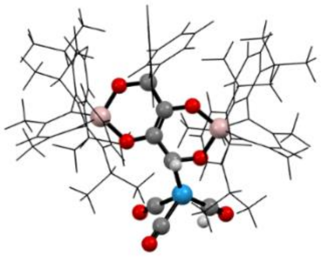

TS-7

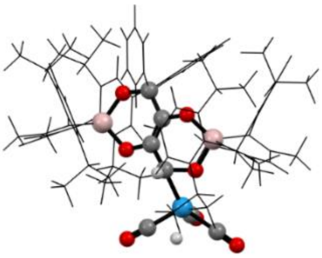

Int-7

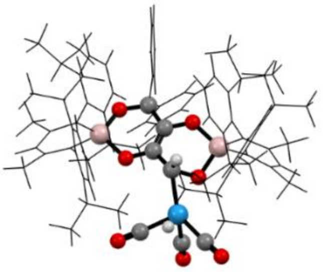

TS-8

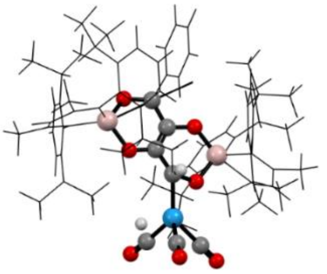

Int-8

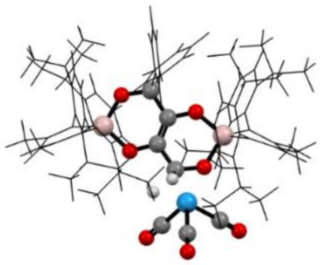

TS-9

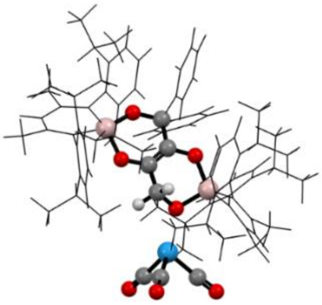

Int-9

Figure S14: Calculated pathway for hydrogenation of **4a**. All energies in kcal mol^{-1}.

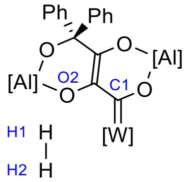

		4a	TS-1	Int-1	TS-2	Int-2	TS-3	Int-3
WBI	W-C1	0.61	0.54	0.53	0.49	0.51	0.60	0.70
	W-H1	-	0.03	0.19	0.49	0.54	0.45	0.21
	W-H2	-	0.03	0.18	0.48	0.53	0.54	0.54
	H1-H2	-	0.93	0.69	0.24	0.13	0.10	0.04
	C1-H1	-	0.00	0.03	0.09	0.11	0.25	0.59
	C1-H2	-	0.00	0.00	0.02	0.04	0.03	0.04
NPA	W	-0.43	-0.47	-0.89	-0.93	-0.81	-0.77	-0.61
	C1	0.26	0.27	0.31	0.35	0.36	0.32	0.14
	H1	-	0.01	0.07	0.02	-0.05	0.03	0.18
	H2	-	0.03	0.10	0.05	-0.01	-0.09	-0.17

		Int-5	TS-6	Int-6	Int-8	TS-9	Int-9
WBI	W-C1	0.58	0.56	0.54	0.53	0.48	0.02
	W-H1	0.58	0.01	0.01	0.01	0.00	0.00
	W-H2	0.53	0.54	0.55	0.57	0.37	0.00
	H1-H2	0.00	0.00	0.00	0.00	0.00	0.00
	C1-H1	0.89	0.89	0.89	0.89	0.88	0.90
	C1-H2	0.07	0.09	0.08	0.08	0.34	0.90
NPA	W	-0.34	0.08	0.16	0.13	-0.31	0.10
	C1	-0.08	-0.23	-0.25	-0.25	-0.03	-0.14
	H1	0.22	0.22	0.22	0.22	0.24	0.23
	H2	-0.15	-0.11	-0.09	-0.01	0.09	0.25

Table S3: Selected calculated NBO data of calculated mechanisms for hydrogenation of **4a**.

To find the most plausible mechanism, a range of different pathways were investigated. All alternative pathways found for hydrogenation of **4a** are shown below.

Alternative calculated mechanism (*a*) for hydrogenation of **4a**: CO dissociation prior to oxidative addition.

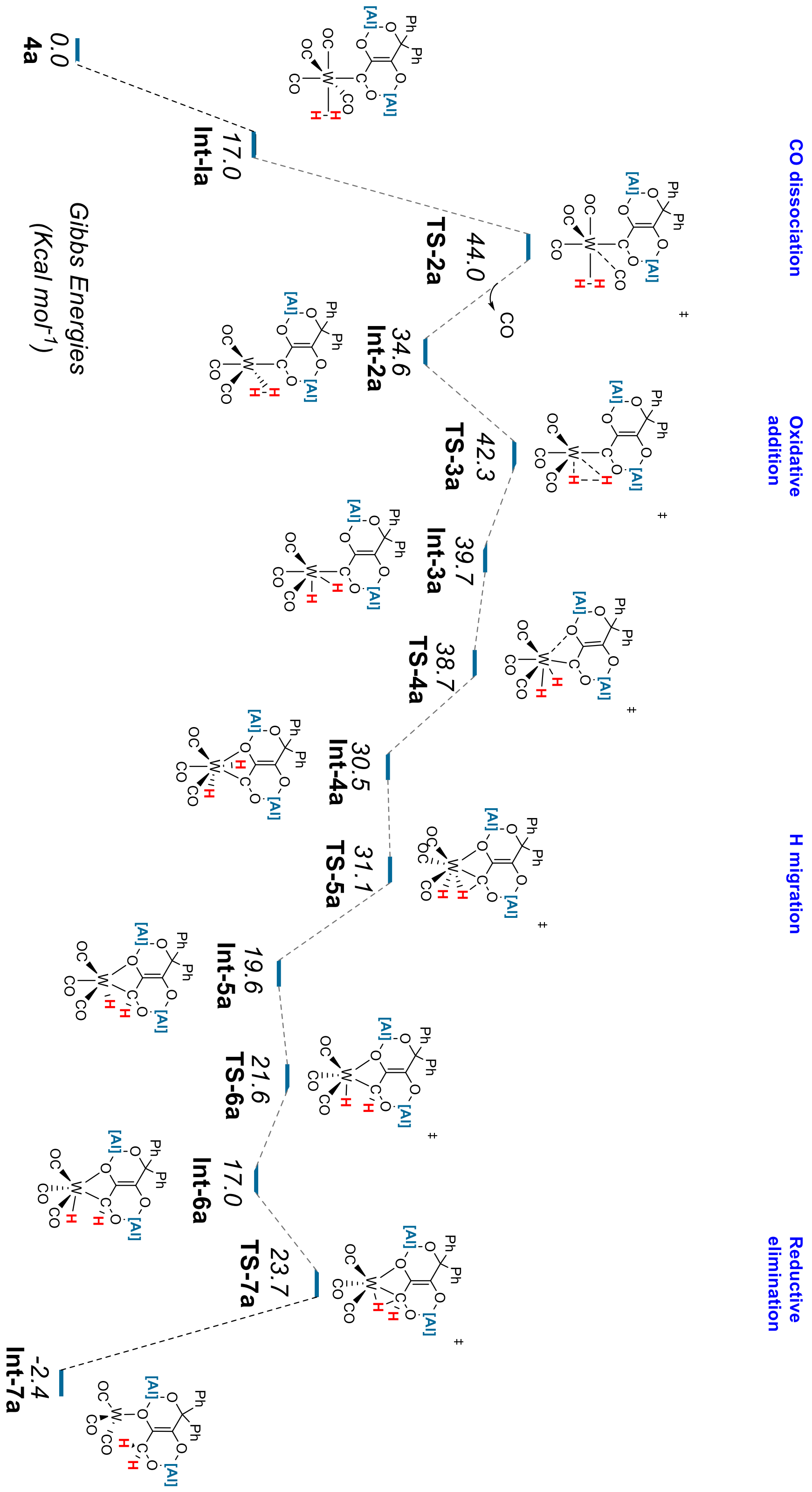

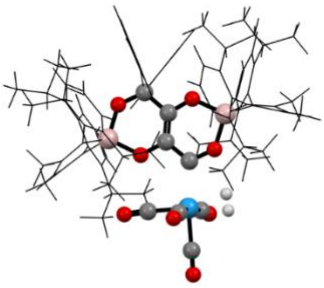

Int-1a

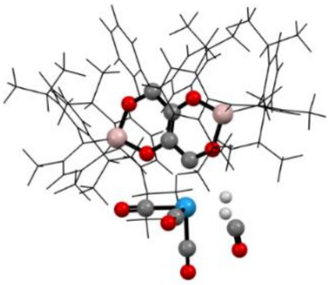

TS-1a

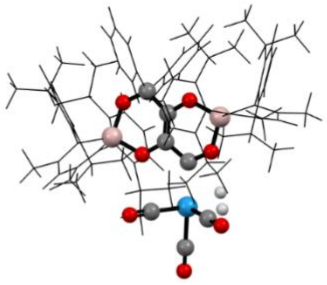

Int-2a

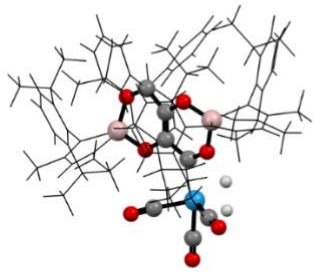

TS-2a

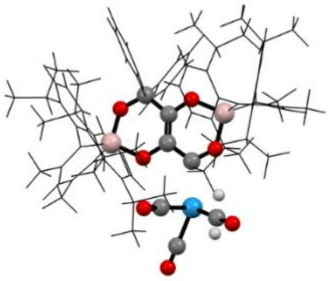

Int-3a

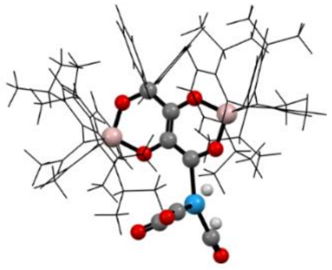

TS-3a

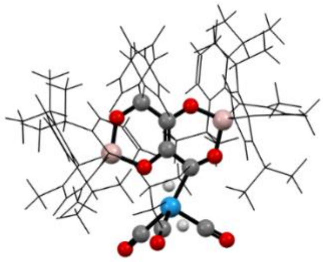

Int-4a

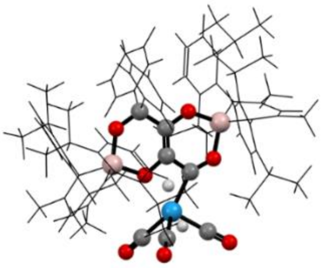

TS-4a

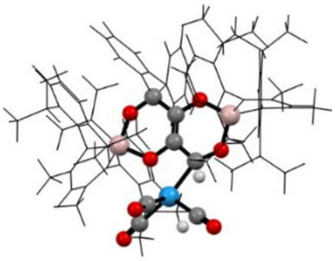

Int-5a

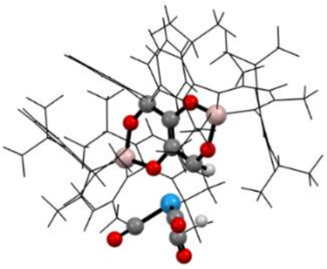

TS-5a

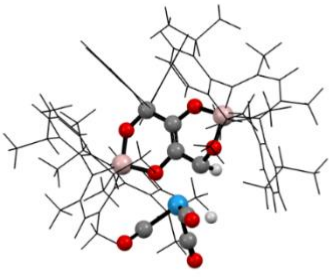

Int-6a

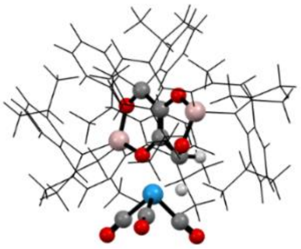

TS-6a

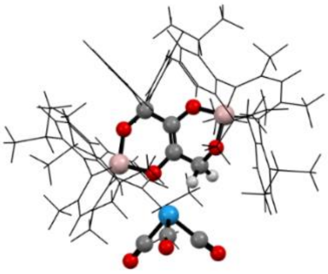

Int-7a

Figure S15: Calculated alternative pathway (*a*) for hydrogenation of **4a**. All energies in kcal mol^{-1}.

Alternative calculated mechanism (*b*) for hydrogenation of **4a**: concerted CO dissociation migration.

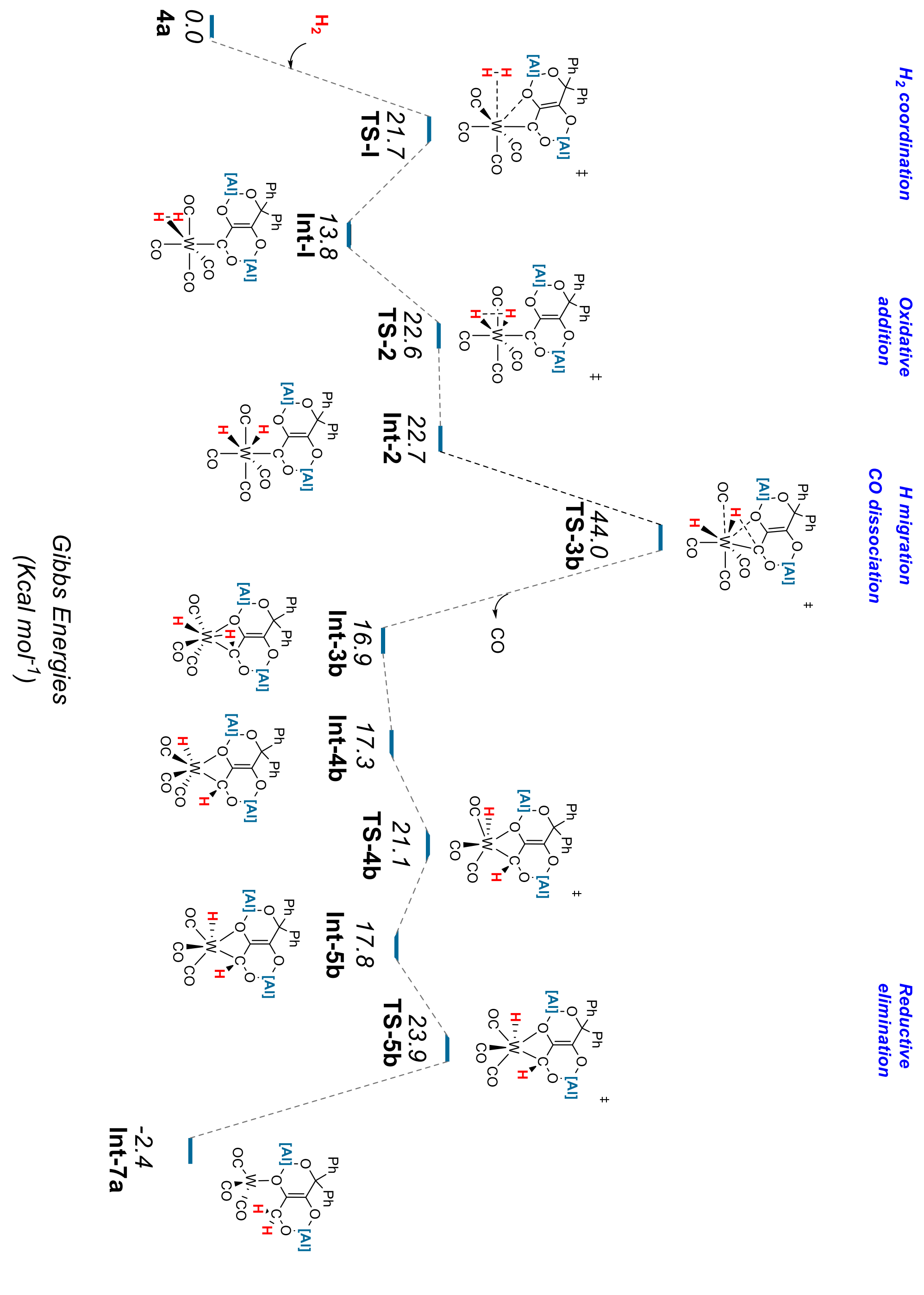

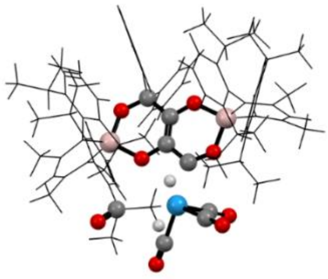

TS-3b

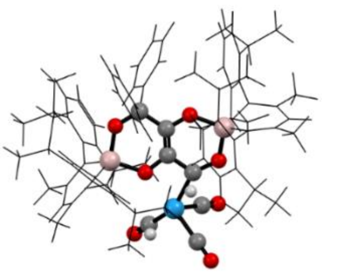

Int-3b

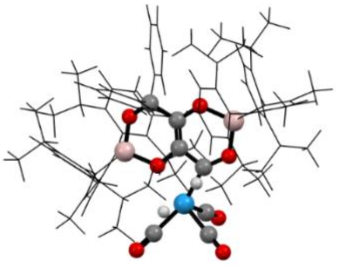

Int-4b

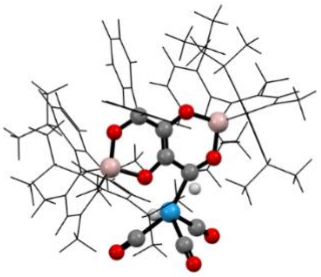

TS-4b

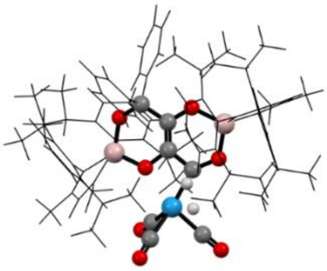

Int-5b

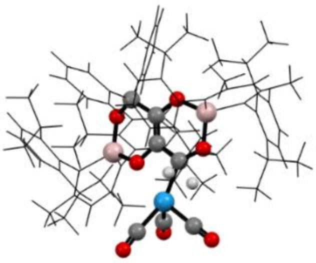

TS-5b

Figure S16: Calculated alternative pathway (*b*) for hydrogenation of **4a**. All energies in kcal mol^{-1}.

Alternative calculated mechanism (*c*) for hydrogenation of **4a**: without CO dissociation.

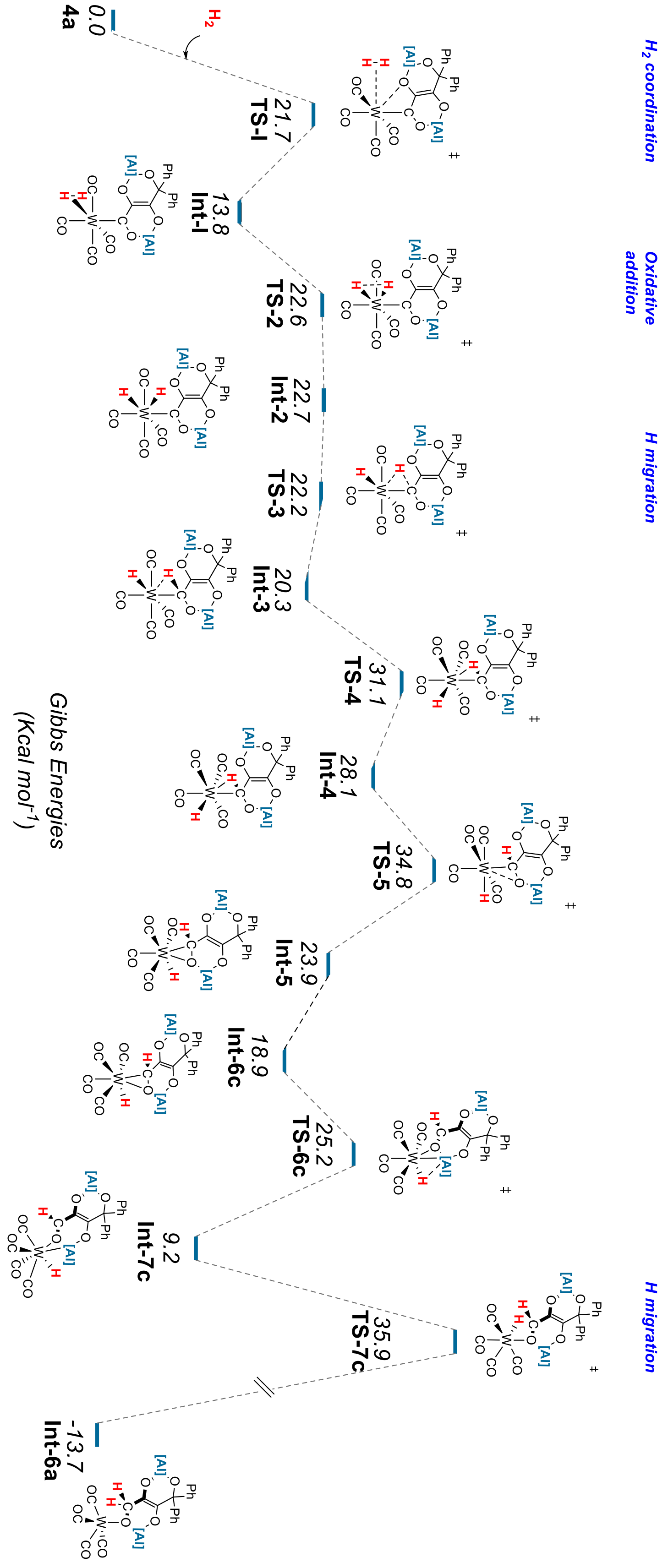

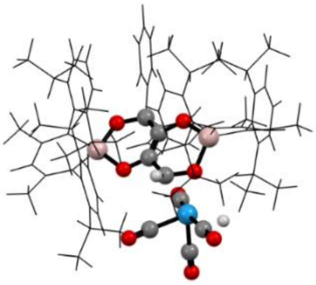

Int-6c

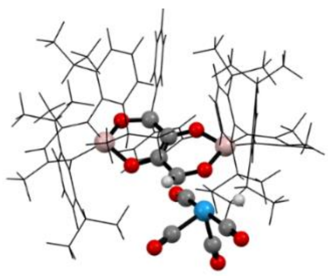

TS-6c

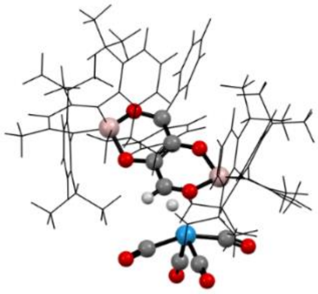

Int-7c

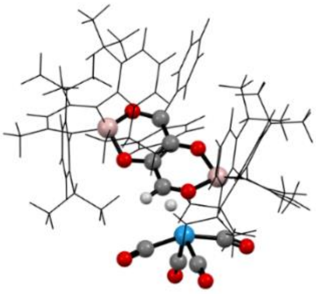

TS-7c

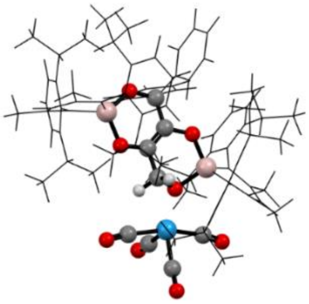

Int-6a

Figure S17: Calculated alternative pathway (*c*) for hydrogenation of **4a**. All energies in kcal mol^{-1}.

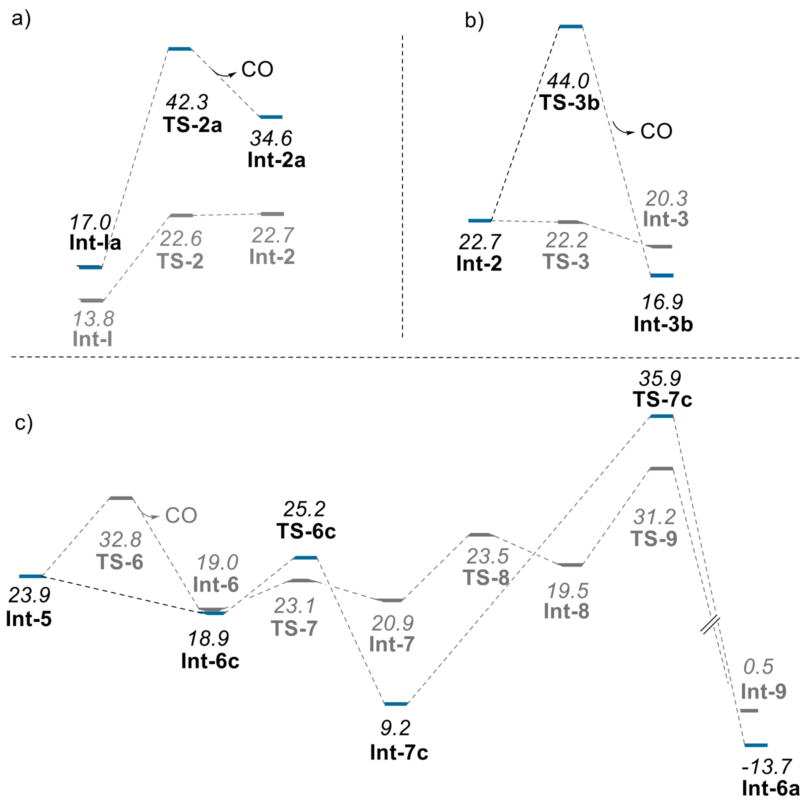

Figure S18: Comparison of key steps of alternative calculated mechanisms (a), (b) and (c) for hydrogenation of **4a** with most plausible mechanism. All energies in kcal mol^{-1}.

4.2.3 – Rotation barriers for **Int-1**

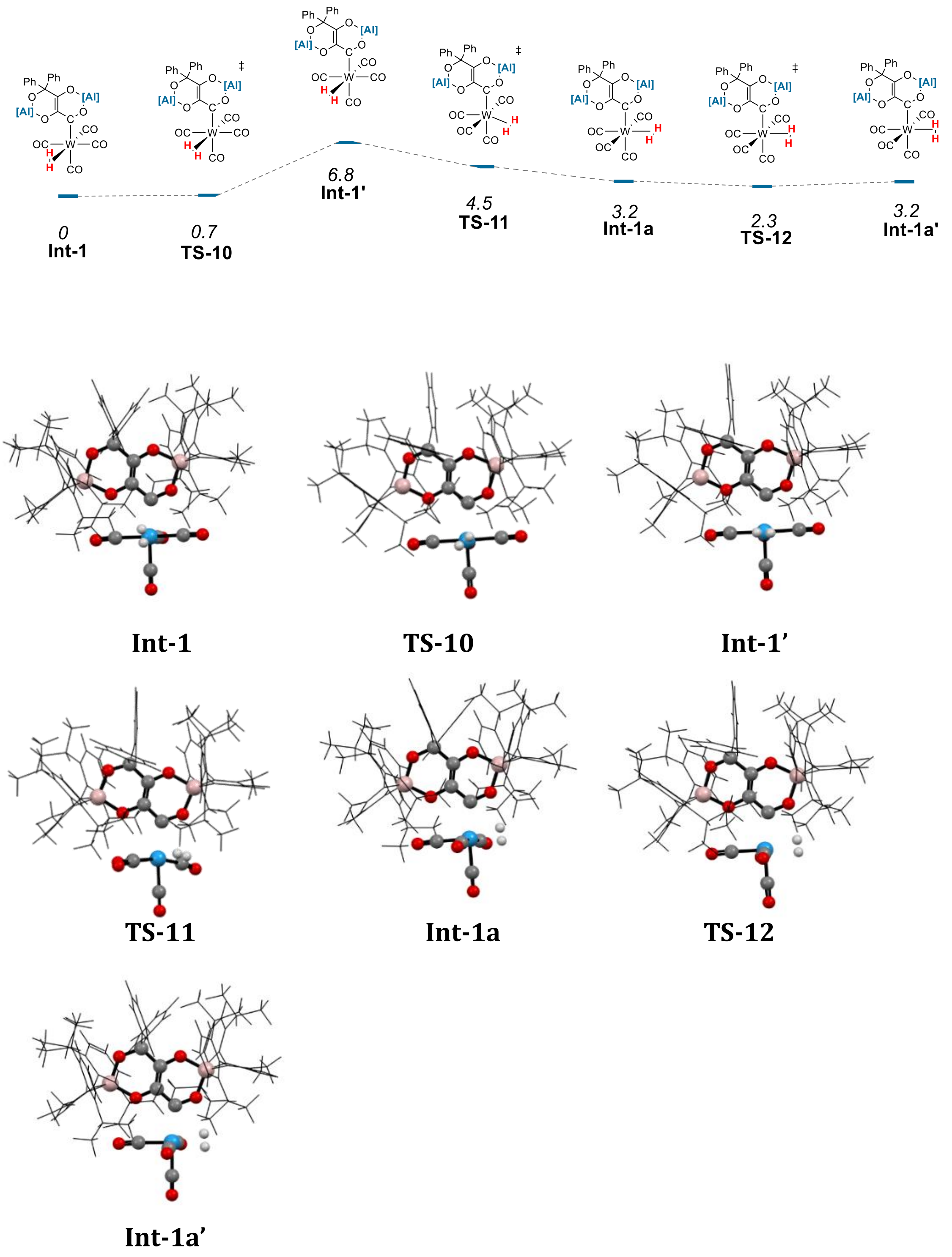

Figure S19: Calculated mechanism for isomerization of **Int-1**. All energies in kcal mol^{-1}.

4.3 - Functional testing on key stationary points

To investigate the hydrogenation reaction of **4a**, functional testing was performed on key stationary points within the calculated pathways. A simplified version of **4a** (**4a'**) was used for these calculations.

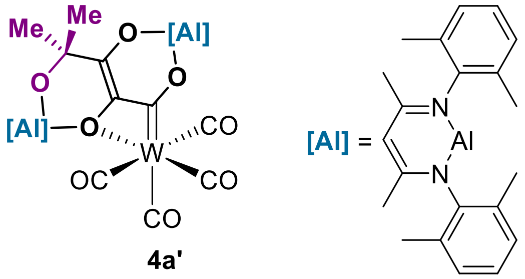

4a'

	TS-2	*TS-6*	*TS-9*	*TS-2a*	*TS-3b*	*Int-7c*	*TS-7c*
ωB97xD	20.0	31.1	35.7	42.5	42.0	1.7	33.2
M06l	21.9	37.0	40.5	40.5	48.2	0.1	26.8
b3pw91	21.1	36.8	38.7	46.9	49.4	-0.1	29.5
b3lyp	21.8	38.8	41.3	46.3	49.7	1.0	-

Table S4: All Gibbs free energies provided in kcal mol^{-1}. Gibbs free energies relative to **4a'**. **TS-7c** with b3lyp functional could not be found.

5 NMR SPECTRA

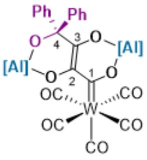

3a

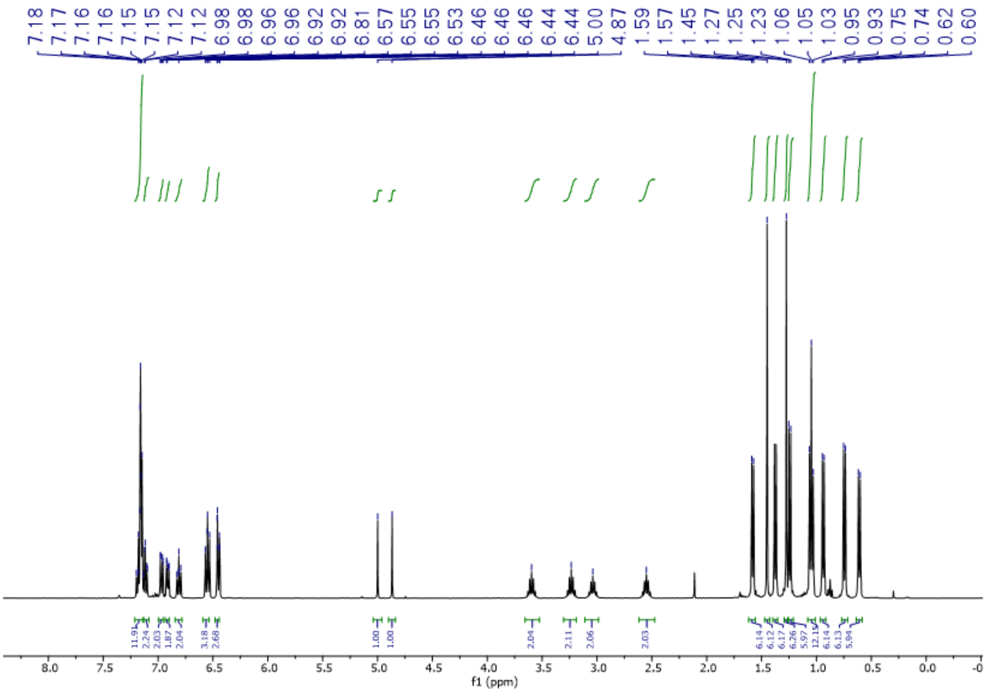

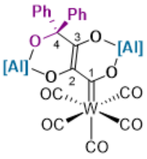

3a

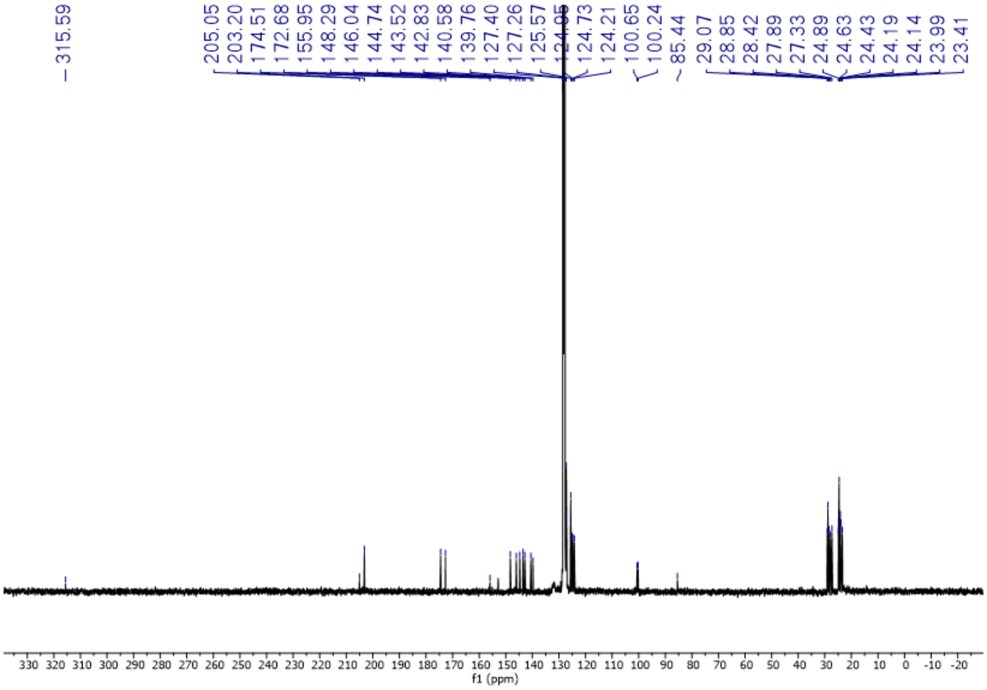

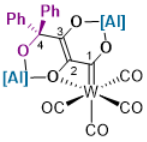

Ph
Ph
O—[Al]
O
[Al]—O
CO
OC
CO
CO
W

4a

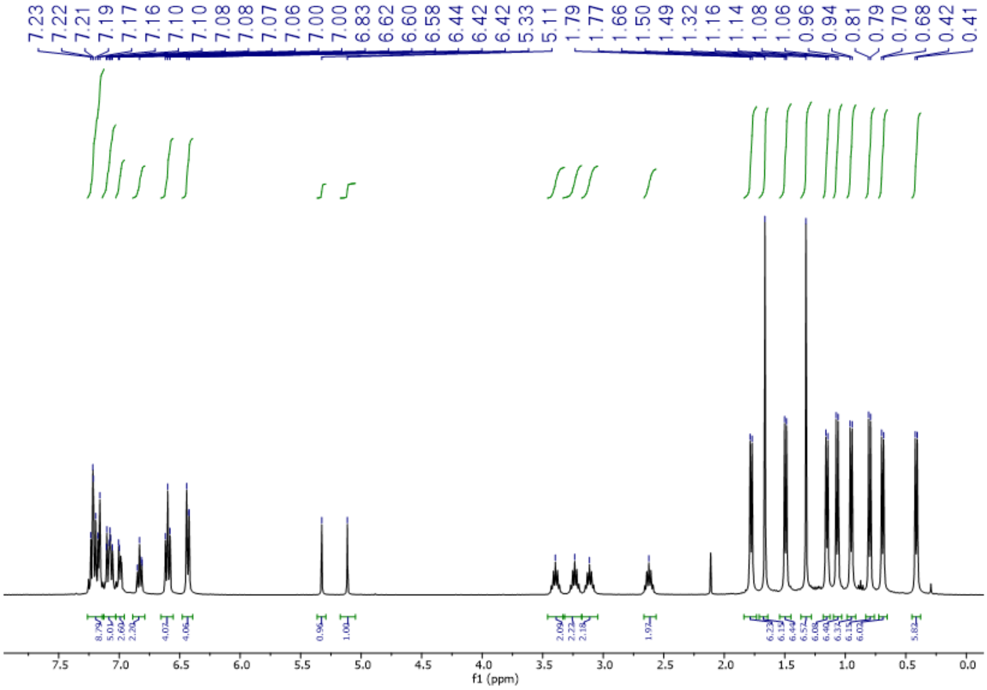

7.23 7.22 7.21 7.19 7.17 7.16 7.10 7.10 7.08 7.08 7.07 7.06 7.00 7.00 6.83 6.62 6.60 6.58 6.44 6.42 6.42 5.33 5.11 1.79 1.77 1.66 1.50 1.49 1.32 1.16 1.14 1.08 1.06 0.96 0.94 0.81 0.79 0.70 0.68 0.42 0.41
f1 (ppm)

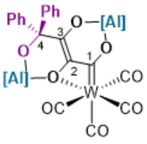

Ph
Ph
O—[Al]
O
[Al]—O
CO
OC
CO
CO
W

4a

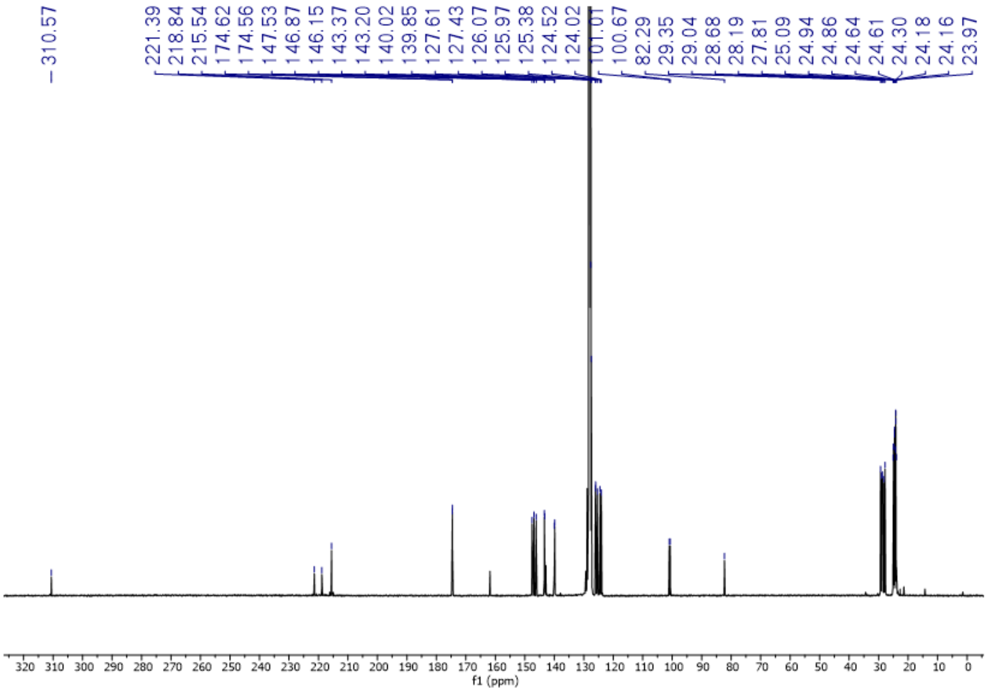

310.57
221.39 218.84 215.54 174.62 174.56 147.53 146.87 146.15 143.37 143.20 140.02 139.85 127.61 127.43 126.07 125.97 125.38 124.52 124.02 101.01 100.67 82.29 29.35 29.04 28.68 28.19 27.81 25.09 24.94 24.86 24.64 24.61 24.30 24.18 24.16 23.97
f1 (ppm)

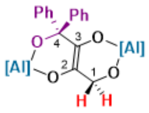
Ph
Ph
O
O
[Al]
[Al]
O
O
H
H

5a

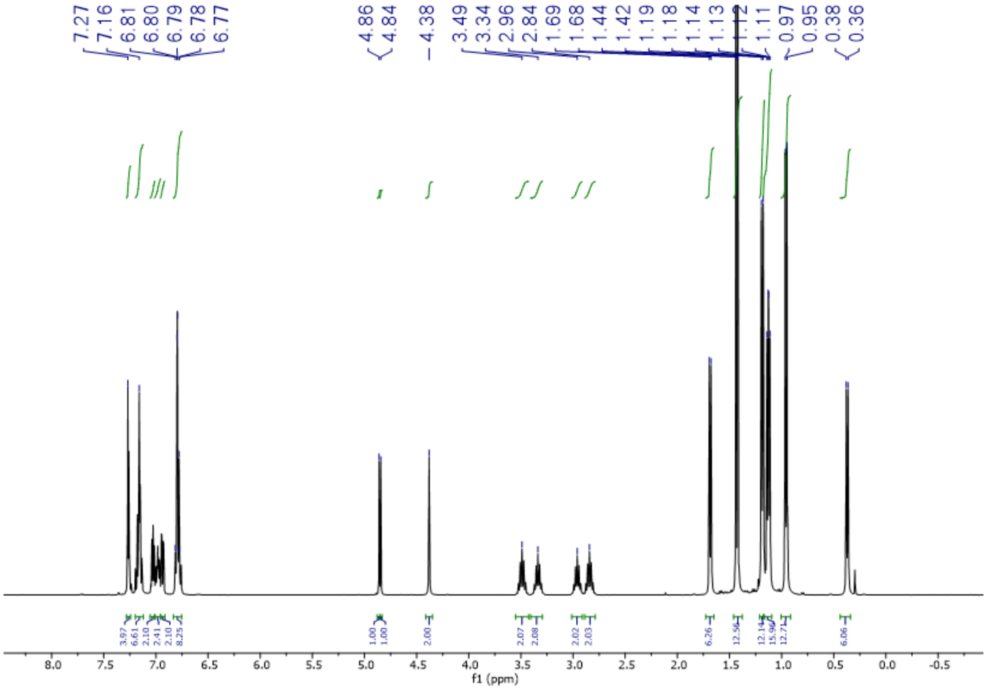
7.27
7.16
6.81
6.80
6.79
6.78
6.77
4.86
4.84
4.38
3.49
3.34
2.96
2.84
1.69
1.68
1.44
1.42
1.19
1.18
1.14
1.13
1.12
1.11
0.97
0.95
0.38
0.36
3.97
6.61
2.10
2.41
2.10
8.25
1.00
1.00
2.00
2.07
2.08
2.02
2.03
6.26
12.56
12.14
15.96
12.71
6.06
f1 (ppm)

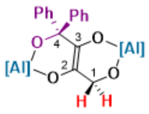
Ph
Ph
O
O
[Al]
[Al]
O
O
H
H

5a

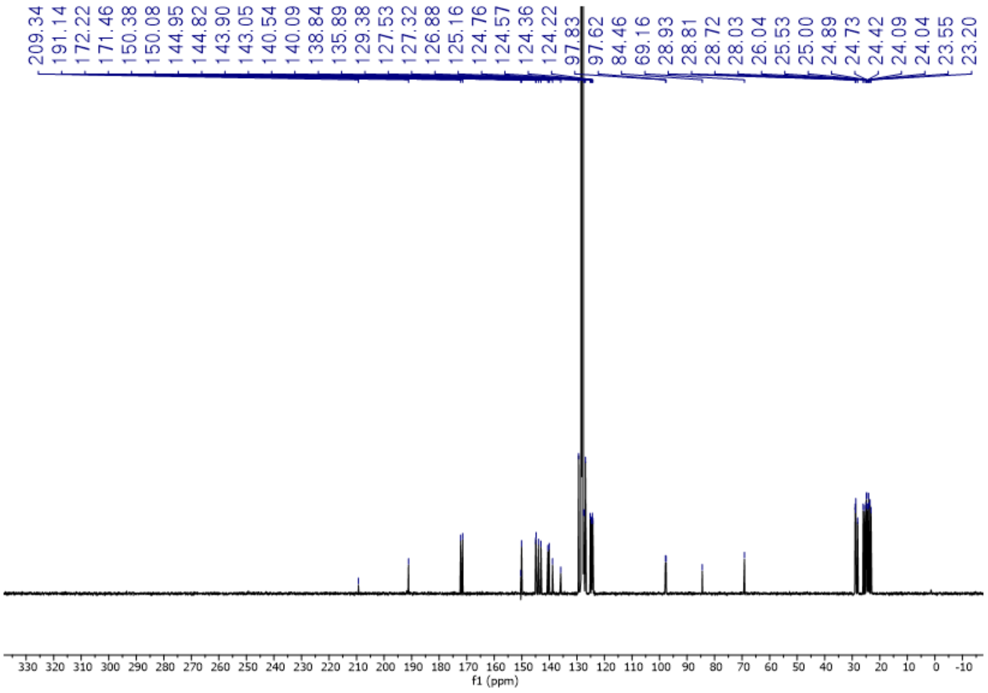
209.34
191.14
172.22
171.46
150.38
150.08
144.95
144.82
143.90
143.05
140.54
140.09
138.84
135.89
129.38
127.53
127.32
126.88
125.16
124.76
124.57
124.36
124.22
97.83
97.62
84.46
69.16
28.93
28.81
28.72
28.03
26.04
25.53
25.00
24.89
24.73
24.42
24.09
24.04
23.55
23.20
f1 (ppm)

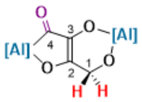

O
[Al]
[Al]
O
O
O
4
3
2
1
H
H

5b

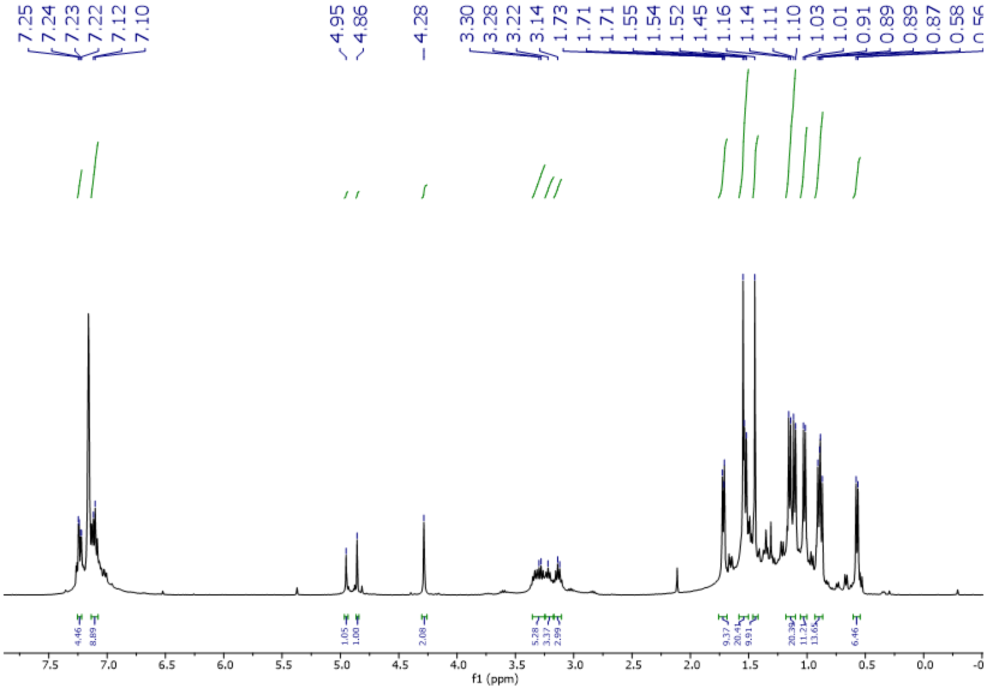

7.25
7.24
7.23
7.22
7.12
7.10
4.95
4.86
4.28
3.30
3.28
3.22
3.14
1.73
1.71
1.71
1.55
1.54
1.52
1.45
1.16
1.14
1.11
1.10
1.03
1.01
0.91
0.89
0.89
0.87
0.58
4.46
8.89
1.05
1.00
2.08
5.28
3.37
2.99
9.37
20.41
9.91
20.39
11.21
13.65
6.46
f1 (ppm)

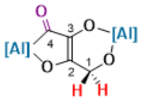

O
[Al]
[Al]
O
O
O
4
3
2
1
H
H

5b

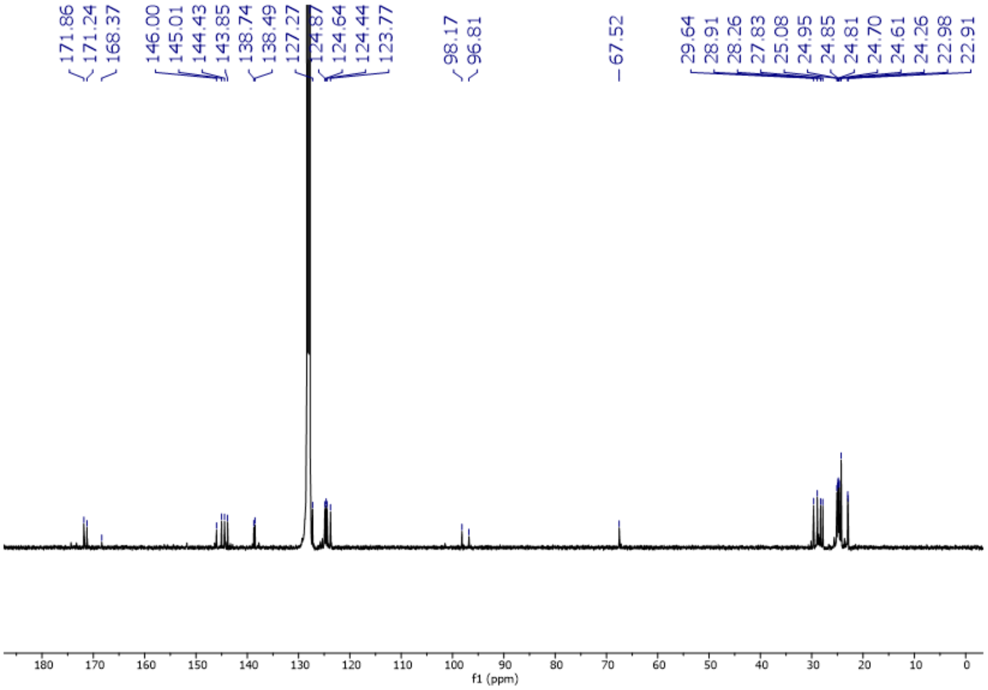

171.86
171.24
168.37
146.00
145.01
144.43
143.85
138.74
138.49
127.27
124.87
124.64
124.44
123.77
98.17
96.81
67.52
29.64
28.91
28.26
27.83
25.08
24.95
24.85
24.81
24.70
24.61
24.26
22.98
22.91
f1 (ppm)

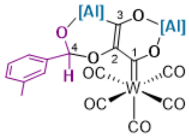
[Al]
[Al]
OC
CO
OC
CO
CO
W
H

S1

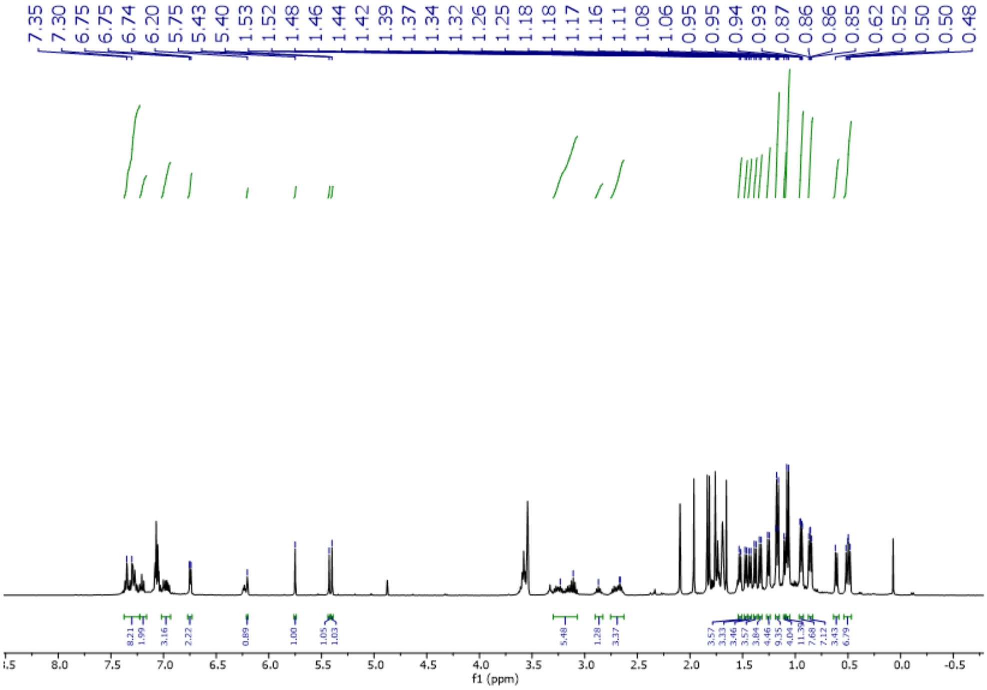
7.35 7.30 6.75 6.75 6.74 6.20 5.75 5.43 5.40 1.53 1.52 1.48 1.46 1.44 1.42 1.39 1.37 1.34 1.32 1.26 1.25 1.18 1.18 1.17 1.16 1.11 1.08 1.06 0.95 0.95 0.94 0.93 0.87 0.86 0.86 0.85 0.62 0.52 0.50 0.48
8.21 1.99 3.16 2.22 0.89 1.00 1.05 1.03 5.48 1.28 3.37 3.57 3.33 3.46 3.57 3.84 4.46 9.35 4.04 11.39 7.68 7.12 3.43 6.79
f1 (ppm)

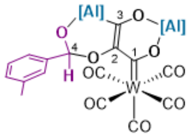
[Al]
[Al]
OC
CO
OC
CO
CO
W
H

S1

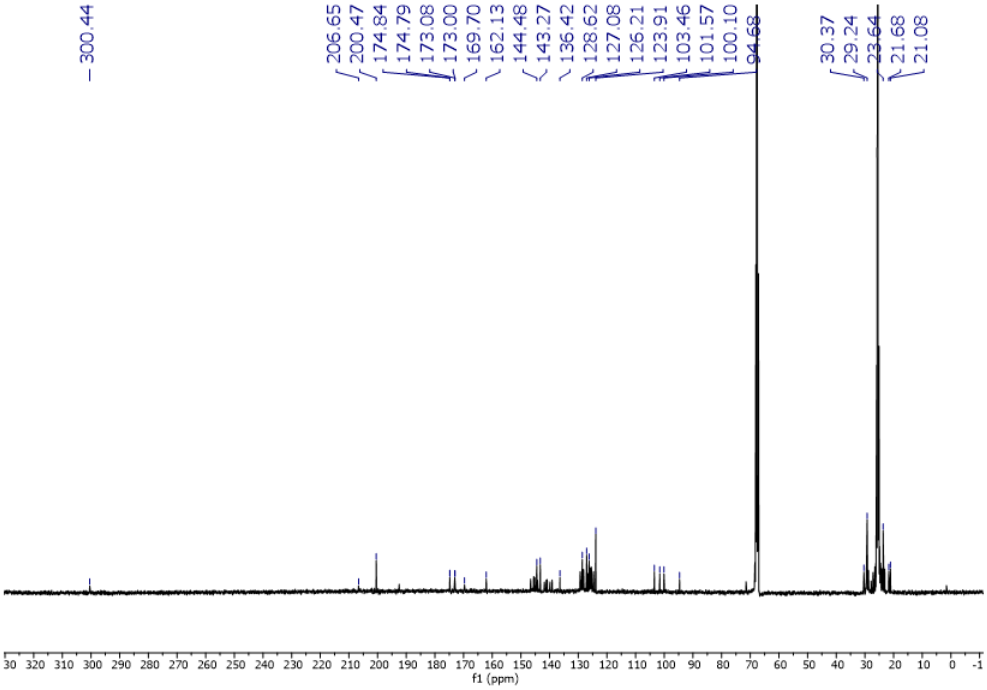
300.44
206.65 200.47 174.84 174.79 173.08 173.00 169.70 162.13 144.48 143.27 136.42 128.62 127.08 126.21 123.91 103.46 101.57 100.10 94.68
30.37 29.24 23.64 21.68 21.08
f1 (ppm)

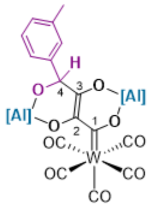

H
O
[Al]
O
O
[Al]
O
OC
CO
OC
CO
CO
W

3c

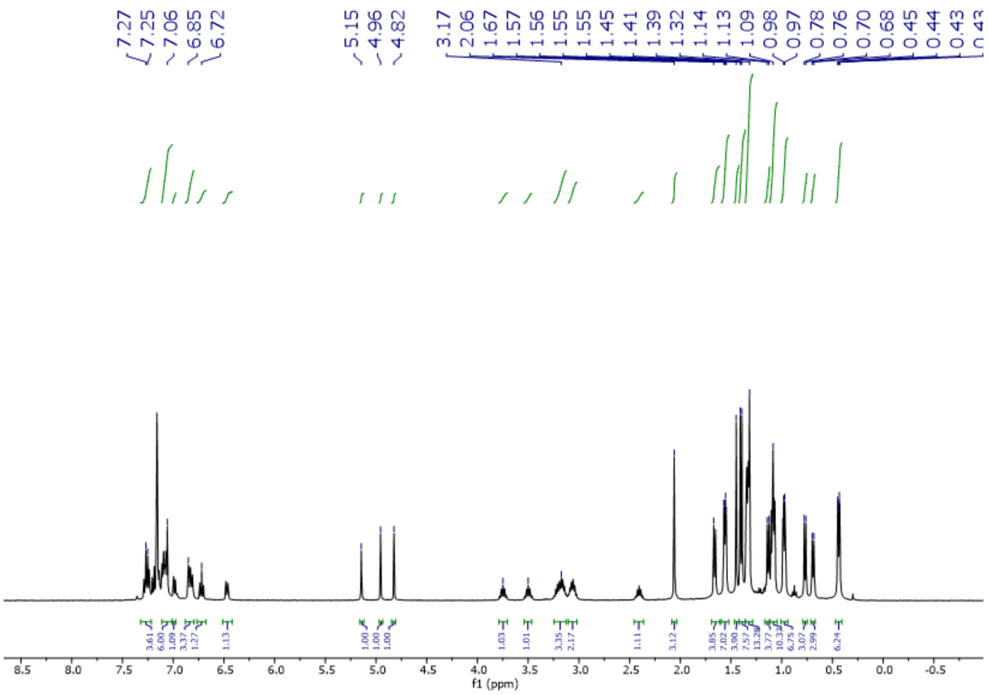

7.27
7.25
7.06
6.85
6.72
5.15
4.96
4.82
3.17
2.06
1.67
1.57
1.56
1.55
1.55
1.45
1.41
1.39
1.32
1.14
1.13
1.09
0.98
0.97
0.78
0.76
0.70
0.68
0.45
0.44
0.43
f1 (ppm)

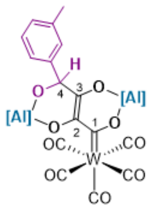

H
O
[Al]
O
O
[Al]
O
OC
CO
OC
CO
CO
W

3c

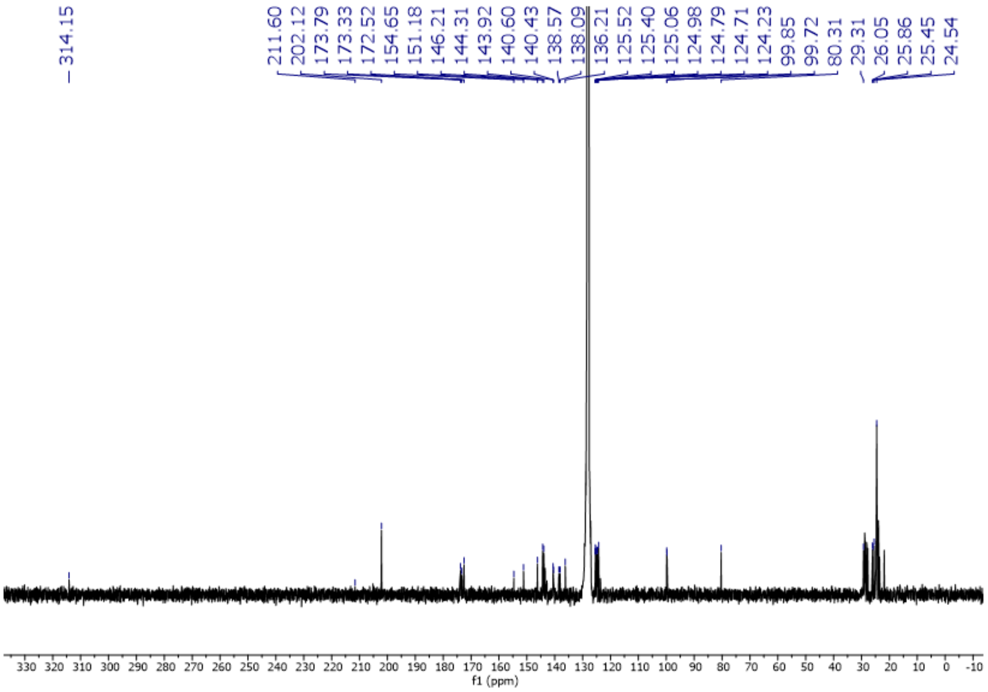

314.15
211.60
202.12
173.79
173.33
172.52
154.65
151.18
146.21
144.31
143.92
140.60
140.43
138.57
138.09
136.21
125.52
125.40
125.06
124.98
124.79
124.71
124.23
99.85
99.72
80.31
29.31
26.05
25.86
25.45
24.54
f1 (ppm)

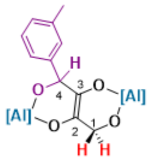

H
O
[Al]
O
O
O
[Al]
H H

5c

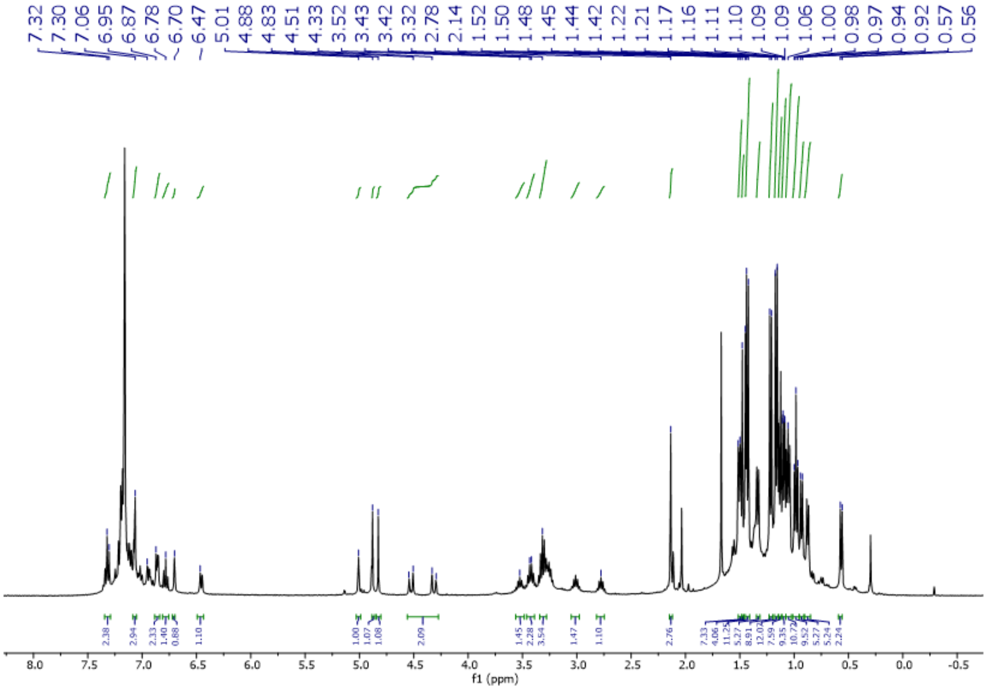

7.32 7.30 7.06 6.95 6.87 6.78 6.70 6.47 5.01 4.88 4.83 4.51 4.33 3.52 3.43 3.42 3.32 2.78 2.14 1.52 1.50 1.48 1.45 1.44 1.42 1.22 1.21 1.17 1.16 1.11 1.10 1.09 1.09 1.06 1.00 0.98 0.97 0.94 0.92 0.57 0.56
f1 (ppm)

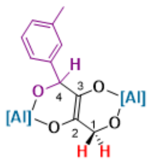

H
O
[Al]
O
O
O
[Al]
H H

5c

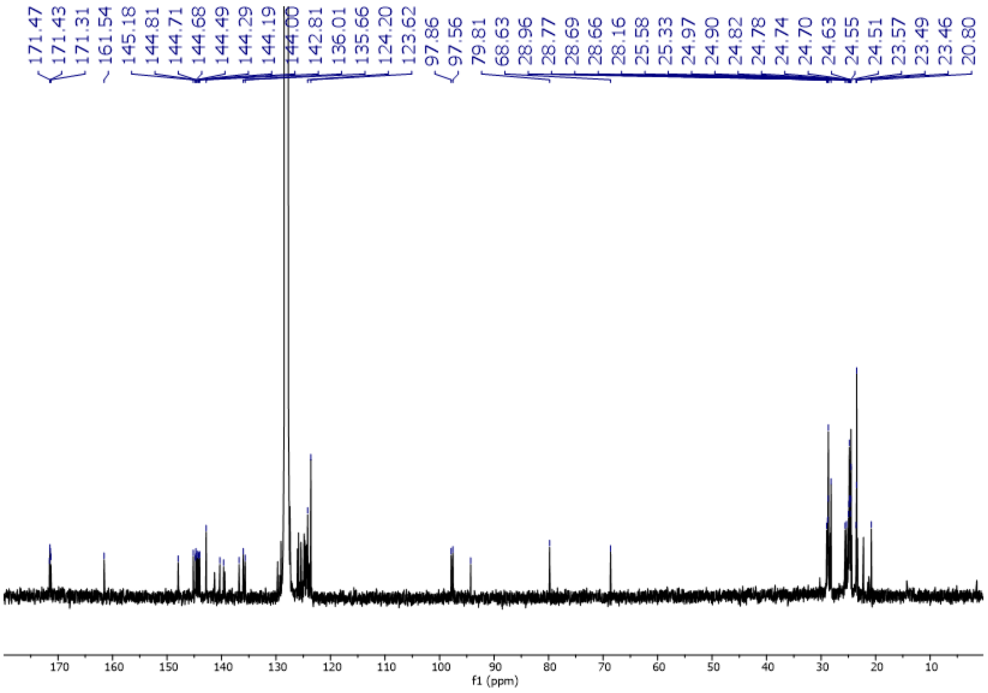

171.47 171.43 171.31 161.54 145.18 144.81 144.71 144.68 144.49 144.29 144.19 144.00 142.81 136.01 135.66 124.20 123.62 97.86 97.56 79.81 68.63 28.96 28.77 28.69 28.66 28.16 25.58 25.33 24.97 24.90 24.82 24.78 24.74 24.70 24.63 24.55 24.51 23.57 23.49 23.46 20.80
f1 (ppm)

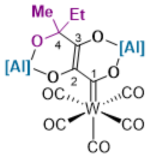
Me
Et
[Al]
[Al]
OC
CO
OC
CO
CO
W

3d

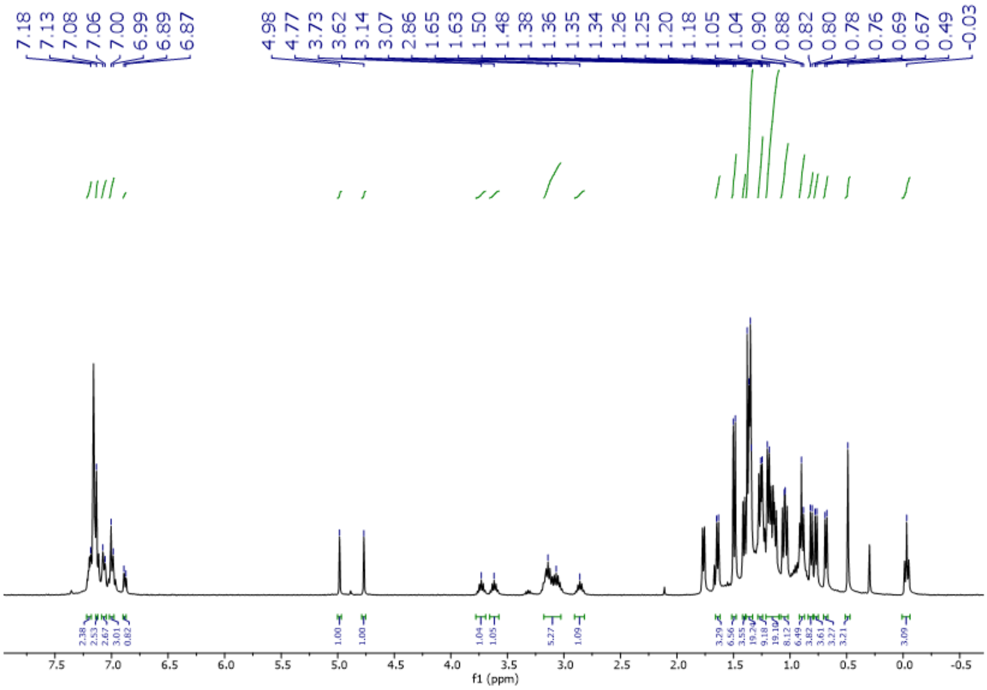
7.18
7.13
7.08
7.06
7.00
6.99
6.89
6.87
4.98
4.77
3.73
3.62
3.14
3.07
2.86
1.65
1.63
1.50
1.48
1.38
1.36
1.35
1.34
1.26
1.25
1.20
1.18
1.05
1.04
0.90
0.88
0.82
0.80
0.78
0.76
0.69
0.67
0.49
-0.03
f1 (ppm)

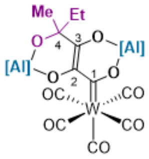
Me
Et
[Al]
[Al]
OC
CO
OC
CO
CO
W

3d

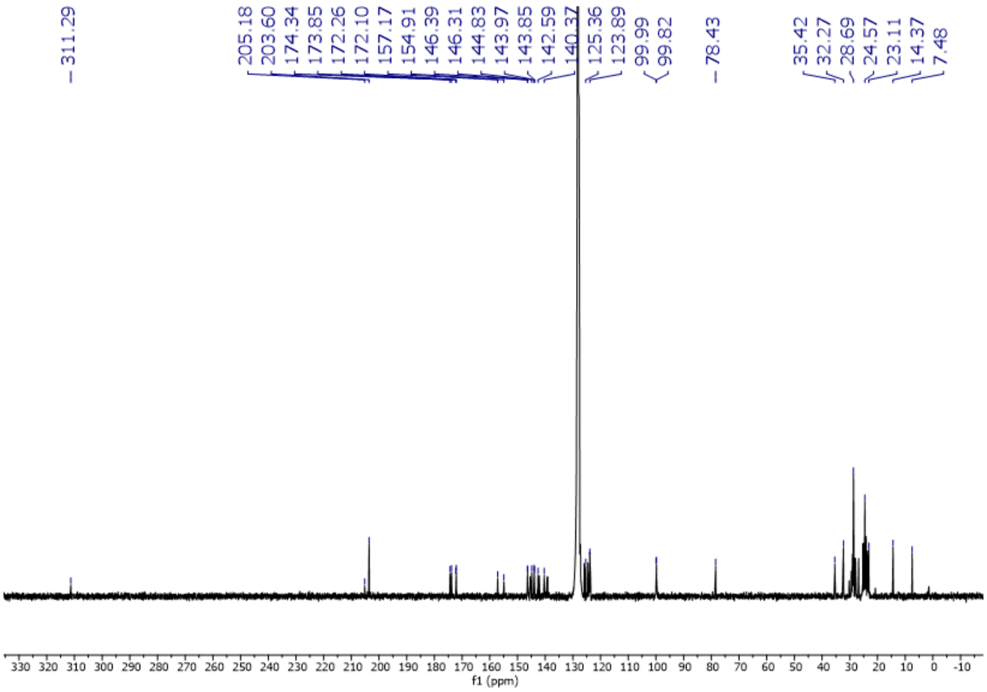
311.29
205.18
203.60
174.34
173.85
172.26
172.10
157.17
154.91
146.39
146.31
144.83
143.97
143.85
142.59
140.37
125.36
123.89
99.99
99.82
78.43
35.42
32.27
28.69
24.57
23.11
14.37
7.48
f1 (ppm)

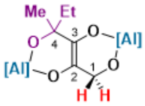

Me
Et
O
[Al]
[Al]
O
O
O
H
H

5d

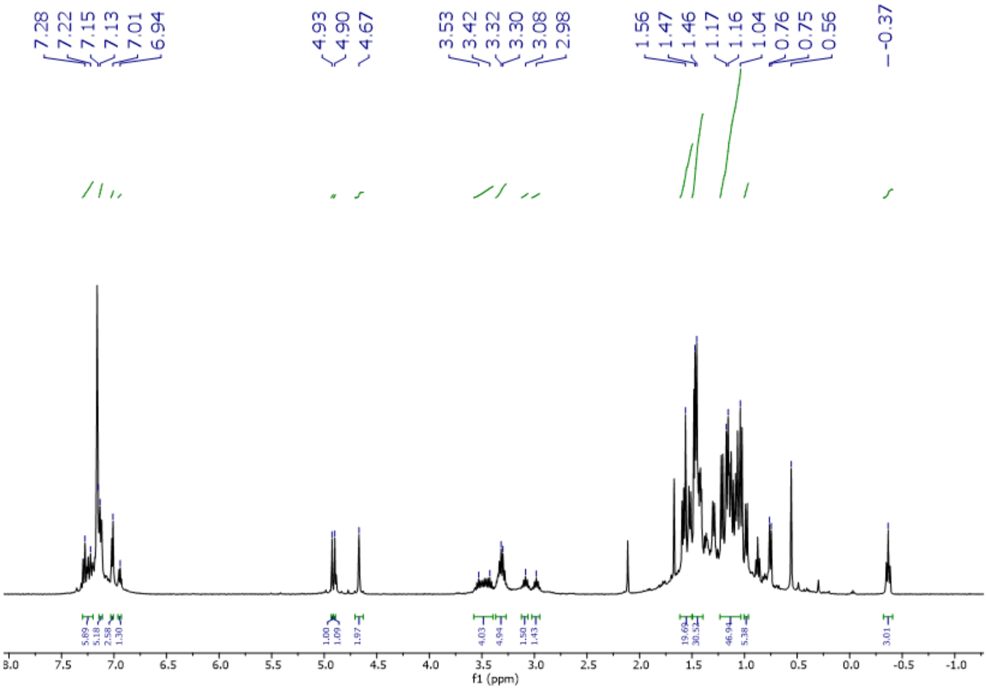

7.28
7.22
7.15
7.13
7.01
6.94
4.93
4.90
4.67
3.53
3.42
3.32
3.30
3.08
2.98
1.56
1.47
1.46
1.17
1.16
1.04
0.76
0.75
0.56
-0.37
f1 (ppm)

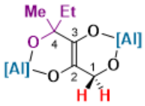

Me
Et
O
[Al]
[Al]
O
O
O
H
H

5d

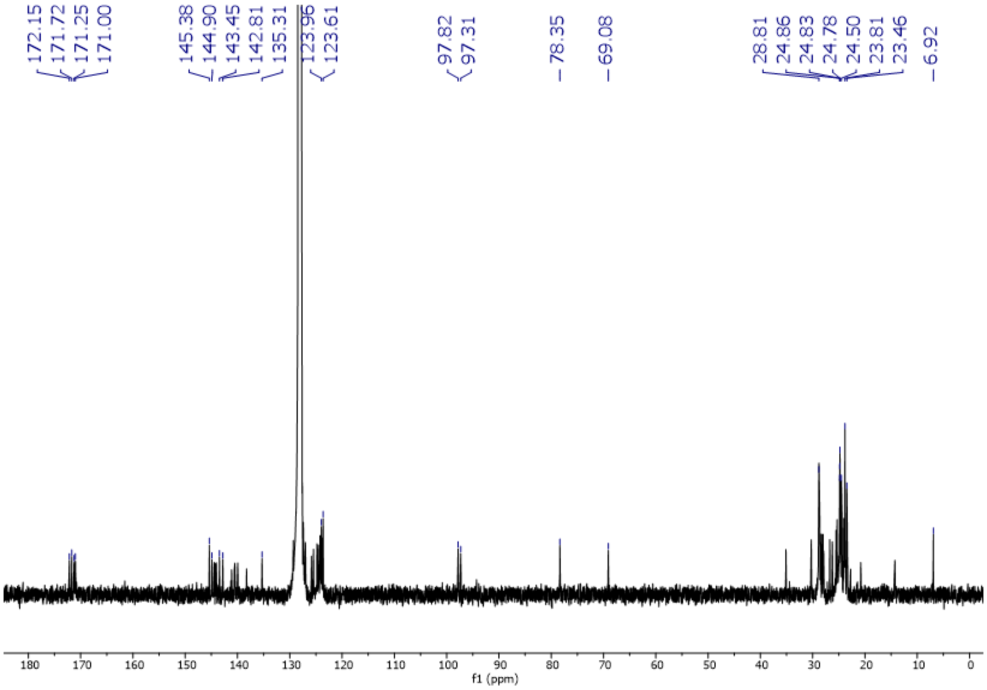

172.15
171.72
171.25
171.00
145.38
144.90
143.45
142.81
135.31
123.96
123.61
97.82
97.31
78.35
69.08
28.81
24.86
24.83
24.78
24.50
23.81
23.46
6.92
f1 (ppm)

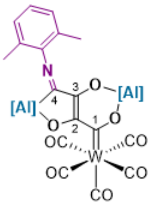
N
[Al]
O
[Al]
O
O
OC
CO
OC
CO
CO
W
3
4
2
1

3e

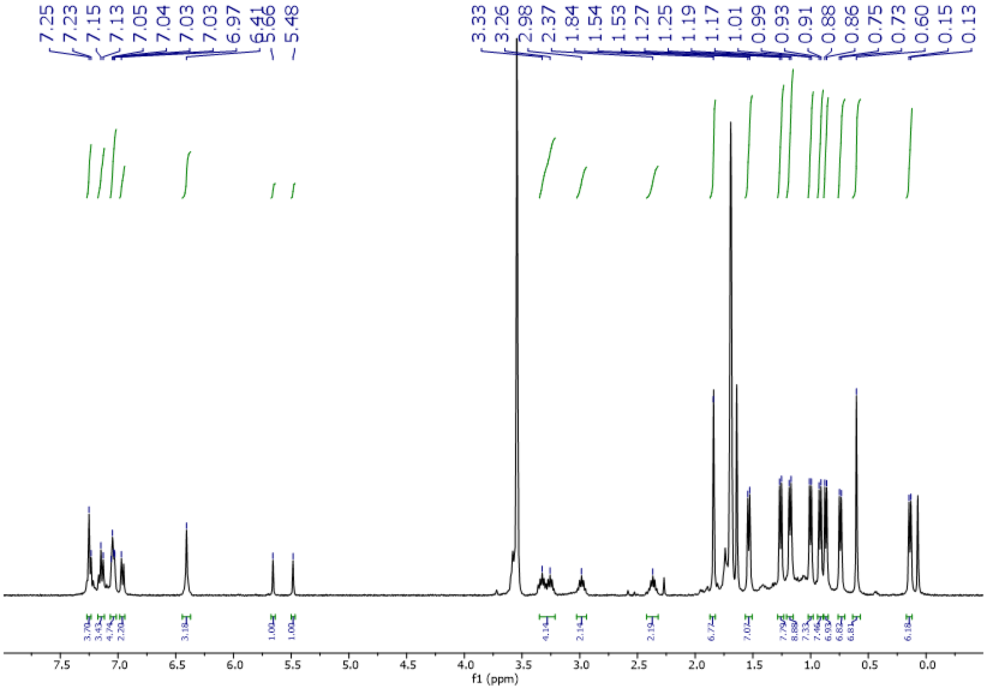
7.25
7.23
7.15
7.13
7.05
7.04
7.03
7.03
6.97
6.41
5.66
5.48
3.33
3.26
2.98
2.37
1.84
1.54
1.53
1.27
1.25
1.19
1.17
1.01
0.99
0.93
0.91
0.88
0.86
0.75
0.73
0.60
0.15
0.13
f1 (ppm)

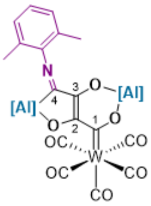
N
[Al]
O
[Al]
O
O
OC
CO
OC
CO
CO
W
3
4
2
1

3e

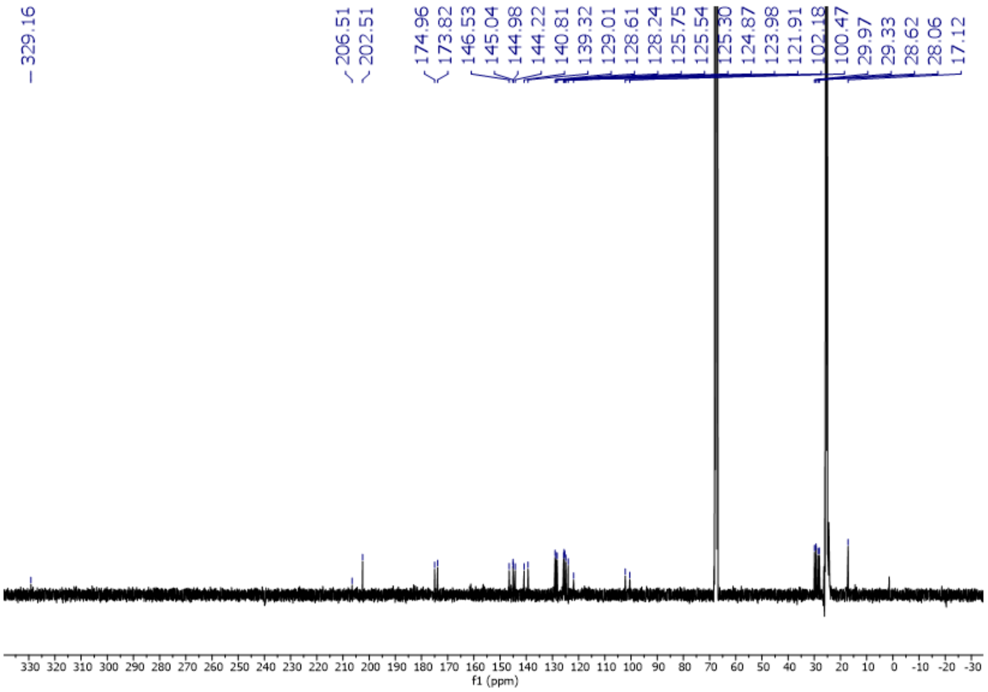
329.16
206.51
202.51
174.96
173.82
146.53
145.04
144.98
144.22
140.81
139.32
129.01
128.61
128.24
125.75
125.54
125.30
124.87
123.98
121.91
102.18
100.47
29.97
29.33
28.62
28.06
17.12
f1 (ppm)

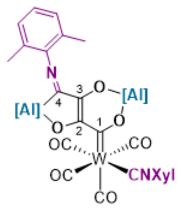
N
[Al]
O
[Al]
O
O
OC
CO
W
OC
CNXyl
CO

S2

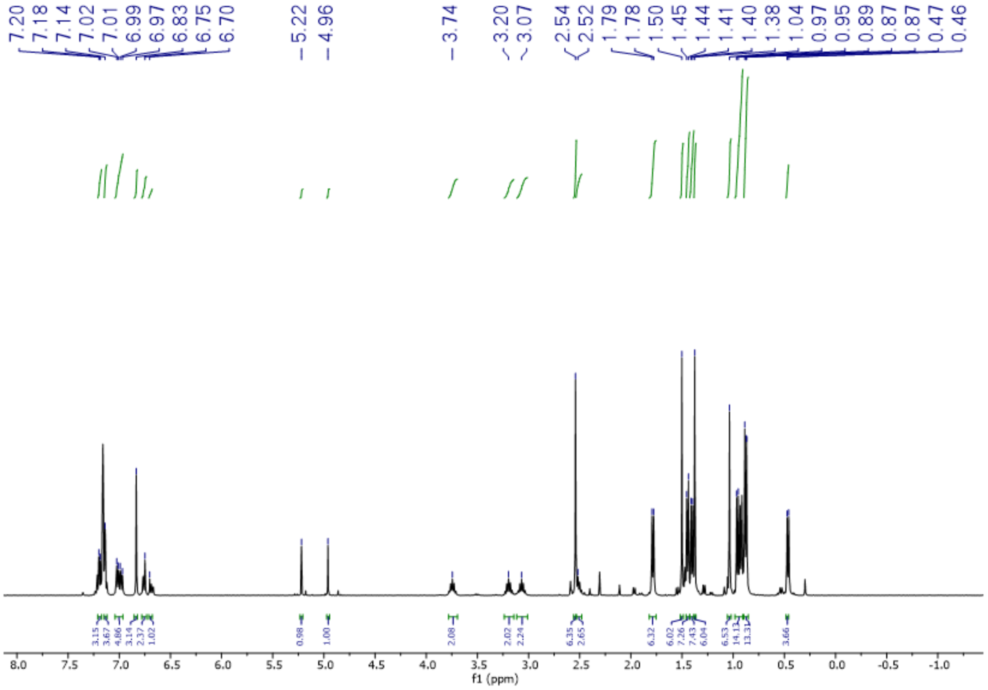
7.20
7.18
7.14
7.02
7.01
6.99
6.97
6.83
6.75
6.70
5.22
4.96
3.74
3.20
3.07
2.54
2.52
1.79
1.78
1.50
1.45
1.44
1.41
1.40
1.38
1.04
0.97
0.95
0.89
0.87
0.87
0.47
0.46
f1 (ppm)

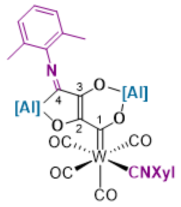
N
[Al]
O
[Al]
O
O
OC
CO
W
OC
CNXyl
CO

S2

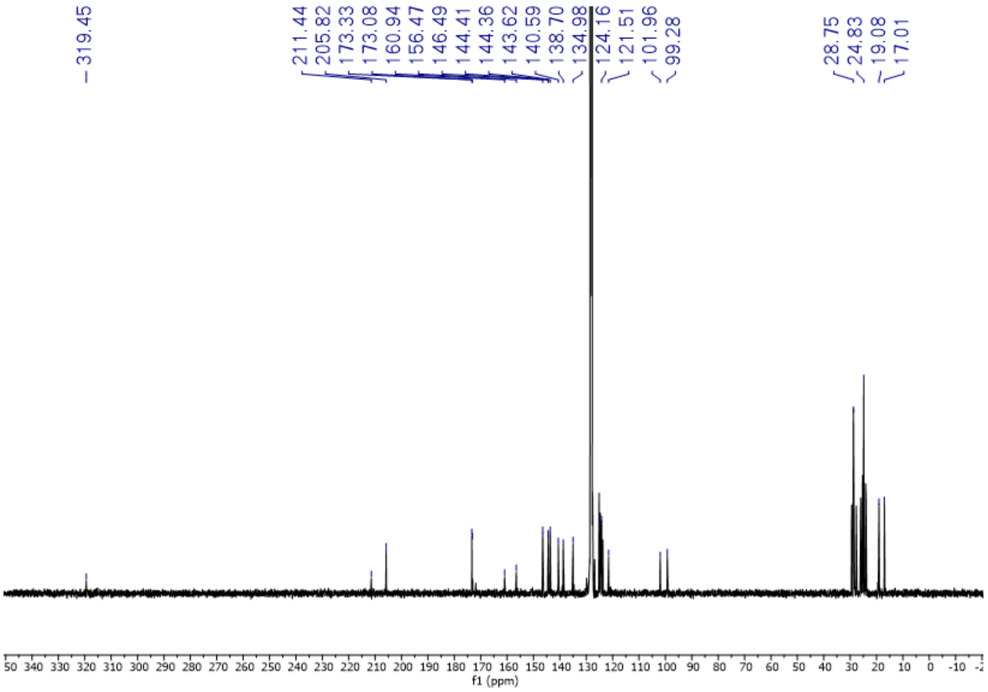
319.45
211.44
205.82
173.33
173.08
160.94
156.47
146.49
144.41
144.36
143.62
140.59
138.70
134.98
124.16
121.51
101.96
99.28
28.75
24.83
19.08
17.01
f1 (ppm)

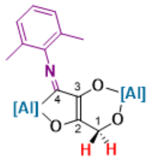
N
[Al]
[Al]
O
O
O
H
H
1
2
3
4

S3

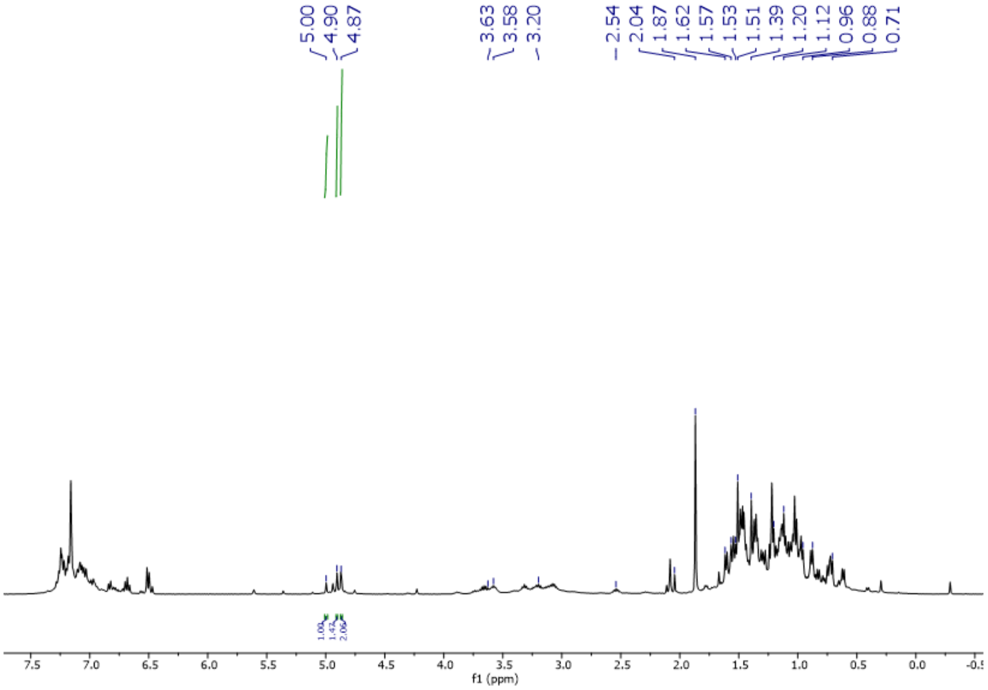
5.00
4.90
4.87
3.63
3.58
3.20
2.54
2.04
1.87
1.62
1.57
1.53
1.51
1.39
1.20
1.12
0.96
0.88
0.71
1.00
1.42
2.06
f1 (ppm)

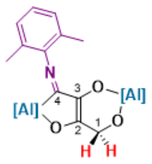
N
[Al]
[Al]
O
O
O
H
H
1
2
3
4

S3

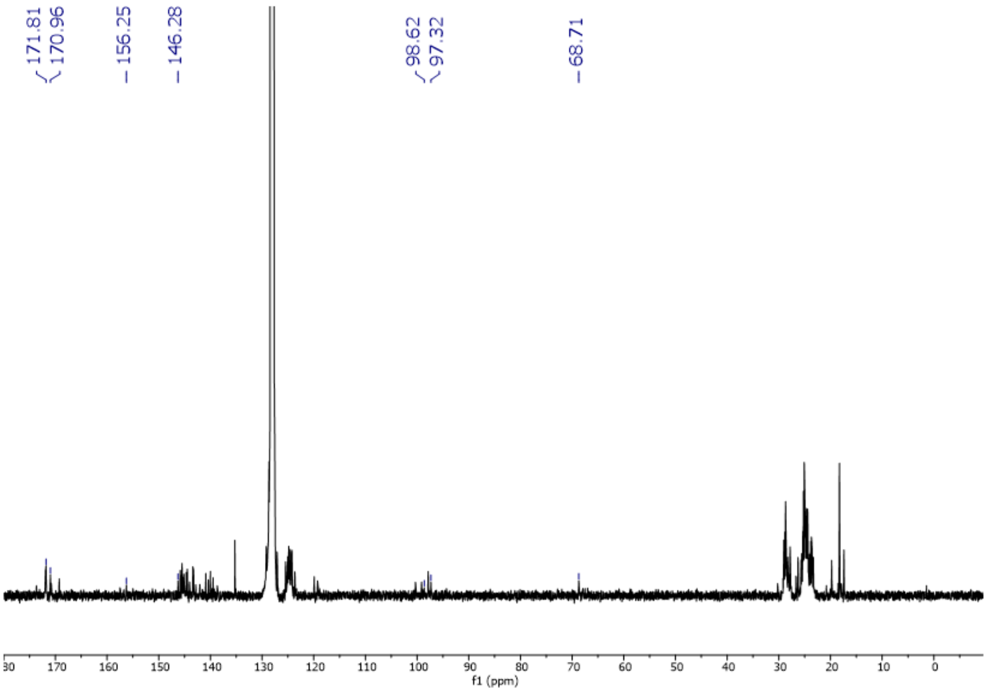
171.81
170.96
156.25
146.28
98.62
97.32
68.71
f1 (ppm)

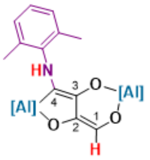
HN
O
[Al]
[Al]
O
O
3
4
1
2
H

5e

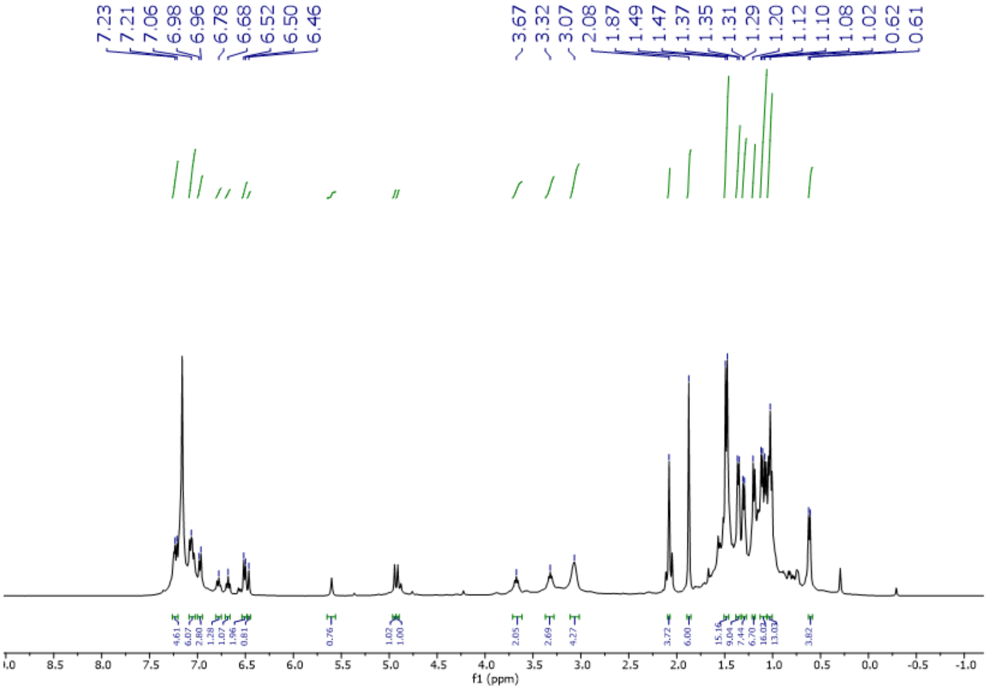
7.23
7.21
7.06
6.98
6.96
6.78
6.68
6.52
6.50
6.46
3.67
3.32
3.07
2.08
1.87
1.49
1.47
1.37
1.35
1.31
1.29
1.20
1.12
1.10
1.08
1.02
0.62
0.61
4.61
6.07
2.80
1.28
1.07
1.96
0.81
0.76
1.02
1.00
2.05
2.69
4.27
3.72
6.00
15.16
9.04
7.44
6.70
16.02
13.02
3.82
f1 (ppm)

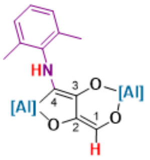
HN
O
[Al]
[Al]
O
O
3
4
1
2
H

5e

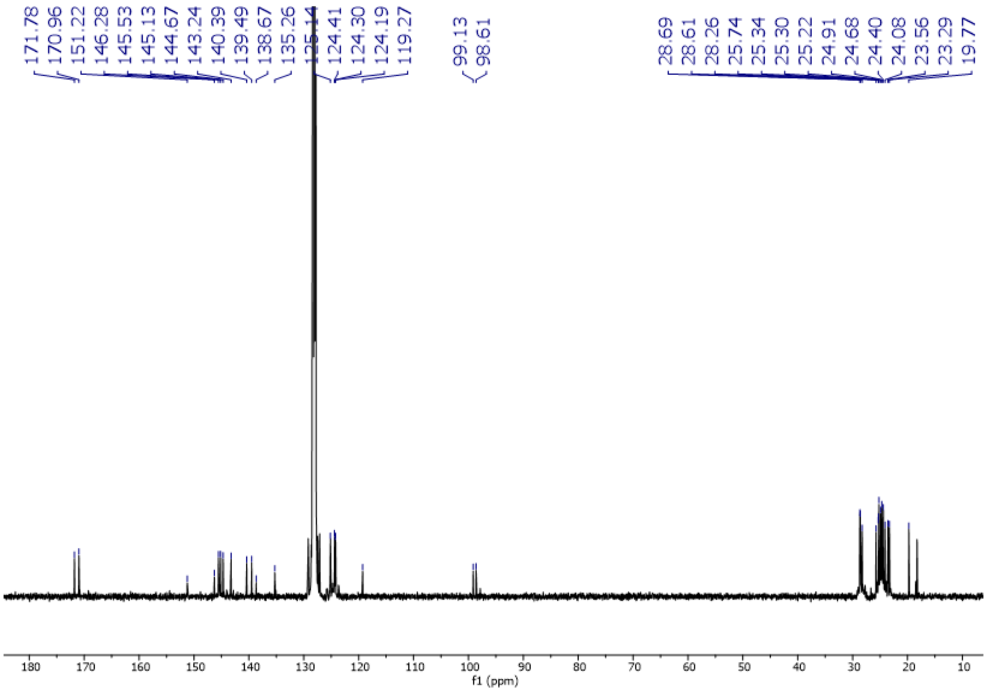
171.78
170.96
151.22
146.28
145.53
145.13
144.67
143.24
140.39
139.49
138.67
135.26
124.41
124.30
124.19
119.27
99.13
98.61
28.69
28.61
28.26
25.74
25.34
25.30
25.22
24.91
24.68
24.40
24.08
23.56
23.29
19.77
f1 (ppm)

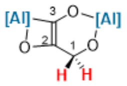
[Al]
[Al]
H H

5f

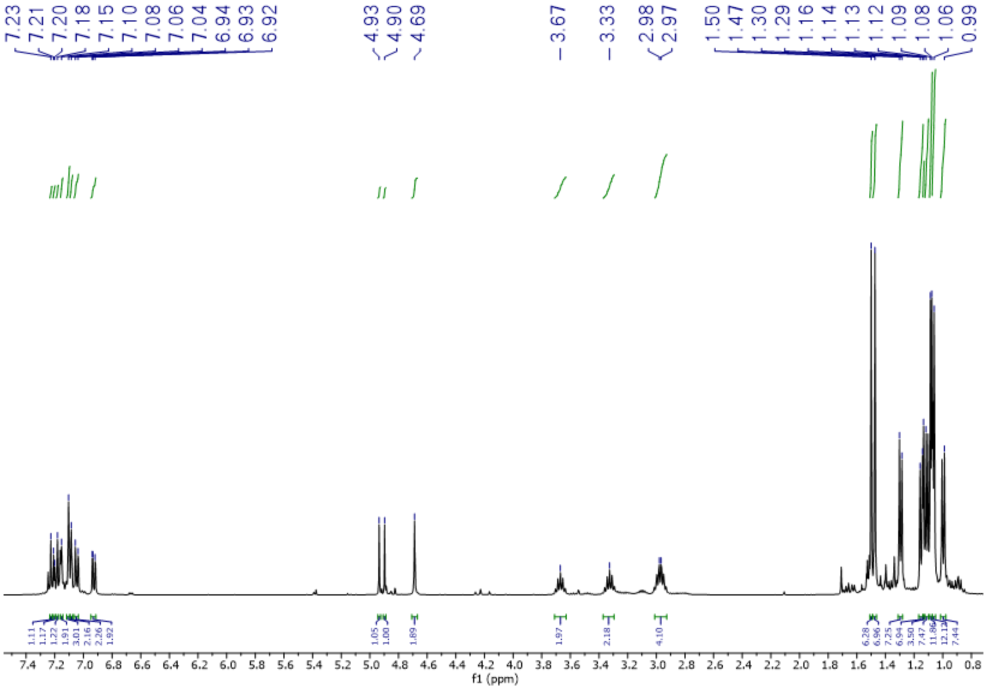
7.23 7.21 7.20 7.18 7.15 7.10 7.08 7.06 7.04 6.94 6.93 6.92
4.93 4.90 4.69
3.67
3.33
2.98 2.97
1.50 1.47 1.30 1.29 1.16 1.14 1.13 1.12 1.09 1.08 1.06 0.99
f1 (ppm)

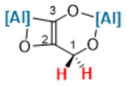
[Al]
[Al]
H H

5f

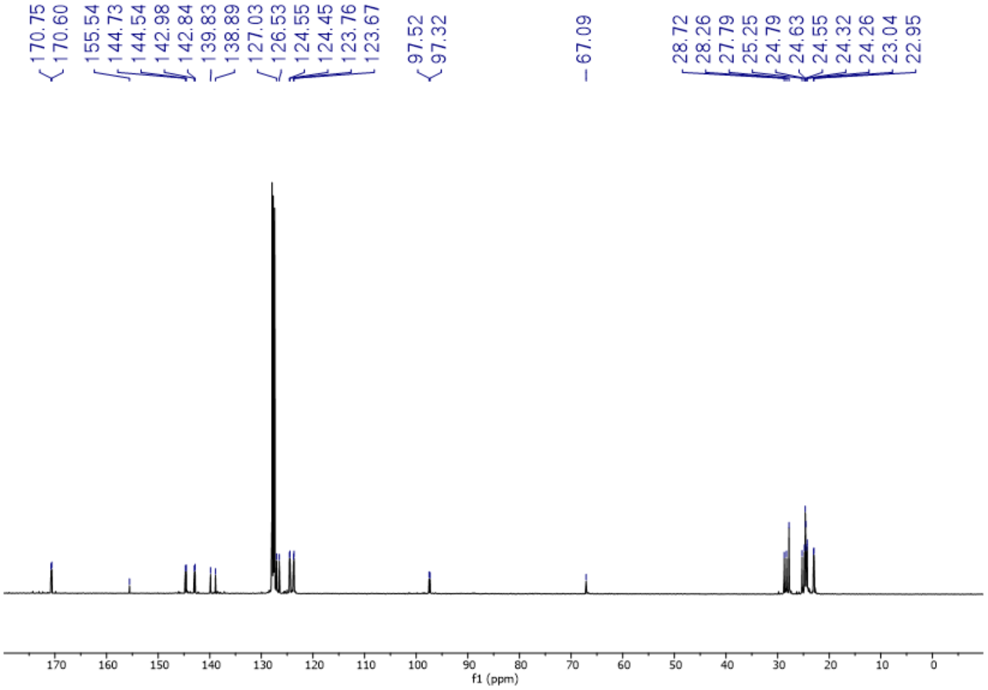
170.75 170.60
155.54 144.73 144.54 142.98 142.84 139.83 138.89
127.03 126.53 124.55 124.45 123.76 123.67
97.52 97.32
67.09
28.72 28.26 27.79 25.25 24.79 24.63 24.55 24.32 24.26 23.04 22.95
f1 (ppm)

6 COMPUTATIONAL COORDINATES

01_3.log

SCF (wB97x) = -4032.58831321

E(SCF)+ZPE(0 K)= -4031.018350

H(298 K)= -4030.919353

G(298 K)= -4031.150263

Lowest Frequency = 16.5812cm-1

W	-3.303679	10.363418	10.738812
Al	-0.365660	13.595587	12.822872
Al	-0.269442	7.998011	14.132813
O	-1.074636	9.147832	13.093285
O	-1.476116	12.510109	11.978806
O	0.604880	9.048177	15.197905
O	0.438768	12.392947	13.800002
C	-4.865266	9.756223	9.597788
N	0.681900	14.573871	11.565428
N	-1.336059	6.771167	15.152473
C	-3.190504	8.493444	11.613189
N	-1.135834	15.080320	13.678128
N	0.723786	6.677843	13.181503
C	-2.120719	9.610813	9.239230
O	-5.768691	9.418343	8.954408
O	-3.228947	13.252413	9.339031
C	-3.260851	12.217578	9.847852
O	-3.161850	7.406516	11.999993
O	-5.586135	11.479256	12.697380
C	-1.666596	11.209272	12.064531
O	-1.556713	9.158067	8.336676
C	0.959097	12.379280	16.703011
H	1.782203	12.802040	16.132301
C	-1.972912	7.194946	16.383895
C	-0.833237	10.492665	13.048799
C	0.549892	13.519368	8.782457
H	-0.193985	14.148869	9.280781
C	0.398325	11.164660	16.298400
C	-1.446745	15.185060	15.088301
C	2.070464	6.932152	12.713412
C	1.019166	16.520312	10.070254
H	1.991819	16.083527	9.844201
H	1.150432	17.563117	10.369195
H	0.414165	16.513037	9.157610
C	-3.333982	7.550253	16.384923
C	2.390260	10.515954	14.811820
C	-2.605109	14.595318	15.614304
C	1.757404	13.360322	9.699511
C	0.101986	11.097048	13.866921
C	-2.905253	14.811165	16.961577
H	-3.797137	14.354986	17.383989
C	2.863981	12.645350	9.232623
H	2.821374	12.191209	8.245386
C	0.235392	5.446731	12.978611
C	1.836926	13.923259	10.986275
C	-2.492079	17.125012	13.415898
H	-3.428280	16.586241	13.595009
H	-2.668795	17.909895	12.679543
H	-2.205119	17.583500	14.365010

C	0.286576	15.775079	11.156900
C	4.641934	10.378592	15.704270
H	5.305457	10.345999	16.564992
C	3.266879	10.469020	15.899210
H	2.868077	10.491215	16.909998
C	-0.587478	15.956031	15.899324
H	0.221394	7.697111	10.973426
C	-0.877697	4.936391	13.651864
H	-1.180380	3.931265	13.387194
C	0.941875	4.511641	12.029617
H	1.067128	4.981227	11.051280
H	0.378791	3.585310	11.912293
H	1.944430	4.272140	12.395601
C	2.946410	5.939839	14.926354
H	1.871739	5.838841	15.110953
C	-1.205177	7.243251	17.566838
C	0.870695	10.415066	15.034474
C	2.926105	10.461422	13.525386
H	2.269107	10.502763	12.660670
C	-0.130539	12.178868	8.508053
H	0.546033	11.485118	7.996482
H	-1.010804	12.321933	7.872649
H	-0.458465	11.714686	9.441290
C	3.004658	13.823185	11.770647
C	0.484748	13.038865	17.831095
H	0.936726	13.979836	18.134501
C	2.300491	7.490807	11.445246
C	-1.445920	16.157008	12.921894
C	3.144037	6.559864	13.548699
C	-1.143060	11.304892	18.165569
H	-1.967826	10.868108	18.724119
C	4.299881	10.354170	13.326771
H	4.686670	10.299790	12.312500
C	3.623642	7.654787	11.025350
H	3.816279	8.076100	10.041194
C	-4.202893	7.513341	15.136981
H	-3.664048	6.983071	14.347531
C	0.707273	16.574816	15.381413
H	0.880569	16.205727	14.365193
C	4.080542	13.104049	11.253795
H	4.986066	13.000554	11.843215
C	4.159658	13.965348	14.057484
H	3.973314	12.904258	14.241536
H	4.112943	14.493385	15.015647
H	5.182680	14.078000	13.680407
C	0.271224	6.866718	17.589366
H	0.696236	7.153650	16.624509
C	-1.530718	5.510371	14.751894
C	-4.722570	11.075704	12.043906
C	-3.532764	13.725266	14.784314
H	-3.208006	13.766198	13.737698
C	0.939261	14.191908	7.457970
H	1.480003	15.131408	7.608767
H	0.042630	14.401926	6.865590
H	1.580844	13.536439	6.858968
C	-0.829392	16.440320	11.708093
H	-1.143663	17.343649	11.198597
C	-0.947448	16.168393	17.229308
H	-0.309679	16.780669	17.863281
C	-0.661919	10.643562	17.037509
H	-1.112574	9.706658	16.732078
C	-3.909152	7.971350	17.586518

H	-4.956401	8.262810	17.598482	C	5.164714	10.313816	14.415341
C	4.689841	7.286783	11.829833	H	6.237905	10.232295	14.261916
H	5.710943	7.419054	11.480919	C	-0.569616	12.504812	18.567883
C	3.452070	16.041662	12.830887	H	-0.940781	13.026448	19.446581
H	4.388993	16.129394	12.269065	C	4.008705	12.505608	10.001447
H	3.571512	16.589767	13.771599	H	4.855650	11.940940	9.619736
H	2.666163	16.538342	12.252986	C	-3.433060	12.269345	15.249826
C	0.457288	5.354301	17.768324	H	-2.418138	11.874427	15.131174
H	-0.008882	5.011913	18.699925	H	-4.119295	11.643715	14.676857
H	1.523357	5.101696	17.813361	H	-3.692609	12.177307	16.311383
H	0.019155	4.786724	16.940139	C	1.904592	16.146080	16.238018
C	1.062239	7.638765	18.649247	C	3.520045	6.849560	16.017143
H	0.877753	8.715101	18.568632	H	4.585776	7.045767	15.853238
H	2.133558	7.466610	18.509215	H	3.415520	6.376815	17.000767
H	0.814223	7.317073	19.667436	H	3.001283	7.812722	16.031333
C	-2.475812	4.609978	15.510055	C	-4.458033	8.931341	14.625637
H	-2.330552	4.699487	16.589088	H	-4.943254	9.543300	15.395205
H	-2.330042	3.570713	15.213697	H	-5.114875	8.908851	13.749850
H	-3.512863	4.886104	15.298248	H	-3.520402	9.418194	14.336507
C	-1.835024	7.646602	18.744664	C	-5.534366	6.786880	15.369392
H	-1.263173	7.691446	19.666218	H	-5.396870	5.799854	15.824446
C	-2.099989	15.605993	17.760992	H	-6.056389	6.654208	14.416348
H	-2.363393	15.780024	18.801127	H	-6.197490	7.360691	16.025994
C	3.130177	14.563340	13.097759	C	-3.174032	8.014063	18.759859
H	2.161217	14.514767	13.609606	H	-3.641594	8.333386	19.687894
C	4.445048	6.751355	13.086199	C	3.557164	4.535050	15.011928
H	5.281729	6.470381	13.721757	H	3.148135	3.863684	14.249371
C	-4.986541	14.210873	14.830371	H	3.350522	4.092357	15.992673
H	-5.407402	14.120677	15.837997	H	4.644893	4.562837	14.882815
H	-5.601864	13.605140	14.158402	C	0.626682	18.105018	15.306613
H	-5.075916	15.260058	14.526992	H	-0.166619	18.438930	14.630331

H 1.572515 18.518506 14.939224
H 0.431855 18.538937 16.293952
C 1.185815 7.909965 10.498182
C 1.246957 7.145570 9.167575
H 2.155132 7.397736 8.608075
H 0.387289 7.413634 8.545675
H 1.239950 6.059719 9.307835
C 1.245728 9.417054 10.226364
H 1.802751 16.489028 17.273533
H 2.832555 16.575064 15.844395
H 2.009537 15.056607 16.252166
H 2.250574 9.733748 9.921868
H 0.959584 9.996922 11.109219
H 0.558133 9.675982 9.421139

02_TS3_4.log

SCF (wB97x) = -4032.54747530
E(SCF)+ZPE(0 K)= -4030.976674
H(298 K)= -4030.878673
G(298 K)= -4031.108696
Lowest Frequency = -138.1393cm-1

W -3.156274 10.168494 11.603204
Al -0.041476 13.695456 12.750103
Al -0.132574 8.234071 14.365771
O -1.114901 9.424642 13.527938
O -1.126302 12.570885 11.914719
O 0.990846 9.246946 15.218046
O 0.655984 12.582964 13.928417
C -4.822679 9.018715 11.427789
N 1.059412 14.562446 11.492453
N -1.143756 6.926363 15.302180
C -2.428903 7.391095 11.776038
N -0.850312 15.210368 13.539313
N 0.894965 7.023788 13.307268
C -2.465845 9.597258 9.758380
O -5.800031 8.396408 11.352789
O -4.531236 12.385322 9.907334
C -4.033759 11.560309 10.572124
O -2.590586 6.431424 11.184370
O -4.262150 11.680823 14.198172
C -1.398448 11.327086 12.231854
O -2.155936 9.371095 8.666692
C -1.760677 7.219767 16.580505
C -0.639315 10.695290 13.296094
C -1.922615 15.061644 14.507944
C 2.185360 7.468472 12.832243
C 2.015033 16.529205 10.348944
H 2.942101 15.979782 10.181174
H 2.245879 17.562681 10.611136
H 1.464507 16.531122 9.403309
C -3.098282 7.661013 16.622637
C -3.255544 14.982931 14.049115
C 1.067078 13.616268 9.219151
C 0.284638 11.311658 14.099729
C -4.262459 14.987716 15.018652
H -5.300368 14.933632 14.711952
C 1.755776 12.940504 8.208556
H 1.273520 12.792995 7.245318
C 0.518429 5.763793 13.091513

C	1.715733	13.789755	10.455645
C	-1.209178	17.641006	13.844328
H	-1.436629	17.484232	14.897682
H	-2.160957	17.797508	13.328158
H	-0.606585	18.544012	13.734631
C	1.164310	15.896522	11.421967
C	-1.593801	15.055454	15.881373
C	-0.583272	5.174420	13.730799
H	-0.813290	4.156132	13.440235
C	1.329704	4.856209	12.199566
H	1.999113	4.249444	12.818997
H	1.939065	5.404435	11.481418
H	0.664089	4.172856	11.666874
C	-1.007528	7.007207	17.752153
C	0.868176	10.643857	15.364792
C	2.998602	13.258896	10.707573
C	2.254342	8.284054	11.686403
C	-0.497344	16.463162	13.221074
C	3.339521	7.116559	13.557215
C	3.512074	8.731223	11.276007
H	3.590750	9.369220	10.400042
C	3.643825	12.606716	9.655291
H	4.636244	12.195918	9.807448
C	-1.269876	5.670793	14.846895
C	-3.885105	11.058160	13.299098
C	0.484236	16.775703	12.271144
H	0.675881	17.829972	12.115601
C	-2.648093	15.053148	16.796577
H	-2.437477	15.047791	17.860417
C	-3.698914	7.772674	17.879011
H	-4.731312	8.097733	17.951628
C	4.663862	8.385956	11.971899
H	5.632092	8.747104	11.634210
C	-2.123480	4.665820	15.588990
H	-1.538850	4.221678	16.400692
H	-2.426098	3.864913	14.911843
H	-3.009233	5.111669	16.037810
C	-1.662006	7.132043	18.978804
H	-1.112822	6.961966	19.899868
C	-3.967839	15.042190	16.371759
H	-4.773408	15.049447	17.101414
C	4.573447	7.585743	13.101032
H	5.476562	7.326187	13.649125
C	3.037622	12.458321	8.413725
H	3.563199	11.947439	7.610946
C	-3.000860	7.486003	19.043169
H	-3.495900	7.569920	20.007286
C	1.011437	8.643375	10.880359
H	0.166906	8.715976	11.574238
C	0.488537	6.692461	17.711013
H	0.911196	7.267647	16.878528
C	-3.876837	8.035691	15.359055
H	-3.175574	8.562158	14.697690
C	3.671446	13.370344	12.077248
H	2.911935	13.143021	12.838693
C	-0.347893	14.107622	8.930151
H	-0.716218	14.650263	9.808423
C	-0.146403	15.026193	16.386594
H	0.397662	14.325544	15.739636
C	-3.631319	14.833453	12.566509
H	-3.008324	14.025978	12.157709
C	3.301531	6.278744	14.830153

H	2.272306	5.941395	14.993824
C	3.706289	7.115149	16.051387
H	4.722963	7.509935	15.938572
H	3.029544	7.963646	16.191968
H	3.690198	6.495701	16.956142
C	4.188704	5.031792	14.720799
H	5.249544	5.301010	14.669839
H	4.054227	4.393097	15.600560
H	3.957436	4.439181	13.829603
C	1.132887	9.991907	10.169099
H	0.164693	10.282651	9.752528
H	1.463601	10.784058	10.849949
H	1.839702	9.951750	9.333048
C	0.679488	7.544679	9.859363
H	1.536846	7.358604	9.201412
H	0.410063	6.600036	10.341560
H	-0.164997	7.850929	9.233825
C	0.800247	5.206865	17.474566
H	1.875091	5.026087	17.593230
H	0.276762	4.579647	18.206505
H	0.526701	4.871865	16.470526
C	1.219814	7.153291	18.977086
H	1.003390	6.501468	19.831685
H	2.301179	7.119636	18.809555
H	0.948736	8.176773	19.249726
C	-5.036482	8.990905	15.656988
H	-4.710104	9.867562	16.224917
H	-5.475065	9.337358	14.717369
H	-5.835775	8.489306	16.214957
C	-4.437986	6.836179	14.579053
H	-3.660251	6.209387	14.137084
H	-5.062980	6.206774	15.224719
H	-5.066790	7.196115	13.757435
C	-3.388521	16.080689	11.701239
H	-3.778913	15.895926	10.695127
H	-3.924084	16.948648	12.106044
H	-2.332802	16.339053	11.590316
C	-5.092791	14.414273	12.367277
H	-5.256457	14.171342	11.315373
H	-5.354774	13.531595	12.956029
H	-5.779318	15.226845	12.635367
C	-0.040840	14.507123	17.826480
H	1.012950	14.382045	18.096258
H	-0.459105	15.226281	18.540284
H	-0.546000	13.546930	17.965422
C	0.580356	16.380160	16.325225
H	0.774898	16.723837	15.307483
H	0.010445	17.155109	16.852026
H	1.552823	16.291431	16.821392
C	-1.294729	12.924682	8.689801
H	-2.320149	13.278024	8.538762
H	-0.997860	12.356445	7.800372
H	-1.301928	12.250230	9.548483
C	-0.396849	15.065374	7.731703
H	0.269157	15.926506	7.849564
H	-0.112247	14.554548	6.805355
H	-1.415455	15.443248	7.597739
C	4.800855	12.354270	12.260897
H	5.649921	12.572365	11.601841
H	5.161997	12.402320	13.290089
H	4.464312	11.328716	12.075096
C	4.225973	14.772146	12.373754

H	4.937981	15.080312	11.598219
H	3.446645	15.533948	12.449742
H	4.757740	14.757637	13.330947
C	-0.090451	10.903866	16.547583
C	-1.396751	11.365069	16.383631
C	0.330276	10.540053	17.831845
C	-2.267516	11.450659	17.470888
H	-1.763264	11.660481	15.405009
C	-0.532068	10.629531	18.916390
H	1.346436	10.178121	17.971653
C	-1.840677	11.079318	18.739034
H	-3.277549	11.820436	17.309532
H	-0.186200	10.340605	19.906395
H	-2.518402	11.137967	19.586667
C	2.280606	11.192889	15.625584
C	3.376670	10.512007	15.097736
C	2.509564	12.341532	16.381386
C	4.673774	10.942751	15.361335
H	3.208984	9.625920	14.495260
C	3.803855	12.788987	16.627879
H	1.666487	12.875370	16.803904
C	4.895079	12.082490	16.128850
H	5.512907	10.382741	14.953337
H	3.958908	13.682440	17.228510
H	5.908129	12.417038	16.338567

03_4.log

SCF (wB97x) = -3919.27240046

E(SCF)+ZPE(0 K)= -3917.709899

H(298 K)= -3917.613759

G(298 K)= -3917.838153

Lowest Frequency = 17.4664cm-1

W	8.544925	2.873334	7.443985
Al	11.083449	1.306542	4.838230
Al	11.301114	6.768475	6.284207
O	10.053469	2.552179	5.575473
O	10.088176	5.733964	7.070079
O	12.154580	2.280251	3.894006
C	9.841296	4.478770	6.782356
O	11.893833	5.667400	5.015832
C	7.586513	3.547647	9.022114
O	7.049275	3.976648	9.963891
N	9.938346	-0.079795	4.236156
C	7.604233	1.081170	7.367064
O	5.914394	4.133830	6.157834
N	10.794947	8.378867	5.446641
O	10.225553	1.627016	9.851775
N	12.270322	0.240168	5.884355
N	12.278980	7.545965	7.735024
C	9.728243	2.062839	8.897988
C	10.527225	3.858749	5.657201
O	7.089233	0.044270	7.257192
C	11.453596	4.411781	4.818615
C	9.266514	-0.123616	2.949493
C	12.646246	-1.613406	7.465136
H	13.671975	-1.723980	7.104422
H	12.224757	-2.598888	7.665334
H	12.691495	-1.057219	8.407030
C	12.834702	9.400660	9.272682
H	12.301875	9.125996	10.186706

H	12.865553	10.488980	9.209906
H	13.852087	9.014993	9.360170
C	9.697850	-1.086120	5.098132
C	13.428026	4.116604	3.202411
C	11.329095	9.743189	7.353867
H	11.243380	10.735835	7.777225
C	12.014771	3.645758	3.585467
C	13.654072	0.630013	6.089287
C	10.525012	-1.389716	6.187906
H	10.205503	-2.217655	6.808355
C	12.808421	3.213673	8.268388
H	13.738369	3.756327	8.461878
H	12.071985	3.524013	9.013302
H	12.445446	3.506636	7.278518
C	11.800001	-0.864495	6.468186
C	11.045091	3.799587	2.399846
C	12.124429	8.833294	8.067629
C	14.224476	-0.363151	3.793194
H	13.190444	-0.092950	3.560146
C	8.477775	-1.952209	4.912691
H	7.604803	-1.383528	5.253294
H	8.549241	-2.857170	5.517077
H	8.315431	-2.223844	3.868606
C	10.759658	9.552221	6.097884
C	14.600490	0.352136	5.081117
C	8.067099	0.572201	2.722595
C	9.990004	2.799825	0.458252
H	9.835540	1.927928	-0.172579
C	7.694070	6.373845	4.265913
H	7.384971	6.176312	3.232094
H	6.866839	6.099297	4.927021
H	8.535226	5.706273	4.484544
C	11.346822	8.813669	1.799750
H	12.162452	9.037899	1.116233
C	7.442696	0.431549	1.479810
H	6.511511	0.962399	1.298491
C	14.035223	1.306928	7.259950
C	10.390535	5.000077	2.118205
H	10.550147	5.870734	2.751174
C	13.048194	1.704696	8.339599
H	12.097008	1.203803	8.142835
C	10.529894	8.426218	4.025865
C	11.603692	8.733315	3.168404
C	7.975443	-0.368377	0.483632
H	7.463903	-0.473595	-0.469621
C	9.838874	-0.920136	1.933646
C	13.725769	4.712999	1.979285
H	12.927323	4.961012	1.286750
C	10.849498	2.710914	1.548580
H	11.376289	1.783656	1.750028
C	12.641269	6.336182	9.866029
C	14.444560	6.486727	8.196226
C	6.922280	3.677817	6.506854
C	15.290686	5.827344	9.088719
H	16.316810	5.620375	8.801892
C	14.479499	3.801588	4.069223
H	14.264674	3.310245	5.016122
C	9.172229	-1.032521	0.713508
H	9.593407	-1.660619	-0.067595
C	7.410777	1.480435	3.747709
H	8.006785	1.459073	4.664102
C	13.123855	6.754425	8.610462

C	10.136124	10.752278	5.433168
H	10.702081	11.039961	4.542205
H	10.105691	11.598340	6.120330
H	9.119829	10.521490	5.101481
C	13.526578	5.659648	10.709822
H	13.175460	5.320609	11.681034
C	16.317207	1.395972	6.444092
H	17.355317	1.688173	6.582418
C	11.137892	-1.692423	2.122315
H	11.608654	-1.344804	3.046410
C	9.534532	5.090692	1.022967
H	9.026768	6.029124	0.821889
C	8.070380	7.848267	4.452819
H	8.398466	7.977415	5.490451
C	14.839806	5.420336	10.338596
H	15.513842	4.906857	11.019463
C	9.229869	8.217436	3.539963
C	11.217922	6.586503	10.358874
H	10.725075	7.276002	9.664989
C	9.324611	3.991902	0.194860
H	8.646592	4.067106	-0.651434
C	13.523257	10.382173	3.265195
H	13.627751	10.461354	2.177581
H	14.508238	10.570181	3.706039
H	12.849094	11.180510	3.594340
C	5.992338	1.014284	4.099011
H	5.321494	1.087699	3.235242
H	5.577218	1.642014	4.894051
H	5.978225	-0.023552	4.447417
C	13.015967	8.995883	3.681054
H	12.994302	8.976911	4.776179

C	15.795885	4.061234	3.709810
H	16.599105	3.780080	4.386869
C	10.394929	5.292955	10.370023
H	10.292469	4.875962	9.367084
H	9.384797	5.484973	10.747177
H	10.857124	4.538373	11.018565
C	15.926131	0.731531	5.289427
H	16.666449	0.520360	4.524175
C	13.506000	1.308925	9.748158
H	14.362545	1.911085	10.072716
H	13.800696	0.255228	9.807342
H	12.691436	1.478672	10.458159
C	15.046522	4.986500	1.623346
H	15.257744	5.445965	0.660771
C	14.297437	-1.884099	3.985257
H	15.303344	-2.182280	4.303072
H	14.071907	-2.410242	3.051026
H	13.586028	-2.232756	4.742389
C	15.371850	1.688168	7.412615
H	15.666837	2.225227	8.311186
C	7.398435	2.926027	3.234089
H	8.414580	3.313220	3.115334
H	6.859321	3.575533	3.927502
H	6.900845	2.995410	2.259780
C	15.070352	0.083753	2.597259
H	15.085258	1.175601	2.514460
H	14.653177	-0.324327	1.672078
H	16.103709	-0.274556	2.667189
C	12.115359	-1.431817	0.970551
H	11.724500	-1.800340	0.016401
H	13.064378	-1.947865	1.149383

H	12.323670	-0.362187	0.856583
C	13.981641	7.898845	3.223808
H	13.657105	6.914649	3.573606
H	14.991793	8.089713	3.606310
H	14.045813	7.856720	2.130501
C	16.086283	4.658477	2.483453
H	17.117225	4.854577	2.199491
C	11.191217	7.215192	11.760768
H	11.502462	6.493154	12.523255
H	10.171255	7.524897	12.008606
H	11.845934	8.088170	11.854358
C	9.019528	8.336705	2.164647
H	8.017852	8.190667	1.766647
C	10.064938	8.627887	1.299258
H	9.881395	8.707316	0.230860
C	15.306488	8.419523	6.789758
H	14.438647	9.062508	6.962256
H	15.723460	8.685300	5.811703
H	16.061557	8.652920	7.549844
C	10.880199	-3.196693	2.279553
H	10.241369	-3.409190	3.142821
H	11.824400	-3.734470	2.421515
H	10.388801	-3.605661	1.389165
C	6.846393	8.746268	4.238923
H	6.406402	8.597499	3.246846
H	7.093205	9.809437	4.336507
H	6.074483	8.507528	4.976984
C	16.165285	6.111828	6.357248
H	17.069182	6.373377	6.920202
H	16.359742	6.318725	5.300674
H	15.991009	5.035639	6.459111
C	14.956895	6.924345	6.826258
H	14.156944	6.741074	6.095105

04_TS1.log

SCF (wB97x) = -3920.42475443

E(SCF)+ZPE(0 K)= -3918.848881

H(298 K)= -3918.751104

G(298 K)= -3918.978608

Lowest Frequency = -216.1987cm-1

W	8.188941	3.171387	7.788275
Al	11.142636	1.161125	4.743859
Al	11.324612	6.603999	6.275668
O	10.086126	2.363724	5.462400
O	10.098608	5.554850	7.011049
O	12.221081	2.145046	3.819656
C	9.779220	4.311988	6.719008
O	11.910058	5.491637	5.018916
C	7.651259	4.647050	8.930563
O	7.335905	5.550399	9.600721
N	10.041898	-0.253707	4.113808
C	6.778512	1.972651	8.609294
O	5.715927	4.128096	6.006256
N	10.792090	8.211848	5.438479
O	9.987199	2.088216	10.206459
N	12.310125	0.094856	5.815121
N	12.351487	7.407238	7.690372
C	9.405705	2.487502	9.287879
C	10.500070	3.664753	5.625422
O	5.962497	1.296689	9.084127

C	11.449973	4.246739	4.814238
C	9.369012	-0.263890	2.828865
C	12.730513	-1.776646	7.359572
H	13.766841	-1.850928	7.020635
H	12.338182	-2.777114	7.544540
H	12.739275	-1.224679	8.304463
C	12.972522	9.305973	9.157670
H	12.457627	9.108752	10.101813
H	13.038734	10.387042	9.028683
H	13.978328	8.891486	9.238890
C	9.834725	-1.312061	4.917121
C	13.417617	4.024799	3.152973
C	11.361210	9.590945	7.323698
H	11.279066	10.585573	7.743255
C	12.022582	3.514640	3.556988
C	13.661124	0.542178	6.090709
C	10.648471	-1.633681	6.008597
H	10.359830	-2.506740	6.580363
C	12.708447	3.148625	8.263813
H	13.629545	3.647841	8.577285
H	11.910009	3.456449	8.942226
H	12.462745	3.494091	7.255519
C	11.881220	-1.047765	6.350566
C	11.033930	3.672367	2.386949
C	12.202338	8.700876	8.007196
C	14.366438	-0.405066	3.812197
H	13.348123	-0.126830	3.523349
C	8.667492	-2.227746	4.643808
H	7.735570	-1.658986	4.726302
H	8.643702	-3.050177	5.359249
H	8.700343	-2.634188	3.630407
C	10.755144	9.386265	6.087584
C	14.665711	0.294617	5.131202
C	8.144589	0.399835	2.639863
C	9.996740	2.702552	0.420065
H	9.862915	1.845487	-0.235184
C	7.654661	6.241272	4.224089
H	7.364584	6.063160	3.181296
H	6.805533	5.979397	4.860444
H	8.476418	5.554214	4.455035
C	11.333273	8.678155	1.793794
H	12.148462	8.908246	1.111632
C	7.515154	0.282980	1.397450
H	6.564489	0.787074	1.241468
C	13.948461	1.238650	7.275687
C	10.351455	4.864536	2.138725
H	10.494513	5.722054	2.792775
C	12.889263	1.628528	8.293529
H	11.935970	1.167170	8.009636
C	10.520737	8.266723	4.018468
C	11.592787	8.580450	3.160788
C	8.071860	-0.456347	0.366794
H	7.557348	-0.542200	-0.586810
C	9.968183	-0.992556	1.778962
C	13.682567	4.621575	1.922572
H	12.866181	4.868579	1.251038
C	10.860153	2.604170	1.506409
H	11.409330	1.685455	1.682427
C	12.831033	6.283736	9.841888
C	14.536499	6.339816	8.069734
C	6.671910	3.783879	6.561977
C	15.408654	5.666432	8.926969

H	16.412227	5.426731	8.591331
C	14.493180	3.712765	3.991193
H	14.303961	3.221250	4.943497
C	9.297876	-1.079082	0.558734
H	9.740743	-1.652352	-0.252686
C	7.481429	1.260033	3.704094
H	8.094355	1.227106	4.607238
C	13.243463	6.641403	8.543285
C	10.095664	10.576578	5.439729
H	10.623090	10.865644	4.526076
H	10.081893	11.425669	6.123683
H	9.069736	10.333238	5.150012
C	13.739942	5.597186	10.650898
H	13.437596	5.300899	11.651812
C	16.270907	1.384348	6.589887
H	17.290984	1.705026	6.786786
C	11.307346	-1.706012	1.926328
H	11.728871	-1.444520	2.901716
C	9.489810	4.965140	1.048868
H	8.960914	5.897089	0.874211
C	8.055225	7.704710	4.440620
H	8.385629	7.807143	5.480535
C	15.016958	5.295007	10.207031
H	15.711709	4.770806	10.858339
C	9.218303	8.073192	3.532841
C	11.456164	6.609550	10.416937
H	10.959534	7.330413	9.759497
C	9.302828	3.885246	0.190961
H	8.621598	3.968421	-0.652031
C	13.483464	10.255237	3.307126
H	13.567367	10.382168	2.222183

H	14.472458	10.441409	3.739944
H	12.801802	11.026737	3.681639
C	6.076946	0.762066	4.066656
H	5.388797	0.843595	3.217491
H	5.666105	1.366270	4.882608
H	6.079315	-0.284746	4.390239
C	13.005725	8.843838	3.670785
H	12.995373	8.780244	4.764058
C	15.799138	3.968508	3.593832
H	16.621354	3.686707	4.247508
C	10.571306	5.362815	10.454717
H	10.388772	4.990024	9.444674
H	9.598186	5.596586	10.897331
H	11.037927	4.565905	11.046043
C	15.965880	0.710963	5.413367
H	16.753881	0.525232	4.690145
C	13.235258	1.175172	9.717799
H	14.104325	1.720353	10.103673
H	13.467630	0.106092	9.772421
H	12.392174	1.378432	10.384466
C	14.993198	4.894383	1.529606
H	15.177275	5.353667	0.561374
C	14.424769	-1.930753	3.966931
H	15.409682	-2.243850	4.332884
H	14.253423	-2.424857	3.003369
H	13.668233	-2.302024	4.666869
C	15.264744	1.655520	7.501782
H	15.494898	2.208273	8.409897
C	7.434312	2.721473	3.241768
H	8.441305	3.121749	3.097962
H	6.920125	3.339607	3.981791

H	6.895083	2.820780	2.292298
C	15.280400	0.059927	2.674719
H	15.297935	1.152458	2.605331
H	14.917856	-0.336755	1.721707
H	16.308409	-0.299153	2.800463
C	12.302122	-1.251194	0.851813
H	11.942862	-1.487980	-0.155405
H	13.263039	-1.760749	0.982848
H	12.481372	-0.171492	0.898910
C	13.984684	7.783587	3.160658
H	13.687113	6.783387	3.487480
H	14.998091	7.983779	3.528759
H	14.028635	7.774744	2.065663
C	16.056510	4.563513	2.358678
H	17.079521	4.755773	2.044962
C	11.541848	7.227808	11.820281
H	11.842275	6.482889	12.564806
H	10.558041	7.602102	12.119659
H	12.257693	8.054973	11.879798
C	9.005274	8.211113	2.159587
H	8.001033	8.078043	1.763390
C	10.049509	8.505276	1.294173
H	9.863263	8.598649	0.227406
C	15.337538	8.277136	6.644292
H	14.464823	8.911529	6.822012
H	15.745239	8.546813	5.663685
H	16.095640	8.519046	7.398703
C	11.145813	-3.231386	1.891820
H	10.492062	-3.588993	2.693724
H	12.118681	-3.720892	2.012524
H	10.719219	-3.562346	0.938070
C	6.843389	8.623493	4.243523
H	6.395940	8.491148	3.252376
H	7.104415	9.682299	4.350051
H	6.072568	8.387864	4.983671
C	16.220154	5.987640	6.192062
H	17.128829	6.267738	6.738304
H	16.390072	6.198851	5.132423
H	16.069268	4.908508	6.296893
C	15.006636	6.777027	6.684962
H	14.193214	6.581142	5.972226
H	8.385686	0.614801	7.106122
H	8.888638	0.878254	6.614252

05_Int1.log

SCF (wB97x) = -3920.43862282

E(SCF)+ZPE(0 K)= -3918.861300

H(298 K)= -3918.763625

G(298 K)= -3918.992330

Lowest Frequency = 13.8683cm-1

W	-2.667586	-0.415828	-2.680474
Al	0.102800	2.866494	-0.463937
Al	0.247871	-2.748646	0.782342
O	-0.563675	-1.587480	-0.237865
O	-0.982023	1.778981	-1.334974
O	1.129482	-1.702565	1.848113
O	0.918417	1.664027	0.505223
C	-4.036883	-1.104442	-4.005058
N	1.161262	3.880530	-1.684736
N	-0.809397	-3.987825	1.785470

C	-4.161472	0.226473	-1.523364	C	2.341171	3.265229	-2.251422
N	-0.704793	4.327756	0.397880	C	-2.079953	6.359940	0.149058
N	1.255775	-4.057562	-0.181930	H	-3.017768	5.809602	0.278129
C	-2.608823	-2.283634	-1.812087	H	-2.237903	7.168105	-0.566008
O	-4.831790	-1.488320	-4.758340	H	-1.828143	6.786846	1.122418
O	-5.046319	0.615075	-0.877200	C	0.757458	5.085090	-2.079198
O	-2.663471	2.454163	-4.105557	C	5.169067	-0.343392	2.270644
O	-2.618066	-3.365603	-1.404533	H	5.855997	-0.382886	3.112542
C	1.500395	1.578802	3.432965	C	3.798871	-0.272408	2.503497
H	2.354515	1.991436	2.901778	H	3.426400	-0.274558	3.524504
C	-1.451901	-3.560115	3.011125	C	-0.234809	5.134163	2.663155
C	-0.327525	-0.240618	-0.276598	H	0.795420	-2.216905	-2.054457
C	1.099484	2.850727	-4.478664	C	-0.286721	-5.842470	0.335342
H	0.320076	3.432676	-3.976752	H	-0.545232	-6.867199	0.099407
C	0.910348	0.399055	2.972823	C	1.611161	-6.298699	-1.168366
C	-1.060779	4.384692	1.799817	H	2.089863	-5.827740	-2.026968
C	2.577240	-3.762630	-0.692592	H	0.954686	-7.100438	-1.511718
C	1.499155	5.857902	-3.140484	H	2.401258	-6.748847	-0.557896
H	2.485216	5.444064	-3.350908	C	3.578886	-4.814259	1.435626
H	1.602869	6.899355	-2.826742	H	2.529239	-5.073561	1.614520
H	0.913736	5.849923	-4.065787	C	-0.684839	-3.508278	4.193824
C	-2.812622	-3.205992	3.005051	C	1.380888	-0.330395	1.696924
C	2.893380	-0.215456	1.440832	C	3.394051	-0.247837	0.138764
C	-2.228188	3.763284	2.266186	H	2.708494	-0.211528	-0.706715
C	2.300614	2.723466	-3.549041	C	0.491061	1.483444	-4.789211
C	0.596882	0.362870	0.549837	H	1.206680	0.845199	-5.319861
C	-2.567070	3.917738	3.612525	H	-0.398638	1.595926	-5.416981
H	-3.466100	3.436424	3.989692	H	0.193657	0.978542	-3.866557
C	3.437144	2.054723	-4.013056	C	3.498084	3.182168	-1.449420
H	3.425146	1.620188	-5.009994	C	1.012709	2.220891	4.566453
C	0.827305	-5.317916	-0.327767	H	1.490184	3.133024	4.915639

C	2.722520	-3.127023	-1.942035
C	-1.012589	5.414265	-0.341693
C	3.694573	-4.163850	0.062199
C	-0.693047	0.545398	4.787471
H	-1.555273	0.127047	5.301429
C	4.763233	-0.334689	-0.097699
H	5.124301	-0.368936	-1.122082
C	4.017383	-2.945618	-2.432063
H	4.164390	-2.470280	-3.396225
C	-3.674156	-3.253089	1.751912
H	-3.130311	-3.791438	0.971012
C	1.056399	5.803049	2.200973
H	1.255289	5.489419	1.170702
C	4.605575	2.510877	-1.964090
H	5.503478	2.421216	-1.360982
C	4.595583	3.274662	0.864892
H	4.429543	2.202842	1.002619
H	4.510950	3.763666	1.841080
H	5.625372	3.425191	0.520900
C	0.790183	-3.890638	4.218738
H	1.206486	-3.662164	3.235082
C	-0.976816	-5.258796	1.404600
C	-2.651311	1.417652	-3.593367
C	-3.120218	2.923257	1.369369
H	-2.760612	3.009734	0.337471
C	1.471836	3.565504	-5.785631
H	1.966770	4.525631	-5.611175
H	0.573103	3.745420	-6.384629
H	2.150471	2.951974	-6.388436
C	-0.374483	5.727964	-1.537872
H	-0.687832	6.638302	-2.035211
C	-0.630970	5.281709	3.991623
H	-0.018102	5.873250	4.668480
C	-0.197684	-0.099821	3.657174
H	-0.677286	-1.006667	3.306598
C	-3.389414	-2.779408	4.204076
H	-4.436749	-2.488099	4.213578
C	5.131092	-3.350892	-1.708192
H	6.128231	-3.195307	-2.112413
C	3.876201	5.376901	-0.313824
H	4.824883	5.500498	-0.848589
H	3.960150	5.898580	0.645576
H	3.094404	5.874475	-0.896797
C	0.973386	-5.392536	4.472289
H	0.527300	-5.685132	5.430285
H	2.039511	-5.647406	4.505167
H	0.513148	-5.998585	3.684237
C	1.596354	-3.066254	5.226000
H	1.419999	-1.995231	5.083184
H	2.665316	-3.256931	5.089107
H	1.352602	-3.325495	6.262760
C	-1.929122	-6.155512	2.157661
H	-1.796804	-6.059374	3.237823
H	-1.780849	-7.196687	1.869341
H	-2.963389	-5.878985	1.932561
C	-1.316042	-3.099889	5.368858
H	-0.745644	-3.051609	6.291472
C	-1.789636	4.682553	4.467296
H	-2.081019	4.806711	5.507151
C	3.577553	3.885446	-0.099284
H	2.598319	3.803015	0.387657
C	4.967249	-3.938190	-0.465147

H	5.841943	-4.232689	0.109605
C	-4.580078	3.391413	1.381974
H	-5.035193	3.259301	2.370056
H	-5.161072	2.801741	0.666087
H	-4.673002	4.449832	1.113394
C	5.657355	-0.379138	0.967095
H	6.726964	-0.443712	0.783388
C	-0.085750	1.706717	5.249667
H	-0.467412	2.215118	6.131537
C	4.574858	1.938695	-3.229992
H	5.446762	1.412102	-3.609859
C	-3.026993	1.449047	1.770937
H	-2.001552	1.070771	1.694999
H	-3.669169	0.848349	1.125036
H	-3.349982	1.302627	2.809147
C	2.249023	5.358181	3.055220
C	4.000976	-3.822184	2.525064
H	5.027992	-3.475463	2.360885
H	3.958018	-4.299118	3.511786
H	3.349488	-2.943102	2.528525
C	-3.922917	-1.839827	1.224877
H	-4.412722	-1.220477	1.985393
H	-4.571058	-1.864926	0.342871
H	-2.982033	-1.358364	0.938393
C	-5.006628	-3.977262	1.983979
H	-4.871117	-4.962503	2.443970
H	-5.525742	-4.113505	1.029940
H	-5.671235	-3.399565	2.635719
C	-2.655314	-2.732055	5.378573
H	-3.124784	-2.408443	6.304224
C	4.395912	-6.108088	1.544035
H	4.161251	-6.815624	0.741589
H	4.191147	-6.600782	2.500579
H	5.472349	-5.908865	1.503783
C	0.943116	7.333151	2.202160
H	0.150198	7.684417	1.534315
H	1.883819	7.784840	1.868094
H	0.727546	7.712046	3.207648
C	1.516942	-2.658689	-2.754381
C	0.797344	-3.809060	-3.474700
H	1.489875	-4.360648	-4.122497
H	-0.005074	-3.409744	-4.105325
H	0.334755	-4.511637	-2.777855
C	1.888922	-1.585702	-3.779362
H	2.118477	5.638930	4.106242
H	3.171728	5.833542	2.704578
H	2.381741	4.272965	3.009382
H	2.523068	-1.989602	-4.577620
H	2.409495	-0.734119	-3.324849
H	0.984784	-1.208438	-4.261410
H	-1.522983	-1.107216	-4.071589
C	-1.152873	0.474024	-1.268378
H	-0.983881	-0.899350	-3.485634

06_TS2.log

SCF (wB97x) = -3920.42594079

E(SCF)+ZPE(0 K)= -3918.851583

H(298 K)= -3918.754417

G(298 K)= -3918.981261

Lowest Frequency = -676.2239cm-1

W	-2.630041	-0.439863	-2.708578	C	-2.847888	-3.221136	2.967462
Al	0.097100	2.861611	-0.454304	C	2.890157	-0.205352	1.447527
Al	0.230665	-2.753226	0.799462	C	-2.190461	3.779048	2.319738
O	-0.629960	-1.578302	-0.164051	C	2.307403	2.713251	-3.564591
O	-1.034649	1.777946	-1.279732	C	0.586618	0.359155	0.566329
O	1.137599	-1.708908	1.849988	C	-2.512521	3.958790	3.667183
O	0.923978	1.653482	0.497533	H	-3.402509	3.478390	4.066218
C	-4.123290	-1.113587	-3.925967	C	3.455772	2.054775	-4.015277
N	1.138730	3.859077	-1.703161	H	3.458118	1.621943	-5.012750
N	-0.825139	-4.003899	1.785579	C	0.826518	-5.315345	-0.323274
C	-4.111788	0.305000	-1.470139	C	2.328739	3.253531	-2.265305
N	-0.699429	4.322896	0.416209	C	-2.089888	6.344888	0.158659
N	1.239640	-4.051141	-0.174556	H	-3.022191	5.791052	0.310214
C	-2.485153	-2.324011	-1.884696	H	-2.263824	7.141390	-0.565707
O	-4.952709	-1.504094	-4.629942	H	-1.825927	6.787993	1.121530
O	-4.952718	0.754403	-0.819595	C	0.725906	5.060838	-2.094306
O	-2.479672	2.369262	-4.235425	C	5.168150	-0.290046	2.275300
O	-2.425512	-3.417409	-1.515870	H	5.856872	-0.308276	3.116446
C	1.469207	1.600004	3.413466	C	3.797362	-0.233956	2.509480
H	2.297922	2.031864	2.857747	H	3.426555	-0.225361	3.530998
C	-1.488277	-3.577762	2.998638	C	-0.202856	5.170112	2.662207
C	-0.365008	-0.241220	-0.231483	H	0.740923	-2.199775	-2.041777
C	1.126780	2.837851	-4.521024	C	-0.281359	-5.854706	0.339520
H	0.319161	3.384944	-4.023078	H	-0.528777	-6.881754	0.102030
C	0.910335	0.392780	2.984650	C	1.621433	-6.283803	-1.167171
C	-1.034566	4.399297	1.822917	H	2.076326	-5.806765	-2.035330
C	2.552123	-3.738676	-0.697592	H	0.979381	-7.102772	-1.496536
C	1.456167	5.840098	-3.157657	H	2.430705	-6.710355	-0.564766
H	2.444455	5.433728	-3.371714	C	3.582282	-4.776051	1.422790
H	1.552487	6.882064	-2.843294	H	2.539614	-5.063479	1.600238
H	0.866996	5.827060	-4.080370	C	-0.740297	-3.527350	4.193761

C	1.379551	-0.334600	1.706813
C	3.388459	-0.249386	0.144884
H	2.701954	-0.236155	-0.700512
C	0.564883	1.467734	-4.900694
H	1.319836	0.858729	-5.410805
H	-0.290243	1.581657	-5.573877
H	0.224495	0.921985	-4.017973
C	3.480678	3.182765	-1.453875
C	0.982402	2.244179	4.545757
H	1.433525	3.178822	4.869359
C	2.672766	-3.105582	-1.950540
C	-1.023886	5.398720	-0.334568
C	3.680761	-4.123896	0.048294
C	-0.657146	0.513179	4.832130
H	-1.492547	0.072920	5.371660
C	4.758280	-0.318984	-0.092458
H	5.117887	-0.361939	-1.116887
C	3.960207	-2.908421	-2.454351
H	4.089057	-2.433988	-3.421646
C	-3.687480	-3.276070	1.698883
H	-3.122244	-3.805293	0.926109
C	1.082522	5.831862	2.175382
H	1.277354	5.490855	1.153259
C	4.598955	2.520685	-1.956245
H	5.491865	2.440626	-1.344622
C	4.561626	3.292613	0.867303
H	4.391901	2.222475	1.013010
H	4.472848	3.790280	1.838583
H	5.593857	3.437298	0.528607
C	0.735287	-3.908942	4.236075
H	1.168600	-3.652011	3.266422

C	-0.983417	-5.275938	1.401325
C	-2.537048	1.358516	-3.677266
C	-3.095112	2.916281	1.457460
H	-2.749569	2.975427	0.418848
C	1.521078	3.600881	-5.795242
H	2.000160	4.561230	-5.583270
H	0.634329	3.786474	-6.410196
H	2.221156	3.012778	-6.398930
C	-0.405931	5.699709	-1.543405
H	-0.731909	6.602416	-2.046539
C	-0.583743	5.344216	3.992005
H	0.033376	5.954512	4.647860
C	-0.163435	-0.133198	3.701293
H	-0.617118	-1.061790	3.375257
C	-3.444189	-2.792121	4.155963
H	-4.491097	-2.498807	4.148162
C	5.086352	-3.297529	-1.740398
H	6.077207	-3.130629	-2.155700
C	3.855174	5.385210	-0.338298
H	4.806154	5.499144	-0.870988
H	3.937404	5.918299	0.614843
H	3.077605	5.878361	-0.930833
C	0.920905	-5.416622	4.451169
H	0.458677	-5.737492	5.392354
H	1.987942	-5.665332	4.497641
H	0.480937	-6.004090	3.638341
C	1.524989	-3.114843	5.279844
H	1.352909	-2.039491	5.168329
H	2.595784	-3.302692	5.152132
H	1.265795	-3.406087	6.304451
C	-1.942799	-6.175995	2.141511

H	-1.836776	-6.070735	3.223601
H	-1.777734	-7.217777	1.864561
H	-2.974196	-5.912229	1.889429
C	-1.390765	-3.116659	5.357548
H	-0.836615	-3.068408	6.290087
C	-1.731613	4.747906	4.495781
H	-2.011192	4.892657	5.536176
C	3.551209	3.896878	-0.109019
H	2.569174	3.822482	0.373469
C	4.944596	-3.883088	-0.493237
H	5.829647	-4.164107	0.072344
C	-4.552739	3.392119	1.480784
H	-4.994504	3.281525	2.477433
H	-5.148578	2.795042	0.783835
H	-4.643219	4.445822	1.194016
C	5.654567	-0.336469	0.971517
H	6.724697	-0.388249	0.786727
C	-0.083273	1.703811	5.260734
H	-0.464292	2.214187	6.141760
C	4.585833	1.946818	-3.221293
H	5.465515	1.426857	-3.592174
C	-2.998596	1.453506	1.900747
H	-1.976322	1.069466	1.814611
H	-3.655841	0.831966	1.290206
H	-3.299098	1.338879	2.949291
C	2.278955	5.413601	3.038092
C	3.974408	-3.775064	2.515626
H	4.992705	-3.401354	2.355999
H	3.939720	-4.255258	3.501495
H	3.299703	-2.913704	2.518558
C	-3.949723	-1.867178	1.165834
H	-4.476026	-1.259452	1.911610
H	-4.569548	-1.909354	0.263836
H	-3.010128	-1.368935	0.906432
C	-5.013462	-4.018790	1.909966
H	-4.871837	-5.003047	2.369737
H	-5.518420	-4.159743	0.948964
H	-5.693502	-3.451145	2.554689
C	-2.729496	-2.745395	5.342747
H	-3.214252	-2.419922	6.259835
C	4.432274	-6.048373	1.531303
H	4.217508	-6.760270	0.727013
H	4.238249	-6.547696	2.486669
H	5.503324	-5.821488	1.494004
C	0.964400	7.361000	2.135109
H	0.170009	7.691283	1.458369
H	1.903374	7.806206	1.787775
H	0.748628	7.766616	3.130086
C	1.450352	-2.653031	-2.746369
C	0.712115	-3.809391	-3.437573
H	1.387515	-4.365496	-4.099914
H	-0.104797	-3.406427	-4.046199
H	0.265073	-4.507147	-2.725896
C	1.791501	-1.594428	-3.795409
H	2.152796	5.726305	4.080461
H	3.200463	5.877698	2.670238
H	2.412602	4.327390	3.025358
H	2.407799	-2.007877	-4.603341
H	2.320129	-0.734286	-3.366132
H	0.866683	-1.232182	-4.248907
H	-1.963363	-1.151754	-4.172406
C	-1.190798	0.478160	-1.213220

H	-0.987138	-0.719304	-3.237180

07_Int2.log

SCF (wB97x) = -3920.42725268

E(SCF)+ZPE(0 K)= -3918.851732

H(298 K)= -3918.754359

G(298 K)= -3918.981534

Lowest Frequency = 15.5464cm-1

W	-2.606099	-0.455622	-2.711494
Al	0.094743	2.852065	-0.448272
Al	0.238114	-2.762373	0.808637
O	-0.636672	-1.585939	-0.141005
O	-1.049046	1.766886	-1.259893
O	1.145028	-1.716367	1.857910
O	0.924347	1.644422	0.502138
C	-4.198313	-1.167786	-3.779983
N	1.129143	3.843838	-1.708929
N	-0.821256	-4.015279	1.785019
C	-4.037217	0.372979	-1.469837
N	-0.696410	4.313425	0.426600
N	1.250491	-4.054789	-0.167615
C	-2.412810	-2.343310	-1.900357
O	-5.073703	-1.595980	-4.399378
O	-4.846872	0.885796	-0.827121
O	-2.460766	2.313281	-4.303466
O	-2.333767	-3.439775	-1.546659
C	1.464672	1.598322	3.416728
H	2.286410	2.035629	2.854908
C	-1.492205	-3.590815	2.993921
C	-0.370668	-0.250620	-0.211831
C	1.134215	2.836709	-4.541228
H	0.317745	3.371448	-4.043806
C	0.914342	0.384516	2.995210
C	-1.020834	4.397069	1.835858
C	2.563738	-3.739332	-0.687606
C	1.440415	5.829505	-3.158418
H	2.429362	5.425914	-3.374450
H	1.534652	6.870754	-2.841090
H	0.850183	5.817503	-4.080390
C	-2.850299	-3.229420	2.953692
C	2.892968	-0.207755	1.455183
C	-2.174065	3.782112	2.345450
C	2.305623	2.705433	-3.573984
C	0.586331	0.351407	0.578210
C	-2.486205	3.971000	3.694068
H	-3.374008	3.494417	4.102344
C	3.456401	2.048740	-4.021543
H	3.462540	1.618882	-5.020174
C	0.839484	-5.318980	-0.321143
C	2.321393	3.241599	-2.272456
C	-2.090679	6.333348	0.169648
H	-3.021578	5.779320	0.328780
H	-2.269674	7.127052	-0.556556
H	-1.821175	6.780285	1.129223
C	0.713800	5.045832	-2.096152
C	5.171994	-0.283276	2.280492
H	5.861809	-0.296780	3.120823
C	3.801338	-0.229912	2.516195
H	3.431723	-0.218571	3.538096
C	-0.182628	5.172130	2.665025

H	0.751651	-2.203344	-2.030032
C	-0.269872	-5.861911	0.336922
H	-0.515467	-6.888420	0.095303
C	1.638433	-6.283799	-1.165256
H	2.091406	-5.804112	-2.032975
H	0.999805	-7.105104	-1.495379
H	2.449435	-6.707259	-0.562956
C	3.590200	-4.776341	1.434557
H	2.547792	-5.067070	1.608313
C	-0.752151	-3.545190	4.194240
C	1.383248	-0.341316	1.716509
C	3.389549	-0.254871	0.152006
H	2.702158	-0.246773	-0.692775
C	0.585243	1.470603	-4.954074
H	1.351239	0.873940	-5.462430
H	-0.257866	1.593584	-5.640653
H	0.230221	0.905241	-4.090207
C	3.472224	3.171500	-1.458800
C	0.978194	2.241911	4.549404
H	1.422151	3.182012	4.867013
C	2.685415	-3.104976	-1.939722
C	-1.026850	5.386292	-0.326626
C	3.691235	-4.124146	0.060166
C	-0.643000	0.496158	4.851557
H	-1.470932	0.049718	5.397556
C	4.759241	-0.321230	-0.086713
H	5.117652	-0.366737	-1.111406
C	3.973383	-2.907994	-2.442218
H	4.102363	-2.432910	-3.409257
C	-3.680320	-3.275930	1.678245
H	-3.108069	-3.798788	0.906051

C	1.101314	5.827810	2.166891
H	1.293756	5.473505	1.148953
C	4.592448	2.510896	-1.958551
H	5.483763	2.431451	-1.344621
C	4.554755	3.287807	0.861653
H	4.386203	2.217980	1.010399
H	4.466663	3.788002	1.831611
H	5.586474	3.432593	0.521552
C	0.723007	-3.928250	4.243733
H	1.163334	-3.661936	3.279604
C	-0.978086	-5.286198	1.395807
C	-2.519314	1.318125	-3.719140
C	-3.090260	2.918141	1.496740
H	-2.754347	2.969499	0.454503
C	1.540508	3.621533	-5.798992
H	2.017346	4.578626	-5.568839
H	0.659730	3.815654	-6.419901
H	2.246617	3.042506	-6.404431
C	-0.417143	5.683252	-1.540319
H	-0.746971	6.584365	-2.043824
C	-0.554668	5.356648	3.995931
H	0.067222	5.971310	4.643049
C	-0.149850	-0.149450	3.719962
H	-0.596190	-1.083513	3.399613
C	-3.453947	-2.802864	4.139372
H	-4.499904	-2.506318	4.125135
C	5.098646	-3.297691	-1.727156
H	6.090038	-3.131091	-2.141365
C	3.846293	5.376194	-0.349708
H	4.795392	5.488017	-0.886141
H	3.932333	5.912140	0.601429

H	3.066976	5.868082	-0.940985
C	0.907363	-5.437965	4.445331
H	0.438384	-5.768003	5.379986
H	1.974165	-5.686562	4.497292
H	0.473931	-6.017748	3.623629
C	1.506196	-3.145331	5.300744
H	1.336415	-2.068623	5.199204
H	2.577614	-3.333183	5.178171
H	1.239802	-3.446774	6.320568
C	-1.943917	-6.186793	2.126715
H	-1.844758	-6.085583	3.209858
H	-1.779362	-7.227961	1.847159
H	-2.973023	-5.919797	1.868736
C	-1.409725	-3.136265	5.354662
H	-0.862092	-3.091763	6.291237
C	-1.699442	4.765133	4.511966
H	-1.972005	4.917672	5.553102
C	3.542841	3.888449	-0.115392
H	2.561579	3.814959	0.368712
C	4.955656	-3.883000	-0.479853
H	5.840168	-4.163806	0.086689
C	-4.544731	3.403334	1.532258
H	-4.978729	3.294992	2.532539
H	-5.150090	2.811549	0.839453
H	-4.630451	4.457953	1.247447
C	5.656832	-0.332830	0.976270
H	6.726852	-0.382461	0.790287
C	-0.078348	1.693999	5.272326
H	-0.459151	2.203956	6.153691
C	4.583577	1.938653	-3.224204
H	5.464477	1.419459	-3.593233
C	-2.997497	1.457629	1.948877
H	-1.979618	1.065250	1.848351
H	-3.669556	0.836307	1.354232
H	-3.280574	1.352822	3.003188
C	2.299095	5.420747	3.033192
C	3.975042	-3.774189	2.529057
H	4.992830	-3.397417	2.373325
H	3.938025	-4.254621	3.514794
H	3.297658	-2.914906	2.529633
C	-3.940724	-1.862906	1.154286
H	-4.475663	-1.263452	1.900639
H	-4.551177	-1.898675	0.245411
H	-2.999596	-1.361266	0.907967
C	-5.007025	-4.021544	1.874253
H	-4.867883	-5.009480	2.326710
H	-5.505884	-4.154865	0.908975
H	-5.691362	-3.459679	2.519518
C	-2.747582	-2.761955	5.331379
H	-3.238107	-2.438222	6.246005
C	4.443430	-6.046176	1.546249
H	4.233370	-6.758689	0.741248
H	4.247487	-6.545992	2.500980
H	5.513962	-5.816309	1.512685
C	0.983030	7.356265	2.106621
H	0.188671	7.677444	1.425470
H	1.921939	7.797024	1.753452
H	0.767152	7.774761	3.096240
C	1.464053	-2.650390	-2.735420
C	0.729745	-3.803327	-3.436173
H	1.407170	-4.350364	-4.104092
H	-0.088687	-3.395848	-4.039585

H 0.286254 -4.509916 -2.730870
C 1.801487 -1.583208 -3.776824
H 2.176394 5.750117 4.070762
H 3.220745 5.876941 2.656158
H 2.430640 4.334144 3.037487
H 2.418403 -1.988866 -4.588384
H 2.328579 -0.724884 -3.341576
H 0.872846 -1.221496 -4.222881
H -2.237117 -1.165822 -4.281670
C -1.197815 0.468535 -1.189785
H -0.906053 -0.610502 -3.089562

08_TS3.log

SCF (wB97x) = -3920.42621948

E(SCF)+ZPE(0 K)= -3918.851855

H(298 K)= -3918.754855

G(298 K)= -3918.981719

Lowest Frequency = -412.5183cm-1

W -3.027619 10.295764 10.638424
Al -0.354780 13.576224 12.868813
Al -0.218190 7.969574 14.143455
O -1.094594 9.144432 13.190277
O -1.491919 12.498205 12.048753
O 0.674409 9.017098 15.200524
O 0.471690 12.379089 13.830747
C -4.565016 9.482145 9.524172
N 0.685722 14.572917 11.612936
N -1.272687 6.713078 15.119095
C -4.511619 11.095227 11.814476
N -1.153169 15.043751 13.731562
N 0.785704 6.679101 13.153409
C -2.828829 8.402501 11.445440
O -5.383250 8.989621 8.880327
O -5.349569 11.602779 12.425168
O -2.974835 13.066696 9.042515
O -2.772277 7.301987 11.789851
C 0.983877 12.328839 16.761610
H 1.816490 12.760523 16.211786
C -1.935187 7.138183 16.332044
C -0.823939 10.478694 13.120221
C 0.691860 13.581985 8.777654
H -0.114083 14.134789 9.272117
C 0.430145 11.120454 16.329845
C -1.490427 15.124913 15.137462
C 2.088614 7.002897 12.612839
C 1.016649 16.558865 10.167698
H 2.003284 16.148034 9.954657
H 1.118777 17.597633 10.490852
H 0.431131 16.557346 9.242670
C -3.292750 7.501957 16.299999
C 2.423913 10.524023 14.810439
C -2.651897 14.514598 15.633525
C 1.856337 13.427214 9.750249
C 0.125358 11.081904 13.908685
C -2.976102 14.701073 16.979564
H -3.870369 14.228137 17.377992
C 2.998951 12.753064 9.308010
H 3.003086 12.321921 8.309807
C 0.378499 5.413023 13.005013

C	1.874108	13.962692	11.052135
C	-2.538316	17.069226	13.469593
H	-3.472898	16.518783	13.619195
H	-2.707875	17.865874	12.744307
H	-2.275181	17.512195	14.432771
C	0.278304	15.776695	11.223577
C	4.695421	10.443648	15.657122
H	5.377266	10.431356	16.503980
C	3.322722	10.502129	15.879725
H	2.944010	10.518085	16.898218
C	-0.655888	15.893042	15.976446
H	0.233034	8.502731	11.304942
C	-0.723057	4.865685	13.672569
H	-0.965381	3.837507	13.434696
C	1.174380	4.450340	12.155320
H	1.621541	4.930953	11.285121
H	0.535478	3.627844	11.828639
H	1.989461	4.027961	12.752999
C	3.155921	5.959095	14.710436
H	2.121027	5.646840	14.891802
C	-1.187036	7.183523	17.526848
C	0.911143	10.393883	15.056796
C	2.932910	10.470482	13.512261
H	2.252993	10.480048	12.661654
C	0.111821	12.229908	8.361639
H	0.863238	11.615265	7.852876
H	-0.728770	12.373249	7.675566
H	-0.255392	11.671809	9.224777
C	3.021310	13.880374	11.869649
C	0.487026	12.974517	17.888713
H	0.934452	13.910392	18.213998
C	2.184834	7.643759	11.361972
C	-1.474147	16.119177	12.979230
C	3.231054	6.621651	13.339425
C	-1.153513	11.242652	18.163389
H	-1.993145	10.802703	18.696607
C	4.304503	10.397113	13.286559
H	4.672114	10.346677	12.265346
C	3.462546	7.858502	10.840982
H	3.571134	8.341545	9.875265
C	-4.128583	7.459782	15.028116
H	-3.562098	6.933676	14.253684
C	0.634934	16.544269	15.490105
H	0.832395	16.193134	14.472007
C	4.134305	13.204109	11.374618
H	5.022665	13.114418	11.991447
C	4.104968	14.000779	14.190363
H	3.934626	12.932076	14.343603
H	4.017754	14.504955	15.158419
H	5.136710	14.142888	13.848877
C	0.287153	6.796981	17.568424
H	0.715766	7.025739	16.589729
C	-1.427685	5.441057	14.733981
C	-2.999325	12.072796	9.629749
C	-3.562977	13.659036	14.770775
H	-3.215244	13.714276	13.732678
C	1.120569	14.358997	7.522554
H	1.614634	15.306278	7.756944
H	0.247647	14.571196	6.896383
H	1.818810	13.766720	6.920845
C	-0.853544	16.418204	11.771424
H	-1.174877	17.322729	11.268494

C	-1.039471	16.075306	17.304351
H	-0.420254	16.684293	17.959453
C	-0.649847	10.594939	17.037844
H	-1.100692	9.665836	16.708972
C	-3.888349	7.928694	17.489593
H	-4.933892	8.226974	17.482356
C	4.602246	7.474431	11.535898
H	5.585588	7.653569	11.107925
C	3.397555	16.086965	12.977579
H	4.347248	16.198673	12.442088
H	3.483059	16.622258	13.929245
H	2.618931	16.579734	12.386381
C	0.466722	5.294150	17.818787
H	0.011536	4.998493	18.771572
H	1.532687	5.040447	17.860888
H	0.015065	4.690483	17.024275
C	1.086428	7.615060	18.585883
H	0.919199	8.687489	18.443826
H	2.155620	7.417616	18.458538
H	0.830197	7.354683	19.619451
C	-2.388598	4.540605	15.471394
H	-2.279512	4.639393	16.553837
H	-2.229063	3.499667	15.188084
H	-3.419521	4.810898	15.223919
C	-1.836463	7.592760	18.691659
H	-1.282331	7.636568	19.624550
C	-2.192493	15.488355	17.807314
H	-2.473970	15.638873	18.846392
C	3.093289	14.599430	13.212150
H	2.112149	14.526067	13.696317
C	4.484261	6.877229	12.780458
H	5.380315	6.599792	13.330149
C	-5.016084	14.148905	14.793546
H	-5.461003	14.037136	15.788678
H	-5.616210	13.561961	14.091822
H	-5.095905	15.205076	14.512745
C	5.192404	10.386711	14.357762
H	6.263980	10.332948	14.181977
C	-0.584112	12.434353	18.595412
H	-0.973072	12.945594	19.472494
C	4.121223	12.628213	10.110453
H	4.995877	12.095354	9.746039
C	-3.480945	12.195441	15.214149
H	-2.463822	11.799282	15.120842
H	-4.149538	11.581643	14.607639
H	-3.775927	12.084482	16.264672
C	1.825635	16.128224	16.361736
C	3.530494	6.961922	15.807770
H	4.539727	7.358530	15.646049
H	3.509935	6.475176	16.790934
H	2.837865	7.808995	15.819301
C	-4.382500	8.875000	14.506401
H	-4.909761	9.477086	15.256008
H	-4.997979	8.844712	13.600462
H	-3.439783	9.371624	14.256274
C	-5.457945	6.720553	15.229160
H	-5.320927	5.730679	15.678097
H	-5.962720	6.592084	14.266270
H	-6.136096	7.283967	15.879555
C	-3.174084	7.968017	18.677190
H	-3.658339	8.292057	19.595046
C	4.034394	4.705353	14.806339

H	3.833363	3.995736	13.996398
H	3.853970	4.194160	15.758028
H	5.100081	4.956073	14.768849
C	0.524112	18.073497	15.435232
H	-0.264387	18.401070	14.750304
H	1.467316	18.511325	15.089902
H	0.303788	18.489015	16.425150
C	0.945949	8.081609	10.585034
C	0.232209	6.919482	9.878274
H	0.916230	6.395986	9.198420
H	-0.601321	7.313380	9.286378
H	-0.186863	6.194606	10.580105
C	1.245547	9.170370	9.554771
H	1.698380	16.454047	17.399959
H	2.751376	16.581891	15.991434
H	1.952208	15.041101	16.362146
H	1.860334	8.790660	8.729070
H	1.760361	10.032979	9.996194
H	0.302880	9.517838	9.126377
H	-2.468198	9.638336	9.085516
C	-1.651251	11.195678	12.121984
H	-1.271360	10.418704	10.593196

09_Int3.log

SCF (wB97x) = -3920.43031309

E(SCF)+ZPE(0 K)= -3918.852999

H(298 K)= -3918.755817

G(298 K)= -3918.983359

Lowest Frequency = 13.6270cm-1

W	-2.544035	-0.427044	-2.719357
Al	0.100981	2.853003	-0.492125
Al	0.263724	-2.744250	0.790970
O	-0.585570	-1.581388	-0.207161
O	-0.986175	1.795264	-1.375334
O	1.106244	-1.689619	1.876453
O	0.923622	1.676596	0.495168
C	-4.162782	-1.285074	-3.661603
N	1.171895	3.867578	-1.719515
N	-0.795685	-4.003960	1.756808
C	-3.944775	0.448354	-1.532338
N	-0.690721	4.329973	0.370676
N	1.269031	-4.034924	-0.202680
C	-2.343801	-2.313260	-1.886958
O	-5.027156	-1.812974	-4.206330
O	-4.760476	0.995203	-0.924319
O	-2.617712	2.309171	-4.381093
O	-2.312807	-3.412918	-1.540897
C	1.402124	1.628733	3.425798
H	2.234714	2.061120	2.876710
C	-1.454305	-3.580015	2.972385
C	-0.328302	-0.242429	-0.252048
C	1.166456	2.878886	-4.548422
H	0.375793	3.454704	-4.056340
C	0.854148	0.417189	2.995631
C	-1.024374	4.419530	1.776429
C	2.562232	-3.700326	-0.760089
C	1.523315	5.852403	-3.161859
H	2.503984	5.429116	-3.378329
H	1.641757	6.885735	-2.826499
H	0.939033	5.872166	-4.087521

C	-2.809741	-3.208078	2.943293	C	1.346224	-0.310969	1.728841
C	2.863904	-0.187214	1.504311	C	3.391129	-0.249209	0.213749
C	-2.185871	3.816559	2.280228	H	2.721796	-0.238923	-0.644999
C	2.329527	2.706578	-3.577158	C	0.552505	1.537115	-4.947405
C	0.581425	0.368992	0.564154	H	1.286201	0.895729	-5.448581
C	-2.509286	4.017784	3.624451	H	-0.287208	1.690210	-5.632481
H	-3.404120	3.550397	4.028130	H	0.177603	1.001209	-4.073475
C	3.460548	2.011515	-4.016446	C	3.498715	3.148023	-1.458456
H	3.458827	1.576907	-5.013335	C	0.899122	2.276989	4.548542
C	0.867479	-5.303250	-0.347273	H	1.341645	3.216133	4.871217
C	2.353847	3.245904	-2.277010	C	2.634821	-3.054538	-2.009824
C	-2.066093	6.362168	0.104647	C	-1.000746	5.409608	-0.379830
H	-3.001260	5.812955	0.255072	C	3.718723	-4.073386	-0.051839
H	-2.233593	7.156100	-0.624268	C	-0.736790	0.540937	4.822805
H	-1.804466	6.809610	1.066243	H	-1.577092	0.100389	5.354615
C	0.771437	5.070242	-2.114532	C	4.765423	-0.329326	0.006939
C	5.124290	-0.268809	2.381964	H	5.146567	-0.385788	-1.009035
H	5.794502	-0.276941	3.238154	C	3.902258	-2.815616	-2.544918
C	3.748765	-0.205333	2.585524	H	3.991331	-2.324975	-3.509019
H	3.357148	-0.180660	3.598893	C	-3.645421	-3.241491	1.671166
C	-0.186519	5.191848	2.608722	H	-3.081593	-3.770917	0.897152
H	0.655888	-2.270045	-2.040753	C	1.109913	5.828266	2.117863
C	-0.233027	-5.853873	0.319589	H	1.309247	5.459620	1.106446
H	-0.469137	-6.884258	0.084992	C	4.600896	2.451576	-1.949873
C	1.669158	-6.265545	-1.192354	H	5.486897	2.349678	-1.331457
H	2.118126	-5.786610	-2.062541	C	4.583116	3.269933	0.862345
H	1.034399	-7.091443	-1.518550	H	4.400726	2.204811	1.025012
H	2.483483	-6.683453	-0.590234	H	4.502194	3.782919	1.826337
C	3.667704	-4.747471	1.314349	H	5.616145	3.397707	0.518683
H	2.643266	-5.092160	1.496027	C	0.764909	-3.944203	4.211946
C	-0.705376	-3.544740	4.166362	H	1.183275	-3.790257	3.214248

C	-0.944453	-5.277662	1.376063
C	-2.584710	1.331785	-3.771675
C	-3.100337	2.955305	1.427292
H	-2.756714	3.002370	0.387194
C	1.607516	3.635314	-5.811249
H	2.116631	4.576269	-5.583488
H	0.738585	3.857196	-6.439724
H	2.296331	3.026755	-6.407542
C	-0.367085	5.712255	-1.579967
H	-0.681174	6.618888	-2.083919
C	-0.570006	5.390691	3.934210
H	0.051334	6.004527	4.582770
C	-0.227370	-0.108767	3.701018
H	-0.674557	-1.039772	3.372267
C	-3.401800	-2.782372	4.134744
H	-4.445415	-2.477343	4.129757
C	5.055449	-3.187539	-1.866052
H	6.031060	-2.990828	-2.303868
C	3.894160	5.355296	-0.360580
H	4.844252	5.456352	-0.897643
H	3.985893	5.893793	0.588647
H	3.119211	5.852700	-0.952533
C	0.927864	-5.429299	4.560134
H	0.487039	-5.653234	5.538802
H	1.990359	-5.697065	4.599055
H	0.450032	-6.077097	3.817060
C	1.584060	-3.068043	5.163276
H	1.431273	-2.006897	4.943720
H	2.649413	-3.290934	5.045956
H	1.329301	-3.248571	6.213951
C	-1.906375	-6.177482	2.112676
H	-1.791310	-6.085258	3.195132
H	-1.754545	-7.217722	1.822731
H	-2.937152	-5.899185	1.873127
C	-1.351547	-3.137423	5.333753
H	-0.796252	-3.103239	6.266598
C	-1.724968	4.813114	4.443511
H	-2.006431	4.976046	5.480753
C	3.576734	3.871567	-0.119114
H	2.594090	3.806247	0.362549
C	4.960875	-3.797565	-0.625401
H	5.867600	-4.067660	-0.089956
C	-4.552167	3.448881	1.455312
H	-4.993329	3.338857	2.452356
H	-5.156733	2.864060	0.756150
H	-4.629395	4.505452	1.175005
C	5.639004	-0.335954	1.089952
H	6.712797	-0.393262	0.929129
C	-0.172791	1.735769	5.253263
H	-0.566622	2.248654	6.127265
C	4.579485	1.870349	-3.211627
H	5.445324	1.320920	-3.572551
C	-3.016087	1.494872	1.880860
H	-2.004761	1.091053	1.758906
H	-3.706549	0.881843	1.299006
H	-3.279249	1.394746	2.940886
C	2.293812	5.418032	3.001607
C	4.014107	-3.741324	2.417601
H	5.011244	-3.314998	2.257185
H	4.008033	-4.234650	3.397627
H	3.298534	-2.913416	2.436249
C	-3.889438	-1.822925	1.153280

H	-4.417468	-1.222080	1.903058
H	-4.501206	-1.845268	0.244178
H	-2.944267	-1.326779	0.910894
C	-4.979381	-3.972951	1.869223
H	-4.847621	-4.966131	2.312408
H	-5.486026	-4.093323	0.906197
H	-5.653382	-3.409060	2.523452
C	-2.686296	-2.753046	5.322207
H	-3.167749	-2.429863	6.241810
C	4.582793	-5.975435	1.398845
H	4.403034	-6.682324	0.581401
H	4.416877	-6.500794	2.345325
H	5.640729	-5.693473	1.365405
C	1.009368	7.357080	2.038274
H	0.228421	7.678472	1.341846
H	1.958025	7.783547	1.693466
H	0.784007	7.789493	3.019860
C	1.379712	-2.644264	-2.774137
C	0.712025	-3.818090	-3.506004
H	1.417210	-4.302967	-4.192627
H	-0.134498	-3.446632	-4.094278
H	0.320451	-4.571770	-2.818334
C	1.633632	-1.518015	-3.775794
H	2.164817	5.761947	4.033749
H	3.224503	5.858533	2.627933
H	2.412279	4.330107	3.020500
H	2.255350	-1.851187	-4.616214
H	2.121894	-0.651137	-3.313209
H	0.676905	-1.192613	-4.192625
H	-1.968503	-1.080383	-4.285015
C	-1.113644	0.460507	-1.317309
H	-0.747666	-0.056897	-2.421131

10_TS4.log

SCF (wB97x) = -3920.41101624
E(SCF)+ZPE(0 K)= -3918.833187
H(298 K)= -3918.736730
G(298 K)= -3918.961495
Lowest Frequency = -37.4264cm-1

W	-2.735759	-0.395523	-2.514431
Al	0.130211	2.840817	-0.504578
Al	0.272035	-2.741890	0.843410
O	-0.563607	-1.588340	-0.181019
O	-0.912879	1.793488	-1.419694
O	1.106257	-1.685903	1.922135
O	0.935791	1.682514	0.524007
C	-2.951975	-1.399675	-4.286715
N	1.240716	3.875503	-1.679368
N	-0.787911	-3.974880	1.842148
C	-4.666651	-0.821224	-2.255993
N	-0.684074	4.334691	0.323492
N	1.222651	-4.052176	-0.179482
C	-2.327819	-2.318306	-1.635183
O	-3.018424	-1.890256	-5.327490
O	-5.780955	-1.072869	-2.045897
O	-3.887194	2.537365	-2.214395
O	-2.291672	-3.420449	-1.324269
C	1.465259	1.561085	3.536184
H	2.362367	1.945912	3.058435
C	-1.396147	-3.534445	3.079717

C	-0.306367	-0.244080	-0.236822
C	1.166002	2.894251	-4.477155
H	0.452114	3.566942	-3.993810
C	0.847926	0.422803	3.016238
C	-1.099112	4.369033	1.707432
C	2.500462	-3.735276	-0.782666
C	1.682503	5.847511	-3.113862
H	2.660185	5.401920	-3.298117
H	1.813123	6.882545	-2.788667
H	1.127076	5.867023	-4.057398
C	-2.748387	-3.148850	3.098572
C	2.871231	-0.210521	1.534054
C	-2.308271	3.779867	2.104706
C	2.342555	2.675175	-3.531345
C	0.595197	0.366823	0.582586
C	-2.692717	3.895572	3.442246
H	-3.624309	3.438141	3.767371
C	3.442644	1.936803	-3.976425
H	3.416831	1.492896	-4.969234
C	0.796542	-5.315211	-0.300853
C	2.399522	3.219306	-2.235024
C	-1.997954	6.405682	0.061892
H	-2.963159	5.888615	0.085177
H	-2.070597	7.257716	-0.615202
H	-1.807864	6.767288	1.074628
C	0.885341	5.091700	-2.078315
C	5.130337	-0.375715	2.406828
H	5.800744	-0.415309	3.262044
C	3.757711	-0.273265	2.613205
H	3.369730	-0.255002	3.628015
C	-0.283030	5.061497	2.625495
H	0.562180	-2.306285	-1.996605
C	-0.289926	-5.844280	0.405814
H	-0.544431	-6.874480	0.190111
C	1.554986	-6.295245	-1.165398
H	1.988719	-5.827884	-2.049598
H	0.896205	-7.110480	-1.470213
H	2.378237	-6.726650	-0.585266
C	3.663796	-4.811274	1.244398
H	2.645135	-5.157470	1.452936
C	-0.606004	-3.497583	4.245774
C	1.351129	-0.306731	1.755925
C	3.395984	-0.273797	0.242360
H	2.725840	-0.227097	-0.614328
C	0.407180	1.599094	-4.765184
H	1.050243	0.863063	-5.261123
H	-0.449653	1.795796	-5.419333
H	0.025231	1.165110	-3.838874
C	3.543268	3.088754	-1.421313
C	0.950412	2.198788	4.661220
H	1.452227	3.078667	5.056675
C	2.540506	-3.080898	-2.029599
C	-0.934268	5.441617	-0.402894
C	3.676365	-4.125583	-0.116602
C	-0.846704	0.609484	4.743115
H	-1.756510	0.225618	5.198616
C	4.766626	-0.400620	0.032625
H	5.144133	-0.459814	-0.984660
C	3.791951	-2.842341	-2.600855
H	3.854237	-2.341819	-3.562095
C	-3.625264	-3.159111	1.854322
H	-3.103502	-3.714298	1.068498

C	1.034403	5.718536	2.222350
H	1.260781	5.424763	1.191989
C	4.619156	2.357419	-1.921340
H	5.507215	2.228916	-1.310556
C	4.646250	3.183227	0.893393
H	4.466934	2.116592	1.046998
H	4.570913	3.685875	1.863741
H	5.676419	3.317127	0.542563
C	0.861477	-3.906898	4.256212
H	1.230143	-3.874526	3.227761
C	-0.958916	-5.251021	1.482025
C	-3.446758	1.477494	-2.321406
C	-3.199859	3.034453	1.126947
H	-2.789144	3.163902	0.120482
C	1.622909	3.546429	-5.789703
H	2.205816	4.457124	-5.618991
H	0.754821	3.803970	-6.405828
H	2.246723	2.861439	-6.374422
C	-0.242333	5.763639	-1.566962
H	-0.508435	6.693586	-2.055733
C	-0.723887	5.173753	3.943289
H	-0.118057	5.719103	4.664054
C	-0.322934	-0.032535	3.625510
H	-0.828783	-0.905491	3.226551
C	-3.295466	-2.716884	4.308919
H	-4.336142	-2.403752	4.339621
C	4.962969	-3.226913	-1.961171
H	5.926041	-3.029902	-2.425614
C	3.940838	5.285799	-0.278346
H	4.891243	5.407134	-0.810997
H	4.028652	5.797583	0.686501

H	3.163613	5.796418	-0.854811
C	1.030631	-5.344774	4.763017
H	0.650491	-5.448136	5.786157
H	2.090084	-5.626522	4.766754
H	0.497055	-6.063713	4.131102
C	1.725350	-2.932898	5.062389
H	1.568423	-1.906747	4.717303
H	2.784264	-3.180571	4.935993
H	1.506015	-2.979977	6.135028
C	-1.895298	-6.134424	2.268995
H	-1.685607	-6.074729	3.340018
H	-1.808359	-7.171560	1.943834
H	-2.930846	-5.810642	2.128339
C	-1.207310	-3.081408	5.433803
H	-0.617785	-3.049491	6.345758
C	-1.918597	4.596851	4.353601
H	-2.242665	4.690805	5.386992
C	3.631165	3.795437	-0.072167
H	2.651511	3.714252	0.413650
C	4.901781	-3.852253	-0.726031
H	5.822634	-4.134580	-0.222127
C	-4.632909	3.578264	1.110708
H	-5.146390	3.386986	2.059921
H	-5.207198	3.091341	0.315941
H	-4.658728	4.658951	0.932523
C	5.641470	-0.446213	1.113434
H	6.712643	-0.536971	0.950357
C	-0.205153	1.723814	5.272356
H	-0.607647	2.227264	6.147887
C	4.564948	1.768274	-3.179339
H	5.409508	1.188728	-3.543958

C	-3.196974	1.535576	1.435379
H	-2.182903	1.120643	1.415804
H	-3.797413	0.993633	0.697200
H	-3.613275	1.339759	2.431652
C	2.198637	5.242130	3.097280
C	4.041036	-3.813039	2.344226
H	5.037714	-3.393794	2.164540
H	4.050993	-4.310256	3.322170
H	3.333633	-2.978572	2.381904
C	-3.831197	-1.730673	1.345040
H	-4.304639	-1.110595	2.115048
H	-4.481073	-1.719783	0.461934
H	-2.877766	-1.266212	1.073520
C	-4.977330	-3.843618	2.090485
H	-4.866105	-4.843792	2.523807
H	-5.517692	-3.939630	1.143246
H	-5.611112	-3.260668	2.767418
C	-2.538593	-2.690094	5.470614
H	-2.984590	-2.361349	6.405892
C	4.580158	-6.040085	1.292896
H	4.376133	-6.740381	0.475377
H	4.441573	-6.572491	2.239703
H	5.636852	-5.758514	1.231000
C	0.939387	7.249796	2.254615
H	0.171336	7.626264	1.571680
H	1.895310	7.696839	1.959619
H	0.697817	7.608126	3.261909
C	1.266426	-2.662200	-2.755790
C	0.594197	-3.829727	-3.493435
H	1.288670	-4.298378	-4.201348
H	-0.266249	-3.462717	-4.065641
H	0.224458	-4.598734	-2.810152
C	1.488982	-1.511479	-3.736829
H	2.047224	5.505182	4.150385
H	3.134525	5.712993	2.775514
H	2.318899	4.157088	3.032209
H	2.082898	-1.820682	-4.605890
H	1.988703	-0.656208	-3.266387
H	0.521391	-1.169725	-4.115847
H	-2.578992	0.605717	-3.926196
C	-1.120660	0.445898	-1.299359
H	-0.797260	-0.081239	-2.353268

11_Int4.log

SCF (wB97x) = -3920.41607082
E(SCF)+ZPE(0 K)= -3918.838555
H(298 K)= -3918.741140
G(298 K)= -3918.969300
Lowest Frequency = 13.8025cm-1

W	-2.704692	-0.306880	-2.666657
Al	0.028502	2.876996	-0.495828
Al	0.264580	-2.720905	0.742433
O	-0.470179	-1.598669	-0.382732
O	-1.148109	1.838236	-1.266746
O	1.070125	-1.661000	1.840046
O	0.898730	1.696594	0.444558
C	-1.862962	-1.660904	-3.954253
N	1.105240	3.857025	-1.752396
N	-0.795152	-3.916288	1.808609
C	-4.656362	-0.774281	-3.014487

N	-0.715878	4.390732	0.350104
N	1.221743	-4.063326	-0.216915
C	-2.814341	-1.984316	-1.523722
O	-1.417909	-2.371536	-4.742297
O	-5.770613	-1.020026	-3.179298
O	-4.189642	2.443188	-1.830459
O	-2.949614	-3.043926	-1.077921
C	1.463496	1.613141	3.402396
H	2.338152	2.006048	2.889869
C	-1.348466	-3.498229	3.084567
C	-0.300899	-0.243887	-0.349361
C	1.047931	2.852300	-4.572130
H	0.286283	3.462519	-4.076387
C	0.855557	0.451024	2.923939
C	-1.075789	4.461493	1.749343
C	2.551524	-3.819425	-0.738557
C	1.510028	5.821514	-3.210399
H	2.478205	5.370093	-3.426395
H	1.657675	6.855631	-2.889706
H	0.922209	5.844702	-4.133809
C	-2.697399	-3.109792	3.179260
C	2.863070	-0.212924	1.447580
C	-2.276582	3.895351	2.203253
C	2.216909	2.655299	-3.611924
C	0.582237	0.378076	0.490610
C	-2.608799	4.037285	3.551764
H	-3.531162	3.594712	3.920082
C	3.328394	1.934958	-4.059425
H	3.307090	1.494272	-5.053624
C	0.698050	-5.282035	-0.401441
C	2.266514	3.203292	-2.315850
C	-2.029029	6.462629	0.091605
H	-2.992350	5.951714	0.195775
H	-2.141181	7.287267	-0.613357
H	-1.776198	6.863901	1.075342
C	0.743394	5.072340	-2.148659
C	5.113518	-0.394675	2.337931
H	5.776871	-0.444575	3.198072
C	3.739915	-0.286490	2.534044
H	3.342159	-0.269605	3.545358
C	-0.222247	5.165757	2.622446
H	0.647204	-3.117723	-2.447857
C	-0.415120	-5.760812	0.297240
H	-0.748477	-6.758200	0.040305
C	1.355756	-6.233931	-1.368376
H	1.427537	-5.779150	-2.359534
H	0.789117	-7.162488	-1.442481
H	2.376661	-6.465596	-1.052078
C	3.514604	-4.832279	1.428981
H	2.448416	-4.933038	1.657915
C	-0.513480	-3.492559	4.221191
C	1.342119	-0.293583	1.663670
C	3.398079	-0.267850	0.160523
H	2.738292	-0.206861	-0.701639
C	0.378290	1.529650	-4.947580
H	1.076901	0.862193	-5.465551
H	-0.478426	1.704732	-5.606815
H	0.005258	1.014761	-4.061519
C	3.418987	3.090724	-1.509233
C	0.970140	2.259776	4.531395
H	1.463473	3.157330	4.896246
C	2.738231	-3.252958	-2.011699

C	-0.995734	5.477836	-0.397364
C	3.656527	-4.192231	0.054389
C	-0.781330	0.626810	4.706473
H	-1.662555	0.225974	5.202095
C	4.769961	-0.397864	-0.039355
H	5.155742	-0.451975	-1.054140
C	4.045695	-3.059035	-2.466061
H	4.202610	-2.624794	-3.450799
C	-3.621349	-3.037399	1.975405
H	-3.152718	-3.566181	1.143349
C	1.085954	5.801811	2.162175
H	1.276578	5.481057	1.132636
C	4.502004	2.370930	-2.009951
H	5.393688	2.256795	-1.402007
C	4.553231	3.215722	0.788308
H	4.385965	2.147901	0.949532
H	4.485473	3.723436	1.756260
H	5.577910	3.356802	0.424974
C	0.958706	-3.875450	4.157476
H	1.316274	-3.644296	3.152011
C	-1.028322	-5.172028	1.414227
C	-3.619200	1.475795	-2.066514
C	-3.211718	3.147431	1.270270
H	-2.873267	3.314093	0.243076
C	1.497622	3.579471	-5.848999
H	2.038321	4.506419	-5.637118
H	0.629451	3.821647	-6.471175
H	2.160293	2.943160	-6.445815
C	-0.362059	5.758481	-1.603744
H	-0.645358	6.677776	-2.102907
C	-0.614047	5.309046	3.953173
H	0.021193	5.865638	4.639024
C	-0.278543	-0.024210	3.583479
H	-0.769066	-0.920503	3.220774
C	-3.197498	-2.729394	4.426973
H	-4.236214	-2.419183	4.507993
C	5.141089	-3.419225	-1.698431
H	6.149284	-3.264822	-2.074568
C	3.829678	5.300601	-0.405449
H	4.769879	5.410828	-0.958046
H	3.936695	5.828792	0.548326
H	3.042728	5.802904	-0.976386
C	1.151023	-5.377199	4.402967
H	0.757595	-5.666537	5.384722
H	2.215220	-5.640214	4.376045
H	0.641896	-5.980601	3.642558
C	1.813477	-3.041407	5.116778
H	1.612331	-1.973617	4.980483
H	2.875083	-3.216600	4.921469
H	1.632211	-3.299620	6.166300
C	-1.967237	-6.050001	2.204941
H	-1.713358	-6.034010	3.268165
H	-1.926673	-7.077727	1.842991
H	-2.995628	-5.688541	2.115193
C	-1.067951	-3.125049	5.447624
H	-0.441548	-3.119623	6.334083
C	-1.795307	4.748891	4.420043
H	-2.079603	4.865693	5.462741
C	3.522122	3.813815	-0.170146
H	2.550366	3.739784	0.331825
C	4.940397	-3.980750	-0.446352
H	5.799157	-4.263064	0.158238

C	-4.657513	3.647360	1.362060
H	-5.108313	3.407652	2.331680
H	-5.264665	3.170723	0.585998
H	-4.722827	4.732607	1.225084
C	5.634992	-0.457493	1.048173
H	6.706843	-0.553981	0.893595
C	-0.153439	1.769017	5.189644
H	-0.538564	2.280019	6.068603
C	4.454332	1.780193	-3.266890
H	5.306174	1.213019	-3.633897
C	-3.141258	1.642809	1.537176
H	-2.121990	1.256146	1.427077
H	-3.785550	1.099333	0.837822
H	-3.469323	1.411989	2.558231
C	2.270835	5.336340	3.015374
C	4.140353	-3.940139	2.505065
H	5.200168	-3.751758	2.299952
H	4.072611	-4.422848	3.486707
H	3.633805	-2.971261	2.551769
C	-3.787179	-1.576584	1.550450
H	-4.195417	-0.978893	2.373684
H	-4.467857	-1.489598	0.696218
H	-2.826416	-1.139301	1.257900
C	-4.989810	-3.681622	2.226586
H	-4.902691	-4.699917	2.621761
H	-5.554076	-3.727541	1.289553
H	-5.588083	-3.102601	2.938708
C	-2.398238	-2.746675	5.558039
H	-2.806476	-2.458941	6.523573
C	4.124843	-6.239458	1.467554
H	3.680682	-6.899702	0.715037
H	3.961448	-6.695140	2.450485
H	5.205692	-6.211381	1.289778
C	1.004497	7.334172	2.160014
H	0.219391	7.700991	1.491355
H	1.954618	7.765425	1.825486
H	0.796517	7.718941	3.164995
C	1.594958	-2.873270	-2.940885
C	1.672696	-3.646617	-4.265866
H	2.558276	-3.354010	-4.841327
H	0.791374	-3.435771	-4.877953
H	1.727941	-4.729376	-4.112691
C	1.595135	-1.366843	-3.219450
H	2.149739	5.624039	4.065782
H	3.201336	5.792577	2.659521
H	2.382525	4.249067	2.972504
H	2.558931	-1.030256	-3.620416
H	1.390824	-0.792776	-2.310051
H	0.828999	-1.120440	-3.959319
H	-2.830503	0.456649	-4.282218
C	-1.169403	0.480187	-1.334796
H	-0.860111	0.171886	-2.474057

12_TS5.log

SCF (wB97x) = -3920.40701687

E(SCF)+ZPE(0 K)= -3918.828892

H(298 K)= -3918.732057

G(298 K)= -3918.957865

Lowest Frequency = -131.3931cm-1

W -3.040508 10.548415 10.536381

Al	-0.440368	13.506207	12.812146
Al	-0.191328	7.962614	14.182292
O	-1.020312	9.071104	13.114937
O	-1.565600	12.469931	11.934682
O	0.610830	9.032535	15.278632
O	0.460465	12.363086	13.765763
C	-4.917260	9.916489	10.210164
N	0.606079	14.535759	11.547119
N	-1.322352	6.724107	15.089377
C	-2.901231	8.663280	11.269034
N	-1.169537	15.004397	13.719532
N	0.856788	6.656617	13.255773
C	-2.127773	9.658065	8.929814
O	-6.008511	9.573944	10.055730
O	-2.914279	7.544638	11.551273
O	-4.838083	12.378265	12.518963
O	-1.674875	9.177928	7.986052
C	0.942226	12.396897	16.731037
H	1.732044	12.833501	16.124863
C	-2.055723	7.172952	16.251361
C	-0.740475	10.407752	13.074491
C	0.490133	13.599461	8.700743
H	-0.266280	14.189647	9.227090
C	0.404584	11.160722	16.363313
C	-1.398348	15.145935	15.144095
C	2.192386	6.961898	12.791114
C	0.899979	16.530324	10.102870
H	1.860632	16.096268	9.826539
H	1.056015	17.551130	10.461279
H	0.271166	16.590026	9.208631
C	-3.392259	7.588874	16.123327
C	2.388893	10.512676	14.880779
C	-2.532003	14.590748	15.755739
C	1.681805	13.389942	9.629683
C	0.117687	11.043226	13.911029
C	-2.757472	14.849738	17.109653
H	-3.627399	14.412796	17.593419
C	2.781049	12.676743	9.142445
H	2.736281	12.256239	8.140305
C	0.433876	5.400292	13.075607
C	1.758955	13.910403	10.936461
C	-2.554846	17.037770	13.497495
H	-3.475138	16.490510	13.726501
H	-2.774394	17.809183	12.758000
H	-2.231758	17.516417	14.425019
C	0.186329	15.734092	11.167553
C	4.647836	10.423374	15.762327
H	5.316592	10.416562	16.619801
C	3.272260	10.497927	15.963051
H	2.878701	10.535518	16.975354
C	-0.496750	15.939228	15.885862
H	0.378819	8.349395	11.391991
C	-0.721003	4.874373	13.668011
H	-0.968587	3.852140	13.410344
C	1.265722	4.423093	12.278123
H	1.771589	4.893207	11.434412
H	0.638395	3.605602	11.918241
H	2.039603	3.995288	12.925079
C	3.120480	5.922532	14.952735
H	2.074422	5.616892	15.070689
C	-1.389591	7.199928	17.494682
C	0.867384	10.410276	15.102787

C	2.919222	10.442963	13.592426
H	2.246518	10.460785	12.737676
C	-0.174617	12.277274	8.318346
H	0.503489	11.633080	7.746639
H	-1.070287	12.453092	7.712668
H	-0.477827	11.736691	9.215606
C	2.932895	13.783266	11.708844
C	0.489187	13.058167	17.867263
H	0.923731	14.016155	18.141508
C	2.370816	7.602070	11.550349
C	-1.515741	16.076464	12.971670
C	3.287018	6.562956	13.579496
C	-1.077342	11.285114	18.279223
H	-1.871995	10.835050	18.870847
C	4.293082	10.355682	13.387445
H	4.676850	10.296672	12.372248
C	3.676639	7.774791	11.087841
H	3.844035	8.248903	10.125088
C	-4.140682	7.574653	14.796867
H	-3.525168	7.059043	14.053085
C	0.776781	16.527309	15.288553
H	0.920175	16.087223	14.296856
C	3.998512	13.060873	11.175253
H	4.903203	12.935391	11.761633
C	4.130321	13.928418	13.976716
H	3.934081	12.874244	14.184525
H	4.112181	14.473404	14.926080
H	5.145914	14.022119	13.574568
C	0.068990	6.775333	17.630978
H	0.574703	7.043394	16.699340
C	-1.482950	5.460473	14.684959
C	-4.120209	11.750829	11.884472
C	-3.526717	13.733203	14.998377
H	-3.287884	13.798416	13.931641
C	0.899907	14.355617	7.427878
H	1.424897	15.290005	7.645627
H	0.015212	14.590604	6.826406
H	1.564071	13.743250	6.808249
C	-0.940705	16.368225	11.740354
H	-1.280257	17.269959	11.243656
C	-0.789844	16.207847	17.222485
H	-0.120355	16.843744	17.797422
C	-0.616810	10.622458	17.143878
H	-1.051374	9.670710	16.861532
C	-4.053408	8.040782	17.268284
H	-5.083820	8.378755	17.187659
C	4.769149	7.364278	11.839725
H	5.777256	7.512731	11.460740
C	3.412237	15.995356	12.743316
H	4.330048	16.067891	12.148456
H	3.571436	16.550917	13.673135
H	2.612476	16.496250	12.188777
C	0.203023	5.258803	17.821155
H	-0.332139	4.928893	18.719617
H	1.257974	4.983057	17.937259
H	-0.190730	4.700168	16.965666
C	0.806143	7.521999	18.745545
H	0.675693	8.604468	18.648544
H	1.877227	7.303311	18.687324
H	0.467631	7.215437	19.742278
C	-2.515937	4.581950	15.346719
H	-2.493029	4.686318	16.433809

H	-2.354649	3.536266	15.082227
H	-3.517445	4.870932	15.013997
C	-2.103419	7.636503	18.610831
H	-1.613738	7.667762	19.579499
C	-1.911577	15.669945	17.837001
H	-2.119075	15.883372	18.882488
C	3.084486	14.521841	13.032895
H	2.124750	14.474898	13.559282
C	4.572366	6.784574	13.083455
H	5.432390	6.492118	13.680679
C	-4.967793	14.224814	15.182463
H	-5.312194	14.083487	16.212892
H	-5.641223	13.664088	14.528513
H	-5.067928	15.289772	14.943973
C	5.164795	10.346203	14.471882
H	6.238546	10.282043	14.313262
C	-0.522552	12.505223	18.648057
H	-0.876388	13.027635	19.533611
C	3.920646	12.493609	9.909264
H	4.758994	11.925504	9.514086
C	-3.393819	12.266943	15.420481
H	-2.406781	11.860732	15.170899
H	-4.151993	11.661143	14.918251
H	-3.526948	12.154589	16.503064
C	1.998476	16.165043	16.141833
C	3.425366	6.945109	16.054010
H	4.445230	7.334429	15.952621
H	3.335853	6.477511	17.042550
H	2.738904	7.796172	16.003261
C	-4.360524	9.000303	14.285134
H	-4.939253	9.584964	15.009668
H	-4.919517	8.985049	13.341943
H	-3.405037	9.504558	14.108840
C	-5.484091	6.839583	14.898149
H	-5.381993	5.841465	15.337382
H	-5.928060	6.731374	13.903168
H	-6.197924	7.396035	15.515570
C	-3.422812	8.058860	18.502470
H	-3.957280	8.405141	19.383478
C	3.983647	4.667133	15.127029
H	3.829453	3.944329	14.318226
H	3.739426	4.173975	16.073963
H	5.051140	4.911374	15.153890
C	0.683926	18.047874	15.107134
H	-0.122853	18.326804	14.421788
H	1.620454	18.440069	14.694657
H	0.502442	18.549434	16.064754
C	1.188790	8.070660	10.708009
C	0.654733	6.954890	9.798097
H	1.454110	6.553695	9.162966
H	-0.131333	7.343734	9.141925
H	0.223538	6.129805	10.372231
C	1.522404	9.307542	9.868947
H	1.926933	16.587855	17.149701
H	2.915256	16.560239	15.692080
H	2.104314	15.079678	16.238232
H	2.224856	9.079020	9.058383
H	1.953579	10.115832	10.470768
H	0.613189	9.685829	9.393908
H	-3.610632	11.428810	9.094400
C	-1.376659	11.079681	11.895247
H	-0.741272	10.858567	10.994055

13_Int5.log

SCF (wB97x) = -3920.43233881

E(SCF)+ZPE(0 K)= -3918.850649

H(298 K)= -3918.754100

G(298 K)= -3918.979475

Lowest Frequency = 13.3598cm-1

W	-2.799129	0.811671	-2.381167
Al	0.070359	2.738958	-0.511636
Al	0.124121	-2.829146	0.849817
O	-0.723236	-1.701820	-0.216856
O	-1.162822	1.719525	-1.292638
O	0.958228	-1.766899	1.935128
O	0.931578	1.525876	0.371717
C	-4.549843	0.506568	-3.398124
N	1.170326	3.638595	-1.820359
N	-0.864153	-4.122706	1.868971
C	-2.443155	-1.127038	-2.894768
N	-0.692282	4.265243	0.281322
N	1.170347	-4.139873	-0.104802
C	-2.105307	1.682254	-4.102844
O	-5.547400	0.383547	-3.956898
O	-2.225378	-2.217126	-3.199621
O	-4.861797	0.355339	-0.007244
O	-1.916885	2.271842	-5.076934
C	1.059915	1.684044	3.305807
H	1.783217	2.175836	2.660238
C	-1.506481	-3.722726	3.102071
C	-0.338757	-0.386861	-0.287029
C	1.424062	2.560039	-4.657419
H	0.562337	3.105759	-4.258190
C	0.678291	0.365397	3.029183
C	-1.018338	4.504055	1.673902
C	2.446573	-3.774171	-0.677912
C	1.526614	5.565698	-3.337557
H	2.529089	5.164749	-3.486254
H	1.596890	6.618017	-3.051923
H	0.984977	5.517357	-4.287733
C	-2.878843	-3.422232	3.098842
C	2.740239	-0.253990	1.665983
C	-2.157591	3.936702	2.263991
C	2.477331	2.419833	-3.561757
C	0.523194	0.226676	0.561694
C	-2.488683	4.312076	3.569323
H	-3.367599	3.874197	4.035272
C	3.642198	1.713743	-3.881493
H	3.721002	1.244906	-4.859319
C	0.819328	-5.423786	-0.209732
C	2.395059	3.014033	-2.285694
C	-2.120947	6.225028	-0.186342
H	-3.032743	5.666645	0.048958
H	-2.329220	6.918464	-1.002056
H	-1.856696	6.794861	0.707693
C	0.759904	4.812648	-2.281175
C	4.938233	-0.186185	2.693135
H	5.548606	-0.106576	3.589501
C	3.550096	-0.158900	2.800482
H	3.090322	-0.066881	3.780727
C	-0.199321	5.394262	2.403004
H	0.424634	-2.598444	-2.006239

C	-0.268418	-5.991011	0.466773
H	-0.466877	-7.036271	0.265332
C	1.664866	-6.392716	-1.004116
H	2.156112	-5.929050	-1.858959
H	1.049165	-7.227118	-1.345991
H	2.446841	-6.799133	-0.353182
C	3.619075	-4.713679	1.412483
H	2.607549	-5.080494	1.622503
C	-0.738110	-3.663265	4.282411
C	1.209328	-0.387015	1.794660
C	3.355401	-0.387987	0.420518
H	2.744060	-0.462285	-0.477101
C	0.914629	1.206774	-5.159711
H	1.739638	0.567027	-5.490262
H	0.238153	1.350394	-6.006656
H	0.365758	0.672701	-4.382383
C	3.486227	2.965393	-1.391259
C	0.528913	2.362432	4.397050
H	0.826250	3.390757	4.588333
C	2.476858	-3.175881	-1.950914
C	-1.031042	5.256868	-0.576663
C	3.624499	-4.070869	0.030558
C	-0.766679	0.425940	4.977184
H	-1.480718	-0.085281	5.619481
C	4.742412	-0.425352	0.310437
H	5.193412	-0.533579	-0.672447
C	3.724585	-2.878628	-2.502931
H	3.779745	-2.415542	-3.483577
C	-3.721555	-3.514927	1.835272
H	-3.179926	-4.124034	1.105127
C	1.101025	5.968890	1.853555
H	1.327162	5.459193	0.912017
C	4.622991	2.252579	-1.764345
H	5.461188	2.189972	-1.078279
C	4.401641	3.211948	0.990547
H	4.160422	2.169197	1.211715
H	4.275748	3.795939	1.907708
H	5.461146	3.274072	0.717486
C	0.753315	-3.984632	4.291516
H	1.155216	-3.686071	3.320277
C	-0.975908	-5.411995	1.522002
C	-3.982881	0.452899	-0.748502
C	-3.036123	2.908256	1.573242
H	-2.707578	2.804785	0.532115
C	1.988261	3.342175	-5.856435
H	2.467261	4.280720	-5.566557
H	1.186874	3.568801	-6.567725
H	2.738485	2.743586	-6.385023
C	-0.406017	5.458162	-1.802816
H	-0.742710	6.309652	-2.382913
C	-0.589682	5.761104	3.690282
H	0.016744	6.469437	4.249973
C	-0.241309	-0.247889	3.875817
H	-0.549677	-1.264125	3.667520
C	-3.470322	-3.024340	4.299262
H	-4.526808	-2.769792	4.315144
C	4.899752	-3.167067	-1.820963
H	5.861130	-2.930771	-2.270690
C	3.887946	5.223448	-0.438839
H	4.874061	5.244112	-0.916632
H	3.939962	5.838317	0.465089
H	3.174573	5.694255	-1.123840

C	1.013886	-5.486069	4.471918
H	0.570443	-5.852126	5.405804
H	2.092479	-5.679571	4.513605
H	0.607059	-6.077839	3.645348
C	1.528461	-3.181123	5.339508
H	1.308665	-2.111818	5.260274
H	2.603495	-3.317372	5.184425
H	1.304247	-3.509623	6.361267
C	-1.876853	-6.333080	2.311656
H	-1.666775	-6.267365	3.381960
H	-1.743504	-7.365334	1.986258
H	-2.926681	-6.058162	2.175449
C	-1.383971	-3.288961	5.461972
H	-0.816206	-3.231394	6.385860
C	-1.732973	5.233053	4.273460
H	-2.023502	5.529116	5.278091
C	3.494220	3.776411	-0.102835
H	2.478746	3.785349	0.307904
C	4.845550	-3.750877	-0.564584
H	5.769204	-3.960004	-0.030561
C	-4.508999	3.336185	1.550453
H	-4.931851	3.376454	2.560436
H	-5.101578	2.620821	0.973880
H	-4.635369	4.326091	1.097649
C	5.541279	-0.321625	1.445762
H	6.625055	-0.344342	1.359422
C	-0.387807	1.736623	5.239684
H	-0.801091	2.272668	6.090667
C	4.697338	1.609330	-2.991951
H	5.587801	1.047340	-3.262029
C	-2.851259	1.555741	2.275013
H	-1.828500	1.185690	2.153430
H	-3.537912	0.805876	1.878888
H	-3.035846	1.645510	3.351134
C	2.257160	5.696926	2.824822
C	3.962187	-3.677947	2.488721
H	4.942589	-3.225833	2.299442
H	3.992787	-4.153513	3.477018
H	3.223472	-2.871608	2.509987
C	-3.910957	-2.136875	1.205041
H	-4.419580	-1.457145	1.897918
H	-4.526065	-2.216812	0.302756
H	-2.951029	-1.692436	0.918473
C	-5.077949	-4.186553	2.080328
H	-4.976199	-5.148537	2.595489
H	-5.585162	-4.361385	1.126114
H	-5.737845	-3.555234	2.685018
C	-2.734155	-2.962309	5.473051
H	-3.212964	-2.661157	6.401415
C	4.570068	-5.914137	1.500822
H	4.395471	-6.641772	0.700705
H	4.439134	-6.425251	2.460602
H	5.618218	-5.601717	1.439599
C	0.995708	7.468432	1.549185
H	0.238217	7.674732	0.786113
H	1.953593	7.850463	1.178519
H	0.732748	8.036855	2.448661
C	1.194870	-2.896466	-2.726065
C	0.672678	-4.134352	-3.469744
H	1.441574	-4.542862	-4.137316
H	-0.196534	-3.858948	-4.075558
H	0.350909	-4.925820	-2.788476

C 1.349914 -1.748348 -3.719318
H 2.105648 6.204849 3.782985
H 3.204875 6.060845 2.414859
H 2.359511 4.625033 3.027095
H 1.967298 -2.028727 -4.582078
H 1.800495 -0.863127 -3.255759
H 0.364196 -1.474441 -4.107328
H -3.766308 2.323428 -2.383228
C -0.789901 0.366853 -1.517484
H -0.021400 0.316014 -2.299981

14_TS6.log

SCF (wB97x) = -3920.40740769
E(SCF)+ZPE(0 K)= -3918.828994
H(298 K)= -3918.731882
G(298 K)= -3918.959846
Lowest Frequency = -101.9528cm-1

W -2.788004 0.588272 -2.139621
Al 0.062956 2.710321 -0.461696
Al 0.220496 -2.837057 0.877701
O -0.608410 -1.719055 -0.198909
O -1.219948 1.688105 -1.161257
O 1.027808 -1.771852 1.981374
O 0.993916 1.527265 0.398981
C -3.715670 0.275746 -3.870627
N 1.125675 3.590373 -1.820065
N -0.803178 -4.109346 1.869426
C -2.969155 -1.406226 -2.093127
N -0.692984 4.263123 0.292503
N 1.247704 -4.148806 -0.089659
C -1.922491 2.665210 -3.960827
O -4.191335 0.145664 -4.919487
O -3.115184 -2.551901 -2.058144
O -5.353157 0.448942 -0.368604
O -2.302588 3.537046 -4.583671
C 1.118148 1.662789 3.367806
H 1.915425 2.122546 2.788678
C -1.449471 -3.682475 3.090087
C -0.221085 -0.407941 -0.274158
C 1.314503 2.528197 -4.654410
H 0.525659 3.168105 -4.249840
C 0.693023 0.370156 3.040047
C -1.048243 4.477097 1.680559
C 2.500935 -3.768156 -0.701425
C 1.510849 5.500546 -3.353428
H 2.496711 5.066443 -3.519188
H 1.622949 6.548922 -3.066469
H 0.953668 5.475967 -4.295773
C -2.803056 -3.307478 3.068179
C 2.810884 -0.260035 1.749505
C -2.224436 3.927909 2.213250
C 2.364702 2.317781 -3.567175
C 0.621990 0.214106 0.582907
C -2.569874 4.249917 3.527883
H -3.473901 3.823890 3.955389
C 3.483979 1.542448 -3.886519
H 3.523365 1.047231 -4.854254
C 0.891713 -5.432507 -0.186893
C 2.326223 2.932406 -2.298580
C -2.081174 6.252442 -0.158672

H	-3.007551	5.720608	0.081726
H	-2.275180	6.956591	-0.968625
H	-1.794474	6.808649	0.737023
C	0.736879	4.771406	-2.283779
C	4.983414	-0.190272	2.830299
H	5.571428	-0.103592	3.740794
C	3.593351	-0.159334	2.902927
H	3.111062	-0.057132	3.871163
C	-0.218853	5.312286	2.457015
H	0.414794	-2.635420	-1.974141
C	-0.197898	-5.991839	0.492955
H	-0.403083	-7.036472	0.294921
C	1.731214	-6.402756	-0.985725
H	2.198024	-5.942853	-1.856530
H	1.120061	-7.249259	-1.304708
H	2.533539	-6.790044	-0.347587
C	3.743914	-4.724558	1.337160
H	2.755514	-5.150303	1.544546
C	-0.693601	-3.654330	4.279050
C	1.277521	-0.390568	1.835931
C	3.456698	-0.411461	0.521597
H	2.868039	-0.494202	-0.390463
C	0.649154	1.233766	-5.128347
H	1.387042	0.525031	-5.519419
H	-0.060321	1.450292	-5.935321
H	0.100530	0.739475	-4.324871
C	3.428943	2.860838	-1.420037
C	0.540616	2.354040	4.426673
H	0.883411	3.357492	4.665842
C	2.484939	-3.148555	-1.965442
C	-1.020232	5.257378	-0.561078
C	3.703820	-4.054150	-0.031042
C	-0.912722	0.491564	4.854368
H	-1.707557	0.015399	5.424244
C	4.846262	-0.456002	0.446928
H	5.321140	-0.580924	-0.522863
C	3.711183	-2.807334	-2.540386
H	3.730044	-2.322878	-3.512276
C	-3.635619	-3.333713	1.794992
H	-3.079716	-3.883576	1.028444
C	1.096742	5.882810	1.939938
H	1.307725	5.424212	0.968562
C	4.524799	2.087583	-1.796478
H	5.372188	2.002817	-1.124478
C	4.434176	3.154046	0.922788
H	4.206460	2.116545	1.178187
H	4.343551	3.758953	1.830615
H	5.481123	3.213803	0.603783
C	0.779467	-4.044766	4.317062
H	1.175655	-3.956553	3.301959
C	-0.926244	-5.396881	1.526045
C	-4.395672	0.476151	-1.025619
C	-3.115592	2.981249	1.428632
H	-2.797701	2.989907	0.379990
C	1.932488	3.241050	-5.869296
H	2.482771	4.144141	-5.592325
H	1.150291	3.520407	-6.583649
H	2.632162	2.578621	-6.390355
C	-0.406966	5.444390	-1.797495
H	-0.724285	6.309265	-2.368712
C	-0.624188	5.630934	3.752834
H	-0.008628	6.294337	4.356495

C	-0.333144	-0.198745	3.791381
H	-0.679978	-1.194314	3.544016
C	-3.389771	-2.886955	4.263760
H	-4.432234	-2.578325	4.264946
C	4.909609	-3.079635	-1.893421
H	5.853539	-2.809590	-2.361064
C	3.847237	5.142331	-0.498702
H	4.821508	5.175159	-0.999706
H	3.912629	5.759315	0.403955
H	3.111563	5.603888	-1.165256
C	0.958783	-5.502049	4.761341
H	0.540606	-5.661622	5.762290
H	2.023254	-5.761719	4.795013
H	0.468365	-6.200787	4.074696
C	1.609628	-3.101440	5.192102
H	1.456846	-2.060581	4.892006
H	2.673795	-3.334744	5.083619
H	1.361467	-3.199926	6.255082
C	-1.875832	-6.289440	2.287595
H	-1.712678	-6.215340	3.365522
H	-1.756845	-7.328278	1.978041
H	-2.910313	-5.985992	2.100182
C	-1.334658	-3.255212	5.452307
H	-0.775022	-3.229048	6.383200
C	-1.793549	5.109141	4.288707
H	-2.092631	5.364992	5.301933
C	3.480624	3.692972	-0.143730
H	2.481854	3.695527	0.306381
C	4.901433	-3.694809	-0.650532
H	5.843326	-3.894383	-0.145765
C	-4.590646	3.398470	1.459928
H	-5.013434	3.317090	2.467456
H	-5.176184	2.745148	0.806011
H	-4.727871	4.432822	1.124288
C	5.617347	-0.339699	1.599715
H	6.702839	-0.365973	1.540749
C	-0.479348	1.771751	5.175998
H	-0.930688	2.317994	6.000740
C	4.543921	1.404941	-3.005874
H	5.398968	0.788120	-3.270922
C	-2.935420	1.555667	1.962179
H	-1.908199	1.199261	1.826853
H	-3.609550	0.866376	1.449267
H	-3.154689	1.509912	3.035473
C	2.253071	5.528123	2.882722
C	4.034413	-3.695737	2.435395
H	4.988734	-3.187974	2.254404
H	4.092374	-4.191623	3.412367
H	3.253664	-2.930155	2.477885
C	-3.849281	-1.917369	1.259768
H	-4.356321	-1.291159	2.003352
H	-4.474501	-1.946447	0.362429
H	-2.895379	-1.447220	0.996227
C	-4.983529	-4.038383	1.993480
H	-4.870999	-5.032278	2.441084
H	-5.490039	-4.152439	1.029834
H	-5.648140	-3.458242	2.642917
C	-2.668857	-2.868611	5.448703
H	-3.145953	-2.551083	6.372557
C	4.756338	-5.875303	1.392792
H	4.613088	-6.591146	0.575698
H	4.657714	-6.414786	2.340609

H	5.786966	-5.508866	1.334713
C	1.025599	7.399964	1.725700
H	0.263082	7.670620	0.988136
H	1.987765	7.779885	1.364251
H	0.789106	7.920489	2.660738
C	1.180128	-2.896325	-2.711882
C	0.691542	-4.141836	-3.465782
H	1.465411	-4.516253	-4.147414
H	-0.192650	-3.892148	-4.061916
H	0.405098	-4.952008	-2.790604
C	1.273507	-1.724942	-3.687119
H	2.117561	5.977176	3.872626
H	3.203775	5.900431	2.485592
H	2.338058	4.444153	3.014185
H	1.870768	-1.971647	-4.574477
H	1.714835	-0.837545	-3.218917
H	0.268069	-1.467847	-4.035600
H	-3.904652	1.948692	-2.408570
C	-0.740121	0.347619	-1.469942
H	0.018339	0.412277	-2.263595

15_Int6.log

SCF (wB97x) = -3807.12573523

E(SCF)+ZPE(0 K)= -3805.554191

H(298 K)= -3805.459324

G(298 K)= -3805.682702

Lowest Frequency = 14.4480cm-1

W	-2.727916	0.564764	-2.222279
Al	0.086041	2.619361	-0.566979
Al	0.274660	-2.957732	0.793824
O	-0.543375	-1.852752	-0.298444
O	-1.055585	1.557245	-1.440484
O	1.022091	-1.876153	1.923150
O	0.949553	1.440287	0.365106
C	-4.561929	0.399558	-1.490633
N	1.105479	3.580858	-1.876452
N	-0.806460	-4.221224	1.727893
C	-3.089387	-1.346945	-2.741196
N	-0.748111	4.115355	0.210100
N	1.325564	-4.263530	-0.150316
C	-3.223355	0.873838	-4.096684
O	-5.603838	0.334209	-0.984084
O	-3.289479	-2.442809	-3.046067
O	-3.432985	1.096367	-5.217367
C	1.230051	1.493470	3.396442
H	2.086501	1.912027	2.874037
C	-1.540445	-3.757255	2.882730
C	-0.201794	-0.528566	-0.345942
C	1.014356	2.508663	-4.678439
H	0.263252	3.142123	-4.195612
C	0.713586	0.262553	2.982919
C	-1.108838	4.248114	1.605247
C	2.597671	-3.892211	-0.731117
C	1.454939	5.506071	-3.393614
H	2.421984	5.054254	-3.614466
H	1.603930	6.544263	-3.086005
H	0.855579	5.518245	-4.309578
C	-2.881451	-3.359240	2.739917
C	2.809956	-0.376933	1.639885
C	-2.289992	3.672450	2.097001

C	2.171095	2.293022	-3.703408	C	0.301826	1.215975	-5.087913
C	0.604008	0.114593	0.528810	H	0.989900	0.515530	-5.574709
C	-2.637900	3.912010	3.427867	H	-0.504022	1.441034	-5.794783
H	-3.546470	3.467184	3.826129	H	-0.145246	0.708683	-4.231021
C	3.254659	1.510557	-4.114771	C	3.403356	2.793525	-1.627899
H	3.214266	1.020251	-5.085052	C	0.670938	2.174922	4.472244
C	0.970067	-5.548432	-0.256325	H	1.092828	3.127279	4.783701
C	2.251632	2.902226	-2.435637	C	2.626585	-3.267995	-1.993404
C	-2.127945	6.131671	-0.132213	C	-1.058118	5.166732	-0.580970
H	-3.061855	5.585822	0.037872	C	3.777469	-4.204047	-0.030292
H	-2.297885	6.896918	-0.890477	C	-0.964458	0.435429	4.725496
H	-1.867300	6.615771	0.811934	H	-1.826112	0.006388	5.232209
C	0.707691	4.768813	-2.311276	C	4.799947	-0.559969	0.264560
C	5.017991	-0.369385	2.648840	H	5.240706	-0.659928	-0.723858
H	5.636475	-0.319035	3.541778	C	3.875602	-2.960963	-2.538523
C	3.631497	-0.324189	2.769014	H	3.930546	-2.478861	-3.510055
H	3.183260	-0.251031	3.756137	C	-3.625053	-3.452717	1.414170
C	-0.278178	5.033694	2.429501	H	-3.010187	-4.030137	0.716146
H	0.578974	-2.672779	-2.054226	C	1.028790	5.647368	1.937117
C	-0.132668	-6.111189	0.398551	H	1.221426	5.275210	0.925415
H	-0.324646	-7.158620	0.202050	C	4.455217	2.006330	-2.091197
C	1.824577	-6.514658	-1.044619	H	5.345053	1.890666	-1.480553
H	2.276817	-6.056124	-1.924054	C	4.532518	3.017862	0.669699
H	1.228118	-7.376282	-1.350018	H	4.307332	1.978791	0.919591
H	2.639665	-6.877602	-0.408360	H	4.497508	3.607211	1.592115
C	3.762556	-4.874295	1.339220	H	5.560953	3.072677	0.293899
H	2.779768	-5.334819	1.488979	C	0.563596	-4.153839	4.310142
C	-0.880877	-3.702441	4.125981	H	1.015316	-4.262419	3.319005
C	1.277712	-0.494542	1.768832	C	-0.907640	-5.511545	1.395296
C	3.414751	-0.497439	0.387274	C	-3.182988	2.799945	1.232537
H	2.795942	-0.540441	-0.507065	H	-2.857062	2.901028	0.191022

C	1.511023	3.221594	-5.947523	H	-1.112239	-3.186236	6.198227
H	2.083482	4.126450	-5.725920	C	-1.856892	4.718425	4.241388
H	0.663544	3.496531	-6.584442	H	-2.156215	4.910580	5.268613
H	2.159805	2.559421	-6.531520	C	3.526990	3.587312	-0.330424
C	-0.421732	5.434542	-1.787792	H	2.552606	3.571152	0.171797
H	-0.734158	6.327311	-2.316921	C	4.998123	-3.872837	-0.619082
C	-0.684755	5.270071	3.742280	H	5.922749	-4.094050	-0.092575
H	-0.068604	5.892704	4.387797	C	-4.656201	3.219721	1.282669
C	-0.399205	-0.246726	3.651320	H	-5.091713	3.052478	2.274176
H	-0.823393	-1.191460	3.330985	H	-5.234982	2.628649	0.565221
C	-3.545054	-2.868826	3.866409	H	-4.786147	4.279474	1.036155
H	-4.577450	-2.540205	3.774275	C	5.609576	-0.490639	1.394426
C	5.050821	-3.263268	-1.863639	H	6.692021	-0.529871	1.299090
H	6.011980	-3.018599	-2.309462	C	-0.428252	1.647640	5.144165
C	3.870808	5.050714	-0.646297	H	-0.865280	2.184412	5.982660
H	4.826467	5.110701	-1.179704	C	4.375497	1.350494	-3.314510
H	3.963958	5.632526	0.277576	H	5.200347	0.727611	-3.651417
H	3.108872	5.531748	-1.267203	C	-3.025548	1.329283	1.631732
C	0.624382	-5.522904	5.000710	H	-1.995283	0.976745	1.504379
H	0.168588	-5.479920	5.996813	H	-3.684508	0.701077	1.025168
H	1.665260	-5.843520	5.120807	H	-3.289881	1.176620	2.685193
H	0.101098	-6.294865	4.425969	C	2.210079	5.218776	2.815156
C	1.402585	-3.123858	5.072064	C	3.949268	-3.836080	2.451249
H	1.374421	-2.156095	4.564959	H	4.897179	-3.299840	2.328039
H	2.445086	-3.456609	5.121205	H	3.959208	-4.328380	3.431777
H	1.051485	-2.991842	6.101740	H	3.144956	-3.093685	2.442645
C	-1.889176	-6.397659	2.121411	C	-3.823933	-2.069021	0.792973
H	-1.798518	-6.286421	3.204509	H	-4.414826	-1.426227	1.456126
H	-1.735088	-7.443264	1.852942	H	-4.360247	-2.155916	-0.158352
H	-2.913865	-6.117891	1.857494	H	-2.859772	-1.588882	0.596697
C	-1.598522	-3.233745	5.227196	C	-4.972716	-4.171453	1.558861

H	-4.873726	-5.144161	2.053488
H	-5.418884	-4.332602	0.572306
H	-5.682731	-3.576448	2.143511
C	-2.914807	-2.809438	5.101506
H	-3.451929	-2.436379	5.970027
C	4.806590	-5.990829	1.459066
H	4.739924	-6.707660	0.632858
H	4.662858	-6.536816	2.397174
H	5.826644	-5.592029	1.471444
C	0.949361	7.177166	1.855324
H	0.164398	7.507347	1.167244
H	1.899893	7.589687	1.499178
H	0.740898	7.615330	2.838000
C	1.348346	-2.974300	-2.772688
C	0.823371	-4.212553	-3.514474
H	1.591525	-4.625827	-4.179962
H	-0.043246	-3.939725	-4.125990
H	0.498344	-5.000779	-2.830773
C	1.507176	-1.826044	-3.770276
H	2.092586	5.567674	3.847300
H	3.145629	5.642248	2.432281
H	2.310816	4.129076	2.835987
H	2.129018	-2.109378	-4.629040
H	1.952059	-0.935916	-3.309964
H	0.522639	-1.553258	-4.163493
H	-3.656813	2.065462	-2.321784
C	-0.696049	0.152280	-1.586280
H	0.034338	0.049480	-2.396884

16_TS7.log

SCF (wB97x) = -3807.12037582

E(SCF)+ZPE(0 K)= -3805.549016

H(298 K)= -3805.455034

G(298 K)= -3805.675538

Lowest Frequency = -12.6039cm-1

W	-2.983863	11.378410	10.945362
Al	-0.325705	13.464547	12.839170
Al	-0.155724	7.903551	14.193657
O	-0.981555	9.016251	13.115324
O	-1.510088	12.411284	12.011278
O	0.617500	8.970902	15.316978
O	0.559528	12.287001	13.758401
C	-4.527391	12.695711	10.896035
N	0.728210	14.417463	11.544145
N	-1.172692	6.632789	15.189380
C	-4.483632	10.145196	11.304338
N	-1.166754	14.974439	13.590259
N	0.861969	6.604095	13.202717
C	-2.968527	10.146830	9.387048
O	-5.377531	13.477957	10.863191
O	-5.369716	9.426586	11.534018
O	-2.888559	9.447584	8.467361
C	0.719911	12.333198	16.818324
H	1.580927	12.771731	16.320207
C	-1.798029	7.044603	16.426764
C	-0.590552	10.325874	13.040884
C	0.766365	13.370225	8.727833
H	-0.005835	14.000810	9.180692
C	0.243119	11.092275	16.387932

C	-1.587413	15.092120	14.969055	H	1.770783	4.822869	11.402694
C	2.117854	6.979429	12.589900	H	0.696303	3.516051	11.959687
C	1.139485	16.350065	10.050504	H	2.126232	3.961395	12.899271
H	2.115570	15.900915	9.867628	C	3.347342	5.995638	14.624606
H	1.273375	17.389236	10.361266	H	2.352898	5.584594	14.832213
H	0.575892	16.357996	9.112031	C	-1.027407	7.053647	17.605751
C	-3.149905	7.426918	16.431471	C	0.852834	10.356526	15.183670
C	2.386533	10.489221	15.094521	C	3.022979	10.348753	13.859348
C	-2.796303	14.520623	15.394681	H	2.427451	10.286666	12.950225
C	1.875548	13.144318	9.754341	C	0.073237	12.082819	8.273097
C	0.205361	10.964328	13.929041	H	0.781765	11.386431	7.810759
C	-3.195129	14.725639	16.716730	H	-0.697671	12.316822	7.530817
H	-4.124217	14.281886	17.065869	H	-0.414697	11.567434	9.101740
C	2.974854	12.362130	9.386398	C	3.021468	13.644025	11.878038
H	2.974733	11.874483	8.413933	C	0.115985	12.998543	17.880012
C	0.493156	5.324840	13.095358	H	0.509004	13.958001	18.206758
C	1.900405	13.746972	11.027489	C	2.112760	7.611285	11.330072
C	-2.520752	17.002118	13.212428	C	-1.448459	16.026960	12.791950
H	-3.470937	16.470220	13.329122	C	3.316173	6.675331	13.260881
H	-2.644573	17.784786	12.463143	C	-1.487640	11.223268	18.082687
H	-2.295636	17.462484	14.177086	H	-2.352428	10.772856	18.564652
C	0.353872	15.611834	11.104937	C	4.410878	10.286011	13.772877
C	4.568417	10.513927	16.158746	H	4.876363	10.168229	12.797607
H	5.164012	10.577827	17.066215	C	3.346810	7.941793	10.764925
C	3.179062	10.559774	16.243138	H	3.376681	8.433409	9.797451
H	2.706092	10.646810	17.217455	C	-3.986320	7.477617	15.162163
C	-0.787403	15.854186	15.843455	H	-3.452169	6.938543	14.372651
H	0.067472	8.219980	11.307434	C	0.535387	16.480883	15.413182
C	-0.596233	4.766718	13.778751	H	0.766860	16.128788	14.402333
H	-0.811345	3.726372	13.568964	C	4.093304	12.861027	11.454975
C	1.315876	4.357090	12.276684	H	4.959375	12.748552	12.099197

C	4.072995	13.872467	14.210701
H	3.854348	12.828436	14.444659
H	3.998208	14.453723	15.135903
H	5.112649	13.945088	13.870464
C	0.448389	6.676544	17.624436
H	0.820591	6.717766	16.597177
C	-1.308162	5.351049	14.829685
C	-3.671118	13.701411	14.461013
H	-3.294145	13.836771	13.440689
C	1.324571	14.093968	7.490655
H	1.890237	14.993814	7.746927
H	0.509137	14.379244	6.817270
H	1.996792	13.434054	6.931229
C	-0.783182	16.286603	11.598834
H	-1.071761	17.184463	11.064866
C	-1.245794	16.059767	17.144384
H	-0.653628	16.662997	17.829582
C	-0.873901	10.553995	17.027139
H	-1.262878	9.596605	16.700682
C	-3.723915	7.812722	17.644826
H	-4.766199	8.121195	17.665726
C	4.540299	7.651855	11.413505
H	5.488326	7.915821	10.950788
C	3.434672	15.904066	12.883594
H	4.406454	15.976284	12.381751
H	3.492629	16.480725	13.813722
H	2.688740	16.382759	12.241910
C	0.650890	5.244377	18.134492
H	0.269076	5.132730	19.156184
H	1.716928	4.989100	18.143210
H	0.137998	4.511687	17.501239
C	1.280309	7.674734	18.434976
H	1.110118	8.693318	18.074282
H	2.346035	7.447386	18.330166
H	1.041383	7.635846	19.503738
C	-2.249493	4.454435	15.596210
H	-2.030371	4.479259	16.666982
H	-2.176516	3.426422	15.239982
H	-3.282306	4.795466	15.476136
C	-1.653630	7.424328	18.796139
H	-1.080462	7.431593	19.719245
C	-2.438838	15.501094	17.582545
H	-2.776417	15.666811	18.602445
C	3.094462	14.435907	13.180742
H	2.104715	14.408083	13.650986
C	4.520835	7.027354	12.650757
H	5.458054	6.813225	13.158275
C	-5.134336	14.158403	14.470090
H	-5.617665	13.946128	15.430135
H	-5.696648	13.630447	13.692906
H	-5.226997	15.233638	14.281168
C	5.191720	10.375113	14.921747
H	6.276293	10.335173	14.854723
C	-0.991498	12.446829	18.517621
H	-1.464612	12.972321	19.343630
C	4.062596	12.202748	10.230946
H	4.900737	11.580263	9.927832
C	-3.566866	12.212228	14.799304
H	-2.536141	11.845555	14.729356
H	-4.187793	11.623356	14.113857
H	-3.911505	12.018278	15.822327
C	1.687413	16.043708	16.324836

C	3.657758	7.014869	15.726048
H	4.621484	7.504705	15.546002
H	3.706723	6.515170	16.701466
H	2.892080	7.795610	15.771921
C	-4.134937	8.928962	14.699251
H	-4.586159	9.540036	15.490024
H	-4.775169	8.983775	13.813988
H	-3.160086	9.357183	14.442254
C	-5.360714	6.819391	15.329296
H	-5.286000	5.799742	15.724093
H	-5.871973	6.775635	14.362540
H	-6.001896	7.390499	16.009604
C	-2.989560	7.801390	18.821518
H	-3.455940	8.092631	19.759348
C	4.341186	4.828435	14.672919
H	4.184618	4.118564	13.853132
H	4.235748	4.286350	15.618546
H	5.377528	5.178177	14.614000
C	0.450828	18.011834	15.358368
H	-0.310301	18.353134	14.649393
H	1.411557	18.435363	15.045219
H	0.205000	18.429083	16.341527
C	0.816339	7.895813	10.576896
C	0.259526	6.648944	9.872886
H	1.010213	6.206272	9.206268
H	-0.608924	6.926352	9.265899
H	-0.071783	5.882853	10.578128
C	0.960892	9.016552	9.547344
H	1.532348	16.374767	17.357966
H	2.632715	16.479275	15.981365
H	1.795209	14.954800	16.330753
H	1.553826	8.701296	8.679086
H	1.432957	9.909844	9.972571
H	-0.030418	9.293126	9.175873
H	-3.173649	12.188387	9.444658
C	-1.017553	11.033201	11.786128
H	-0.179592	11.087139	11.079014

17_Int7.log

SCF (wB97x) = -3807.12157481

E(SCF)+ZPE(0 K)= -3805.549824

H(298 K)= -3805.454893

G(298 K)= -3805.679146

Lowest Frequency = 9.6642cm-1

W	-2.706295	0.584289	-2.332136
Al	0.057403	2.600777	-0.546923
Al	0.311997	-2.979453	0.769089
O	-0.521134	-1.878704	-0.315249
O	-1.076921	1.548884	-1.440015
O	1.052466	-1.898065	1.903296
O	0.942823	1.423690	0.360342
C	-3.738455	2.142161	-3.122190
N	1.055297	3.589654	-1.856283
N	-0.769439	-4.258689	1.680663
C	-4.415737	0.466929	-1.384367
N	-0.785898	4.078659	0.255819
N	1.385969	-4.270027	-0.170448
C	-3.319998	-1.225094	-2.919462
O	-4.276994	3.039168	-3.612466
O	-5.414852	0.396299	-0.785988

O	-3.639054	-2.278466	-3.270876	H	5.673684	-0.321747	3.475152
C	1.258947	1.469542	3.383695	C	3.660406	-0.337026	2.724261
H	2.098919	1.900383	2.844640	H	3.221954	-0.281685	3.716990
C	-1.534299	-3.797601	2.816320	C	-0.286290	5.004330	2.465865
C	-0.201928	-0.549446	-0.354037	H	0.649434	-2.673207	-2.064876
C	0.961243	2.539182	-4.667837	C	-0.059855	-6.135388	0.352312
H	0.196653	3.144706	-4.170035	H	-0.239056	-7.183770	0.148840
C	0.747179	0.234546	2.975849	C	1.913640	-6.510497	-1.075914
C	-1.120295	4.204136	1.658590	H	2.364006	-6.042701	-1.951518
C	2.662041	-3.886429	-0.734176	H	1.329439	-7.378291	-1.387436
C	1.354355	5.536216	-3.357340	H	2.730809	-6.865624	-0.437963
H	2.324749	5.101158	-3.596204	C	3.809481	-4.874653	1.342587
H	1.493427	6.571028	-3.033921	H	2.831278	-5.350129	1.474235
H	0.744027	5.556426	-4.265860	C	-0.908845	-3.744344	4.077002
C	-2.870124	-3.396158	2.635991	C	1.296990	-0.514039	1.749681
C	2.827001	-0.383007	1.603624	C	3.419093	-0.477991	0.342929
C	-2.279801	3.607775	2.175769	H	2.789668	-0.513346	-0.544234
C	2.130656	2.339967	-3.704937	C	0.283210	1.232130	-5.090296
C	0.605650	0.093706	0.519027	H	0.986349	0.559784	-5.594768
C	-2.604361	3.844659	3.513299	H	-0.535438	1.444168	-5.786472
H	-3.495513	3.383109	3.931162	H	-0.139406	0.696695	-4.238525
C	3.225968	1.584578	-4.135064	C	3.369193	2.839137	-1.634267
H	3.187343	1.105430	-5.110938	C	0.717087	2.139015	4.475715
C	1.043121	-5.557822	-0.288296	H	1.134687	3.094888	4.782271
C	2.209047	2.937077	-2.431118	C	2.701426	-3.254097	-1.991818
C	-2.202596	6.074316	-0.057136	C	-1.125124	5.127484	-0.526122
H	-3.121969	5.512976	0.139156	C	3.836280	-4.197957	-0.023662
H	-2.404625	6.832332	-0.814816	C	-0.890946	0.378767	4.758486
H	-1.927830	6.568198	0.877974	H	-1.735761	-0.062948	5.282536
C	0.629546	4.772119	-2.278325	C	4.803392	-0.523213	0.204010
C	5.045860	-0.365919	2.588388	H	5.234429	-0.602804	-0.790516

C	3.954464	-2.944423	-2.525908
H	4.016724	-2.458247	-3.495056
C	-3.586367	-3.512780	1.296780
H	-2.944762	-4.079192	0.613987
C	1.004115	5.633634	1.950465
H	1.187742	5.257279	0.938660
C	4.432022	2.077575	-2.115077
H	5.328380	1.971494	-1.512326
C	4.509869	3.052627	0.657890
H	4.299567	2.007535	0.896052
H	4.472482	3.630152	1.587570
H	5.535416	3.125379	0.277320
C	0.523691	-4.214502	4.305026
H	0.991596	-4.368989	3.327063
C	-0.854129	-5.547627	1.341482
C	-3.178606	2.714967	1.337845
H	-2.870067	2.805009	0.289288
C	1.427454	3.282027	-5.931371
H	1.977750	4.198522	-5.702639
H	0.567824	3.543498	-6.557735
H	2.088475	2.644921	-6.529332
C	-0.511036	5.409572	-1.741814
H	-0.846464	6.298847	-2.262800
C	-0.670799	5.237852	3.785587
H	-0.052380	5.872106	4.417379
C	-0.343829	-0.290388	3.666921
H	-0.764925	-1.237566	3.350191
C	-3.558917	-2.892475	3.741204
H	-4.586633	-2.558579	3.619683
C	5.124125	-3.248291	-1.842450
H	6.088859	-3.000952	-2.278938
C	3.817018	5.090361	-0.637030
H	4.767088	5.164372	-1.178586
H	3.912346	5.666681	0.289875
H	3.044304	5.567297	-1.247971
C	0.543068	-5.557903	5.047060
H	0.077477	-5.466361	6.035273
H	1.574240	-5.898564	5.192130
H	0.007011	-6.338408	4.496247
C	1.368852	-3.174816	5.047077
H	1.393149	-2.233740	4.492294
H	2.396612	-3.538990	5.153849
H	0.983066	-2.979071	6.053941
C	-1.838892	-6.445033	2.048340
H	-1.776086	-6.327681	3.132662
H	-1.663947	-7.489443	1.788066
H	-2.860463	-6.180385	1.758120
C	-1.652166	-3.263843	5.156076
H	-1.192136	-3.216902	6.140075
C	-1.823114	4.667615	4.309161
H	-2.105578	4.857095	5.341632
C	3.491481	3.620852	-0.329948
H	2.520395	3.589179	0.177696
C	5.061348	-3.861335	-0.599986
H	5.981731	-4.082667	-0.066270
C	-4.653953	3.125517	1.413107
H	-5.062691	2.976135	2.418640
H	-5.245782	2.514153	0.724707
H	-4.797644	4.179588	1.149583
C	5.624697	-0.462625	1.325907
H	6.706370	-0.489045	1.218102
C	-0.359220	1.594928	5.171121

H -0.782014 2.121848 6.023083
C 4.356244 1.436421 -3.346139
H 5.190468 0.834467 -3.697528
C -2.998825 1.251216 1.754467
H -1.972620 0.903541 1.588983
H -3.677783 0.610117 1.186755
H -3.216692 1.118004 2.820894
C 2.202256 5.226507 2.816033
C 3.962340 -3.838686 2.461954
H 4.903390 -3.286955 2.355031
H 3.964945 -4.334992 3.440466
H 3.146627 -3.109006 2.443767
C -3.815710 -2.141702 0.659057
H -4.449146 -1.515211 1.297635
H -4.323150 -2.256075 -0.305201
H -2.865324 -1.628349 0.482071
C -4.917303 -4.265917 1.426596
H -4.802270 -5.232493 1.929693
H -5.345170 -4.444172 0.434853
H -5.649743 -3.684622 1.997261
C -2.960319 -2.827485 4.991816
H -3.517142 -2.443463 5.842989
C 4.867358 -5.976500 1.475299
H 4.826143 -6.689604 0.644178
H 4.714463 -6.529741 2.407665
H 5.881121 -5.563119 1.508859
C 0.902975 7.161611 1.859845
H 0.105420 7.476629 1.179160
H 1.843468 7.584656 1.489542
H 0.700381 7.603282 2.842178
C 1.429550 -2.956203 -2.779390
C 0.924855 -4.186724 -3.547485
H 1.704637 -4.581284 -4.210816
H 0.062290 -3.913318 -4.164476
H 0.601478 -4.989370 -2.879780
C 1.589998 -1.789002 -3.754735
H 2.093381 5.581211 3.847039
H 3.127535 5.659009 2.418871
H 2.316868 4.138221 2.843582
H 2.227503 -2.049987 -4.609049
H 2.017346 -0.901689 -3.272698
H 0.607974 -1.521285 -4.157486
H -2.974179 0.424443 -4.024868
C -0.717950 0.129675 -1.586995
H 0.031286 0.048453 -2.386384

18_TS8.log

SCF (wB97x) = -3807.12086699
E(SCF)+ZPE(0 K)= -3805.549006
H(298 K)= -3805.455105
G(298 K)= -3805.675354
Lowest Frequency = -20.8090cm-1

W -3.011242 11.279420 11.033598
Al -0.374962 13.449770 12.883460
Al -0.073690 7.886463 14.167863
O -0.888215 8.990670 13.074637
O -1.559037 12.384558 12.067056
O 0.664832 8.959644 15.311174
O 0.557245 12.289733 13.778765

C	-3.874595	12.421171	9.635983
N	0.626011	14.411340	11.547925
N	-1.151640	6.609185	15.084323
C	-4.824965	11.621434	11.779668
N	-1.189197	14.952989	13.675701
N	0.992715	6.594931	13.220591
C	-3.765804	9.450689	10.832075
O	-4.287203	13.099254	8.792300
O	-5.873918	11.781557	12.253885
O	-4.155215	8.367244	10.719365
C	0.785663	12.322584	16.815843
H	1.633106	12.767286	16.300263
C	-1.874404	7.040518	16.258725
C	-0.534434	10.312276	13.031639
C	0.585102	13.366373	8.725168
H	-0.190442	13.969485	9.209259
C	0.306480	11.079623	16.393047
C	-1.536052	15.088178	15.074144
C	2.262553	6.985307	12.648044
C	0.950492	16.352032	10.042470
H	1.925517	15.918321	9.821126
H	1.080731	17.392019	10.352072
H	0.351046	16.354965	9.126490
C	-3.214312	7.449191	16.143691
C	2.433126	10.489428	15.074735
C	-2.720395	14.524394	15.571846
C	1.732856	13.162104	9.713341
C	0.240456	10.955941	13.932954
C	-3.054597	14.759133	16.907044
H	-3.963904	14.321483	17.311040
C	2.834356	12.404671	9.302514
H	2.810752	11.924280	8.326836
C	0.642105	5.311519	13.095031
C	1.789526	13.759246	10.988553
C	-2.578368	16.966480	13.349456
H	-3.509613	16.424613	13.544807
H	-2.763541	17.727087	12.590088
H	-2.296285	17.456521	14.284327
C	0.216884	15.599251	11.123578
C	4.625825	10.535651	16.115156
H	5.230490	10.597224	17.016808
C	3.237078	10.557114	16.215462
H	2.773422	10.623899	17.195878
C	-0.694614	15.866592	15.893892
H	0.221855	8.158781	11.325081
C	-0.471377	4.741006	13.726160
H	-0.663461	3.697514	13.509912
C	1.511511	4.355184	12.311401
H	1.980294	4.823790	11.446072
H	0.922266	3.496150	11.985336
H	2.314366	3.986233	12.959782
C	3.436831	6.006864	14.714389
H	2.454459	5.545590	14.865754
C	-1.203629	7.049320	17.497555
C	0.900862	10.346996	15.179036
C	3.057870	10.377297	13.830959
H	2.452664	10.321359	12.928068
C	-0.083099	12.063802	8.273253
H	0.634101	11.394131	7.785262
H	-0.876467	12.285527	7.551003
H	-0.538042	11.521636	9.103875
C	2.940833	13.668901	11.799956

C	0.201219	12.982320	17.891492
H	0.594174	13.944419	18.210430
C	2.285767	7.620308	11.391142
C	-1.518699	15.997599	12.884212
C	3.444583	6.687336	13.350385
C	-1.386771	11.196694	18.125624
H	-2.238911	10.740749	18.624858
C	4.445519	10.339608	13.728095
H	4.902210	10.246093	12.746156
C	3.531053	7.954547	10.853739
H	3.580079	8.446383	9.886618
C	-3.962226	7.446701	14.817310
H	-3.350862	6.922238	14.075502
C	0.610205	16.476241	15.392349
H	0.801548	16.090321	14.385711
C	4.009635	12.903782	11.338185
H	4.897541	12.800648	11.953713
C	4.073080	13.913494	14.093877
H	3.872897	12.869000	14.342167
H	4.028198	14.499407	15.017832
H	5.098496	13.993269	13.714295
C	0.248620	6.611682	17.649225
H	0.692335	6.550893	16.650550
C	-1.255492	5.325744	14.724103
C	-3.637563	13.677908	14.706346
H	-3.330077	13.800561	13.660687
C	1.082625	14.116906	7.477616
H	1.636219	15.026877	7.723147
H	0.237651	14.389540	6.836246
H	1.749954	13.478333	6.888275
C	-0.911496	16.256993	11.660696
H	-1.236003	17.147331	11.134728
C	-1.089946	16.102564	17.210297
H	-0.465705	16.720772	17.852163
C	-0.794737	10.535072	17.052749
H	-1.187852	9.578853	16.727485
C	-3.871374	7.883048	17.297239
H	-4.904312	8.215495	17.227192
C	4.708828	7.666941	11.530741
H	5.667416	7.932613	11.091387
C	3.363106	15.932955	12.783936
H	4.313023	16.013013	12.242958
H	3.452652	16.514398	13.708406
H	2.586916	16.401092	12.170880
C	0.339272	5.216887	18.282020
H	-0.108394	5.209951	19.282729
H	1.386572	4.909711	18.379906
H	-0.175698	4.460916	17.679032
C	1.076259	7.626463	18.443581
H	1.023193	8.613213	17.975937
H	2.125605	7.313808	18.468779
H	0.734081	7.710237	19.481219
C	-2.247624	4.430923	15.424600
H	-2.134620	4.488205	16.510104
H	-2.125054	3.395574	15.105147
H	-3.268845	4.749666	15.194424
C	-1.913265	7.465997	18.624255
H	-1.418146	7.475102	19.591877
C	-2.260466	15.555527	17.717694
H	-2.549795	15.745375	18.748202
C	3.050775	14.462124	13.098896
H	2.079391	14.422530	13.604539

C	4.661660	7.044485	12.769209
H	5.587790	6.834660	13.297950
C	-5.105290	14.110432	14.805433
H	-5.517560	13.911890	15.800903
H	-5.708195	13.552317	14.082558
H	-5.228570	15.180434	14.602912
C	5.237475	10.424656	14.869497
H	6.321719	10.404869	14.789867
C	-0.887902	12.421641	18.553044
H	-1.345241	12.942185	19.390972
C	3.951214	12.255929	10.109758
H	4.789402	11.650456	9.774154
C	-3.483883	12.196466	15.061416
H	-2.458954	11.840169	14.904752
H	-4.156610	11.592521	14.446083
H	-3.731663	12.016988	16.114752
C	1.793217	16.064226	16.275861
C	3.630435	7.037309	15.832414
H	4.575259	7.578089	15.704990
H	3.651492	6.537616	16.808918
H	2.822899	7.776233	15.836490
C	-4.158430	8.877584	14.311377
H	-4.714983	9.472476	15.044779
H	-4.725317	8.876245	13.374115
H	-3.192948	9.359743	14.126385
C	-5.312315	6.724683	14.912190
H	-5.216837	5.717386	15.332599
H	-5.760226	6.638753	13.917098
H	-6.018453	7.277374	15.541610
C	-3.233671	7.885454	18.529476
H	-3.765929	8.214553	19.418491
C	4.481924	4.889697	14.818850
H	4.408427	4.178030	13.988799
H	4.346467	4.337853	15.754815
H	5.501894	5.288905	14.824446
C	0.526628	18.004631	15.289244
H	-0.257220	18.323413	14.594580
H	1.476913	18.414906	14.929709
H	0.314461	18.455347	16.265479
C	1.006410	7.896274	10.608328
C	0.527201	6.661264	9.831155
H	1.315681	6.285414	9.167204
H	-0.338267	6.923809	9.213477
H	0.216098	5.847940	10.491825
C	1.138880	9.074201	9.642647
H	1.677517	16.431412	17.301745
H	2.727707	16.481935	15.884474
H	1.895733	14.975241	16.316013
H	1.763247	8.828179	8.773950
H	1.568290	9.958708	10.127201
H	0.146550	9.338753	9.264659
H	-2.940279	10.584052	9.459174
C	-1.002630	11.035063	11.805325
H	-0.186660	11.139305	11.078591

19_Int8.log

SCF (wB97x) = -3807.12288674

E(SCF)+ZPE(0 K)= -3805.551694

H(298 K)= -3805.456588

G(298 K)= -3805.680960

Lowest Frequency = 12.1798cm-1

W	-2.793958	0.670034	-2.201292
Al	0.063523	2.597219	-0.565531
Al	0.329763	-2.995825	0.735128
O	-0.521434	-1.896000	-0.334165
O	-1.046830	1.538378	-1.480232
O	1.060870	-1.915916	1.875375
O	0.916957	1.416837	0.361558
C	-3.171444	0.268220	-4.085071
N	1.069007	3.605202	-1.848557
N	-0.714173	-4.295051	1.663935
C	-3.778531	2.394362	-2.653838
N	-0.813758	4.061965	0.228726
N	1.406989	-4.268309	-0.225900
C	-4.591334	0.221515	-1.496272
O	-3.314802	0.023379	-5.211145
O	-4.321215	3.374024	-2.940978
O	-5.611486	-0.045496	-1.011419
C	1.205832	1.438562	3.384798
H	2.052183	1.880097	2.864823
C	-1.450203	-3.861670	2.829356
C	-0.228065	-0.560751	-0.367058
C	0.992938	2.568440	-4.659108
H	0.234833	3.187598	-4.168916
C	0.710802	0.203949	2.956372
C	-1.174418	4.172443	1.626391
C	2.675276	-3.867992	-0.795833
C	1.377608	5.556658	-3.340411
H	2.351541	5.124790	-3.570561
H	1.510262	6.590665	-3.011630
H	0.777420	5.578475	-4.255647
C	-2.800269	-3.491245	2.698467
C	2.811310	-0.375951	1.593383
C	-2.344196	3.571446	2.115262
C	2.158753	2.366513	-3.692576
C	0.585263	0.082222	0.502083
C	-2.694870	3.790559	3.448603
H	-3.594684	3.325159	3.843303
C	3.257280	1.612781	-4.117233
H	3.225195	1.135756	-5.094401
C	1.075336	-5.558116	-0.348955
C	2.228767	2.957728	-2.416066
C	-2.238744	6.051040	-0.093599
H	-3.163241	5.483123	0.055086
H	-2.417534	6.826792	-0.838963
H	-1.997805	6.521811	0.862469
C	0.642587	4.786576	-2.272634
C	5.024804	-0.347638	2.590425
H	5.647434	-0.301969	3.480769
C	3.638411	-0.327468	2.718710
H	3.194526	-0.278597	3.709355
C	-0.356329	4.963052	2.458159
H	0.640658	-2.667162	-2.108194
C	-0.014608	-6.150876	0.300943
H	-0.187274	-7.198944	0.090241
C	1.946499	-6.497777	-1.151150
H	2.392363	-6.018625	-2.022787
H	1.364528	-7.364362	-1.470329
H	2.766548	-6.857230	-0.519342
C	3.848585	-4.858264	1.266090
H	2.870301	-5.330017	1.410585
C	-0.785140	-3.807305	4.069533

C	1.282686	-0.527795	1.729663
C	3.410977	-0.463673	0.335713
H	2.786695	-0.501275	-0.555070
C	0.298955	1.264065	-5.061046
H	0.994115	0.574383	-5.552863
H	-0.516952	1.472114	-5.761656
H	-0.129124	0.753532	-4.196686
C	3.382123	2.858672	-1.610099
C	0.638580	2.094970	4.472046
H	1.044170	3.050677	4.794893
C	2.699806	-3.225008	-2.048522
C	-1.144310	5.117312	-0.549271
C	3.857348	-4.171939	-0.095327
C	-0.962618	0.321766	4.708433
H	-1.813445	-0.130591	5.213201
C	4.796281	-0.501572	0.204490
H	5.233061	-0.577250	-0.787820
C	3.946013	-2.894383	-2.586096
H	3.997149	-2.398366	-3.550788
C	-3.546998	-3.582704	1.374658
H	-2.926844	-4.146518	0.670248
C	0.939265	5.603999	1.970994
H	1.141359	5.238818	0.958576
C	4.449753	2.101407	-2.086911
H	5.342201	1.994099	-1.478571
C	4.503562	3.065503	0.692395
H	4.297761	2.018118	0.923789
H	4.456056	3.637977	1.624815
H	5.531284	3.146227	0.319359
C	0.670377	-4.226750	4.240702
H	1.118747	-4.313445	3.245936
C	-0.792764	-5.581742	1.313416
C	-3.221345	2.692231	1.242782
H	-2.892185	2.815762	0.204792
C	1.467041	3.294647	-5.928756
H	2.021389	4.210852	-5.706858
H	0.610274	3.554302	-6.559669
H	2.125892	2.648612	-6.519414
C	-0.509040	5.415197	-1.749727
H	-0.841150	6.306126	-2.269910
C	-0.765339	5.178904	3.773831
H	-0.159116	5.805434	4.424859
C	-0.389135	-0.335139	3.623015
H	-0.796800	-1.283634	3.292280
C	-3.468393	-3.029555	3.834322
H	-4.508079	-2.722270	3.751421
C	5.123338	-3.190217	-1.912174
H	6.082379	-2.926822	-2.351830
C	3.805216	5.107204	-0.592382
H	4.759659	5.193265	-1.124360
H	3.887209	5.676719	0.340083
H	3.034366	5.582801	-1.206613
C	0.767794	-5.601461	4.915622
H	0.317131	-5.580483	5.914773
H	1.816717	-5.898585	5.026109
H	0.259566	-6.379283	4.335254
C	1.488163	-3.185122	5.009845
H	1.434459	-2.213195	4.512826
H	2.538503	-3.493172	5.051517
H	1.138368	-3.072079	6.042207
C	-1.748646	-6.498252	2.036134
H	-1.640305	-6.408401	3.119681

H	-1.583080	-7.535491	1.743121
H	-2.782068	-6.228835	1.796859
C	-1.507028	-3.368433	5.180291
H	-1.016574	-3.322136	6.149273
C	-1.926841	4.602231	4.269219
H	-2.227600	4.778597	5.298804
C	3.489008	3.632805	-0.299683
H	2.514186	3.590220	0.200518
C	5.075113	-3.816129	-0.675644
H	6.001001	-4.031316	-0.148807
C	-4.702004	3.083799	1.292567
H	-5.137024	2.896461	2.280506
H	-5.268581	2.489156	0.567676
H	-4.852473	4.143112	1.056638
C	5.611158	-0.438895	1.331028
H	6.693546	-0.459570	1.229171
C	-0.447687	1.538356	5.141212
H	-0.890790	2.055178	5.989046
C	4.382417	1.464182	-3.320746
H	5.219713	0.864193	-3.668258
C	-3.036457	1.219436	1.621725
H	-1.999288	0.888518	1.493467
H	-3.680668	0.584704	1.005380
H	-3.301210	1.048293	2.672093
C	2.126740	5.196167	2.850567
C	4.025340	-3.829864	2.388757
H	4.967810	-3.282957	2.270589
H	4.040585	-4.332023	3.364194
H	3.213603	-3.095488	2.387473
C	-3.765644	-2.196980	0.765097
H	-4.363901	-1.568834	1.435986
H	-4.302994	-2.282178	-0.185021
H	-2.809926	-1.701919	0.565837
C	-4.884097	-4.321292	1.517514
H	-4.770374	-5.296949	2.003364
H	-5.331525	-4.479761	0.531092
H	-5.600692	-3.741100	2.108937
C	-2.832968	-2.971880	5.066861
H	-3.373399	-2.621670	5.942835
C	4.903849	-5.965448	1.374009
H	4.844598	-6.674088	0.540211
H	4.765701	-6.522939	2.306189
H	5.919790	-5.556422	1.390645
C	0.828715	7.132067	1.893289
H	0.038648	7.447844	1.204217
H	1.771414	7.565084	1.540635
H	0.608821	7.562853	2.876750
C	1.420289	-2.938599	-2.827930
C	0.926358	-4.172232	-3.598260
H	1.708946	-4.557755	-4.263575
H	0.061130	-3.904850	-4.214021
H	0.611307	-4.979641	-2.932310
C	1.562567	-1.767035	-3.800539
H	2.000251	5.539359	3.883497
H	3.054499	5.639432	2.471385
H	2.249096	4.108568	2.868260
H	2.202677	-2.017045	-4.656205
H	1.977441	-0.874849	-3.316529
H	0.576678	-1.512500	-4.202043
H	-3.256823	-0.984791	-2.433576
C	-0.780999	0.101700	-1.595515
H	-0.092971	-0.062446	-2.433012

20_TS0.log

SCF (wB97x) = -3807.10643037

E(SCF)+ZPE(0 K)= -3805.535542

H(298 K)= -3805.441188

G(298 K)= -3805.662782

Lowest Frequency = -784.5541cm-1

W	-3.342363	11.527312	11.376098
Al	-0.325078	13.473484	12.812351
Al	-0.106876	7.877525	14.165623
O	-0.952506	8.993456	13.104683
O	-1.372440	12.386472	11.864163
O	0.661971	8.957955	15.276232
O	0.487122	12.305706	13.805639
C	-3.264622	11.166452	9.484196
N	0.742923	14.493261	11.597302
N	-1.135789	6.605384	15.140902
C	-4.548247	13.109839	10.963481
N	-1.217221	14.941092	13.589274
N	0.921187	6.594560	13.174649
C	-4.952731	10.380578	11.453956
O	-3.184906	10.939593	8.338245
O	-5.228617	14.009443	10.696603
O	-5.879991	9.680880	11.523148
C	0.808759	12.272459	16.845109
H	1.686816	12.701581	16.369483
C	-1.791756	7.024712	16.359990
C	-0.654793	10.325459	13.073928
C	0.730071	13.470359	8.796648
H	-0.011968	14.128695	9.257857
C	0.295043	11.065495	16.366514
C	-1.633045	15.023238	14.973203
C	2.184744	6.977771	12.582025
C	1.134363	16.447021	10.127864
H	2.114032	16.008679	9.937575
H	1.258786	17.482829	10.453791
H	0.570946	16.463662	9.189227
C	-3.147235	7.395267	16.331588
C	2.410063	10.490948	15.005221
C	-2.847206	14.450258	15.384934
C	1.877528	13.262046	9.781625
C	0.176482	10.972604	13.922571
C	-3.243249	14.613206	16.712112
H	-4.177071	14.170839	17.048577
C	2.982467	12.505378	9.379723
H	2.971793	12.033481	8.399675
C	0.559097	5.312966	13.058739
C	1.916439	13.840835	11.063971
C	-2.588571	16.966680	13.256346
H	-3.535267	16.420047	13.328698
H	-2.705509	17.775908	12.534707
H	-2.391330	17.392877	14.242642
C	0.352256	15.686231	11.168851
C	4.622540	10.474478	16.003934
H	5.245330	10.502967	16.894773
C	3.236835	10.516913	16.131879
H	2.793496	10.561582	17.122852
C	-0.828607	15.755435	15.867049
H	0.134156	8.167857	11.294694

C	-0.529775	4.745819	13.734392
H	-0.735613	3.704290	13.521111
C	1.392475	4.353698	12.241124
H	1.859635	4.827882	11.378209
H	0.777011	3.515940	11.907800
H	2.194394	3.951211	12.870305
C	3.388253	5.978481	14.624199
H	2.393049	5.560503	14.813446
C	-1.044218	7.057704	17.553474
C	0.882821	10.344615	15.139982
C	3.008532	10.400124	13.747391
H	2.386370	10.373673	12.854588
C	-0.001356	12.172752	8.451812
H	0.673711	11.441831	7.992455
H	-0.819680	12.365152	7.751023
H	-0.439630	11.724987	9.345071
C	3.047432	13.733814	11.899511
C	0.217814	12.918971	17.926223
H	0.638920	13.853271	18.289462
C	2.195852	7.622364	11.329636
C	-1.493510	16.022330	12.824209
C	3.375131	6.666093	13.264139
C	-1.446332	11.192904	18.050900
H	-2.329465	10.754824	18.510208
C	4.393166	10.337503	13.616855
H	4.828789	10.257370	12.624437
C	3.436081	7.950196	10.776990
H	3.476506	8.448443	9.813091
C	-3.966704	7.402845	15.049579
H	-3.405860	6.866053	14.277187
C	0.487774	16.403435	15.449007
H	0.728726	16.063056	14.436406
C	4.126638	12.980976	11.441557
H	5.004428	12.868318	12.069843
C	4.122299	13.917734	14.224056
H	3.936017	12.860328	14.424099
H	4.041728	14.464316	15.169717
H	5.155658	14.030899	13.875710
C	0.430830	6.680282	17.608337
H	0.824383	6.702866	16.588317
C	-1.256789	5.323041	14.779779
C	-3.709565	13.673133	14.407841
H	-3.426960	13.991592	13.398468
C	1.232268	14.142456	7.509751
H	1.800657	15.056015	7.710074
H	0.386376	14.397242	6.862720
H	1.884401	13.467217	6.944997
C	-0.803982	16.332022	11.658318
H	-1.098929	17.237105	11.140376
C	-1.282397	15.919101	17.176594
H	-0.686466	16.498552	17.878609
C	-0.847311	10.543648	16.975194
H	-1.268438	9.613542	16.610098
C	-3.745628	7.800575	17.526769
H	-4.790830	8.099880	17.522803
C	4.621432	7.648856	11.434399
H	5.575327	7.910424	10.982729
C	3.420609	15.972512	12.966699
H	4.380772	16.080828	12.449284
H	3.484757	16.521848	13.912662
H	2.652375	16.454225	12.354338
C	0.619370	5.256184	18.146057

H	0.216938	5.162990	19.161595
H	1.684271	4.998690	18.180482
H	0.117180	4.513867	17.515596
C	1.250202	7.688201	18.419510
H	1.090196	8.702311	18.042149
H	2.316899	7.455265	18.337662
H	0.991730	7.664975	19.484146
C	-2.199842	4.419253	15.534782
H	-2.009816	4.463290	16.610348
H	-2.099015	3.388078	15.195008
H	-3.235129	4.738984	15.382113
C	-1.695013	7.447006	18.724518
H	-1.139938	7.473749	19.658269
C	-2.475888	15.352933	17.600826
H	-2.808812	15.487618	18.626758
C	3.117705	14.488734	13.223653
H	2.133642	14.421668	13.703060
C	4.586796	7.018118	12.668554
H	5.518260	6.797032	13.183460
C	-5.208581	13.942480	14.557498
H	-5.602248	13.544976	15.499447
H	-5.754535	13.455839	13.742387
H	-5.430996	15.014324	14.523691
C	5.207929	10.380429	14.744150
H	6.289779	10.340347	14.642964
C	-0.911633	12.381488	18.535526
H	-1.373745	12.891126	19.377440
C	4.088072	12.351683	10.202257
H	4.933382	11.753480	9.871240
C	-3.416838	12.174324	14.520179
H	-2.350731	11.939491	14.437273
H	-3.983027	11.606716	13.758194
H	-3.744328	11.782048	15.490121
C	1.644027	15.979112	16.360876
C	3.673536	6.995057	15.734860
H	4.639764	7.487036	15.574770
H	3.703527	6.493430	16.710115
H	2.905890	7.774686	15.765999
C	-4.153764	8.838418	14.553132
H	-4.661186	9.445641	15.312968
H	-4.760879	8.853377	13.643082
H	-3.187038	9.298025	14.323719
C	-5.323947	6.708003	15.211337
H	-5.226142	5.697228	15.623325
H	-5.823383	6.635629	14.240222
H	-5.987908	7.271548	15.875823
C	-3.033018	7.817631	18.716798
H	-3.518925	8.123774	19.639849
C	4.387729	4.816832	14.683964
H	4.248871	4.110175	13.858240
H	4.269222	4.269587	15.624987
H	5.422985	5.172349	14.644515
C	0.378185	17.933336	15.404900
H	-0.383333	18.267523	14.693069
H	1.334002	18.374834	15.101652
H	0.118548	18.338334	16.389683
C	0.909076	7.912905	10.564192
C	0.417703	6.688152	9.778296
H	1.198844	6.317727	9.103026
H	-0.452700	6.959173	9.171685
H	0.112376	5.868600	10.433940
C	1.039215	9.100712	9.609927

H	1.481298	16.298812	17.396335
H	2.581817	16.435064	16.023901
H	1.770802	14.892503	16.357029
H	1.679519	8.869504	8.749466
H	1.449536	9.987914	10.107347
H	0.052521	9.355541	9.209767
H	-2.643658	9.936789	11.754907
C	-1.256783	10.974517	11.857461
H	-0.749880	10.627416	10.952623

21_Int9.log

SCF (wB97x) = -3807.15629040

E(SCF)+ZPE(0 K)= -3805.578986

H(298 K)= -3805.484189

G(298 K)= -3805.709313

Lowest Frequency = 12.2468cm-1

W	-3.164303	1.603593	-1.902970
Al	-0.020808	2.429042	-0.436788
Al	0.682229	-3.119787	0.724919
O	-0.028627	-2.056021	-0.473638
O	-1.190192	1.238607	-1.152087
O	1.377677	-2.009276	1.857917
O	1.052151	1.335808	0.384705
C	-3.653377	1.419956	-3.758577
N	0.720376	3.391712	-1.912279
N	-0.599866	-4.298896	1.489404
C	-3.819208	3.401156	-2.037852
N	-0.867953	3.854978	0.428977
N	1.778313	-4.471504	-0.051460
C	-4.992541	1.112240	-1.469482
O	-3.891296	1.300539	-4.899882
O	-4.183822	4.516710	-2.090001
O	-6.065740	0.781635	-1.139550
C	1.751568	1.276403	3.424651
H	2.626261	1.668619	2.912576
C	-1.556124	-3.707020	2.395790
C	0.143146	-0.707800	-0.390145
C	0.292564	2.199844	-4.658710
H	-0.459680	2.723026	-4.056280
C	1.144505	0.112479	2.950956
C	-1.056607	3.958500	1.861540
C	3.104768	-4.116966	-0.506194
C	0.702104	5.298799	-3.494084
H	1.657533	4.914753	-3.851197
H	0.808448	6.357967	-3.247786
H	-0.033894	5.222596	-4.300755
C	-2.703451	-3.086233	1.863513
C	3.159039	-0.495255	1.489046
C	-2.166503	3.366233	2.484282
C	1.600766	2.134217	-3.867724
C	0.881806	-0.023946	0.504976
C	-2.348316	3.578415	3.852255
H	-3.202923	3.125247	4.348889
C	2.681452	1.454067	-4.438485
H	2.540040	0.942257	-5.387653
C	1.396153	-5.747590	-0.178150
C	1.817486	2.784922	-2.634317
C	-2.341698	5.829380	0.280441
H	-3.253076	5.262326	0.495090
H	-2.593106	6.619893	-0.426124

H	-2.000500	6.271236	1.219609
C	0.192375	4.553848	-2.287516
C	5.409003	-0.614589	2.389933
H	6.067649	-0.698528	3.251168
C	4.030815	-0.612122	2.575613
H	3.623343	-0.705035	3.579172
C	-0.136897	4.737566	2.591829
H	1.180578	-3.420134	-2.178098
C	0.215407	-6.264666	0.366577
H	0.023438	-7.315944	0.190822
C	2.298393	-6.730882	-0.882529
H	2.740976	-6.301650	-1.783100
H	1.746614	-7.635252	-1.142495
H	3.126592	-7.011976	-0.222702
C	4.037420	-5.063511	1.695073
H	3.046027	-5.528141	1.729461
C	-1.297561	-3.726074	3.778692
C	1.638809	-0.626418	1.695660
C	3.706436	-0.397706	0.210471
H	3.049926	-0.292217	-0.649229
C	-0.293743	0.827740	-5.021399
H	0.397485	0.253617	-5.649297
H	-1.224354	0.963901	-5.581609
H	-0.532452	0.223555	-4.144769
C	3.087494	2.800099	-2.015495
C	1.260242	1.925748	4.553129
H	1.749642	2.829442	4.908770
C	3.275712	-3.527354	-1.771100
C	-1.312188	4.900447	-0.308558
C	4.201373	-4.389613	0.336211
C	-0.482408	0.283729	4.739431
H	-1.363215	-0.115888	5.238159
C	5.086118	-0.415285	0.018484
H	5.483594	-0.345038	-0.991485
C	4.574926	-3.213704	-2.178095
H	4.731142	-2.770394	-3.159029
C	-3.051892	-3.144179	0.379703
H	-2.180960	-3.525918	-0.159295
C	1.078358	5.395952	1.947274
H	1.135477	5.064435	0.905330
C	4.125536	2.101903	-2.630308
H	5.107384	2.086607	-2.167575
C	4.584295	3.200159	0.032372
H	4.570562	2.136422	0.281404
H	4.629368	3.766473	0.968165
H	5.505694	3.419560	-0.520116
C	-0.086926	-4.431036	4.380616
H	0.420129	-4.980891	3.579348
C	-0.684323	-5.599759	1.209905
C	-3.173865	2.521208	1.723299
H	-2.962114	2.634336	0.651693
C	0.501780	2.978658	-5.970057
H	0.996144	3.941174	-5.820059
H	-0.460925	3.156483	-6.460331
H	1.122950	2.398312	-6.661642
C	-0.858315	5.183203	-1.591802
H	-1.294252	6.047970	-2.077059
C	-0.374790	4.939532	3.950842
H	0.313035	5.555313	4.526680
C	0.010743	-0.361550	3.609811
H	-0.484817	-1.252617	3.239423
C	-3.572225	-2.447542	2.750369

H	-4.464619	-1.961260	2.363058
C	5.664544	-3.456769	-1.356464
H	6.666810	-3.197082	-1.687814
C	3.428083	5.115076	-1.089353
H	4.246971	5.298304	-1.794512
H	3.627504	5.699503	-0.183863
H	2.506709	5.500301	-1.534555
C	-0.507414	-5.449333	5.448861
H	-0.934270	-4.952117	6.326759
H	0.361483	-6.022867	5.788156
H	-1.257670	-6.154071	5.073638
C	0.925506	-3.433044	4.956333
H	1.299505	-2.762041	4.178153
H	1.774793	-3.969790	5.395074
H	0.470580	-2.820312	5.743613
C	-1.749316	-6.440358	1.866637
H	-1.431864	-6.687181	2.886263
H	-1.893564	-7.373717	1.320564
H	-2.700241	-5.911929	1.945999
C	-2.196932	-3.070141	4.621503
H	-2.010817	-3.060410	5.693182
C	-1.473402	4.370259	4.579854
H	-1.643957	4.540168	5.639937
C	3.339878	3.619098	-0.751257
H	2.488078	3.470995	-0.076775
C	5.473924	-4.034348	-0.108087
H	6.333221	-4.218978	0.531303
C	-4.619834	2.986338	1.929067
H	-4.956491	2.808710	2.956726
H	-5.286744	2.434774	1.257914
H	-4.737652	4.054415	1.718417
C	5.944060	-0.518878	1.107377
H	7.021209	-0.525646	0.960151
C	0.146253	1.426895	5.221130
H	-0.235053	1.934804	6.103345
C	3.923618	1.420980	-3.825034
H	4.744180	0.877009	-4.286154
C	-3.025456	1.041136	2.087613
H	-2.025282	0.658065	1.852652
H	-3.763037	0.444133	1.541654
H	-3.186495	0.883856	3.160981
C	2.379340	4.967713	2.634684
C	4.091656	-4.037357	2.831709
H	5.056737	-3.517674	2.838128
H	3.962340	-4.534445	3.800680
H	3.308765	-3.281497	2.717748
C	-3.364651	-1.762726	-0.202473
H	-4.310413	-1.359774	0.177899
H	-3.464795	-1.830905	-1.293017
H	-2.566155	-1.053686	0.036366
C	-4.231111	-4.094303	0.127865
H	-4.014112	-5.118303	0.448271
H	-4.473376	-4.123015	-0.939718
H	-5.125491	-3.757405	0.664040
C	-3.318795	-2.426688	4.115692
H	-4.005590	-1.918953	4.788141
C	5.066255	-6.177954	1.920463
H	5.084235	-6.895113	1.092044
H	4.830803	-6.724304	2.839613
H	6.079060	-5.776659	2.033532
C	0.954004	6.924880	1.932092
H	0.065350	7.254206	1.384027

H	1.829725	7.373749	1.450192
H	0.888627	7.326459	2.949675
C	2.115206	-3.272029	-2.725332
C	2.142141	-4.252193	-3.905740
H	3.070508	-4.150911	-4.479870
H	1.304490	-4.056216	-4.583639
H	2.065584	-5.293415	-3.576194
C	2.105417	-1.829004	-3.238542
H	2.398556	5.273774	3.686847
H	3.241560	5.433192	2.143237
H	2.504913	3.881211	2.595566
H	2.977325	-1.609365	-3.865452
H	2.103352	-1.111911	-2.411875
H	1.212001	-1.649956	-3.848219
H	-1.255410	-0.679770	-1.975622
C	-0.523802	0.006101	-1.535694
H	0.214666	0.258375	-2.307203

22_Int1a.log

SCF (wB97x) = -3920.43645857

E(SCF)+ZPE(0 K)= -3918.858565

H(298 K)= -3918.760955

G(298 K)= -3918.987935

Lowest Frequency = 16.6896cm-1

W	-2.756003	-0.298725	-2.663006
Al	0.148213	2.940809	-0.564507
Al	0.214914	-2.655053	0.762999
O	-0.603490	-1.493716	-0.253808
O	-0.933619	1.855590	-1.449003
O	1.112212	-1.605695	1.814165
O	0.944865	1.741513	0.419113
C	-4.312939	-0.872646	-3.819323
N	1.197605	3.946036	-1.800238
N	-0.854683	-3.889191	1.760433
C	-2.694475	-2.124488	-1.864435
N	-0.643139	4.413167	0.297217
N	1.255449	-3.968672	-0.159379
C	-1.594976	-0.970698	-4.206536
O	-5.216756	-1.206600	-4.466744
O	-2.674824	-3.212245	-1.457217
O	-5.062982	0.765762	-0.711193
C	-1.135728	0.549629	-1.341121
O	-1.047063	-1.376417	-5.143036
C	1.465110	1.720254	3.330725
H	2.284976	2.147107	2.758443
C	-1.516346	-3.465433	2.977368
C	-0.330819	-0.157551	-0.330422
C	1.058486	2.920961	-4.582656
H	0.332277	3.565229	-4.077464
C	0.904221	0.507153	2.922191
C	-0.958290	4.502310	1.708011
C	2.600128	-3.690745	-0.617198
C	1.540632	5.912150	-3.268044
H	2.514352	5.478100	-3.494769
H	1.671319	6.949743	-2.951076
H	0.942758	5.921640	-4.185437
C	-2.878461	-3.116486	2.953876
C	2.887516	-0.130407	1.414425
C	-2.110952	3.896046	2.227288
C	2.273432	2.755217	-3.677356

C	0.602295	0.446232	0.488790	H	1.005894	0.891140	-5.380228
C	-2.416954	4.101763	3.574948	H	-0.546639	1.736907	-5.438010
H	-3.304830	3.633463	3.992498	H	0.063376	1.116352	-3.894995
C	3.378540	2.045575	-4.154254	C	3.522420	3.197792	-1.603025
H	3.333560	1.597363	-5.144087	C	0.995695	2.372582	4.465279
C	0.816739	-5.221494	-0.328716	H	1.448842	3.311812	4.772462
C	2.354688	3.304916	-2.385050	C	2.800127	-3.062470	-1.860051
C	-2.004955	6.456052	0.053524	C	-0.952099	5.497874	-0.445932
H	-2.937541	5.910582	0.230650	C	3.684833	-4.108356	0.175770
H	-2.186195	7.244695	-0.677827	C	-0.629750	0.635855	4.796588
H	-1.720722	6.910752	1.005214	H	-1.452028	0.195066	5.355699
C	0.800113	5.150105	-2.197790	C	4.773714	-0.291989	-0.099744
C	5.153084	-0.243265	2.272383	H	5.145745	-0.353729	-1.118908
H	5.830477	-0.263909	3.122628	C	4.111213	-2.923853	-2.319772
C	3.780576	-0.160539	2.488347	H	4.290942	-2.461877	-3.285929
H	3.397233	-0.132715	3.504882	C	-3.727502	-3.172699	1.693058
C	-0.107660	5.275948	2.525919	H	-3.167922	-3.693031	0.911245
H	0.826033	-2.261100	-2.043044	C	1.184560	5.907943	2.016608
C	-0.319502	-5.737805	0.304845	H	1.363803	5.549171	0.997610
H	-0.583993	-6.757671	0.054723	C	4.598210	2.485157	-2.129736
C	1.612306	-6.208600	-1.150669	H	5.504278	2.375005	-1.542318
H	2.138821	-5.738337	-1.980938	C	4.669127	3.309036	0.689051
H	0.951073	-6.988605	-1.533139	H	4.476015	2.247301	0.861122
H	2.363645	-6.687606	-0.513763	H	4.621800	3.827030	1.652735
C	3.513100	-4.732066	1.555113	H	5.694050	3.420072	0.316677
H	2.449441	-4.942569	1.714556	C	0.708311	-3.791254	4.217975
C	-0.767431	-3.411939	4.172510	H	1.148960	-3.508118	3.258841
C	1.370900	-0.237597	1.653795	C	-1.021923	-5.157816	1.366803
C	3.402637	-0.196009	0.119917	C	-4.186373	0.388152	-1.365607
H	2.725015	-0.180405	-0.731770	C	-3.028129	3.020664	1.391083
C	0.357261	1.586893	-4.836738	H	-2.689690	3.055193	0.348435

C	1.437397	3.584158	-5.914287	H	-0.859735	-2.952484	6.268630
H	1.978237	4.525013	-5.771327	C	-1.622359	4.900647	4.380749
H	0.537299	3.790651	-6.502917	H	-1.890347	5.065986	5.421105
H	2.074994	2.925830	-6.514032	C	3.645887	3.922208	-0.267279
C	-0.326748	5.800679	-1.651689	H	2.674720	3.870687	0.240098
H	-0.637668	6.712163	-2.148840	C	4.976949	-3.922002	-0.317919
C	-0.473453	5.476947	3.855912	H	5.826967	-4.232824	0.284867
H	0.157138	6.090624	4.495684	C	-4.481965	3.508078	1.415461
C	-0.152799	-0.018522	3.662917	H	-4.913041	3.429027	2.419680
H	-0.604195	-0.954209	3.354747	H	-5.090202	2.894829	0.743779
C	-3.473290	-2.690034	4.144166	H	-4.567861	4.554179	1.100228
H	-4.521857	-2.402880	4.138106	C	5.655693	-0.314422	0.975863
C	5.192401	-3.355885	-1.564139	H	6.726929	-0.388511	0.805865
H	6.204658	-3.236266	-1.942439	C	-0.054617	1.833384	5.203914
C	3.972821	5.402502	-0.515995	H	-0.421561	2.349049	6.087924
H	4.911660	5.494475	-1.073940	C	4.524726	1.899605	-3.388006
H	4.090896	5.939049	0.431481	H	5.370511	1.337585	-3.775789
H	3.189883	5.908078	-1.090337	C	-2.937314	1.567019	1.865643
C	0.891621	-5.303804	4.399769	H	-1.921574	1.170359	1.762619
H	0.416774	-5.645560	5.327239	H	-3.616169	0.939453	1.286242
H	1.957585	-5.555560	4.454866	H	-3.212255	1.480350	2.923676
H	0.461594	-5.871924	3.568020	C	2.382460	5.479179	2.872491
C	1.485725	-3.022380	5.289912	C	3.956064	-3.739669	2.636273
H	1.310042	-1.944926	5.206188	H	4.997971	-3.434918	2.483134
H	2.558253	-3.201589	5.168252	H	3.880999	-4.198366	3.629530
H	1.218518	-3.342254	6.303848	H	3.337870	-2.836982	2.614953
C	-1.986984	-6.057663	2.100005	C	-4.001680	-1.763308	1.169767
H	-1.883518	-5.955761	3.182741	H	-4.511368	-1.155176	1.926890
H	-1.822639	-7.098949	1.820873	H	-4.641540	-1.805971	0.282778
H	-3.016827	-5.791551	1.845159	H	-3.067962	-1.264217	0.890160
C	-1.416275	-3.002715	5.337891	C	-5.048895	-3.921748	1.911638

H -4.901206 -4.903847 2.374210

H -5.555118 -4.067980 0.952136

H -5.730392 -3.355765 2.556397

C -2.756266 -2.637198 5.328417

H -3.239391 -2.313198 6.246881

C 4.271192 -6.057508 1.701065

H 4.017334 -6.769436 0.908539

H 4.030944 -6.522898 2.663227

H 5.355786 -5.905646 1.673368

C 1.095544 7.438359 1.955725

H 0.303411 7.774800 1.279338

H 2.040669 7.860398 1.596366

H 0.894362 7.861869 2.946272

C 1.636608 -2.572953 -2.713224

C 1.082115 -3.686647 -3.613976

H 1.881560 -4.126677 -4.223216

H 0.324216 -3.281906 -4.291061

H 0.610963 -4.484903 -3.033603

C 2.014312 -1.358702 -3.564886

H 2.274912 5.810962 3.911048

H 3.308261 5.919070 2.485906

H 2.495255 4.390518 2.876054

H 2.705369 -1.624756 -4.374196

H 2.480734 -0.563211 -2.971693

H 1.118061 -0.954268 -4.035091

H -2.376482 1.587392 -3.160796

H -2.958955 1.396616 -3.667312

23_TS1a.log

SCF (wB97x) = -3920.37906419

E(SCF)+ZPE(0 K)= -3918.804960

H(298 K)= -3918.706203

G(298 K)= -3918.939043

Lowest Frequency = -51.1712cm-1

W -3.264062 10.098493
11.029522

Al -0.281055 13.545885
12.825699

Al -0.127727 7.957234 14.201496

O -1.008598 9.128379 13.264572

O -1.412599 12.438597 12.021696

O 0.740623 9.022711 15.268385

O 0.545526 12.369135 13.831377

C -4.946214 9.354180 10.241140

N 0.777053 14.529049 11.580072

N -1.239906 6.728313 15.123946

C -2.675689 8.249840 11.371542

N -1.068481 15.049136 13.649101

N 0.886271 6.657179 13.242911

C -2.488194 9.943048 9.273710

O -5.928908 8.920072 9.787934

O -2.350044 7.142118 11.549489

O -7.307339 10.971359 12.199023

C -1.583520 11.134255 12.172982

O -2.013622 9.804594 8.208646

C 1.036702 12.311102 16.840142

H 1.931692 12.698962 16.360828

C -1.969845 7.206363 16.272925

C -0.727513 10.455251 13.144672

C 0.628935 13.533685 8.796216

H -0.042567 14.252447 9.274030

C	0.449854	11.142766	16.350588	C	1.265235	4.437421	12.229226
C	-1.425566	15.138564	15.048873	H	1.681653	4.918311	11.343455
C	2.175042	6.993460	12.678556	H	0.637611	3.598029	11.925546
C	1.196741	16.477588	10.107745	H	2.105188	4.041349	12.811208
H	2.159441	16.013618	9.893973	C	3.288844	5.904613	14.721077
H	1.354619	17.512442	10.421397	H	2.284072	5.490367	14.860581
H	0.610501	16.499434	9.183103	C	-1.323469	7.222387	17.524437
C	-3.289736	7.669977	16.120661	C	0.981249	10.397916	15.109230
C	2.500709	10.513214	14.905499	C	3.041728	10.429515	13.622306
C	-2.634781	14.598995	15.511237	H	2.385643	10.439657	12.753887
C	1.823802	13.302344	9.715461	C	-0.191377	12.265100	8.570076
C	0.221887	11.074205	13.932601	H	0.401649	11.481592	8.087298
C	-2.970872	14.778426	16.854408	H	-1.049127	12.473365	7.922544
H	-3.899931	14.357099	17.230659	H	-0.576985	11.869813	9.513467
C	2.903293	12.543497	9.255460	C	3.082716	13.729677	11.792182
H	2.846484	12.084251	8.271435	C	0.496420	12.970407	17.939566
C	0.468606	5.396291	13.081741	H	0.974181	13.872740	18.313437
C	1.920343	13.855769	11.004648	C	2.234372	7.667373	11.446006
C	-2.366155	17.132862	13.388628	C	-1.334291	16.144475	12.903290
H	-3.327154	16.623656	13.516008	C	3.339008	6.597786	13.364389
H	-2.488316	17.944967	12.670942	C	-1.261809	11.338482	18.057116
H	-2.100934	17.553276	14.361243	H	-2.163924	10.943981	18.518720
C	0.422163	15.743082	11.173708	C	4.416497	10.320129	13.432715
C	4.747769	10.395163	15.811058	H	4.807333	10.241683	12.422160
H	5.407214	10.379167	16.675352	C	3.495105	7.904257	10.892178
C	3.371558	10.483829	15.997962	H	3.567814	8.411089	9.933934
H	2.969478	10.514609	17.006942	C	-4.017142	7.657108	14.782061
C	-0.563051	15.847940	15.909229	H	-3.391098	7.140223	14.047925
H	0.184902	8.265955	11.440402	C	0.758037	16.450517	15.440383
C	-0.642890	4.853250	13.738296	H	0.946118	16.106921	14.417788
H	-0.886450	3.825468	13.500460	C	4.134889	12.974251	11.277759

H	5.037820	12.845566	11.866157
C	4.265227	13.887445	14.067317
H	4.082085	12.830125	14.269546
H	4.233387	14.428576	15.018939
H	5.281699	13.995603	13.671287
C	0.098781	6.710302	17.720332
H	0.537889	6.536104	16.732347
C	-1.391806	5.451841	14.753072
C	-6.246774	11.290630	12.445365
C	-3.574366	13.832978	14.597319
H	-3.221361	13.951972	13.566392
C	1.072859	14.131961	7.453919
H	1.691505	15.026634	7.582190
H	0.196672	14.401349	6.854933
H	1.653763	13.408907	6.871510
C	-0.686349	16.432085	11.708119
H	-0.962295	17.353369	11.208674
C	-0.958186	16.028881	17.234224
H	-0.316952	16.591269	17.909618
C	-0.713508	10.674882	16.963383
H	-1.191148	9.775435	16.589463
C	-3.947390	8.164725	17.249033
H	-4.965557	8.534502	17.150577
C	4.653826	7.505048	11.543008
H	5.624991	7.695900	11.092436
C	3.530157	15.955666	12.857272
H	4.459720	16.054031	12.285022
H	3.659353	16.494262	13.802671
H	2.735340	16.456372	12.296536
C	0.096614	5.370020	18.467385
H	-0.336668	5.478681	19.468488
H	1.119822	4.995807	18.583077
H	-0.481404	4.607526	17.933936
C	0.987447	7.732876	18.435300
H	1.022737	8.669401	17.872420
H	2.008108	7.344408	18.521571
H	0.628939	7.945665	19.448771
C	-2.408311	4.587535	15.454791
H	-2.325942	4.677868	16.540773
H	-2.286495	3.542153	15.169805
H	-3.419142	4.905330	15.181747
C	-2.031749	7.708156	18.624293
H	-1.553237	7.723585	19.600492
C	-2.151127	15.500169	17.708232
H	-2.438878	15.647745	18.745996
C	3.220244	14.473606	13.117872
H	2.257850	14.415094	13.641385
C	4.573212	6.872233	12.775713
H	5.486566	6.581779	13.288228
C	-5.012654	14.360234	14.654698
H	-5.463449	14.194898	15.639358
H	-5.631724	13.836879	13.918260
H	-5.065074	15.433796	14.442522
C	5.276559	10.307624	14.525880
H	6.350661	10.226946	14.378673
C	-0.654040	12.485691	18.554363
H	-1.076224	13.005572	19.410749
C	4.039440	12.366805	10.030552
H	4.864704	11.766610	9.655733
C	-3.536800	12.339256	14.924509
H	-2.521589	11.932290	14.860376
H	-4.174280	11.790197	14.226158

H	-3.897280	12.148356	15.943035
C	1.931637	15.978420	16.305532
C	3.522631	6.918795	15.847271
H	4.500713	7.401617	15.735868
H	3.497341	6.417346	16.822960
H	2.761352	7.705794	15.838045
C	-4.223299	9.084527	14.268944
H	-4.840996	9.669396	14.961563
H	-4.737093	9.056428	13.297178
H	-3.258965	9.587784	14.148197
C	-5.365470	6.929165	14.859521
H	-5.272082	5.921897	15.278874
H	-5.800303	6.841674	13.858492
H	-6.080430	7.477348	15.483614
C	-3.330650	8.180942	18.492377
H	-3.863821	8.559393	19.361058
C	4.276591	4.737272	14.828064
H	4.172962	4.034069	13.994147
H	4.106611	4.188135	15.759984
H	5.315311	5.084690	14.843213
C	0.705897	17.983745	15.406956
H	-0.067906	18.351904	14.725878
H	1.666018	18.389328	15.069255
H	0.500155	18.393209	16.402508
C	0.975256	8.094939	10.704075
C	0.471067	7.005424	9.747875
H	1.254216	6.718642	9.034798
H	-0.388296	7.379708	9.181566
H	0.140553	6.110971	10.283518
C	1.153671	9.405947	9.940311
H	1.818111	16.295354	17.348316
H	2.872190	16.403253	15.937515
H	2.018386	14.888135	16.289953
H	1.837407	9.301648	9.088118
H	1.535073	10.211299	10.580027
H	0.186072	9.712962	9.539970
H	-3.565611	12.087101	11.007794
H	-4.084373	11.819992	10.468703

24_Int2a.log

SCF (wB97x) = -3807.09878597

E(SCF)+ZPE(0 K)= -3805.530035

H(298 K)= -3805.434833

G(298 K)= -3805.657867

Lowest Frequency = 14.8746cm-1

W	-3.116278	-0.394854	-2.086768
Al	0.097930	2.850528	-0.554623
Al	0.260421	-2.747520	0.687319
O	-0.615497	-1.581837	-0.274293
O	-1.128150	1.805620	-1.269895
O	1.180709	-1.714729	1.734832
O	0.939414	1.655454	0.401199
C	-4.743170	-1.268948	-2.902377
N	1.106202	3.763685	-1.880138
N	-0.907676	-3.962871	1.589887
C	-1.997257	-1.802639	-2.923855
N	-0.604672	4.376131	0.296922
N	1.290083	-4.094338	-0.186390
C	-3.005129	0.715860	-3.664214
O	-5.667514	-1.772597	-3.398919

O	-1.357694	-2.635773	-3.427310
C	-1.330653	0.510255	-1.127395
O	-2.965866	1.420771	-4.599982
C	1.493621	1.607306	3.290113
H	2.310129	2.047043	2.723426
C	-1.703077	-3.498234	2.702877
C	-0.368124	-0.232021	-0.295147
C	0.837387	2.449517	-4.538379
H	0.085468	3.058694	-4.025793
C	0.957150	0.384047	2.879541
C	-0.889143	4.500602	1.709820
C	2.662430	-3.826502	-0.559153
C	1.363221	5.615879	-3.500672
H	2.349586	5.203296	-3.713512
H	1.447920	6.689461	-3.318536
H	0.734835	5.477658	-4.386963
C	-3.027634	-3.082673	2.481038
C	2.933224	-0.194676	1.337722
C	-2.046658	3.929838	2.260911
C	2.113310	2.435265	-3.705473
C	0.614214	0.355353	0.471119
C	-2.316039	4.148388	3.613951
H	-3.207248	3.707552	4.054264
C	3.229002	1.752243	-4.199171
H	3.148953	1.228167	-5.148607
C	0.830081	-5.330667	-0.406676
C	2.245525	3.096203	-2.471850
C	-1.910247	6.453414	0.036032
H	-2.855585	5.955179	0.273757
H	-2.094022	7.224643	-0.712703
H	-1.562928	6.925997	0.957639
C	0.699906	4.948737	-2.323676
C	5.213703	-0.250330	2.163214
H	5.902796	-0.263806	3.004173
C	3.842189	-0.214660	2.398157
H	3.472983	-0.212979	3.420334
C	-0.003879	5.258917	2.503908
H	0.952785	-3.547146	-2.544040
C	-0.343862	-5.828984	0.168519
H	-0.626092	-6.837630	-0.104669
C	1.620673	-6.278646	-1.272350
H	1.726201	-5.870293	-2.281533
H	1.125838	-7.248264	-1.333566
H	2.631169	-6.417298	-0.878175
C	3.371269	-4.655242	1.773236
H	2.287848	-4.787923	1.864302
C	-1.124282	-3.467386	3.988638
C	1.424322	-0.337851	1.598150
C	3.431692	-0.215385	0.034674
H	2.747353	-0.195689	-0.810902
C	0.245752	1.046902	-4.688893
H	0.954445	0.377326	-5.188904
H	-0.671446	1.087096	-5.281530
H	-0.011237	0.615615	-3.716909
C	3.458543	3.097285	-1.752512
C	0.998528	2.257593	4.415070
H	1.429497	3.208057	4.719753
C	2.970334	-3.374563	-1.852319
C	-0.921515	5.436430	-0.477499
C	3.668568	-4.076864	0.394067
C	-0.599422	0.493359	4.736862
H	-1.422790	0.042257	5.286832

C	4.802019	-0.262481	-0.203215
H	5.161569	-0.288243	-1.228307
C	4.312344	-3.154159	-2.169234
H	4.570498	-2.803712	-3.166065
C	-3.651961	-3.055707	1.092379
H	-2.991510	-3.585021	0.399026
C	1.287195	5.861160	1.959903
H	1.413455	5.524096	0.925833
C	4.541952	2.411482	-2.297354
H	5.486189	2.392012	-1.762262
C	4.682374	3.335827	0.485435
H	4.513242	2.279836	0.712202
H	4.665203	3.896022	1.425729
H	5.690350	3.443519	0.068788
C	0.332059	-3.864362	4.215706
H	0.902095	-3.481671	3.362181
C	-1.098546	-5.222064	1.178134
C	-3.009895	3.081322	1.447890
H	-2.685987	3.090835	0.400941
C	1.085366	3.056264	-5.927629
H	1.573733	4.034749	-5.879009
H	0.135911	3.168931	-6.460624
H	1.724102	2.401859	-6.531239
C	-0.366349	5.660493	-1.733475
H	-0.692042	6.550122	-2.259276
C	-0.330605	5.471276	3.842784
H	0.330953	6.071779	4.463642
C	-0.097116	-0.157307	3.612126
H	-0.529721	-1.100464	3.300080
C	-3.775286	-2.643652	3.575840
H	-4.799510	-2.311751	3.423412
C	5.317247	-3.380512	-1.240339
H	6.356426	-3.202706	-1.505935
C	3.892064	5.364782	-0.774622
H	4.815645	5.460035	-1.357023
H	4.014603	5.943635	0.147783
H	3.082693	5.824432	-1.351141
C	0.514800	-5.387161	4.274191
H	-0.090266	-5.820837	5.079498
H	1.564468	-5.632991	4.474633
H	0.238878	-5.877118	3.335512
C	0.943522	-3.218878	5.462109
H	0.790060	-2.135088	5.468794
H	2.022030	-3.405735	5.476652
H	0.529812	-3.637549	6.387321
C	-2.159882	-6.075629	1.828929
H	-2.101374	-6.016628	2.918512
H	-2.053340	-7.116060	1.520532
H	-3.156689	-5.730135	1.540945
C	-1.919878	-3.037005	5.051658
H	-1.501270	-3.003509	6.052820
C	-1.479597	4.925827	4.398796
H	-1.717064	5.100561	5.444993
C	3.612456	3.888200	-0.457918
H	2.660716	3.835538	0.084938
C	4.993214	-3.837202	0.029729
H	5.785088	-4.018265	0.753165
C	-4.442547	3.626963	1.490457
H	-4.864542	3.570570	2.500011
H	-5.089325	3.040536	0.828817
H	-4.492239	4.672657	1.168418
C	5.699669	-0.279048	0.859348

H	6.770296	-0.314539	0.673274
C	-0.051862	1.704588	5.143536
H	-0.440595	2.220137	6.018198
C	4.429102	1.735449	-3.506636
H	5.284373	1.200148	-3.911624
C	-2.971387	1.630298	1.936980
H	-1.973360	1.191099	1.829742
H	-3.677536	1.020945	1.366451
H	-3.244230	1.562105	2.996611
C	2.499670	5.368687	2.758657
C	3.809991	-3.711019	2.896056
H	4.880256	-3.482361	2.834202
H	3.623436	-4.174651	3.872782
H	3.257290	-2.769508	2.841187
C	-3.743001	-1.610782	0.597213
H	-4.436681	-1.025989	1.211328
H	-4.160280	-1.625579	-0.429784
H	-2.759550	-1.136485	0.623702
C	-5.033046	-3.719072	1.038010
H	-5.021214	-4.740374	1.432904
H	-5.386847	-3.758939	0.002535
H	-5.772773	-3.153488	1.615206
C	-3.233505	-2.629194	4.851954
H	-3.832370	-2.294225	5.694985
C	4.012302	-6.039418	1.941363
H	3.688996	-6.736224	1.160418
H	3.736657	-6.470002	2.910616
H	5.105746	-5.979153	1.902277
C	1.248517	7.394638	1.943228
H	0.435222	7.776153	1.317869
H	2.188260	7.794068	1.546114
H	1.113488	7.798602	2.952955
C	1.900820	-3.136816	-2.905893
C	2.226694	-3.826961	-4.236348
H	3.080720	-3.356211	-4.736685
H	1.366714	-3.751360	-4.909032
H	2.466947	-4.888121	-4.105955
C	1.672086	-1.642390	-3.134926
H	2.442806	5.680330	3.807597
H	3.426145	5.782892	2.345683
H	2.571609	4.276617	2.734293
H	2.598534	-1.133981	-3.433893
H	1.285822	-1.153835	-2.233867
H	0.928034	-1.504647	-3.922035
H	-3.856136	1.031179	-0.959633
H	-4.493301	0.823507	-1.422508

25_TS2a.log

SCF (wB97x) = -3807.08533154

E(SCF)+ZPE(0 K)= -3805.520150

H(298 K)= -3805.425099

G(298 K)= -3805.648286

Lowest Frequency = -605.4264cm-1

W	-3.120938	-0.227646	-1.999638
Al	0.177172	2.949814	-0.511099
Al	0.355705	-2.658139	0.707993
O	-0.498018	-1.496953	-0.280642
O	-1.035639	1.892672	-1.231530
O	1.260380	-1.624086	1.765386
O	1.013659	1.748153	0.445534

C	-4.609149	-1.276809	-2.893865
N	1.175560	3.851192	-1.848811
N	-0.821284	-3.870573	1.601702
C	-1.859516	-1.646792	-2.822425
N	-0.514453	4.476919	0.339504
N	1.395723	-4.005187	-0.155313
C	-2.963916	0.931548	-3.547597
O	-5.466077	-1.861079	-3.411894
O	-1.200549	-2.445716	-3.335298
C	-1.253005	0.610801	-1.073213
O	-2.899810	1.681034	-4.442776
C	1.577983	1.693976	3.325315
H	2.392673	2.135523	2.757473
C	-1.618838	-3.411672	2.716985
C	-0.277614	-0.142171	-0.272217
C	0.873172	2.511909	-4.490749
H	0.123839	3.116584	-3.969232
C	1.041701	0.470836	2.914408
C	-0.795386	4.601691	1.753781
C	2.775280	-3.743712	-0.506497
C	1.418488	5.693006	-3.484140
H	2.402185	5.277934	-3.704557
H	1.506484	6.767849	-3.311497
H	0.780057	5.548824	-4.362203
C	-2.941240	-2.989828	2.496091
C	3.016287	-0.105745	1.370479
C	-1.953534	4.033698	2.305760
C	2.160741	2.507857	-3.675345
C	0.698326	0.448454	0.503905
C	-2.219800	4.256932	3.658988
H	-3.112668	3.820405	4.100143

C	3.272177	1.825068	-4.178783
H	3.182328	1.295356	-5.124276
C	0.933018	-5.240172	-0.381136
C	2.306766	3.178404	-2.448205
C	-1.815611	6.556590	0.083600
H	-2.760834	6.061505	0.327890
H	-2.001196	7.327316	-0.665164
H	-1.460267	7.028563	1.002442
C	0.767053	5.036052	-2.294694
C	5.296055	-0.170878	2.196620
H	5.984728	-0.191518	3.037756
C	3.924596	-0.135267	2.431245
H	3.554496	-0.139866	3.453078
C	0.094831	5.357694	2.544418
H	1.103094	-3.460192	-2.520572
C	-0.251660	-5.732227	0.176039
H	-0.535639	-6.738767	-0.103105
C	1.732529	-6.192913	-1.233567
H	1.860137	-5.785680	-2.240573
H	1.232700	-7.159352	-1.303860
H	2.734573	-6.338568	-0.820710
C	3.447376	-4.572224	1.837263
H	2.362252	-4.699174	1.914284
C	-1.044152	-3.396364	4.005082
C	1.507372	-0.248106	1.630846
C	3.515480	-0.115751	0.067689
H	2.831913	-0.085835	-0.778151
C	0.289032	1.104737	-4.630725
H	0.989875	0.442823	-5.151641
H	-0.642774	1.140464	-5.200628
H	0.062020	0.668868	-3.653416

C	3.527954	3.184613	-1.742539
C	1.084309	2.342500	4.451740
H	1.513404	3.294106	4.755409
C	3.107420	-3.295366	-1.795088
C	-0.834940	5.534960	-0.434816
C	3.765975	-3.995375	0.462532
C	-0.509123	0.574378	4.776169
H	-1.331078	0.122052	5.327081
C	4.885894	-0.163046	-0.170110
H	5.246136	-0.179585	-1.195228
C	4.454831	-3.079117	-2.090592
H	4.730432	-2.730759	-3.083473
C	-3.562455	-2.933459	1.106987
H	-2.898731	-3.443844	0.402068
C	1.386263	5.955269	1.995944
H	1.504438	5.623502	0.959126
C	4.606189	2.497334	-2.295986
H	5.556724	2.482548	-1.771934
C	4.776589	3.435933	0.480856
H	4.615435	2.379782	0.712879
H	4.766523	3.998845	1.419592
H	5.779446	3.547567	0.052932
C	0.410261	-3.798104	4.234637
H	0.984599	-3.412414	3.385375
C	-1.014239	-5.126017	1.181729
C	-2.916547	3.181334	1.497391
H	-2.591246	3.167406	0.451376
C	1.096953	3.115330	-5.885501
H	1.579593	4.096892	-5.847091
H	0.139090	3.220594	-6.404805
H	1.731510	2.463097	-6.495972
C	-0.288686	5.753869	-1.696890
H	-0.616094	6.643152	-2.222095
C	-0.227709	5.571756	3.883932
H	0.437558	6.169780	4.503346
C	-0.008914	-0.074271	3.649337
H	-0.441529	-1.017339	3.336877
C	-3.691869	-2.564379	3.594477
H	-4.714189	-2.226893	3.442302
C	5.443825	-3.305420	-1.145063
H	6.487581	-3.130076	-1.393527
C	3.963105	5.457724	-0.775061
H	4.880299	5.556342	-1.366954
H	4.092068	6.038593	0.145240
H	3.145492	5.912355	-1.343706
C	0.587573	-5.321798	4.286037
H	-0.023955	-5.757222	5.085460
H	1.634975	-5.573116	4.491483
H	0.314845	-5.805777	3.343186
C	1.017778	-3.160527	5.487175
H	0.865750	-2.076562	5.498942
H	2.095822	-3.349055	5.506189
H	0.599241	-3.583372	6.408211
C	-2.084268	-5.978433	1.819088
H	-2.027709	-5.932373	2.909447
H	-1.985980	-7.016094	1.498892
H	-3.077086	-5.619972	1.533515
C	-1.842292	-2.978042	5.070790
H	-1.426671	-2.956885	6.073457
C	-1.378882	5.031830	4.441568
H	-1.614392	5.209233	5.487839
C	3.693772	3.980602	-0.452283

H	2.748382	3.925808	0.101430
C	5.097070	-3.759285	0.119727
H	5.876203	-3.941598	0.856484
C	-4.346705	3.733632	1.518620
H	-4.779198	3.692348	2.524883
H	-4.982157	3.137878	0.855269
H	-4.389980	4.775289	1.181950
C	5.782762	-0.189819	0.892793
H	6.853441	-0.225154	0.707086
C	0.036559	1.786665	5.181931
H	-0.352151	2.301457	6.056996
C	4.480774	1.815278	-3.500689
H	5.332666	1.280415	-3.913380
C	-2.894311	1.737281	2.005474
H	-1.892758	1.297931	1.933228
H	-3.578759	1.130608	1.408844
H	-3.199135	1.681259	3.057513
C	2.600770	5.451086	2.784259
C	3.877864	-3.630651	2.965450
H	4.949396	-3.405506	2.914612
H	3.680525	-4.094854	3.939545
H	3.328793	-2.687160	2.906521
C	-3.658723	-1.475074	0.655891
H	-4.328224	-0.902260	1.305504
H	-4.117159	-1.447047	-0.352829
H	-2.675039	-1.002005	0.667173
C	-4.941696	-3.599537	1.035234
H	-4.929489	-4.629807	1.406331
H	-5.293794	-3.616139	-0.001535
H	-5.683679	-3.048224	1.623029
C	-3.155077	-2.567214	4.872334
H	-3.756351	-2.241884	5.717372
C	4.079097	-5.959858	2.012688
H	3.761842	-6.654759	1.227578
H	3.789394	-6.389522	2.978188
H	5.173226	-5.905244	1.987062
C	1.356725	7.488991	1.987887
H	0.540089	7.878405	1.371797
H	2.295350	7.885384	1.584976
H	1.232472	7.888203	3.000852
C	2.058851	-3.055786	-2.869156
C	2.406461	-3.754411	-4.189926
H	3.273962	-3.292003	-4.674243
H	1.562507	-3.676489	-4.882578
H	2.636593	-4.816424	-4.049824
C	1.846305	-1.560280	-3.108550
H	2.551271	5.756290	3.835484
H	3.527319	5.862732	2.368532
H	2.666398	4.358710	2.752924
H	2.785329	-1.057902	-3.375436
H	1.432869	-1.068258	-2.221451
H	1.133553	-1.416203	-3.923319
H	-3.696992	1.062787	-0.939499
H	-4.687728	0.527121	-1.815119

26_Int3a.log

SCF (wB97x) = -3807.08660673

E(SCF)+ZPE(0 K)= -3805.521044

H(298 K)= -3805.425397

G(298 K)= -3805.651192

Lowest Frequency = 9.8690cm-1

W	-3.177443	-0.342618	-2.068942
Al	0.086668	2.867319	-0.566479
Al	0.257470	-2.742049	0.678514
O	-0.601430	-1.582910	-0.311606
O	-1.114735	1.800930	-1.303798
O	1.144007	-1.704467	1.746303
O	0.907186	1.663933	0.409039
C	-4.516189	-1.760152	-2.571791
N	1.104410	3.762201	-1.895616
N	-0.902987	-3.967801	1.574597
C	-1.878397	-1.653968	-2.981396
N	-0.595769	4.404931	0.273330
N	1.304851	-4.071051	-0.203482
C	-3.162914	0.627318	-3.759938
O	-5.305226	-2.584346	-2.785136
O	-1.169164	-2.356805	-3.566076
C	-1.325863	0.521817	-1.130443
O	-3.188109	1.248245	-4.745854
C	1.365244	1.615458	3.316776
H	2.181271	2.074146	2.764682
C	-1.681716	-3.543730	2.717723
C	-0.376395	-0.228189	-0.304898
C	0.798076	2.458755	-4.551173
H	0.067551	3.089599	-4.034577
C	0.866712	0.379870	2.897183
C	-0.877461	4.540170	1.687068
C	2.683476	-3.809159	-0.561332
C	1.394823	5.609602	-3.518917
H	2.369674	5.173361	-3.737316
H	1.508927	6.679407	-3.330358
H	0.760148	5.494798	-4.403949
C	-3.012440	-3.132021	2.534298
C	2.894081	-0.177944	1.414458
C	-2.052330	4.005551	2.235539
C	2.078965	2.416592	-3.725480
C	0.593072	0.366733	0.478119
C	-2.320481	4.246058	3.585411
H	-3.227328	3.836744	4.024059
C	3.180986	1.717552	-4.226369
H	3.088329	1.195729	-5.176019
C	0.836063	-5.300586	-0.449418
C	2.228573	3.074839	-2.491661
C	-1.872670	6.498826	0.009624
H	-2.827411	6.013388	0.235999
H	-2.037494	7.276117	-0.737225
H	-1.526449	6.960858	0.936800
C	0.717446	4.954744	-2.342298
C	5.144076	-0.252111	2.316764
H	5.804448	-0.265192	3.180387
C	3.766024	-0.196379	2.505312
H	3.361978	-0.181172	3.514082
C	0.028715	5.278626	2.475606
H	0.989723	-3.420044	-2.546708
C	-0.347869	-5.798772	0.103402
H	-0.636552	-6.798347	-0.195118
C	1.630607	-6.242559	-1.318011
H	1.767186	-5.816548	-2.315622
H	1.122788	-7.202972	-1.409797
H	2.629186	-6.405485	-0.902630
C	3.379365	-4.682981	1.760383
H	2.293978	-4.792642	1.855265

C	-1.084913	-3.557466	3.995592
C	1.378507	-0.325980	1.625374
C	3.435696	-0.216911	0.129239
H	2.781188	-0.195804	-0.739244
C	0.163876	1.073610	-4.695377
H	0.836316	0.391246	-5.226922
H	-0.770027	1.142920	-5.259850
H	-0.069818	0.637947	-3.719310
C	3.445594	3.058549	-1.779482
C	0.832670	2.253581	4.431264
H	1.232624	3.215476	4.741822
C	3.008272	-3.334509	-1.842597
C	-0.897338	5.467975	-0.500804
C	3.684389	-4.092995	0.389200
C	-0.721883	0.445621	4.727878
H	-1.541543	-0.027169	5.264559
C	4.812338	-0.285627	-0.062115
H	5.205822	-0.327623	-1.074180
C	4.355446	-3.131486	-2.150163
H	4.623140	-2.763831	-3.138353
C	-3.644690	-3.003805	1.156334
H	-2.997221	-3.492431	0.421233
C	1.336877	5.838932	1.926546
H	1.457601	5.480341	0.899001
C	4.514396	2.353745	-2.329602
H	5.461267	2.319422	-1.799948
C	4.704454	3.311491	0.440920
H	4.548300	2.255638	0.675901
H	4.695618	3.875822	1.378878
H	5.705023	3.426910	0.008401
C	0.378221	-3.940016	4.191628
H	0.929202	-3.553635	3.327751
C	-1.101906	-5.213299	1.128215
C	-3.027510	3.170336	1.425374
H	-2.696382	3.143180	0.381421
C	1.052176	3.056042	-5.943304
H	1.563657	4.022610	-5.898222
H	0.103245	3.191116	-6.472230
H	1.671943	2.385909	-6.549329
C	-0.337230	5.682915	-1.757739
H	-0.646559	6.578528	-2.283131
C	-0.296247	5.512422	3.811343
H	0.380197	6.098563	4.429991
C	-0.182994	-0.192740	3.613096
H	-0.583310	-1.149024	3.297572
C	-3.752685	-2.761648	3.659195
H	-4.782387	-2.435511	3.535108
C	5.353196	-3.395229	-1.224523
H	6.396465	-3.232096	-1.483313
C	3.882312	5.330565	-0.808243
H	4.794497	5.430818	-1.407545
H	4.020085	5.907198	0.113467
H	3.060276	5.788985	-1.366916
C	0.569866	-5.461405	4.247894
H	-0.016085	-5.896487	5.066344
H	1.624344	-5.704947	4.424527
H	0.272253	-5.951777	3.315518
C	1.002560	-3.282801	5.425732
H	0.833355	-2.201076	5.428101
H	2.083292	-3.454955	5.426240
H	0.608263	-3.700640	6.359496
C	-2.165604	-6.079046	1.757933

H	-2.079832	-6.075508	2.847658
H	-2.086735	-7.104389	1.395633
H	-3.161613	-5.699280	1.513967
C	-1.870629	-3.188400	5.088262
H	-1.436613	-3.191144	6.083352
C	-1.464423	5.006499	4.366187
H	-1.701475	5.199234	5.409387
C	3.616145	3.852150	-0.487960
H	2.673262	3.793488	0.069814
C	5.014492	-3.870020	0.034549
H	5.799543	-4.079175	0.757753
C	-4.447157	3.748341	1.432494
H	-4.889130	3.717253	2.435137
H	-5.085327	3.160632	0.765028
H	-4.469434	4.789897	1.093001
C	5.673205	-0.304022	1.030503
H	6.748798	-0.357180	0.881326
C	-0.216334	1.673265	5.140243
H	-0.635640	2.179783	6.005988
C	4.383985	1.680247	-3.538699
H	5.228163	1.130580	-3.947794
C	-3.037984	1.728434	1.938117
H	-2.041265	1.273725	1.889176
H	-3.716370	1.134983	1.322181
H	-3.366653	1.680332	2.983499
C	2.536745	5.333175	2.735820
C	3.849452	-3.758783	2.887553
H	4.923116	-3.550835	2.814035
H	3.667268	-4.228455	3.861695
H	3.315990	-2.804766	2.850226
C	-3.705877	-1.520878	0.792402
H	-4.321224	-0.963880	1.506753
H	-4.204529	-1.390871	-0.183472
H	-2.708760	-1.076399	0.781445
C	-5.038596	-3.633852	1.058535
H	-5.046528	-4.682290	1.375617
H	-5.393711	-3.589591	0.023451
H	-5.766504	-3.097440	1.677121
C	-3.195157	-2.800334	4.927286
H	-3.788455	-2.518158	5.793248
C	3.990727	-6.082696	1.908685
H	3.647726	-6.762388	1.121018
H	3.710727	-6.519208	2.873971
H	5.085004	-6.046621	1.864527
C	1.335603	7.372422	1.880163
H	0.533855	7.761206	1.244562
H	2.286126	7.741757	1.479303
H	1.206432	7.798146	2.881672
C	1.955910	-3.046327	-2.900335
C	2.264746	-3.737642	-4.234765
H	3.142104	-3.297159	-4.721966
H	1.415096	-3.620727	-4.914789
H	2.460823	-4.808683	-4.114136
C	1.802262	-1.540393	-3.120072
H	2.488157	5.667369	3.778135
H	3.473071	5.716083	2.314561
H	2.582752	4.239411	2.734367
H	2.763340	-1.071433	-3.366633
H	1.396196	-1.045102	-2.231337
H	1.107406	-1.357397	-3.941677
H	-3.765319	0.986822	-1.036205
H	-4.816014	0.126378	-2.406599

27_TS3a.log

SCF (wB97x) = -3807.09097171

E(SCF)+ZPE(0 K)= -3805.524482

H(298 K)= -3805.429845

G(298 K)= -3805.652176

Lowest Frequency = -10.8288cm-1

W	-3.000644	0.016318	-2.500690
Al	0.048613	2.995790	-0.656748
Al	0.051084	-2.604164	0.700234
O	-0.796648	-1.431578	-0.285588
O	-1.016812	1.899224	-1.563199
O	0.990478	-1.567398	1.727395
O	0.812953	1.787665	0.356014
C	-4.996512	0.160668	-2.864952
N	1.103192	3.990948	-1.881127
N	-1.057891	-3.844456	1.639850
C	-3.452919	-1.880953	-2.308300
N	-0.750442	4.463282	0.204727
N	1.108808	-3.911943	-0.211984
C	-1.873894	-0.833489	-3.951125
O	-6.132025	0.285908	-3.029363
O	-3.694383	-3.009614	-2.147493
C	-1.241189	0.618673	-1.350272
O	-1.278493	-1.338262	-4.810437
C	1.272311	1.754233	3.294559
H	2.083334	2.205910	2.728735
C	-1.777779	-3.434400	2.827740
C	-0.465325	-0.105318	-0.359286
C	0.951268	2.904291	-4.643423
H	0.229036	3.565384	-4.154534
C	0.750318	0.525675	2.880699
C	-1.088015	4.531641	1.612391
C	2.466432	-3.626619	-0.624012
C	1.492104	5.934958	-3.348870
H	2.560025	5.712162	-3.304449
H	1.340895	7.011430	-3.256774
H	1.132278	5.624706	-4.333804
C	-3.139623	-3.095682	2.742031
C	2.759070	-0.068391	1.385188
C	-2.246459	3.917389	2.108172
C	2.180628	2.784353	-3.749238
C	0.478300	0.496284	0.449060
C	-2.582317	4.128999	3.448470
H	-3.480680	3.663452	3.846326
C	3.293967	2.083876	-4.221206
H	3.252199	1.624478	-5.205969
C	0.678809	-5.163113	-0.404764
C	2.261036	3.349236	-2.463264
C	-2.074019	6.533669	-0.032037
H	-3.046363	6.029233	-0.041651
H	-2.126230	7.410820	-0.677905
H	-1.894165	6.853257	0.995785
C	0.729714	5.207627	-2.271896
C	5.018846	-0.146441	2.260323
H	5.690122	-0.148081	3.115578
C	3.643852	-0.070593	2.465549
H	3.253662	-0.029779	3.478846
C	-0.248189	5.297040	2.449910
H	0.730645	-2.230117	-2.120300

C	-0.484634	-5.679266	0.178862
H	-0.741372	-6.697182	-0.087147
C	1.508966	-6.153586	-1.187569
H	2.114642	-5.684822	-1.962213
H	0.857310	-6.904999	-1.638335
H	2.189122	-6.669874	-0.501188
C	3.310399	-4.638261	1.589037
H	2.241674	-4.845558	1.715218
C	-1.087225	-3.401142	4.058728
C	1.241647	-0.193568	1.608596
C	3.284131	-0.148126	0.094966
H	2.613616	-0.153777	-0.762562
C	0.251676	1.557774	-4.829786
H	0.896984	0.846719	-5.356756
H	-0.665019	1.679429	-5.416721
H	-0.025446	1.123968	-3.866511
C	3.428020	3.254217	-1.676669
C	0.774888	2.389864	4.426521
H	1.196347	3.342356	4.737514
C	2.700924	-3.020898	-1.872252
C	-1.025036	5.566715	-0.522800
C	3.525557	-4.025833	0.210716
C	-0.801011	0.604817	4.743082
H	-1.615777	0.139563	5.293306
C	4.657134	-0.232274	-0.113693
H	5.037132	-0.302967	-1.129220
C	4.024742	-2.889506	-2.295410
H	4.233761	-2.447335	-3.264910
C	-3.921575	-3.103147	1.435988
H	-3.322891	-3.595390	0.662780
C	1.056279	5.923516	1.965162

H	1.260916	5.550120	0.956461
C	4.509910	2.547067	-2.198485
H	5.415394	2.443383	-1.608920
C	4.581115	3.366337	0.612232
H	4.425657	2.295548	0.762393
H	4.515671	3.861427	1.586885
H	5.601046	3.522025	0.241715
C	0.388125	-3.773993	4.163920
H	0.879937	-3.429350	3.249660
C	-1.220025	-5.107073	1.221804
C	-3.142873	3.033358	1.258752
H	-2.723513	2.964295	0.248334
C	1.304922	3.500891	-6.013615
H	1.874819	4.432661	-5.934658
H	0.392470	3.701252	-6.584486
H	1.908572	2.800860	-6.601217
C	-0.375417	5.880061	-1.715345
H	-0.665780	6.804552	-2.199028
C	-0.641180	5.498971	3.771600
H	-0.021684	6.109231	4.425369
C	-0.294976	-0.033436	3.612917
H	-0.716117	-0.981536	3.300877
C	-3.797313	-2.708599	3.912752
H	-4.847469	-2.432226	3.862496
C	5.083142	-3.301750	-1.496799
H	6.106196	-3.187068	-1.846496
C	3.816124	5.460693	-0.540370
H	4.710086	5.597074	-1.159827
H	3.996017	5.957198	0.419166
H	2.983131	5.977548	-1.026798
C	0.576743	-5.293501	4.267383

H	0.067775	-5.687837	5.155014
H	1.641851	-5.540013	4.353590
H	0.186904	-5.819538	3.389920
C	1.102164	-3.072202	5.322421
H	0.935371	-1.990571	5.294649
H	2.179701	-3.249925	5.251534
H	0.776284	-3.451145	6.298118
C	-2.220158	-6.009770	1.903070
H	-2.200899	-5.886058	2.987775
H	-2.018318	-7.052758	1.656695
H	-3.232075	-5.767039	1.564830
C	-1.795739	-3.021837	5.199201
H	-1.286918	-2.986162	6.157456
C	-1.806044	4.929745	4.269791
H	-2.098620	5.099801	5.302750
C	3.535725	3.964509	-0.329745
H	2.569476	3.869957	0.181700
C	4.831815	-3.841636	-0.245687
H	5.664036	-4.136652	0.389068
C	-4.559951	3.601991	1.120613
H	-5.072959	3.629081	2.088951
H	-5.145831	2.970737	0.444773
H	-4.558730	4.621239	0.719070
C	5.531254	-0.233128	0.969036
H	6.604269	-0.300308	0.807858
C	-0.265437	1.818837	5.155543
H	-0.655634	2.323110	6.036079
C	4.442429	1.956629	-3.455113
H	5.293676	1.400640	-3.839701
C	-3.193620	1.615558	1.833662
H	-2.195889	1.165204	1.881423
H	-3.821676	0.988042	1.199043
H	-3.604888	1.610333	2.850204
C	2.235585	5.509610	2.853407
C	3.720682	-3.643352	2.680888
H	4.769347	-3.345424	2.564192
H	3.607771	-4.097952	3.672639
H	3.107289	-2.738650	2.633746
C	-4.162813	-1.668663	0.967350
H	-4.699721	-1.095761	1.733213
H	-4.760677	-1.653790	0.051515
H	-3.215831	-1.164514	0.754746
C	-5.254413	-3.854319	1.549529
H	-5.137809	-4.855827	1.978347
H	-5.703675	-3.956084	0.556686
H	-5.969192	-3.310505	2.177078
C	-3.139270	-2.674691	5.131313
H	-3.671411	-2.378868	6.031929
C	4.059591	-5.965503	1.762813
H	3.823529	-6.680773	0.967810
H	3.793534	-6.424617	2.721213
H	5.145039	-5.817341	1.761508
C	0.966437	7.452716	1.878977
H	0.186620	7.777481	1.182965
H	1.917823	7.870845	1.531751
H	0.745389	7.889899	2.859409
C	1.559002	-2.550576	-2.765679
C	1.026867	-3.676972	-3.664143
H	1.837403	-4.107579	-4.265369
H	0.266657	-3.282138	-4.344380
H	0.563677	-4.480988	-3.085966
C	1.951261	-1.351722	-3.631333

H	2.108302	5.859361	3.883799
H	3.168816	5.942826	2.477178
H	2.349498	4.421249	2.877502
H	2.688481	-1.623778	-4.396429
H	2.368760	-0.527614	-3.040170
H	1.066897	-0.988209	-4.155580
H	-3.680575	0.746937	-0.991246
H	-3.289584	0.686322	-4.068830

28_Int4a.log

SCF (wB97x) = -3807.10497111

E(SCF)+ZPE(0 K)= -3805.537283

H(298 K)= -3805.442107

G(298 K)= -3805.665410

Lowest Frequency = 15.3774cm-1

W	-2.424208	-0.908307	-2.091713
Al	0.003997	3.041410	-0.650733
Al	0.012196	-2.530405	0.651180
O	-0.761144	-1.342129	-0.432042
O	-1.077625	1.974487	-1.572940
O	0.864063	-1.496987	1.737901
O	0.791052	1.887902	0.412420
C	-3.794720	-2.406776	-1.826191
N	1.106080	3.974768	-1.893341
N	-1.137141	-3.729198	1.609904
C	-1.646016	-2.276874	-3.398707
N	-0.756397	4.564575	0.150177
N	1.041740	-3.840238	-0.277818
C	-2.861757	-0.018073	-3.794430
O	-4.624467	-3.196121	-1.657973
O	-1.294558	-3.080762	-4.160235
C	-1.175824	0.674767	-1.420949
O	-3.116223	0.551902	-4.775753
C	1.214497	1.776144	3.381345
H	2.076581	2.194622	2.868759
C	-1.783000	-3.369361	2.864566
C	-0.392825	0.006343	-0.374635
C	0.909777	2.802864	-4.615355
H	0.193339	3.469960	-4.126183
C	0.662788	0.578304	2.922239
C	-1.118963	4.668282	1.547361
C	2.424239	-3.619179	-0.665824
C	1.533387	5.861370	-3.438278
H	2.495637	5.387880	-3.633273
H	1.690872	6.911300	-3.179903
H	0.940766	5.833599	-4.358677
C	-3.146276	-3.018714	2.895247
C	2.713875	-0.068762	1.503794
C	-2.329592	4.135224	2.013296
C	2.126636	2.648985	-3.708615
C	0.486622	0.577098	0.489404
C	-2.662465	4.323072	3.356938
H	-3.593953	3.907969	3.734312
C	3.213952	1.893518	-4.156468
H	3.151885	1.400522	-5.123977
C	0.509143	-5.040588	-0.549723
C	2.235889	3.264904	-2.448417
C	-1.990897	6.679526	-0.145365
H	-2.968147	6.200314	-0.027506
H	-2.079020	7.491259	-0.868396

H	-1.724144	7.095580	0.828671
C	0.764216	5.179329	-2.335313
C	4.931117	-0.244095	2.469441
H	5.565696	-0.289592	3.351094
C	3.551403	-0.132695	2.619844
H	3.119270	-0.104500	3.616737
C	-0.249185	5.370658	2.407371
H	0.726517	-3.107310	-2.602427
C	-0.660067	-5.518871	0.050301
H	-0.989250	-6.500148	-0.266525
C	1.199799	-5.963896	-1.521044
H	1.147474	-5.537443	-2.527734
H	0.719639	-6.942603	-1.532861
H	2.257806	-6.084487	-1.276711
C	3.152640	-4.621281	1.593311
H	2.071981	-4.598167	1.762150
C	-1.024324	-3.411912	4.055170
C	1.186803	-0.132916	1.661839
C	3.289723	-0.129357	0.233892
H	2.661118	-0.078693	-0.652593
C	0.183840	1.475950	-4.848191
H	0.829363	0.764277	-5.376098
H	-0.711403	1.634779	-5.457649
H	-0.135596	1.022474	-3.906511
C	3.404015	3.164828	-1.664526
C	0.677618	2.425731	4.487204
H	1.121402	3.356648	4.831446
C	2.746626	-3.110839	-1.935726
C	-0.986337	5.655563	-0.611882
C	3.439371	-3.981614	0.242584
C	-0.991811	0.709284	4.689107
H	-1.855528	0.276059	5.188263
C	4.667205	-0.254466	0.081179
H	5.087172	-0.309216	-0.919650
C	4.093317	-2.939451	-2.264643
H	4.349269	-2.543626	-3.244851
C	-4.012877	-2.863406	1.655809
H	-3.432292	-3.174123	0.780838
C	1.083926	5.949470	1.943002
H	1.265371	5.611858	0.917188
C	4.459650	2.403606	-2.162924
H	5.367910	2.301259	-1.577234
C	4.565332	3.345549	0.617541
H	4.361456	2.294018	0.833752
H	4.531644	3.901454	1.560346
H	5.588501	3.429640	0.233039
C	0.454837	-3.773029	4.072275
H	0.889670	-3.437567	3.128028
C	-1.339512	-4.970576	1.150029
C	-3.281371	3.365736	1.114072
H	-2.894692	3.396316	0.089152
C	1.302017	3.424060	-5.964399
H	1.867790	4.354042	-5.849756
H	0.405243	3.636167	-6.555817
H	1.921222	2.733470	-6.547473
C	-0.326149	5.904697	-1.810315
H	-0.583820	6.819732	-2.330521
C	-0.640856	5.558266	3.732145
H	0.006808	6.115572	4.405797
C	-0.449691	0.058995	3.583090
H	-0.892514	-0.867587	3.235966
C	-3.736611	-2.743970	4.132104

H	-4.789219	-2.474177	4.162940
C	5.101807	-3.283420	-1.379356
H	6.144791	-3.153544	-1.657202
C	3.876670	5.405378	-0.639532
H	4.818878	5.479611	-1.194623
H	3.993036	5.963489	0.296295
H	3.099502	5.902587	-1.227890
C	0.642503	-5.291530	4.187271
H	0.156885	-5.673515	5.093033
H	1.706662	-5.548152	4.243314
H	0.219142	-5.821506	3.326314
C	1.225087	-3.039984	5.175559
H	1.019709	-1.964755	5.145834
H	2.300083	-3.183728	5.036426
H	0.976662	-3.414934	6.174957
C	-2.321000	-5.885045	1.839167
H	-2.195103	-5.856264	2.924029
H	-2.195589	-6.910166	1.489965
H	-3.344034	-5.568415	1.622168
C	-1.664568	-3.131883	5.262499
H	-1.095086	-3.163732	6.185825
C	-1.837985	5.040674	4.208729
H	-2.123825	5.192735	5.246409
C	3.541131	3.933887	-0.353841
H	2.572057	3.900397	0.158505
C	4.769552	-3.804584	-0.137930
H	5.560815	-4.087576	0.551957
C	-4.686069	3.980875	1.095889
H	-5.165642	3.910175	2.078614
H	-5.319032	3.447798	0.379501
H	-4.669054	5.039062	0.812536
C	5.494479	-0.310851	1.198366
H	6.570957	-0.406149	1.080151
C	-0.428877	1.895488	5.145720
H	-0.849436	2.409030	6.006753
C	4.363612	1.762014	-3.392692
H	5.195577	1.166295	-3.760186
C	-3.346470	1.897093	1.538775
H	-2.356055	1.426695	1.514680
H	-3.997450	1.337864	0.861657
H	-3.733082	1.798228	2.560648
C	2.244247	5.440614	2.805648
C	3.819861	-3.847877	2.733713
H	4.906629	-3.796716	2.605870
H	3.627831	-4.343845	3.691603
H	3.439957	-2.822859	2.784106
C	-4.371035	-1.384701	1.484352
H	-4.970653	-1.033039	2.332776
H	-4.943119	-1.223443	0.565749
H	-3.469638	-0.772031	1.420630
C	-5.299581	-3.698858	1.700705
H	-5.120516	-4.752274	1.938154
H	-5.798986	-3.650342	0.728208
H	-5.995614	-3.311049	2.452987
C	-3.012820	-2.807158	5.309677
H	-3.492764	-2.598013	6.262249
C	3.583658	-6.094316	1.604487
H	3.078404	-6.670532	0.821893
H	3.342374	-6.556332	2.568382
H	4.663856	-6.191200	1.446574
C	1.068418	7.483629	1.925798
H	0.302123	7.876955	1.250420

H	2.037260	7.870163	1.590476
H	0.874071	7.887525	2.925896
C	1.698563	-2.779878	-2.980538
C	1.958456	-3.508627	-4.306331
H	2.838513	-3.101737	-4.817433
H	1.096951	-3.386170	-4.968878
H	2.131232	-4.580680	-4.163365
C	1.617409	-1.273193	-3.227667
H	2.137673	5.753810	3.850281
H	3.196730	5.841708	2.441369
H	2.301495	4.348196	2.783856
H	2.586908	-0.872188	-3.548456
H	1.305563	-0.729601	-2.329646
H	0.884479	-1.062091	-4.009989
H	-3.014569	-0.030389	-0.655890
H	-4.027246	-0.361504	-2.379959

29_TS4a.log

SCF (wB97x) = -3807.10495920

E(SCF)+ZPE(0 K)= -3805.537544

H(298 K)= -3805.443132

G(298 K)= -3805.664150

Lowest Frequency = -54.0836cm-1

W	-2.286599	-1.028446	-2.109292
Al	0.123060	2.925307	-0.657358
Al	0.134523	-2.644276	0.647611
O	-0.646109	-1.455768	-0.431409
O	-0.952620	1.858086	-1.584827
O	0.986524	-1.610726	1.733530
O	0.910618	1.774731	0.408050
C	-3.660369	-2.519379	-1.819819
N	1.225721	3.862109	-1.897667
N	-1.012423	-3.842966	1.607344
C	-1.529977	-2.415435	-3.406152
N	-0.640013	4.447227	0.144158
N	1.160242	-3.953995	-0.284917
C	-2.725626	-0.153545	-3.818671
O	-4.491981	-3.305126	-1.643196
O	-1.181828	-3.222709	-4.165904
C	-1.050862	0.557581	-1.431349
O	-2.980839	0.403473	-4.807136
C	1.343723	1.661227	3.377460
H	2.206085	2.078128	2.864035
C	-1.657397	-3.479121	2.860836
C	-0.274359	-0.107683	-0.375575
C	1.032518	2.693589	-4.621450
H	0.315241	3.359701	-4.132197
C	0.788762	0.464923	2.918315
C	-1.002096	4.549762	1.541482
C	2.542605	-3.733425	-0.673725
C	1.652088	5.750900	-3.440187
H	2.614813	5.278433	-3.635294
H	1.808668	6.800496	-3.179869
H	1.060141	5.724347	-4.361053
C	-3.018952	-3.122573	2.888383
C	2.836019	-0.183014	1.495145
C	-2.211932	4.015319	2.007852
C	2.248656	2.539424	-3.713833
C	0.605935	0.463140	0.486131
C	-2.544268	4.201887	3.351807

H	-3.475217	3.785759	3.729406	H	-0.013770	0.913297	-3.914892
C	3.336733	1.785104	-4.161796	C	3.524408	3.054058	-1.668538
H	3.275553	1.293064	-5.129851	C	0.809541	2.311520	4.484249
C	0.626168	-5.153793	-0.556506	H	1.255748	3.241301	4.828465
C	2.356584	3.154041	-2.452880	C	2.864702	-3.224178	-1.943307
C	-1.876836	6.561023	-0.150386	C	-0.870544	5.538748	-0.616984
H	-2.853814	6.080659	-0.034958	C	3.557714	-4.096413	0.234493
H	-1.964609	7.373885	-0.872201	C	-0.863670	0.598951	4.687013
H	-1.612094	6.975735	0.824769	H	-1.727954	0.167567	5.186788
C	0.882616	5.066466	-2.338871	C	4.786513	-0.366610	0.068418
C	5.055006	-0.362631	2.456165	H	5.204538	-0.419147	-0.933341
H	5.691223	-0.410894	3.336495	C	4.211374	-3.052778	-2.272280
C	3.675697	-0.250542	2.609423	H	4.467263	-2.656279	-3.252232
H	3.245460	-0.224554	3.607206	C	-3.882259	-2.973983	1.646141
C	-0.132515	5.252269	2.401643	H	-3.306308	-3.307937	0.776891
H	0.844396	-3.219528	-2.608983	C	1.199640	5.832998	1.936909
C	-0.542573	-5.631562	0.045241	H	1.381599	5.494998	0.911327
H	-0.873845	-6.611888	-0.272378	C	4.581006	2.294375	-2.167233
C	1.313964	-6.076619	-1.530266	H	5.489064	2.192297	-1.581186
H	1.255250	-5.651006	-2.537021	C	4.684932	3.233481	0.613974
H	0.835775	-7.056326	-1.538892	H	4.482587	2.181289	0.828472
H	2.373469	-6.194718	-1.291348	H	4.649981	3.787896	1.557617
C	3.270663	-4.737050	1.584730	H	5.708143	3.319742	0.230048
H	2.190135	-4.712251	1.754263	C	0.578020	-3.886341	4.070556
C	-0.899631	-3.519247	4.051944	H	1.014874	-3.551017	3.127107
C	1.309319	-0.246439	1.656599	C	-1.219679	-5.082969	1.146177
C	3.409391	-0.240744	0.224036	C	-3.163768	3.245665	1.108809
H	2.779111	-0.187125	-0.661064	H	-2.777023	3.275846	0.083870
C	0.307602	1.366483	-4.856046	C	1.425662	3.316456	-5.969460
H	0.954503	0.654961	-5.382494	H	1.990908	4.246575	-5.853267
H	-0.586208	1.524992	-5.467703	H	0.529312	3.528841	-6.561432

H	2.045682	2.626817	-6.552795	C	3.660234	3.821741	-0.356957
C	-0.209495	5.789855	-1.814452	H	2.691043	3.786366	0.155011
H	-0.467835	6.704998	-2.334153	C	4.887846	-3.919241	-0.146073
C	-0.523544	5.438426	3.726801	H	5.679209	-4.202600	0.543542
H	0.124032	5.995747	4.400512	C	-4.568438	3.860893	1.090668
C	-0.324355	-0.051965	3.580011	H	-5.047874	3.790330	2.073473
H	-0.770134	-0.977028	3.232596	H	-5.201614	3.327951	0.374345
C	-3.608852	-2.836129	4.122683	H	-4.551302	4.919055	0.807312
H	-4.659927	-2.560097	4.150951	C	5.615889	-0.426520	1.183827
C	5.219908	-3.397355	-1.387273	H	6.692063	-0.522388	1.063291
H	6.262862	-3.267281	-1.665127	C	-0.297372	1.783498	5.143772
C	3.994286	5.293884	-0.641065	H	-0.715786	2.297539	6.005557
H	4.936217	5.369615	-1.196432	C	4.486126	1.653790	-3.397606
H	4.110514	5.851086	0.295305	H	5.318792	1.059150	-3.765269
H	3.216426	5.791018	-1.228574	C	-3.228798	1.777244	1.534180
C	0.759996	-5.405690	4.183344	H	-2.238619	1.306486	1.507634
H	0.272251	-5.787318	5.088118	H	-3.882612	1.218432	0.859385
H	1.823196	-5.666211	4.239911	H	-3.612692	1.678708	2.557095
H	0.335583	-5.932803	3.321180	C	2.360926	5.326779	2.799781
C	1.350096	-3.158098	5.175634	C	3.939654	-3.966412	2.725952
H	1.149513	-2.081923	5.147017	H	5.026546	-3.917543	2.598274
H	2.424622	-3.306456	5.037587	H	3.746426	-4.463130	3.683220
H	1.098802	-3.533274	6.174247	H	3.562002	-2.940612	2.777587
C	-2.204683	-5.993879	1.835017	C	-4.216128	-1.493032	1.449327
H	-2.075549	-5.968631	2.919640	H	-4.800218	-1.114132	2.296874
H	-2.085937	-7.018766	1.482798	H	-4.795087	-1.338749	0.533736
H	-3.226740	-5.670977	1.621886	H	-3.304670	-0.897766	1.364581
C	-1.539588	-3.229255	5.257090	C	-5.180081	-3.790920	1.703422
H	-0.971193	-3.258633	6.181148	H	-5.013896	-4.841916	1.960919
C	-1.719924	4.919457	4.203721	H	-5.676538	-3.753956	0.728933
H	-2.005266	5.070409	5.241697	H	-5.873061	-3.380623	2.446495

C	-2.885960	-2.896064	5.301076
H	-3.365222	-2.678034	6.252006
C	3.698887	-6.210911	1.593693
H	3.192115	-6.785034	0.810529
H	3.457227	-6.673783	2.557090
H	4.778816	-6.309571	1.435020
C	1.181399	7.367123	1.918659
H	0.414619	7.758616	1.242766
H	2.149655	7.755152	1.583364
H	0.986017	7.771313	2.918446
C	1.816372	-2.892456	-2.987625
C	2.075272	-3.621510	-4.313432
H	2.955485	-3.215297	-4.824799
H	1.213640	-3.498728	-4.975767
H	2.247306	-4.693647	-4.170245
C	1.735442	-1.385740	-3.234834
H	2.253865	5.640263	3.844278
H	3.312608	5.729538	2.435286
H	2.420188	4.234476	2.778396
H	2.704316	-0.985206	-3.558179
H	1.426163	-0.841676	-2.336189
H	1.000369	-1.174357	-4.015130
H	-2.835968	-0.070028	-0.700536
H	-3.892376	-0.491928	-2.397984

30_Int5a.log

SCF (wB97x) = -3807.12256177

E(SCF)+ZPE(0 K)= -3805.551733

H(298 K)= -3805.456924

G(298 K)= -3805.680272

Lowest Frequency = 10.5650cm-1

W	-2.034286	-1.181855	-2.190970
Al	-0.102284	3.031152	-0.711496
Al	0.134723	-2.492362	0.713050
O	-0.656408	-1.344981	-0.414022
O	-1.182184	1.989241	-1.598797
O	0.980949	-1.417349	1.744444
O	0.762485	1.973006	0.398544
C	-2.847064	-3.037887	-1.880207
N	0.938915	4.058830	-1.943154
N	-1.124200	-3.584341	1.657947
C	-1.232611	-2.185988	-3.682714
N	-0.882305	4.521035	0.178309
N	1.088325	-3.874412	-0.147270
C	-2.834866	-0.253646	-3.754183
O	-3.350669	-4.063509	-1.698881
O	-0.786815	-2.737998	-4.607461
C	-1.299705	0.640432	-1.330939
O	-3.299284	0.302396	-4.655811
C	1.972529	1.343603	3.650768
H	2.991045	1.455345	3.291040
C	-1.799782	-3.033748	2.819845
C	-0.373331	0.035807	-0.325124
C	0.628984	2.779619	-4.605445
H	-0.111397	3.375283	-4.062303
C	1.038583	0.647016	2.889515
C	-1.276895	4.455918	1.566996
C	2.504692	-3.761962	-0.438703
C	1.201391	5.964947	-3.493994
H	2.252290	5.689805	-3.593650

H	1.119951	7.047549	-3.383757
H	0.691922	5.680303	-4.420332
C	-3.141444	-2.613980	2.735275
C	2.862451	-0.092422	1.233651
C	-2.520790	3.912704	1.923811
C	1.937611	2.807435	-3.825281
C	0.538460	0.626143	0.470111
C	-2.866102	3.877330	3.276523
H	-3.826756	3.458446	3.568457
C	3.062905	2.191336	-4.381091
H	2.966973	1.674710	-5.333202
C	0.473375	-5.035576	-0.437839
C	2.083858	3.451562	-2.583588
C	-1.947933	6.739691	0.161267
H	-2.896282	6.322269	0.508414
H	-2.144442	7.545263	-0.547201
H	-1.447573	7.159978	1.039086
C	0.537476	5.269599	-2.330771
C	5.068641	-0.897416	1.850433
H	5.715906	-1.399218	2.566136
C	3.720687	-0.734489	2.135329
H	3.309920	-1.112996	3.068784
C	-0.394915	4.962521	2.540880
H	0.997650	-3.036831	-2.465713
C	-0.718633	-5.438252	0.162093
H	-1.115908	-6.397408	-0.148354
C	1.094777	-5.967309	-1.447410
H	1.039906	-5.504425	-2.439196
H	0.564310	-6.919721	-1.478001
H	2.150894	-6.148078	-1.235820
C	2.966640	-4.920118	1.815372

H	1.916160	-4.662819	1.983114
C	-1.070510	-2.891181	4.019463
C	1.363954	-0.068044	1.561494
C	3.390983	0.378388	0.035731
H	2.753766	0.895513	-0.676111
C	0.062320	1.362563	-4.710146
H	0.751597	0.701841	-5.250436
H	-0.886758	1.375393	-5.256517
H	-0.129224	0.943198	-3.719373
C	3.329706	3.512132	-1.920820
C	1.617056	1.898941	4.881541
H	2.366827	2.427630	5.465844
C	2.955289	-3.201431	-1.644050
C	-1.089662	5.673566	-0.471320
C	3.416391	-4.254236	0.519928
C	-0.630839	1.104380	4.590121
H	-1.654831	1.006026	4.942398
C	4.741839	0.191734	-0.265288
H	5.123960	0.547510	-1.219177
C	4.332829	-3.107039	-1.857460
H	4.694040	-2.659724	-2.780261
C	-4.002503	-2.779248	1.491974
H	-3.416831	-3.294410	0.727077
C	0.955840	5.571587	2.186889
H	1.039705	5.616385	1.095472
C	4.425161	2.906973	-2.536209
H	5.397241	2.945129	-2.054626
C	4.741003	3.820944	0.190978
H	4.809637	2.737552	0.316486
H	4.720008	4.273214	1.187197
H	5.653904	4.175288	-0.302696

C	0.345233	-3.427702	4.171126
H	0.845524	-3.338234	3.204728
C	-1.382058	-4.825016	1.244864
C	-3.489739	3.354200	0.894598
H	-3.049426	3.479876	-0.100008
C	0.802823	3.377148	-6.009771
H	1.278468	4.363191	-5.991934
H	-0.171059	3.472627	-6.501017
H	1.422618	2.728566	-6.638834
C	-0.497280	5.980180	-1.699149
H	-0.762103	6.933161	-2.140657
C	-0.780720	4.898392	3.878877
H	-0.105122	5.270342	4.645878
C	-0.270521	0.541996	3.373996
H	-1.013100	-0.003431	2.794946
C	-3.723946	-2.022527	3.859694
H	-4.757675	-1.689578	3.803918
C	5.241074	-3.586075	-0.929075
H	6.308786	-3.511703	-1.118582
C	3.488506	5.761321	-0.747742
H	4.297523	6.073465	-1.418904
H	3.656475	6.242226	0.223255
H	2.546805	6.149548	-1.143308
C	0.296834	-4.913250	4.554266
H	-0.232747	-5.049283	5.504349
H	1.307351	-5.320875	4.670478
H	-0.219400	-5.510829	3.794065
C	1.196084	-2.628705	5.161192
H	1.166166	-1.558245	4.931204
H	2.236970	-2.963803	5.103534
H	0.870532	-2.772779	6.197878
C	-2.422411	-5.678754	1.925343
H	-2.700143	-5.299840	2.908919
H	-2.050893	-6.701302	2.022303
H	-3.319731	-5.711512	1.297955
C	-1.702152	-2.301469	5.113405
H	-1.151819	-2.174960	6.040301
C	-2.006717	4.363117	4.249538
H	-2.288928	4.321849	5.298504
C	3.486545	4.233570	-0.583523
H	2.622556	3.955187	0.032658
C	4.779802	-4.165424	0.244548
H	5.496104	-4.548930	0.966509
C	-4.840004	4.080269	0.911426
H	-5.345535	3.958567	1.876101
H	-5.498427	3.673179	0.136924
H	-4.731581	5.154515	0.728091
C	5.585096	-0.438217	0.639289
H	6.636463	-0.581365	0.402347
C	0.318096	1.780773	5.356238
H	0.041331	2.215573	6.313781
C	4.294743	2.240029	-3.749437
H	5.160548	1.765187	-4.204141
C	-3.685542	1.853334	1.117609
H	-2.721396	1.332762	1.135559
H	-4.297611	1.421387	0.318183
H	-4.186233	1.652689	2.072879
C	2.096892	4.688968	2.700202
C	3.762507	-4.424930	3.027035
H	4.812864	-4.731844	2.976339
H	3.349802	-4.847171	3.949705
H	3.730288	-3.334024	3.098822

C	-4.400994	-1.416520	0.923180
H	-5.031370	-0.863059	1.629078
H	-4.957632	-1.531673	-0.013309
H	-3.516529	-0.809464	0.716420
C	-5.259663	-3.615762	1.768460
H	-5.027047	-4.571767	2.246706
H	-5.786024	-3.820946	0.830256
H	-5.953219	-3.079892	2.426004
C	-3.015806	-1.860092	5.038470
H	-3.487030	-1.397832	5.902087
C	3.049724	-6.449619	1.716073
H	2.406173	-6.843380	0.923004
H	2.735886	-6.911159	2.659232
H	4.076846	-6.770613	1.507125
C	1.100260	7.003512	2.717078
H	0.292187	7.655162	2.367528
H	2.049489	7.436052	2.382035
H	1.095227	7.027738	3.812396
C	2.015125	-2.740570	-2.744507
C	2.351852	-3.416107	-4.080902
H	3.311985	-3.062678	-4.473253
H	1.580138	-3.187175	-4.820322
H	2.419555	-4.505056	-3.979760
C	2.025980	-1.216584	-2.898387
H	2.056692	4.590935	3.790989
H	3.066791	5.127235	2.436764
H	2.033768	3.681064	2.277548
H	3.037673	-0.843745	-3.102418
H	1.663599	-0.717433	-1.993750
H	1.382103	-0.917324	-3.730590
H	-2.350371	0.433116	-0.861388

H	-3.751946	-1.219381	-2.273954

31_TS5a.log

SCF (wB97x) = -3807.11819660
E(SCF)+ZPE(0 K)= -3805.547622
H(298 K)= -3805.452996
G(298 K)= -3805.676342
Lowest Frequency = -84.3405cm-1

W	-1.959031	-1.403714	-1.868788
Al	0.063075	2.891384	-0.829257
Al	0.378771	-2.563724	0.863894
O	-0.508749	-1.442110	-0.227918
O	-0.826575	1.746961	-1.788983
O	1.418674	-1.486025	1.682590
O	0.944233	1.903441	0.362075
C	-3.668711	-2.334155	-1.458208
N	1.200877	4.035891	-1.845332
N	-0.813256	-3.485384	2.010908
C	-1.666998	-3.290028	-2.508153
N	-1.026509	4.305117	-0.118818
N	1.106788	-4.059255	-0.032068
C	-2.077205	-1.047891	-3.806609
O	-4.689352	-2.799834	-1.158769
O	-1.486802	-4.357469	-2.930899
C	-1.196666	0.547650	-1.139858
O	-2.122118	-0.794572	-4.935332
C	2.914531	1.141240	3.359605
H	3.836081	1.228678	2.791935
C	-1.273730	-2.858026	3.233563

C	-0.184378	-0.058215	-0.217441
C	1.241884	2.868648	-4.552436
H	0.430335	3.434475	-4.083041
C	1.794288	0.548353	2.780964
C	-1.844371	3.951624	1.015465
C	2.471076	-4.050904	-0.527527
C	1.744223	6.063231	-3.137811
H	2.797260	6.026925	-2.847575
H	1.424625	7.104284	-3.194433
H	1.672105	5.620207	-4.135383
C	-2.525222	-2.218280	3.275285
C	3.203373	-0.242126	0.783691
C	-3.134437	3.422465	0.822292
C	2.485917	3.007592	-3.682136
C	0.797205	0.544564	0.472712
C	-3.779260	2.874686	1.933603
H	-4.770906	2.443332	1.811302
C	3.726900	2.607545	-4.186834
H	3.785801	2.201575	-5.193740
C	0.373296	-5.175362	-0.168635
C	2.444562	3.526715	-2.377159
C	-2.026468	6.542759	-0.025164
H	-3.058158	6.201590	-0.142874
H	-1.918602	7.513997	-0.509031
H	-1.847657	6.660640	1.048010
C	0.882096	5.297102	-2.163227
C	5.430249	-1.199547	0.927900
H	6.167349	-1.776233	1.481987
C	4.176258	-0.976466	1.475401
H	3.926976	-1.378660	2.454366
C	-1.282777	4.086021	2.299100
H	0.735531	-3.230184	-2.270934
C	-0.781085	-5.430495	0.582182
H	-1.291793	-6.361386	0.368692
C	0.778029	-6.218175	-1.177983
H	0.417653	-5.889896	-2.160410
H	0.317667	-7.180811	-0.951139
H	1.860851	-6.336102	-1.240040
C	3.190772	-5.167258	1.676245
H	2.181594	-4.862413	1.971184
C	-0.427498	-2.876687	4.361578
C	1.802711	-0.148981	1.405640
C	3.521503	0.260472	-0.473462
H	2.792822	0.846725	-1.026114
C	0.786628	1.406427	-4.617857
H	1.582987	0.771100	-5.024850
H	-0.093030	1.306653	-5.263564
H	0.507209	1.048758	-3.622623
C	3.598172	3.606839	-1.567996
C	2.876595	1.603880	4.675902
H	3.768172	2.046118	5.113712
C	2.771858	-3.544042	-1.802042
C	-1.061828	5.542603	-0.607852
C	3.483179	-4.570730	0.305153
C	0.571365	0.945533	4.839542
H	-0.353735	0.859289	5.404997
C	4.776854	0.018321	-1.037678
H	4.994175	0.407281	-2.028216
C	4.103847	-3.540969	-2.220249
H	4.347901	-3.138563	-3.200750
C	-3.426619	-2.066465	2.059718
H	-2.996498	-2.644260	1.233838

C	-0.021113	4.909630	2.537129
H	0.262054	5.375700	1.587084
C	4.814591	3.227894	-2.133266
H	5.721854	3.286868	-1.539381
C	4.652943	3.522691	0.756179
H	4.772292	2.442368	0.628102
H	4.423542	3.717775	1.809711
H	5.612596	4.006301	0.536136
C	0.949847	-3.524339	4.325190
H	1.335708	-3.421310	3.307139
C	-1.262094	-4.700716	1.683376
C	-3.894771	3.563071	-0.495431
H	-3.251777	4.101987	-1.199544
C	1.450756	3.427186	-5.965849
H	1.837421	4.452478	-5.958256
H	0.501446	3.425930	-6.510802
H	2.154504	2.814576	-6.540080
C	-0.209820	5.987781	-1.625632
H	-0.355684	7.004024	-1.968940
C	-1.961833	3.512707	3.374354
H	-1.526600	3.567253	4.369844
C	0.615434	0.480695	3.531220
H	-0.273973	0.033259	3.092727
C	-2.936365	-1.643590	4.480955
H	-3.902889	-1.147406	4.527555
C	5.109480	-4.055984	-1.419008
H	6.139756	-4.055344	-1.765177
C	3.480214	5.604558	0.013765
H	4.362980	6.057249	-0.454324
H	3.481169	5.888603	1.072454
H	2.586856	6.043374	-0.439453
C	0.854872	-5.019933	4.654496
H	0.416400	-5.169414	5.648111
H	1.848177	-5.483133	4.652724
H	0.236939	-5.558658	3.927253
C	1.963738	-2.822517	5.233329
H	1.983148	-1.743981	5.042890
H	2.965199	-3.221905	5.043663
H	1.749574	-2.985358	6.295906
C	-2.331053	-5.357834	2.518131
H	-2.138011	-5.224065	3.585175
H	-2.393830	-6.422666	2.291935
H	-3.302147	-4.901765	2.303414
C	-0.889036	-2.297729	5.542933
H	-0.254298	-2.306135	6.423656
C	-3.184498	2.879632	3.189093
H	-3.694880	2.423970	4.034263
C	3.521200	4.075347	-0.116144
H	2.590395	3.670248	0.295226
C	4.793655	-4.575013	-0.171209
H	5.585203	-4.985827	0.450500
C	-5.162712	4.403578	-0.267952
H	-5.898801	3.848110	0.323870
H	-5.630379	4.652158	-1.226407
H	-4.955208	5.336926	0.266522
C	5.733555	-0.705247	-0.340845
H	6.708805	-0.893554	-0.783147
C	1.712051	1.493639	5.426066
H	1.687319	1.842499	6.455575
C	4.883449	2.741832	-3.434546
H	5.843054	2.448937	-3.853847
C	-4.269743	2.237134	-1.164753

H	-3.392098	1.748378	-1.589656
H	-4.975090	2.420447	-1.983012
H	-4.753638	1.551655	-0.458141
C	1.165586	4.064988	2.996180
C	4.158223	-4.653138	2.746792
H	5.185825	-4.982305	2.558367
H	3.873358	-5.037977	3.732135
H	4.153825	-3.559745	2.787739
C	-3.465407	-0.593360	1.637540
H	-3.927646	0.019303	2.419398
H	-4.052437	-0.464628	0.720391
H	-2.459827	-0.193550	1.466506
C	-4.853346	-2.575631	2.301475
H	-4.871606	-3.597237	2.694676
H	-5.416238	-2.562016	1.362963
H	-5.383736	-1.940563	3.019834
C	-2.136775	-1.692393	5.611044
H	-2.477628	-1.248036	6.542742
C	3.209238	-6.701121	1.630233
H	2.468104	-7.096063	0.927841
H	2.988134	-7.116951	2.619752
H	4.194073	-7.069464	1.320341
C	-0.297135	6.041821	3.536191
H	-1.147697	6.662617	3.232518
H	0.582229	6.688589	3.625527
H	-0.517653	5.645688	4.533542
C	1.697284	-3.057904	-2.755699
C	1.699813	-3.856371	-4.064912
H	2.604374	-3.656482	-4.650564
H	0.836556	-3.576403	-4.677945
H	1.652046	-4.935092	-3.883470
C	1.802391	-1.559045	-3.041533
H	0.971399	3.608869	3.971686
H	2.061847	4.691047	3.093150
H	1.372464	3.258420	2.288560
H	2.774393	-1.298813	-3.478739
H	1.679329	-0.963206	-2.131367
H	1.023665	-1.259975	-3.751058
H	-2.085986	0.733634	-0.492322
H	-3.363054	-0.587966	-2.450306

32_Int6a.log

SCF (wB97x) = -3807.12760943

E(SCF)+ZPE(0 K)= -3805.555792

H(298 K)= -3805.461172

G(298 K)= -3805.682775

Lowest Frequency = 15.2300cm-1

W	-2.326206	-1.012670	-1.296404
Al	0.070157	3.080623	-0.605286
Al	0.254318	-2.302953	1.141570
O	-0.738407	-1.139580	0.199097
O	-0.814661	1.902411	-1.530386
O	1.397894	-1.275435	1.897011
O	0.908150	2.129769	0.638543
C	-4.167554	-1.523350	-0.811075
N	1.257313	4.201199	-1.586171
N	-0.818247	-3.281073	2.350789
C	-2.348457	-2.999943	-1.736305
N	-1.112506	4.470035	-0.038009
N	0.824730	-3.736682	0.043577

C	-2.523582	-0.866972	-3.248785	H	-1.534087	7.793309	0.150717
O	-5.249397	-1.766298	-0.462256	H	-1.973754	6.624776	1.419165
O	-2.348407	-4.112777	-2.061533	C	1.124466	5.526474	-1.688631
C	-1.384226	0.915041	-0.667560	C	5.482761	-0.587952	1.019219
O	-2.597254	-0.739755	-4.399266	H	6.360839	-0.835729	1.610913
C	2.580119	2.017510	3.078829	C	4.273035	-0.334743	1.654329
H	3.027247	2.451880	2.190353	H	4.208421	-0.389905	2.738316
C	-1.160200	-2.727773	3.645766	C	-2.610668	3.776206	1.796892
C	-0.362925	0.232069	0.194849	H	0.079568	-2.998697	-2.192668
C	0.675612	3.238625	-4.317214	C	-0.953235	-5.162367	0.843350
H	0.009833	3.779399	-3.634942	H	-1.481102	-6.087358	0.649473
C	1.901284	0.800756	2.979193	C	0.454052	-5.904493	-1.058807
C	-2.413509	4.049160	0.433048	H	0.169332	-5.525495	-2.046260
C	2.096514	-3.674782	-0.654093	H	-0.089454	-6.831691	-0.876536
C	1.975999	6.297504	-2.668921	H	1.526839	-6.109834	-1.082203
H	2.991123	5.908399	-2.746942	C	3.190598	-4.681164	1.449309
H	2.010837	7.353805	-2.396857	H	2.157674	-4.622998	1.808012
H	1.516618	6.218090	-3.660768	C	-0.257327	-2.911664	4.716392
C	-2.355275	-2.007475	3.814020	C	1.770691	0.065176	1.634963
C	3.127849	-0.016704	0.916363	C	3.229476	0.061978	-0.470250
C	-3.445043	3.865448	-0.512275	H	2.360209	0.336631	-1.061556
C	2.036077	3.093169	-3.644126	C	0.029607	1.874541	-4.586614
C	0.709841	0.786065	0.785841	H	0.663752	1.256473	-5.234278
C	-4.666001	3.365017	-0.058539	H	-0.935908	2.005322	-5.088757
H	-5.471545	3.205129	-0.771644	H	-0.152133	1.353732	-3.643014
C	3.080817	2.492250	-4.353129	C	3.579065	3.422153	-1.750211
H	2.894491	2.119769	-5.357979	C	2.695424	2.667030	4.303316
C	0.111778	-4.869454	-0.020110	H	3.233538	3.610199	4.362025
C	2.305877	3.559628	-2.346614	C	2.180934	-3.215443	-1.980174
C	-1.869034	6.772922	0.340298	C	-0.894420	5.772389	-0.226455
H	-2.862951	6.638722	-0.094576	C	3.245037	-4.104045	0.040468

C	1.448268	0.904874	5.357941
H	0.991783	0.458156	6.239005
C	4.439268	-0.201110	-1.111324
H	4.491210	-0.136847	-2.195118
C	3.436518	-3.182266	-2.589822
H	3.514937	-2.828253	-3.615297
C	-3.321539	-1.707479	2.676364
H	-2.986800	-2.249900	1.784203
C	-1.521975	3.988560	2.837679
H	-0.641872	4.397192	2.329372
C	4.590954	2.843911	-2.515034
H	5.581329	2.722421	-2.087433
C	5.039040	3.157896	0.329588
H	4.975571	2.072799	0.227652
H	5.074631	3.396643	1.398181
H	5.990301	3.492499	-0.102003
C	1.088284	-3.606138	4.523004
H	1.470854	-3.291837	3.544838
C	-1.331231	-4.467106	2.000928
C	-3.300572	4.218439	-1.990280
H	-2.283508	4.589163	-2.157736
C	0.780616	4.050284	-5.615768
H	1.264673	5.020677	-5.460967
H	-0.216615	4.228346	-6.031448
H	1.361112	3.511871	-6.373354
C	0.180139	6.272808	-0.970274
H	0.227477	7.348461	-1.087267
C	-3.854758	3.286213	2.199903
H	-4.022308	3.065814	3.252056
C	1.331206	0.259343	4.128632
H	0.790705	-0.676985	4.057967
C	-2.652996	-1.508515	5.085382
H	-3.571894	-0.945908	5.230780
C	4.577381	-3.590310	-1.917964
H	5.545656	-3.553206	-2.410322
C	4.080716	5.391716	-0.189620
H	4.870652	5.720719	-0.875785
H	4.405813	5.631165	0.828573
H	3.184051	5.982921	-0.385560
C	0.961608	-5.135668	4.520820
H	0.522663	-5.487984	5.461795
H	1.951273	-5.597012	4.419914
H	0.344345	-5.503753	3.695815
C	2.135371	-3.174462	5.554442
H	2.225159	-2.084604	5.605674
H	3.113434	-3.578177	5.276642
H	1.904070	-3.554416	6.556212
C	-2.355477	-5.126639	2.888312
H	-2.027167	-5.133212	3.930738
H	-2.545296	-6.149917	2.563450
H	-3.295464	-4.568355	2.849771
C	-0.612226	-2.407945	5.967749
H	0.060633	-2.541037	6.808898
C	-4.872973	3.069701	1.283005
H	-5.831939	2.678358	1.612998
C	3.848963	3.876916	-0.314355
H	2.954000	3.617180	0.265448
C	4.475944	-4.047558	-0.612439
H	5.370695	-4.374214	-0.087567
C	-4.275905	5.338157	-2.382557
H	-5.315869	5.007755	-2.282423
H	-4.120098	5.626983	-3.427413

H	-4.153468	6.234178	-1.764527
C	5.568241	-0.529503	-0.370905
H	6.511562	-0.735367	-0.871025
C	2.135322	2.110355	5.451999
H	2.233998	2.615081	6.409856
C	4.348374	2.382531	-3.804608
H	5.152979	1.927892	-4.377529
C	-3.494247	3.001433	-2.903196
H	-2.736464	2.244053	-2.688262
H	-3.398760	3.304599	-3.952835
H	-4.490456	2.560773	-2.776604
C	-1.103497	2.663185	3.479124
C	4.060340	-3.881657	2.423499
H	5.106260	-3.852374	2.098178
H	4.035813	-4.343915	3.417096
H	3.704155	-2.850148	2.505394
C	-3.298632	-0.208107	2.353159
H	-3.649714	0.377560	3.211071
H	-3.957181	0.023086	1.508065
H	-2.289130	0.139932	2.110774
C	-4.757055	-2.151751	2.987805
H	-4.812684	-3.202170	3.292457
H	-5.387314	-2.019968	2.102925
H	-5.189685	-1.553839	3.797673
C	-1.802163	-1.715943	6.157169
H	-2.055404	-1.327565	7.140083
C	3.583448	-6.164503	1.449141
H	2.947791	-6.750644	0.776530
H	3.486583	-6.585569	2.456171
H	4.622482	-6.299886	1.128535
C	-1.954974	4.992262	3.914008
H	-2.255877	5.955072	3.486385
H	-1.132478	5.173594	4.613793
H	-2.803626	4.610729	4.493018
C	0.968883	-2.786239	-2.789836
C	0.843101	-3.579304	-4.097603
H	1.638247	-3.314129	-4.803407
H	-0.114277	-3.355139	-4.579974
H	0.897489	-4.660101	-3.930401
C	0.981389	-1.282322	-3.089492
H	-1.931457	2.215945	4.043496
H	-0.268054	2.816733	4.169301
H	-0.780446	1.944604	2.720943
H	1.921902	-0.976303	-3.564636
H	0.858105	-0.680289	-2.182586
H	0.168117	-1.024338	-3.775702
H	-2.135290	1.386186	-0.001013
H	-3.558304	0.027500	-1.896151

33_TS6a.log

SCF (wB97x) = -3807.11491573

E(SCF)+ZPE(0 K)= -3805.544175

H(298 K)= -3805.449491

G(298 K)= -3805.672724

Lowest Frequency = -818.5346cm-1

W	-2.036425	-1.279599	-1.335113
Al	0.263155	2.917143	-0.535236
Al	0.489935	-2.516693	1.159849
O	-0.462991	-1.356050	0.180630
O	-0.647257	1.726104	-1.429841

O	1.609850	-1.468480	1.927213
O	1.067836	1.945687	0.710396
C	-3.943653	-1.208303	-0.966702
N	1.443961	4.011976	-1.541292
N	-0.601551	-3.483927	2.359674
C	-2.316826	-3.282767	-1.597772
N	-0.916144	4.310571	0.009892
N	1.056660	-3.960170	0.075419
C	-2.341352	-1.117537	-3.270038
O	-5.068818	-1.143742	-0.658192
O	-2.538800	-4.399598	-1.815661
C	-1.221292	0.746983	-0.589151
O	-2.435383	-0.998144	-4.424426
C	2.796120	1.816984	3.108925
H	3.255837	2.246208	2.224157
C	-0.961910	-2.926652	3.647635
C	-0.164312	0.017764	0.223782
C	0.826628	3.001560	-4.248213
H	0.160985	3.530757	-3.556631
C	2.102425	0.609386	3.003711
C	-2.218221	3.886153	0.476126
C	2.321303	-3.897816	-0.632155
C	2.169312	6.095499	-2.645206
H	3.184038	5.704875	-2.719770
H	2.205092	7.155284	-2.387522
H	1.708771	6.002706	-3.635267
C	-2.160792	-2.208786	3.796088
C	3.315632	-0.196730	0.922504
C	-3.246239	3.699445	-0.473137
C	2.199802	2.881804	-3.596327
C	0.893155	0.596448	0.821218
C	-4.454442	3.160884	-0.029116
H	-5.254474	2.988230	-0.745216
C	3.240851	2.287157	-4.316181
H	3.044434	1.906258	-5.315873
C	0.346378	-5.095332	0.016521
C	2.484130	3.361203	-2.306801
C	-1.672130	6.616651	0.359335
H	-2.646373	6.511187	-0.125968
H	-1.310601	7.634047	0.205762
H	-1.830309	6.447541	1.428195
C	1.318625	5.338922	-1.653239
C	5.674456	-0.755915	0.991973
H	6.562963	-0.995146	1.571458
C	4.473999	-0.503111	1.644631
H	4.426955	-0.551317	2.729738
C	-2.413601	3.598407	1.837217
H	0.272271	-3.282726	-2.175350
C	-0.724180	-5.382396	0.872544
H	-1.251598	-6.308157	0.682287
C	0.708978	-6.143587	-1.002002
H	0.508792	-5.761246	-2.008031
H	0.120827	-7.048897	-0.852051
H	1.772864	-6.390161	-0.954372
C	3.441504	-4.858911	1.478233
H	2.404729	-4.837477	1.830624
C	-0.077860	-3.116238	4.732381
C	1.967562	-0.125118	1.659060
C	3.395045	-0.128827	-0.466323
H	2.514488	0.134135	-1.046556
C	0.205311	1.624130	-4.507636
H	0.832668	1.025988	-5.179846

H	-0.779096	1.732678	-4.976692
H	0.067417	1.087113	-3.566048
C	3.764967	3.234782	-1.724836
C	2.912914	2.463519	4.334952
H	3.463837	3.398927	4.398369
C	2.386636	-3.462279	-1.967396
C	-0.694384	5.610244	-0.188958
C	3.480016	-4.301580	0.060476
C	1.634054	0.717722	5.379148
H	1.165710	0.276518	6.256666
C	4.595344	-0.391890	-1.124906
H	4.629233	-0.338051	-2.209911
C	3.637289	-3.421329	-2.586936
H	3.703578	-3.085583	-3.619344
C	-3.084947	-1.873440	2.633343
H	-2.716843	-2.379609	1.733572
C	-1.336174	3.833876	2.885177
H	-0.459315	4.255385	2.381608
C	4.771961	2.662121	-2.499991
H	5.768539	2.549869	-2.084521
C	5.241176	2.970200	0.341392
H	5.174887	1.885580	0.236319
H	5.285031	3.205933	1.410327
H	6.189783	3.304268	-0.096200
C	1.274746	-3.801653	4.555043
H	1.665397	-3.488625	3.579616
C	-1.113727	-4.672811	2.017026
C	-3.117329	4.098601	-1.941437
H	-2.119442	4.523582	-2.097587
C	0.893569	3.812852	-5.549361
H	1.353067	4.796268	-5.402629
H	-0.113367	3.963395	-5.952207
H	1.478494	3.288364	-6.313211
C	0.384041	6.097817	-0.936136
H	0.437793	7.172102	-1.062533
C	-3.648451	3.078426	2.231287
H	-3.814780	2.844814	3.280679
C	1.516311	0.074804	4.148688
H	0.963823	-0.854526	4.074264
C	-2.489548	-1.731664	5.068280
H	-3.414009	-1.174952	5.201192
C	4.789437	-3.798772	-1.915702
H	5.753313	-3.754990	-2.416153
C	4.283798	5.206859	-0.170877
H	5.068818	5.533373	-0.863756
H	4.617740	5.446378	0.844374
H	3.386901	5.800020	-0.360178
C	1.160495	-5.332138	4.555879
H	0.715847	-5.685730	5.493692
H	2.154804	-5.785599	4.465062
H	0.553699	-5.707135	3.726247
C	2.308010	-3.358014	5.595178
H	2.388021	-2.267283	5.643727
H	3.292192	-3.754634	5.328154
H	2.070097	-3.736536	6.595914
C	-2.146198	-5.322584	2.901871
H	-1.811977	-5.347388	3.942327
H	-2.356373	-6.338312	2.566308
H	-3.075178	-4.746281	2.876852
C	-0.459791	-2.627959	5.981844
H	0.197864	-2.765279	6.834469
C	-4.656395	2.842804	1.308052

H	-5.604110	2.418332	1.628670
C	4.047827	3.692535	-0.292429
H	3.157706	3.435919	0.296538
C	4.705390	-4.235903	-0.601943
H	5.609047	-4.539871	-0.078571
C	-4.146277	5.182008	-2.299397
H	-5.167588	4.790853	-2.234767
H	-3.989848	5.528430	-3.326397
H	-4.085398	6.051961	-1.636630
C	5.737354	-0.709152	-0.399796
H	6.673166	-0.916206	-0.913262
C	2.337772	1.913195	5.479070
H	2.437570	2.415136	6.438257
C	4.516984	2.194276	-3.784861
H	5.318318	1.745262	-4.366624
C	-3.257370	2.905898	-2.895898
H	-2.442939	2.194228	-2.738891
H	-3.219705	3.252578	-3.935486
H	-4.213289	2.388183	-2.754740
C	-0.898959	2.521967	3.541396
C	4.272413	-4.009491	2.444270
H	5.316614	-3.938619	2.119322
H	4.265447	-4.459340	3.443962
H	3.871132	-2.993757	2.512514
C	-3.047579	-0.363396	2.369003
H	-3.433705	0.189802	3.233652
H	-3.669244	-0.103708	1.505891
H	-2.027278	-0.011260	2.183949
C	-4.534617	-2.320452	2.861027
H	-4.615761	-3.388171	3.090190
H	-5.124879	-2.119638	1.960993

H	-4.994579	-1.771373	3.690505
C	-1.660473	-1.949766	6.155285
H	-1.937845	-1.577646	7.138022
C	3.894365	-6.324751	1.505014
H	3.291828	-6.946554	0.834002
H	3.801645	-6.733847	2.517310
H	4.942110	-6.423317	1.199933
C	-1.797636	4.838585	3.948913
H	-2.109494	5.792897	3.510199
H	-0.986462	5.039085	4.656613
H	-2.646632	4.447125	4.520614
C	1.161549	-3.058464	-2.772412
C	1.043990	-3.847934	-4.083063
H	1.829035	-3.564261	-4.792944
H	0.079177	-3.638776	-4.556971
H	1.120151	-4.928608	-3.922981
C	1.152991	-1.554214	-3.071196
H	-1.723410	2.061613	4.099855
H	-0.074080	2.696969	4.239302
H	-0.551819	1.804887	2.792900
H	2.080361	-1.240939	-3.567242
H	1.052686	-0.956789	-2.158108
H	0.320099	-1.301826	-3.735301
H	-1.944219	1.203278	0.108485
H	-2.470974	0.349810	-1.660672

34_Int7a.log

SCF (wB97x) = -3807.16079222

E(SCF)+ZPE(0 K)= -3805.584590

H(298 K)= -3805.489473

G(298 K)= -3805.714945

Lowest Frequency = 10.9045cm-1

W	-2.548716	-1.643002	-1.143121
Al	0.166326	3.117680	-0.717862
Al	0.250274	-2.372623	0.999552
O	-0.724780	-1.197602	0.032143
O	-0.706753	1.961047	-1.683261
O	1.378100	-1.322684	1.752440
O	0.934345	2.063353	0.469795
C	-4.372459	-0.994748	-1.206930
N	1.359429	4.259541	-1.634547
N	-0.799763	-3.338750	2.234002
C	-3.307629	-3.323634	-0.590336
N	-1.074678	4.446455	-0.175161
N	0.811536	-3.805407	-0.088724
C	-2.822234	-2.219133	-2.967007
O	-5.457771	-0.555621	-1.189672
O	-3.804168	-4.324664	-0.229970
C	-1.248887	0.863438	-1.029793
O	-2.915087	-2.578384	-4.078428
C	2.545216	1.963167	2.940358
H	3.026532	2.381622	2.062881
C	-1.096745	-2.769694	3.533465
C	-0.323124	0.144209	-0.066975
C	0.900466	3.346414	-4.407098
H	0.194383	3.848457	-3.735933
C	1.839620	0.763050	2.826969
C	-2.366576	3.956006	0.257994
C	2.028183	-3.702429	-0.868729
C	2.075407	6.394651	-2.640306
H	3.104180	6.035473	-2.666088
H	2.064130	7.447341	-2.353230
H	1.675485	6.316598	-3.657588
C	-2.258407	-2.004726	3.729140
C	3.096313	-0.041561	0.770887
C	-3.333062	3.653367	-0.726614
C	2.240955	3.225850	-3.691664
C	0.695274	0.729669	0.593882
C	-4.545436	3.105825	-0.304667
H	-5.301270	2.847982	-1.041630
C	3.324856	2.671552	-4.378757
H	3.185999	2.329614	-5.401852
C	0.133475	-4.964736	-0.104997
C	2.449863	3.655859	-2.370426
C	-1.896242	6.724822	0.233925
H	-2.922460	6.415383	0.026516
H	-1.734961	7.731774	-0.152766
H	-1.783832	6.748258	1.323796
C	1.194721	5.586249	-1.718565
C	5.462314	-0.562043	0.893940
H	6.338418	-0.796435	1.493897
C	4.240220	-0.340857	1.517899
H	4.165846	-0.408400	2.600412
C	-2.615592	3.742842	1.623377
H	-0.129883	-3.062706	-2.253480
C	-0.895203	-5.270411	0.788384
H	-1.416654	-6.203795	0.619562
C	0.498263	-6.015156	-1.120278
H	0.308023	-5.638011	-2.129554
H	-0.089453	-6.920415	-0.968213
H	1.561982	-6.262064	-1.064822

C	3.296996	-4.707766	1.134623
H	2.275405	-4.787313	1.522383
C	-0.174969	-2.983734	4.580724
C	1.731288	0.015527	1.485814
C	3.211241	0.044495	-0.615538
H	2.342476	0.289598	-1.220995
C	0.315885	1.965914	-4.729236
H	0.977388	1.402502	-5.398215
H	-0.654172	2.072386	-5.227902
H	0.161297	1.393722	-3.811556
C	3.699438	3.517696	-1.728202
C	2.651606	2.612240	4.165718
H	3.214440	3.540304	4.234567
C	1.999898	-3.227069	-2.192303
C	-0.893618	5.757359	-0.340087
C	3.236872	-4.097222	-0.260798
C	1.336681	0.884991	5.194867
H	0.853005	0.448668	6.066189
C	4.434573	-0.180346	-1.244755
H	4.495756	-0.108658	-2.327486
C	3.210570	-3.125801	-2.881484
H	3.205223	-2.761132	-3.906222
C	-3.239727	-1.696275	2.611694
H	-2.923062	-2.243429	1.719227
C	-1.593891	4.067413	2.702176
H	-0.733017	4.546591	2.222376
C	4.751333	2.980622	-2.468823
H	5.724957	2.858676	-2.004845
C	5.061904	3.182617	0.401278
H	4.994635	2.101633	0.259028
H	5.053830	3.385133	1.477823

H	6.032726	3.523351	0.021978
C	1.130876	-3.738907	4.357823
H	1.453438	-3.530953	3.331251
C	-1.295465	-4.542800	1.919046
C	-3.128983	3.948925	-2.210020
H	-2.066317	4.148611	-2.376863
C	1.016005	4.190278	-5.683634
H	1.452477	5.176035	-5.490710
H	0.027510	4.336943	-6.130998
H	1.644945	3.694104	-6.431064
C	0.196748	6.294327	-1.037126
H	0.219730	7.372323	-1.138476
C	-3.849438	3.201632	1.992188
H	-4.057838	3.025357	3.045124
C	1.229335	0.239553	3.964367
H	0.672649	-0.686663	3.888107
C	-2.504341	-1.487527	5.003849
H	-3.399894	-0.893028	5.168748
C	4.411734	-3.481650	-2.288776
H	5.343116	-3.387716	-2.841255
C	4.130425	5.439190	-0.094660
H	4.955077	5.784178	-0.730002
H	4.400127	5.653854	0.944938
H	3.244451	6.034707	-0.325627
C	0.947010	-5.255920	4.494841
H	0.593629	-5.512683	5.500317
H	1.901179	-5.770504	4.330487
H	0.228401	-5.653131	3.771329
C	2.257552	-3.256614	5.276242
H	2.378608	-2.169585	5.222615
H	3.202972	-3.718027	4.975895

H	2.082745	-3.536806	6.321156
C	-2.356297	-5.175534	2.779715
H	-2.247342	-4.922321	3.835090
H	-2.349916	-6.259767	2.660129
H	-3.329016	-4.807747	2.431546
C	-0.477523	-2.463066	5.839026
H	0.211023	-2.621715	6.663346
C	-4.804133	2.875648	1.040989
H	-5.752945	2.440616	1.343403
C	3.905055	3.929036	-0.270185
H	2.985471	3.657652	0.263730
C	4.418836	-3.966059	-0.988596
H	5.361167	-4.255206	-0.528745
C	-3.914647	5.203778	-2.618959
H	-4.988414	5.062517	-2.451153
H	-3.766448	5.417026	-3.683072
H	-3.601491	6.088388	-2.054517
C	5.563170	-0.487034	-0.494098
H	6.516948	-0.664830	-0.984876
C	2.053441	2.071729	5.302699
H	2.146690	2.573725	6.262529
C	4.570959	2.564182	-3.783139
H	5.406053	2.143584	-4.337732
C	-3.511339	2.771936	-3.113913
H	-2.952685	1.870818	-2.850747
H	-3.278535	3.017611	-4.156092
H	-4.581428	2.544260	-3.064185
C	-1.088072	2.789331	3.376024
C	4.089237	-3.825254	2.103465
H	5.104222	-3.640119	1.733500
H	4.173029	-4.317047	3.079608
H	3.600301	-2.855487	2.238019
C	-3.221468	-0.199892	2.278099
H	-3.565669	0.394675	3.133034
H	-3.888897	0.016339	1.435837
H	-2.213515	0.149048	2.022833
C	-4.667498	-2.151175	2.933677
H	-4.704866	-3.207605	3.217821
H	-5.306774	-2.012632	2.055413
H	-5.097777	-1.569093	3.756931
C	-1.634192	-1.723480	6.055142
H	-1.847892	-1.323522	7.042942
C	3.875456	-6.128616	1.094825
H	3.317497	-6.776169	0.410023
H	3.837918	-6.580697	2.092067
H	4.922119	-6.125244	0.771041
C	-2.155659	5.043378	3.743585
H	-2.545605	5.957964	3.283363
H	-1.375102	5.324964	4.457981
H	-2.973708	4.588749	4.313189
C	0.716617	-2.847399	-2.916615
C	0.512394	-3.665637	-4.199499
H	1.257558	-3.403096	-4.959226
H	-0.480407	-3.462799	-4.613542
H	0.594376	-4.742698	-4.020065
C	0.684963	-1.351366	-3.251115
H	-1.899748	2.269100	3.899142
H	-0.305264	3.017023	4.106365
H	-0.662635	2.100570	2.641047
H	1.523188	-1.074487	-3.902142
H	0.749391	-0.732369	-2.350156
H	-0.242575	-1.100156	-3.778396

H	-2.193692	1.127916	-0.513886
H	-1.504480	0.134499	-1.849092

35_TS3b.log

SCF (wB97x) = -3920.38434637

E(SCF)+ZPE(0 K)= -3918.811062

H(298 K)= -3918.713210

G(298 K)= -3918.942783

Lowest Frequency = -137.5456cm-1

W	-2.538352	-0.672833	-2.430873
Al	0.115820	2.893129	-0.482948
Al	0.223798	-2.701599	0.779600
O	-0.670572	-1.519577	-0.161988
O	-0.974797	1.797080	-1.362966
O	1.127812	-1.675054	1.842581
O	0.933524	1.701367	0.508905
C	-4.257209	-1.773584	-2.364297
N	1.155362	3.910591	-1.713582
N	-0.845889	-3.966032	1.727513
C	-3.738368	0.742255	-1.676739
N	-0.687147	4.352811	0.389699
N	1.253480	-3.981749	-0.204377
C	-2.004299	-3.479172	-1.866395
O	-5.200891	-2.439949	-2.323836
O	-4.423112	1.623095	-1.349067
O	-3.068429	0.980604	-5.013444
O	-2.120307	-4.523495	-2.303600
C	1.432013	1.597112	3.482943
H	2.275797	2.033481	2.954541
C	-1.513102	-3.577665	2.952474
C	-0.332149	-0.191589	-0.210671
C	1.028093	2.951755	-4.527650
H	0.298382	3.584036	-4.012988
C	0.878163	0.402042	3.018410
C	-1.020549	4.410296	1.797833
C	2.592873	-3.680663	-0.669704
C	1.507306	5.899825	-3.148711
H	2.480612	5.467866	-3.380736
H	1.638849	6.932493	-2.816428
H	0.910156	5.922602	-4.066168
C	-2.870914	-3.214679	2.929145
C	2.893399	-0.174861	1.514963
C	-2.202011	3.827921	2.278888
C	2.232061	2.753349	-3.612766
C	0.608742	0.402442	0.592696
C	-2.516175	3.987694	3.630660
H	-3.425774	3.535847	4.018816
C	3.338556	2.049871	-4.096778
H	3.296134	1.620699	-5.095102
C	0.830307	-5.236797	-0.392663
C	2.311543	3.277310	-2.309350
C	-2.039369	6.405796	0.174376
H	-2.987435	5.869202	0.285092
H	-2.180161	7.233042	-0.522258
H	-1.784770	6.805826	1.158352
C	0.764139	5.123951	-2.090570
C	5.155193	-0.292444	2.380683
H	5.829309	-0.320179	3.233270
C	3.781402	-0.215189	2.592263
H	3.394141	-0.200829	3.607531

C	-0.160528	5.132946	2.650999	H	-3.118836	-3.620636	0.841837
H	0.805355	-2.198288	-2.028841	C	1.141022	5.766660	2.169192
C	-0.298618	-5.779729	0.235642	H	1.328406	5.425593	1.145694
H	-0.554346	-6.795878	-0.037524	C	4.553916	2.451933	-2.061055
C	1.628132	-6.197924	-1.242073	H	5.459474	2.332674	-1.474535
H	2.155835	-5.702975	-2.057028	C	4.640531	3.258404	0.761271
H	0.967667	-6.966616	-1.648203	H	4.465680	2.192577	0.925131
H	2.377983	-6.695244	-0.617508	H	4.593088	3.766350	1.730313
C	3.533548	-4.775268	1.465637	H	5.660242	3.389580	0.380973
H	2.475061	-5.011497	1.624108	C	0.705542	-3.944554	4.192531
C	-0.772968	-3.575519	4.153654	H	1.142788	-3.654791	3.233791
C	1.377361	-0.296949	1.739731	C	-1.007392	-5.227096	1.306624
C	3.413449	-0.226140	0.221072	C	-2.895722	0.368421	-4.040832
H	2.740738	-0.201268	-0.634544	C	-3.143586	3.039205	1.386903
C	0.316343	1.636009	-4.843370	H	-2.804229	3.132643	0.349192
H	0.977242	0.951089	-5.387322	C	1.433932	3.655379	-5.831200
H	-0.565558	1.819419	-5.465359	H	1.989728	4.581566	-5.653216
H	-0.022132	1.136852	-3.932162	H	0.543046	3.895258	-6.420878
C	3.478142	3.158146	-1.525943	H	2.066232	3.005923	-6.446368
C	0.922731	2.221040	4.616632	C	-0.354951	5.774776	-1.531312
H	1.370948	3.147238	4.967565	H	-0.659921	6.695884	-2.013876
C	2.774635	-3.017985	-1.898008	C	-0.535322	5.292837	3.984230
C	-0.986878	5.453054	-0.335123	H	0.102745	5.867173	4.652525
C	3.687168	-4.111754	0.102387	C	-0.216123	-0.133115	3.697342
C	-0.732613	0.493316	4.828757	H	-0.666498	-1.053814	3.344315
H	-1.582127	0.046819	5.340465	C	-3.479587	-2.854142	4.134033
C	4.785310	-0.317795	0.006332	H	-4.526660	-2.561499	4.132054
H	5.161369	-0.366539	-1.011959	C	5.171786	-3.298008	-1.629867
C	4.080473	-2.853939	-2.364098	H	6.179568	-3.158590	-2.013230
H	4.247099	-2.361234	-3.317200	C	3.900206	5.355185	-0.403532
C	-3.693800	-3.155034	1.650118	H	4.833752	5.472932	-0.965529

H	4.016253	5.874995	0.553793
H	3.105792	5.860030	-0.961796
C	0.902468	-5.456235	4.365463
H	0.433402	-5.807992	5.292068
H	1.970741	-5.698708	4.415890
H	0.474950	-6.023052	3.531274
C	1.478178	-3.171399	5.264924
H	1.291220	-2.095685	5.183773
H	2.552177	-3.339921	5.139960
H	1.216239	-3.496076	6.278591
C	-1.980980	-6.138053	2.013258
H	-1.836458	-6.107888	3.096033
H	-1.865183	-7.164720	1.664754
H	-3.008587	-5.818606	1.817401
C	-1.433047	-3.223995	5.331214
H	-0.884473	-3.215534	6.268116
C	-1.704191	4.727040	4.475620
H	-1.977681	4.858957	5.519391
C	3.599440	3.865335	-0.179653
H	2.633895	3.787892	0.334913
C	4.972924	-3.900992	-0.398267
H	5.831249	-4.220790	0.187646
C	-4.582542	3.565445	1.441781
H	-5.033629	3.399206	2.426668
H	-5.190975	3.042606	0.698234
H	-4.633696	4.639770	1.232190
C	5.662867	-0.349524	1.085682
H	6.734968	-0.418717	0.919323
C	-0.161560	1.671609	5.295304
H	-0.560193	2.165290	6.178052
C	4.481510	1.887071	-3.329212
H	5.327374	1.330183	-3.724474
C	-3.103760	1.554447	1.755788
H	-2.095475	1.136217	1.654428
H	-3.778414	0.992563	1.105780
H	-3.415792	1.397682	2.795767
C	2.331429	5.322775	3.026866
C	3.961321	-3.805123	2.573103
H	4.997699	-3.478965	2.427024
H	3.895522	-4.292808	3.553320
H	3.328511	-2.912359	2.578816
C	-3.925552	-1.694298	1.260921
H	-4.426815	-1.153248	2.072102
H	-4.553887	-1.624633	0.367122
H	-2.975275	-1.197583	1.045277
C	-5.032733	-3.894405	1.765621
H	-4.914816	-4.923760	2.121435
H	-5.524648	-3.924282	0.788000
H	-5.712878	-3.384701	2.456723
C	-2.775230	-2.866284	5.327288
H	-3.268860	-2.592415	6.256284
C	4.317168	-6.089631	1.572709
H	4.077209	-6.782848	0.759476
H	4.086447	-6.587249	2.520873
H	5.398537	-5.916182	1.549998
C	1.053446	7.298098	2.133015
H	0.267402	7.645770	1.455220
H	2.001955	7.725766	1.789429
H	0.842842	7.704315	3.128920
C	1.597698	-2.509802	-2.720700
C	1.005824	-3.596034	-3.629212
H	1.764025	-3.973236	-4.326669

H	0.178953	-3.176503	-4.212943
H	0.610712	-4.444773	-3.063986
C	1.954782	-1.288595	-3.568126
H	2.220635	5.643040	4.068785
H	3.260893	5.763475	2.649729
H	2.440073	4.233966	3.017186
H	2.632639	-1.544372	-4.392013
H	2.425273	-0.493949	-2.976913
H	1.039913	-0.887198	-4.007557
H	-2.637519	-1.873839	-3.744986
C	-1.132948	0.507625	-1.210074
H	-0.891795	-0.698690	-3.083505

36_Int3b.log

SCF (wB97x) = -3807.12471457

E(SCF)+ZPE(0 K)= -3805.554668

H(298 K)= -3805.459840

G(298 K)= -3805.683054

Lowest Frequency = 15.2223cm-1

W	-2.133753	-1.015678	-2.063008
Al	0.053937	3.023729	-0.513619
Al	0.180028	-2.568940	0.702993
O	-0.628697	-1.362738	-0.348585
O	-1.051791	2.008075	-1.420428
O	0.994300	-1.534871	1.813445
O	0.900098	1.884060	0.529114
C	-3.404344	-2.515488	-1.635457
N	1.158433	4.031724	-1.718162
N	-0.917842	-3.792400	1.665880
C	-3.745299	0.131206	-1.895460
N	-0.704340	4.536190	0.328666
N	1.162963	-3.858103	-0.310115
O	-4.124905	-3.387566	-1.388177
O	-4.692757	0.795852	-1.810503
O	-2.715932	-0.588082	-5.115835
C	1.372702	1.698498	3.527901
H	2.228870	2.125500	3.012606
C	-1.496395	-3.434804	2.948518
C	-0.260164	-0.009064	-0.257047
C	0.983279	3.068695	-4.523600
H	0.307468	3.767651	-4.023229
C	0.807131	0.516503	3.045816
C	-1.090351	4.574986	1.720014
C	2.501590	-3.598727	-0.807244
C	1.619866	5.991945	-3.161804
H	2.572486	5.510269	-3.382755
H	1.802447	7.017044	-2.829788
H	1.035151	6.045293	-4.086080
C	-2.841797	-3.034903	3.030396
C	2.838839	-0.109252	1.587693
C	-2.303192	4.006949	2.135440
C	2.167676	2.794946	-3.599703
C	0.605775	0.557593	0.605266
C	-2.656589	4.113711	3.482213
H	-3.588758	3.669448	3.823106
C	3.229524	2.016746	-4.068828
H	3.165715	1.577136	-5.061849
C	0.660965	-5.078839	-0.530681
C	2.277521	3.335512	-2.304645
C	-1.966912	6.642211	0.105162

H	-2.939732	6.145111	0.183671	C	3.603123	-4.037480	-0.049060
H	-2.054146	7.484423	-0.582439	C	-0.824794	0.617091	4.835833
H	-1.723816	7.017431	1.101614	H	-1.686875	0.179330	5.333791
C	0.827622	5.256618	-2.108169	C	4.760598	-0.355584	0.128207
C	5.074875	-0.288318	2.509588	H	5.157928	-0.440334	-0.879878
H	5.727418	-0.319329	3.378690	C	3.981862	-2.810963	-2.532926
C	3.699505	-0.159481	2.686749	H	4.135819	-2.345375	-3.503019
H	3.291088	-0.106626	3.692192	C	-3.733099	-2.885506	1.809730
C	-0.242063	5.242962	2.626494	H	-3.228367	-3.343118	0.955202
H	0.615571	-2.489028	-2.282614	C	1.082166	5.872933	2.203101
C	-0.492488	-5.573635	0.094183	H	1.275793	5.593093	1.162074
H	-0.805227	-6.565681	-0.206845	C	4.463374	2.377672	-2.035006
C	1.396049	-6.065781	-1.405347	H	5.358211	2.210915	-1.443441
H	1.988668	-5.586976	-2.183364	C	4.607697	3.244779	0.766793
H	0.684695	-6.758057	-1.859950	H	4.398219	2.187788	0.944682
H	2.081167	-6.651796	-0.782770	H	4.580718	3.765123	1.730290
C	3.465145	-4.711017	1.309290	H	5.629702	3.338039	0.380652
H	2.401217	-4.882497	1.508699	C	0.782067	-3.901619	4.047108
C	-0.687638	-3.505046	4.102113	H	1.168074	-3.609448	3.067673
C	1.311968	-0.163092	1.763413	C	-1.146704	-5.030295	1.205135
C	3.388923	-0.207416	0.308441	C	-2.544696	-0.755575	-3.981575
H	2.737647	-0.165567	-0.561919	C	-3.222006	3.282004	1.167342
C	0.156981	1.816085	-4.822605	H	-2.846085	3.438448	0.150571
H	0.763142	1.044743	-5.310369	C	1.450770	3.715165	-5.835938
H	-0.674431	2.061274	-5.491801	H	2.080623	4.593804	-5.664168
H	-0.267625	1.404534	-3.904250	H	0.586655	4.022380	-6.434518
C	3.433052	3.159434	-1.515278	H	2.030336	3.007651	-6.439013
C	0.857703	2.321944	4.659251	C	-0.268498	5.959252	-1.567752
H	1.314672	3.239386	5.021911	H	-0.520726	6.897255	-2.048527
C	2.679854	-2.970179	-2.053776	C	-0.653503	5.350241	3.954311
C	-0.941028	5.652161	-0.389412	H	-0.022789	5.877144	4.667583

C	-0.303460	-0.008294	3.706250
H	-0.761700	-0.919363	3.339015
C	-3.369025	-2.720524	4.285165
H	-4.406475	-2.403804	4.358134
C	5.078316	-3.243940	-1.801761
H	6.083709	-3.113628	-2.194274
C	3.931298	5.354068	-0.404105
H	4.871517	5.442636	-0.960721
H	4.058076	5.870592	0.554038
H	3.155968	5.883409	-0.965490
C	0.955719	-5.418624	4.192716
H	0.544678	-5.769327	5.146748
H	2.018261	-5.688744	4.163988
H	0.453099	-5.964572	3.386109
C	1.624643	-3.147029	5.079968
H	1.444473	-2.069028	5.012424
H	2.687984	-3.326494	4.898229
H	1.413376	-3.472785	6.104727
C	-2.126836	-5.929070	1.916739
H	-1.926432	-5.954859	2.990980
H	-2.075473	-6.942473	1.517993
H	-3.146515	-5.555663	1.787074
C	-1.267814	-3.198593	5.333363
H	-0.663876	-3.249492	6.233888
C	-1.849198	4.789586	4.384254
H	-2.148957	4.877288	5.425481
C	3.582521	3.875581	-0.175663
H	2.615987	3.828581	0.339560
C	4.884384	-3.840784	-0.565581
H	5.745319	-4.167781	0.012636
C	-4.660602	3.808288	1.213976
H	-5.134237	3.602838	2.180634
H	-5.260492	3.317919	0.440596
H	-4.705595	4.890379	1.046735
C	5.611077	-0.392814	1.229288
H	6.683915	-0.501919	1.091202
C	-0.243219	1.783495	5.319204
H	-0.646803	2.276177	6.200370
C	4.357534	1.795131	-3.293204
H	5.167369	1.178388	-3.675191
C	-3.190030	1.776577	1.434870
H	-2.172871	1.374957	1.359117
H	-3.820002	1.252258	0.710368
H	-3.556745	1.549820	2.443681
C	2.253030	5.347988	3.040810
C	4.005215	-3.793816	2.411591
H	5.051387	-3.527392	2.222782
H	3.957816	-4.297922	3.383860
H	3.432060	-2.863120	2.468193
C	-3.921480	-1.399623	1.497380
H	-4.381228	-0.881584	2.347245
H	-4.568737	-1.259716	0.624953
H	-2.961903	-0.917347	1.286830
C	-5.094671	-3.572529	1.966165
H	-5.001721	-4.612756	2.296871
H	-5.619728	-3.565794	1.005924
H	-5.727060	-3.051745	2.693760
C	-2.597108	-2.810979	5.431758
H	-3.026019	-2.573638	6.401966
C	4.166216	-6.074940	1.353881
H	3.836293	-6.735083	0.544827
H	3.956758	-6.574770	2.305923

H	5.253472	-5.969610	1.270832
C	1.031429	7.405210	2.267755
H	0.258360	7.817160	1.611600
H	1.992267	7.831186	1.958146
H	0.824703	7.749878	3.287455
C	1.510276	-2.513970	-2.910296
C	1.222951	-3.486861	-4.060954
H	2.086389	-3.568053	-4.732454
H	0.365406	-3.129135	-4.640550
H	0.976035	-4.490132	-3.701634
C	1.716317	-1.105925	-3.468794
H	2.137108	5.601328	4.100837
H	3.195079	5.793050	2.700554
H	2.338018	4.260769	2.958485
H	2.562696	-1.057151	-4.163981
H	1.893010	-0.366207	-2.678833
H	0.822911	-0.802804	-4.021350
H	-1.654642	-2.548623	-2.871901
C	-1.042843	0.650841	-1.333481
H	-0.594555	0.181967	-2.350142

37_Int4b.log

SCF (wB97x) = -3807.12427409

E(SCF)+ZPE(0 K)= -3805.553869

H(298 K)= -3805.458925

G(298 K)= -3805.682933

Lowest Frequency = 10.5486cm-1

W	-2.148387	-1.266250	-1.935629
Al	-0.269313	3.072330	-0.671370
Al	0.286360	-2.449422	0.716561
O	-0.431380	-1.348223	-0.490909
O	-1.283481	1.945107	-1.545486
O	1.207002	-1.354604	1.654772
O	0.725918	2.039894	0.375921
C	-4.040367	-0.990549	-1.477786
N	0.837402	4.252428	-1.693216
N	-0.902339	-3.355417	1.876916
C	-2.966741	-0.856717	-3.695573
N	-1.338080	4.453014	0.106785
N	0.990680	-3.958218	-0.183459
O	-5.160434	-0.859605	-1.198725
O	-3.421838	-0.601817	-4.732131
O	-1.770618	-4.159729	-3.252818
C	2.766267	1.223911	3.316712
H	3.680831	1.295851	2.735469
C	-1.285209	-2.776981	3.150696
C	-0.240487	0.039570	-0.396147
C	0.964978	3.192156	-4.437500
H	0.128818	3.718263	-3.965564
C	1.624746	0.660863	2.751227
C	-2.088980	4.123243	1.296418
C	2.339136	-3.974443	-0.717663
C	1.313200	6.323978	-2.950801
H	2.365023	6.325143	-2.653588
H	0.953128	7.352450	-2.989792
H	1.265832	5.899014	-3.957196
C	-2.495445	-2.075238	3.277548
C	3.001951	-0.128920	0.735928
C	-3.391486	3.604333	1.185241
C	2.192148	3.349130	-3.548166

C	0.620645	0.678530	0.418480	H	1.364676	1.132156	-5.013242
C	-4.011341	3.156971	2.353776	H	-0.351468	1.588888	-5.104846
H	-5.012075	2.734501	2.294247	H	0.406228	1.301766	-3.531771
C	3.452106	3.012702	-4.049903	C	3.249062	3.907472	-1.397553
H	3.540212	2.651752	-5.071602	C	2.758793	1.670377	4.639405
C	0.203867	-5.033908	-0.347479	H	3.665371	2.090781	5.067978
C	2.108760	3.809404	-2.222679	C	2.622883	-3.421068	-1.977861
C	-2.390992	6.666500	0.231481	C	-1.428991	5.687071	-0.388157
H	-3.408608	6.269681	0.228421	C	3.359204	-4.559075	0.061537
H	-2.376223	7.615551	-0.305153	C	0.448084	1.037569	4.838677
H	-2.121837	6.848104	1.276592	H	-0.465516	0.951093	5.422253
C	0.477635	5.509241	-1.992286	C	4.606275	0.249685	-1.037687
C	5.174757	-1.210698	0.783878	H	4.853660	0.733338	-1.978532
H	5.875534	-1.883078	1.273521	C	3.943648	-3.439316	-2.431479
C	3.930899	-0.978753	1.351504	H	4.173691	-3.006040	-3.402067
H	3.650794	-1.467661	2.281661	C	-3.390699	-1.743780	2.095763
C	-1.470167	4.318204	2.545144	H	-3.039134	-2.295762	1.217417
H	0.590521	-2.954888	-2.371262	C	-0.165922	5.095858	2.686613
C	-0.961952	-5.256990	0.397054	H	0.104014	5.490039	1.700379
H	-1.515246	-6.155813	0.153942	C	4.485728	3.586983	-1.956796
C	0.545984	-6.066839	-1.391152	H	5.382104	3.658583	-1.348218
H	1.621027	-6.165152	-1.546172	C	4.279705	3.796230	0.933236
H	0.093797	-5.745651	-2.337610	H	4.424199	2.722724	0.776381
H	0.124111	-7.038675	-1.129725	H	4.035510	3.955108	1.989199
C	3.087986	-5.208942	1.411902	H	5.230265	4.306754	0.736389
H	2.100169	-4.883390	1.752229	C	0.945477	-3.592717	4.123751
C	-0.410453	-2.915101	4.246937	H	1.255305	-3.522385	3.078020
C	1.605374	-0.026842	1.374713	C	-1.407589	-4.539470	1.521388
C	3.358444	0.492114	-0.457192	C	-1.894095	-3.105774	-2.791255
H	2.664651	1.168877	-0.948230	C	-4.161983	3.600316	-0.130699
C	0.572086	1.715306	-4.529178	H	-3.535914	4.081689	-0.889340

C	1.161387	3.780062	-5.839835
H	1.500430	4.821346	-5.812246
H	0.218278	3.745076	-6.394332
H	1.897706	3.209407	-6.416082
C	-0.631421	6.155028	-1.438978
H	-0.821622	7.165619	-1.776723
C	-2.124924	3.840778	3.680726
H	-1.651509	3.949012	4.654072
C	0.460016	0.592102	3.523523
H	-0.438973	0.150582	3.100012
C	-2.850299	-1.584709	4.537327
H	-3.786524	-1.042975	4.647591
C	4.955466	-4.015258	-1.681446
H	5.975328	-4.030252	-2.056850
C	3.070657	5.869652	0.215080
H	3.950994	6.348193	-0.231144
H	3.047439	6.137596	1.277858
H	2.175713	6.296134	-0.247559
C	0.847909	-5.078566	4.491231
H	0.489294	-5.200829	5.519913
H	1.826507	-5.566637	4.416311
H	0.157919	-5.612971	3.827754
C	2.028609	-2.879810	4.939515
H	2.047318	-1.809043	4.708431
H	3.010952	-3.301552	4.703519
H	1.879698	-2.998686	6.018551
C	-2.491802	-5.171331	2.355070
H	-2.206377	-5.192053	3.410530
H	-2.697652	-6.187229	2.017010
H	-3.411385	-4.582923	2.283678
C	-0.813934	-2.416541	5.484828

H	-0.156685	-2.525777	6.342515
C	-3.374793	3.242205	3.585327
H	-3.869006	2.869776	4.479208
C	3.145302	4.343942	0.061866
H	2.219411	3.913960	0.456774
C	4.656680	-4.578679	-0.449149
H	5.451067	-5.039879	0.132170
C	-5.463871	4.406008	-0.003813
H	-6.169613	3.907809	0.670033
H	-5.949436	4.492240	-0.981397
H	-5.300541	5.416737	0.386024
C	5.514734	-0.600268	-0.423323
H	6.481459	-0.793924	-0.881475
C	1.606544	1.565623	5.408769
H	1.606440	1.899202	6.443708
C	4.591575	3.151280	-3.272675
H	5.566749	2.905983	-3.686291
C	-4.470910	2.191527	-0.635182
H	-3.553389	1.614142	-0.768741
H	-4.980833	2.239760	-1.603832
H	-5.133624	1.660238	0.057591
C	0.990786	4.216016	3.157185
C	4.108747	-4.781036	2.471230
H	5.112962	-5.153405	2.241651
H	3.832939	-5.186104	3.450814
H	4.161865	-3.690548	2.551299
C	-3.269355	-0.246487	1.788712
H	-3.528459	0.352678	2.669044
H	-3.944697	0.035170	0.979045
H	-2.251036	0.027846	1.488430
C	-4.861228	-2.115442	2.322009

H	-4.987120	-3.164449	2.611971
H	-5.428816	-1.944301	1.401514
H	-5.314780	-1.500877	3.107843
C	-2.031438	-1.768052	5.639243
H	-2.329839	-1.389818	6.613754
C	3.049689	-6.738563	1.294271
H	2.274555	-7.073235	0.597424
H	2.842996	-7.192969	2.269786
H	4.011016	-7.125529	0.936831
C	-0.342731	6.297643	3.624790
H	-1.173174	6.943531	3.318511
H	0.570659	6.902076	3.638768
H	-0.539220	5.973376	4.652596
C	1.546786	-2.863282	-2.891943
C	1.450997	-3.668453	-4.194347
H	2.355901	-3.546733	-4.800695
H	0.599030	-3.325276	-4.790911
H	1.320383	-4.738299	-4.003430
C	1.761796	-1.379727	-3.197246
H	0.803956	3.821678	4.160630
H	1.920355	4.798159	3.196172
H	1.139463	3.363088	2.489458
H	2.728825	-1.205241	-3.684224
H	1.735002	-0.774606	-2.285234
H	0.977474	-1.022690	-3.873190
H	-2.871749	-2.606295	-1.087022
C	-1.188091	0.582478	-1.410094
H	-0.757911	0.122648	-2.428054

38_TS4b.log

SCF (wB97x) = -3807.11986928
E(SCF)+ZPE(0 K)= -3805.550274
H(298 K)= -3805.456023
G(298 K)= -3805.676912
Lowest Frequency = -32.7788cm-1

W	-2.016952	-1.208970	-1.965722
Al	-0.084294	3.057458	-0.579580
Al	0.414386	-2.489228	0.750941
O	-0.345111	-1.371255	-0.420749
O	-1.098441	1.955485	-1.481624
O	1.357156	-1.407681	1.686401
O	0.890057	1.996027	0.449332
C	-3.603996	-0.367145	-2.786040
N	1.025913	4.240694	-1.585614
N	-0.728010	-3.422400	1.946830
C	-1.817984	-1.830343	-3.842930
N	-1.137449	4.425224	0.232171
N	1.127492	-3.996628	-0.141889
O	-4.546420	0.144683	-3.223999
O	-1.707059	-2.143001	-4.957194
O	-2.811514	-4.283516	-1.611184
C	2.893876	1.111649	3.419521
H	3.827362	1.161488	2.867087
C	-1.125489	-2.814120	3.202036
C	-0.126568	0.015047	-0.319561
C	1.124932	3.167924	-4.326736
H	0.286350	3.672622	-3.835946
C	1.749991	0.600173	2.810417
C	-1.866042	4.074151	1.429672
C	2.464126	-3.990639	-0.703526

C	1.497118	6.321670	-2.825705	H	0.271659	-7.106658	-1.068544
H	2.551472	6.318104	-2.537382	C	3.281355	-5.127213	1.457799
H	1.139072	7.351150	-2.855835	H	2.299921	-4.796730	1.811273
H	1.440529	5.901626	-3.833846	C	-0.242326	-2.883013	4.298538
C	-2.364774	-2.162590	3.309695	C	1.742743	-0.073273	1.426256
C	3.135026	-0.154303	0.774951	C	3.454391	0.427133	-0.448951
C	-3.157823	3.526268	1.333592	H	2.738002	1.070665	-0.951707
C	2.362302	3.338255	-3.455064	C	0.763935	1.682600	-4.421990
C	0.754196	0.638244	0.486744	H	1.549985	1.123409	-4.943644
C	-3.747555	3.048702	2.506108	H	-0.178919	1.539067	-4.961315
H	-4.740855	2.607482	2.456789	H	0.649952	1.252698	-3.424091
C	3.617810	3.005165	-3.970011	C	3.436665	3.875686	-1.309094
H	3.696454	2.650020	-4.994584	C	2.862623	1.537655	4.748578
C	0.397208	-5.120935	-0.242794	H	3.771069	1.915757	5.211207
C	2.290836	3.793911	-2.127513	C	2.705999	-3.482283	-1.989952
C	-2.181992	6.638827	0.395399	C	-1.228070	5.665872	-0.245484
H	-3.196123	6.234178	0.416488	C	3.512429	-4.525045	0.076592
H	-2.186235	7.588102	-0.141074	C	0.522178	1.002397	4.863739
H	-1.889080	6.822700	1.433819	H	-0.413714	0.949927	5.415039
C	0.667952	5.501855	-1.866546	C	4.694712	0.189116	-1.046361
C	5.332447	-1.186857	0.816981	H	4.911902	0.641851	-2.009808
H	6.057608	-1.824967	1.317055	C	4.016961	-3.497938	-2.473496
C	4.094305	-0.961882	1.400017	H	4.217882	-3.101099	-3.465823
H	3.842539	-1.425397	2.350985	C	-3.290670	-1.942007	2.125633
C	-1.229625	4.262662	2.670880	H	-2.907615	-2.501979	1.265316
H	0.648357	-3.056286	-2.367632	C	0.066745	5.055004	2.802042
C	-0.713937	-5.392870	0.559143	H	0.317280	5.464963	1.817187
H	-1.231133	-6.323549	0.362884	C	4.668365	3.555773	-1.879200
C	0.761416	-6.154930	-1.278246	H	5.568389	3.615526	-1.274611
H	1.839191	-6.308067	-1.352304	C	4.456029	3.694624	1.020631
H	0.413807	-5.798098	-2.254887	H	4.559925	2.618525	0.848887

H	4.216733	3.848051	2.078517	H	2.182982	-1.701815	4.774273
H	5.425254	4.171801	0.831763	H	3.188486	-3.165818	4.770243
C	1.134976	-3.520806	4.191583	H	2.049381	-2.894644	6.086133
H	1.448862	-3.447568	3.147778	C	-2.201243	-5.302244	2.545105
C	-1.169962	-4.649748	1.661303	H	-1.923595	-5.222071	3.599189
C	-2.508596	-3.180849	-1.787673	H	-2.324629	-6.353106	2.282045
C	-3.951842	3.520461	0.032270	H	-3.164561	-4.796869	2.426755
H	-3.332580	3.982530	-0.743758	C	-0.661824	-2.355559	5.518843
C	1.286370	3.770368	-5.727015	H	0.001423	-2.407873	6.377247
H	1.591846	4.821871	-5.696046	C	-3.092278	3.130083	3.727750
H	0.338610	3.709193	-6.271248	H	-3.562069	2.733369	4.624332
H	2.034168	3.227330	-6.315051	C	3.343909	4.294959	0.155200
C	-0.435478	6.144126	-1.296076	H	2.402615	3.893016	0.542444
H	-0.625826	7.158913	-1.621147	C	4.797864	-4.540331	-0.462329
C	-1.854735	3.755860	3.810260	H	5.614880	-4.960490	0.118752
H	-1.365635	3.858493	4.776433	C	-5.238912	4.347078	0.177576
C	0.558815	0.572760	3.543735	H	-5.931755	3.870613	0.880183
H	-0.345436	0.179567	3.083836	H	-5.749154	4.421893	-0.788278
C	-2.732933	-1.636184	4.551113	H	-5.052947	5.362433	0.543967
H	-3.690865	-1.130680	4.647508	C	5.634896	-0.615664	-0.418892
C	5.056116	-4.024083	-1.724678	H	6.596493	-0.805439	-0.889196
H	6.066981	-4.036521	-2.123813	C	1.682850	1.470174	5.479707
C	3.319020	5.820345	0.324380	H	1.662922	1.789283	6.519017
H	4.215963	6.274242	-0.114343	C	4.763863	3.135437	-3.200732
H	3.299524	6.079738	1.389304	H	5.735233	2.891633	-3.623966
H	2.440245	6.279049	-0.139199	C	-4.297941	2.111902	-0.449873
C	1.083947	-5.005675	4.573154	H	-3.398108	1.507617	-0.585843
H	0.714371	-5.130992	5.597690	H	-4.818364	2.158303	-1.412122
H	2.081569	-5.456806	4.518820	H	-4.961572	1.607761	0.262881
H	0.426859	-5.573874	3.904722	C	1.240506	4.182086	3.243496
C	2.194133	-2.772259	5.006684	C	4.322461	-4.656461	2.478436

H	5.323818	-5.032105	2.241265
H	4.068571	-5.029152	3.476771
H	4.370350	-3.564426	2.520088
C	-3.288860	-0.457585	1.749294
H	-3.624801	0.159770	2.590867
H	-3.954718	-0.280559	0.901972
H	-2.288831	-0.110693	1.461819
C	-4.726496	-2.413064	2.387346
H	-4.770497	-3.451527	2.732580
H	-5.314526	-2.336106	1.466827
H	-5.219454	-1.794088	3.145386
C	-1.901935	-1.745809	5.653863
H	-2.210328	-1.342239	6.615059
C	3.254309	-6.660967	1.402270
H	2.454555	-7.033193	0.754612
H	3.092581	-7.076160	2.403443
H	4.204890	-7.053111	1.022288
C	-0.109132	6.241748	3.759288
H	-0.951256	6.882317	3.474528
H	0.797574	6.856223	3.768509
H	-0.286929	5.900922	4.785138
C	1.603397	-2.958511	-2.894662
C	1.504905	-3.770998	-4.193324
H	2.392652	-3.626260	-4.819759
H	0.631275	-3.452072	-4.771592
H	1.413795	-4.844591	-3.997625
C	1.804039	-1.474889	-3.213815
H	1.076002	3.778019	4.246832
H	2.165094	4.772655	3.269439
H	1.383362	3.335213	2.566653
H	2.772643	-1.299004	-3.697148
H	1.770635	-0.862512	-2.306358
H	1.022976	-1.127772	-3.898179
H	-3.418130	-1.543067	-0.994579
C	-1.042477	0.594133	-1.344586
H	-0.594278	0.124586	-2.354722

39_Int5b.log

SCF (wB97x) = -3807.12739478
E(SCF)+ZPE(0 K)= -3805.556455
H(298 K)= -3805.462006
G(298 K)= -3805.682981
Lowest Frequency = 15.3537cm-1

W	-2.079760	-1.110194	-1.859281
Al	-0.284809	3.117018	-0.308538
Al	0.453005	-2.404766	0.690746
O	-0.217360	-1.289323	-0.528366
O	-1.374857	2.022953	-1.126070
O	1.430528	-1.376393	1.653762
O	0.743483	2.059929	0.666108
C	-3.397049	-0.401067	-3.155898
N	0.827356	4.194457	-1.415795
N	-0.816847	-3.273598	1.784784
C	-1.862053	-2.494906	-3.343630
N	-1.366642	4.513415	0.396398
N	1.146156	-3.911120	-0.214994
O	-4.157937	0.057938	-3.900695
O	-1.804821	-3.211114	-4.252552
O	-3.710158	-3.487899	-0.566823
C	2.737874	1.768840	2.981308

H	3.251769	2.202437	2.129293
C	-1.168246	-2.755543	3.092506
C	-0.119706	0.089285	-0.283665
C	0.094298	2.906509	-3.951142
H	-0.545588	2.763149	-3.074904
C	1.929783	0.645781	2.794848
C	-2.530455	4.156329	1.185120
C	2.461907	-3.876056	-0.825326
C	1.531163	6.270227	-2.554971
H	2.592778	6.167327	-2.324159
H	1.269747	7.329119	-2.553761
H	1.392194	5.872521	-3.562795
C	-2.100563	-1.711281	3.230656
C	3.138676	-0.076055	0.677534
C	-3.731745	3.841658	0.517883
C	1.537926	3.020289	-3.459022
C	0.725316	0.699148	0.570043
C	-4.842005	3.504392	1.293769
H	-5.778463	3.260499	0.798246
C	2.586918	2.561601	-4.261496
H	2.367780	2.145445	-5.240502
C	0.377295	-4.999182	-0.355686
C	1.866976	3.568606	-2.204091
C	-2.082511	6.841702	0.733539
H	-3.140060	6.581183	0.656439
H	-1.918676	7.823197	0.287674
H	-1.847894	6.898964	1.801886
C	0.676005	5.515405	-1.562481
C	5.495173	-0.652351	0.731719
H	6.375507	-0.940836	1.301898
C	4.284997	-0.459761	1.384924

H	4.219886	-0.608734	2.459945
C	-2.448527	4.153535	2.587600
H	0.516193	-3.402963	-2.565243
C	-0.844671	-5.180686	0.312197
H	-1.397848	-6.075146	0.054128
C	0.822614	-6.115926	-1.265497
H	1.887952	-6.325686	-1.145138
H	0.661799	-5.827831	-2.309078
H	0.250639	-7.023084	-1.068026
C	3.446630	-4.448731	1.475809
H	2.663221	-3.793818	1.875494
C	-0.519951	-3.297111	4.220964
C	1.786746	-0.029417	1.420921
C	3.240594	0.104598	-0.699999
H	2.373360	0.424860	-1.271628
C	-0.121032	1.686154	-4.855640
H	0.304661	1.834763	-5.855275
H	-1.194198	1.507697	-4.982162
H	0.322383	0.780939	-4.427940
C	3.186603	3.555086	-1.701995
C	2.922906	2.310388	4.248695
H	3.573674	3.172598	4.375453
C	2.612385	-3.569707	-2.189525
C	-1.204208	5.803681	0.080590
C	3.585682	-4.137721	-0.010812
C	1.451537	0.649100	5.170269
H	0.936776	0.199925	6.016656
C	4.449746	-0.112451	-1.362678
H	4.496924	0.028942	-2.439534
C	3.900786	-3.562042	-2.730397
H	4.029574	-3.326535	-3.784059

C	-2.764420	-1.028957	2.047747
H	-2.291425	-1.398233	1.133933
C	-1.174471	4.516107	3.334733
H	-0.463681	4.937796	2.615004
C	4.192777	3.099706	-2.554631
H	5.221209	3.081424	-2.208621
C	4.815892	3.312848	0.243189
H	4.845702	2.233517	0.070195
H	4.917043	3.488789	1.319639
H	5.694733	3.764495	-0.232542
C	0.589753	-4.335401	4.123779
H	0.721544	-4.608676	3.071334
C	-1.357589	-4.428238	1.381188
C	-3.113034	-2.636929	-1.081530
C	-3.887343	3.898151	-0.997521
H	-2.896447	4.023497	-1.441984
C	-0.398268	4.167686	-4.675436
H	-0.509703	5.018991	-3.997867
H	-1.380926	3.980247	-5.121085
H	0.287344	4.452003	-5.483340
C	-0.250355	6.271039	-0.831369
H	-0.245408	7.338440	-1.011620
C	-3.586592	3.802147	3.316481
H	-3.537437	3.786536	4.402709
C	1.275942	0.102686	3.900596
H	0.643274	-0.768801	3.768501
C	-2.409877	-1.262856	4.517066
H	-3.132077	-0.458464	4.632436
C	5.009594	-3.845459	-1.953644
H	6.003674	-3.838084	-2.393093
C	3.605413	5.467232	-0.009610
H	4.314886	5.936921	-0.702272
H	3.973186	5.647057	1.006234
H	2.643444	5.977346	-0.094242
C	0.261376	-5.618570	4.896149
H	0.169673	-5.426299	5.970879
H	1.058068	-6.358138	4.759306
H	-0.677182	-6.070681	4.558883
C	1.911354	-3.731265	4.616547
H	2.187320	-2.849465	4.027234
H	2.718089	-4.469314	4.546117
H	1.836102	-3.424040	5.665574
C	-2.531779	-5.001659	2.128845
H	-2.202350	-5.424324	3.083507
H	-3.009953	-5.787512	1.543443
H	-3.264258	-4.223579	2.354039
C	-0.864458	-2.815318	5.484355
H	-0.372664	-3.228346	6.362206
C	-4.775390	3.478650	2.679813
H	-5.652897	3.211652	3.263087
C	3.514981	3.953011	-0.258359
H	2.696370	3.556475	0.356442
C	4.847419	-4.124796	-0.603647
H	5.725141	-4.324559	0.002046
C	-4.751920	5.094805	-1.418639
H	-5.755627	5.027947	-0.983166
H	-4.861727	5.116696	-2.508128
H	-4.316172	6.050850	-1.108771
C	5.580747	-0.487775	-0.650889
H	6.524151	-0.653413	-1.165060
C	2.287311	1.745510	5.352514
H	2.439871	2.162008	6.345263

C	3.902779	2.631210	-3.829949
H	4.706185	2.286846	-4.476553
C	-4.464209	2.595375	-1.557986
H	-3.859096	1.741511	-1.243643
H	-4.465459	2.622712	-2.652884
H	-5.499765	2.437211	-1.234141
C	-0.529896	3.267018	3.940323
C	4.720752	-4.146324	2.269475
H	5.518213	-4.864798	2.047075
H	4.516795	-4.214293	3.341811
H	5.091134	-3.139320	2.056747
C	-2.559654	0.490223	2.092434
H	-3.162898	0.955952	2.879930
H	-2.860183	0.936630	1.137946
H	-1.511032	0.745760	2.275414
C	-4.259232	-1.357991	1.960196
H	-4.441315	-2.432119	1.853877
H	-4.698882	-0.858471	1.089543
H	-4.788084	-1.008223	2.854648
C	-1.810666	-1.812234	5.639463
H	-2.068849	-1.451148	6.631613
C	3.024142	-5.905917	1.712589
H	2.040058	-6.130176	1.290198
H	2.977464	-6.120993	2.786797
H	3.752504	-6.592731	1.265434
C	-1.421160	5.576199	4.415799
H	-1.934260	6.459505	4.019138
H	-0.468863	5.899891	4.848339
H	-2.032368	5.180484	5.234210
C	1.450349	-3.230127	-3.110256
C	1.437128	-4.101988	-4.373527
H	2.271852	-3.850774	-5.037269
H	0.509257	-3.941603	-4.930457
H	1.516744	-5.169034	-4.140774
C	1.487496	-1.746837	-3.499952
H	-1.229752	2.747727	4.606135
H	0.356519	3.532999	4.523135
H	-0.213709	2.564185	3.163341
H	2.428526	-1.497752	-4.005031
H	1.394723	-1.107131	-2.617396
H	0.666003	-1.512888	-4.187199
H	-3.540065	-0.606232	-1.116010
C	-1.154152	0.676883	-1.174898
H	-0.760420	0.370636	-2.270624

40_TS5b.log

SCF (wB97x) = -3807.11624730

E(SCF)+ZPE(0 K)= -3805.546171

H(298 K)= -3805.451992

G(298 K)= -3805.673573

Lowest Frequency = -760.0027cm-1

W	-1.963720	-1.149892	-1.907339
Al	-0.262266	3.113590	-0.268200
Al	0.517728	-2.406342	0.731752
O	-0.083027	-1.297450	-0.524369
O	-1.316561	1.975516	-1.099200
O	1.472530	-1.362448	1.706725
O	0.760365	2.056247	0.713261
C	-3.401515	-0.442420	-3.035503
N	0.827221	4.181661	-1.397232

N	-0.776684	-3.252873	1.806527
C	-1.726969	-2.565211	-3.348738
N	-1.364680	4.491611	0.423676
N	1.211256	-3.918479	-0.164015
O	-4.242176	0.015331	-3.702466
O	-1.666666	-3.329784	-4.220740
O	-3.885994	-3.160401	-0.453100
C	2.788878	1.787043	3.008868
H	3.305521	2.212801	2.154675
C	-1.127655	-2.772893	3.128386
C	-0.029671	0.067401	-0.272333
C	0.128872	2.884439	-3.942881
H	-0.523861	2.753992	-3.074359
C	1.980266	0.663206	2.830014
C	-2.495912	4.129834	1.258037
C	2.537153	-3.893292	-0.752746
C	1.504882	6.255288	-2.556211
H	2.561875	6.198882	-2.289542
H	1.205246	7.303319	-2.592427
H	1.413079	5.825350	-3.555664
C	-2.042214	-1.719520	3.306134
C	3.195641	-0.077505	0.724477
C	-3.705219	3.757170	0.636527
C	1.565494	3.009254	-3.432339
C	0.782148	0.703715	0.597757
C	-4.779105	3.405467	1.456410
H	-5.720513	3.113748	0.998333
C	2.624682	2.556243	-4.224604
H	2.416655	2.135766	-5.204069
C	0.413949	-4.978600	-0.338700
C	1.879895	3.562088	-2.175344
C	-2.109709	6.816755	0.730120
H	-3.157469	6.511958	0.744136
H	-2.017612	7.775663	0.219147
H	-1.802232	6.948546	1.773344
C	0.652048	5.498167	-1.564264
C	5.547644	-0.668006	0.802579
H	6.418790	-0.968652	1.380359
C	4.330984	-0.474867	1.442420
H	4.250937	-0.633889	2.514958
C	-2.372428	4.177401	2.656936
H	0.627207	-3.399483	-2.532975
C	-0.834804	-5.127228	0.293121
H	-1.410591	-5.995966	-0.000980
C	0.840382	-6.099604	-1.251753
H	1.916088	-6.278396	-1.195617
H	0.601268	-5.834034	-2.286841
H	0.305515	-7.017572	-1.005104
C	3.475094	-4.487512	1.560671
H	2.674339	-3.848342	1.951663
C	-0.503700	-3.376484	4.239820
C	1.840650	-0.023340	1.460699
C	3.314726	0.115053	-0.649783
H	2.456916	0.441523	-1.231974
C	-0.068188	1.651164	-4.834872
H	0.361493	1.795942	-5.833221
H	-1.137864	1.456764	-4.964804
H	0.384321	0.754941	-4.397787
C	3.196022	3.563239	-1.663418
C	2.973086	2.337511	4.272255
H	3.624583	3.199958	4.393750
C	2.715299	-3.584063	-2.113352

C	-1.228983	5.779164	0.080405
C	3.644893	-4.158413	0.082027
C	1.501383	0.682109	5.204688
H	0.988099	0.238241	6.054530
C	4.531644	-0.100390	-1.299163
H	4.592265	0.049754	-2.374140
C	4.014841	-3.575978	-2.627355
H	4.163874	-3.340021	-3.678254
C	-2.657737	-0.949136	2.152365
H	-2.229713	-1.330591	1.221247
C	-1.092705	4.610663	3.355790
H	-0.412200	5.023104	2.602127
C	4.212083	3.111936	-2.506502
H	5.237985	3.103639	-2.153203
C	4.826214	3.361776	0.286470
H	4.876476	2.281498	0.122296
H	4.923835	3.548807	1.361288
H	5.696361	3.825952	-0.192876
C	0.581267	-4.436569	4.107373
H	0.758475	-4.620425	3.042741
C	-1.353753	-4.377259	1.357788
C	-3.181748	-2.427494	-1.021596
C	-3.907376	3.771103	-0.874551
H	-2.927626	3.844580	-1.353386
C	-0.357472	4.133075	-4.693168
H	-0.486655	4.993655	-4.030677
H	-1.330146	3.932698	-5.154413
H	0.341546	4.409539	-5.492120
C	-0.292027	6.246221	-0.848843
H	-0.308772	7.310091	-1.048066
C	-3.476022	3.811558	3.430136
H	-3.395258	3.833239	4.514335
C	1.329284	0.123835	3.939510
H	0.703532	-0.754097	3.813954
C	-2.364884	-1.330591	4.608843
H	-3.076622	-0.521371	4.752992
C	5.108329	-3.859614	-1.829381
H	6.110996	-3.851052	-2.248937
C	3.572431	5.490165	0.021428
H	4.274996	5.970784	-0.670654
H	3.932384	5.682357	1.037748
H	2.600521	5.980177	-0.069414
C	0.172077	-5.770637	4.742650
H	0.001031	-5.663960	5.819744
H	0.962175	-6.516981	4.603542
H	-0.745501	-6.168283	4.296627
C	1.892525	-3.922854	4.715203
H	2.219795	-2.999540	4.223419
H	2.682415	-4.674831	4.611720
H	1.778372	-3.712398	5.784175
C	-2.590678	-4.896206	2.041160
H	-2.382385	-5.153857	3.083447
H	-2.978216	-5.774810	1.525201
H	-3.360818	-4.120001	2.042802
C	-0.860213	-2.953318	5.520430
H	-0.390587	-3.419647	6.383613
C	-4.669866	3.424909	2.839831
H	-5.519313	3.144724	3.457318
C	3.513629	3.973346	-0.220596
H	2.702830	3.563953	0.396230
C	4.918748	-4.143177	-0.483962
H	5.783424	-4.346395	0.139195

C	-4.738045	4.988189	-1.306348
H	-5.721414	4.982546	-0.821992
H	-4.898505	4.969865	-2.389483
H	-4.247421	5.935612	-1.058912
C	5.651473	-0.488279	-0.577073
H	6.600208	-0.654070	-1.081170
C	2.334504	1.781723	5.378964
H	2.484688	2.206340	6.368584
C	3.936031	2.636304	-3.782146
H	4.747214	2.295562	-4.420782
C	-4.556118	2.480305	-1.381300
H	-4.011980	1.599182	-1.030406
H	-4.552248	2.456168	-2.475238
H	-5.600021	2.394947	-1.057838
C	-0.393422	3.411760	4.000840
C	4.726471	-4.181306	2.388239
H	5.537328	-4.887610	2.176045
H	4.498538	-4.264177	3.454601
H	5.090266	-3.167581	2.195489
C	-2.312724	0.541891	2.258267
H	-2.843786	1.014034	3.092305
H	-2.613387	1.068642	1.344797
H	-1.240788	0.697828	2.413593
C	-4.177641	-1.128094	2.070096
H	-4.465586	-2.178179	1.965662
H	-4.566693	-0.589166	1.198791
H	-4.667380	-0.724710	2.964447
C	-1.794553	-1.945302	5.711743
H	-2.065260	-1.631904	6.716662
C	3.062457	-5.953101	1.759231
H	2.095782	-6.178708	1.298385

H	2.982891	-6.190585	2.826576
H	3.812408	-6.622598	1.321915
C	-1.349716	5.708199	4.396687
H	-1.902523	6.555874	3.976665
H	-0.399316	6.081568	4.791701
H	-1.927793	5.327375	5.245591
C	1.574200	-3.248578	-3.060848
C	1.579315	-4.144299	-4.307562
H	2.428089	-3.910369	-4.960007
H	0.661955	-3.990218	-4.883094
H	1.649113	-5.206691	-4.051879
C	1.631492	-1.773526	-3.477916
H	-1.056078	2.909343	4.715513
H	0.502172	3.732315	4.540731
H	-0.082192	2.677011	3.251721
H	2.579853	-1.542590	-3.978226
H	1.534184	-1.118283	-2.607371
H	0.817307	-1.546655	-4.175970
H	-2.614157	-0.016970	-0.713344
C	-1.046925	0.660681	-1.176698
H	-0.716596	0.388305	-2.287606

41_Int6c.log

SCF (wB97x) = -3920.44183652

E(SCF)+ZPE(0 K)= -3918.858996

H(298 K)= -3918.762952

G(298 K)= -3918.986663

Lowest Frequency = 12.9693cm-1

W	-3.045857	0.636392	-1.584115

Al	0.249653	2.531917	-0.790796
Al	0.380580	-3.020532	0.673997
O	-0.527070	-1.913454	-0.356449
O	-1.053534	1.517892	-1.462127
O	1.050613	-1.936273	1.844605
O	0.925869	1.397590	0.349819
C	-5.015017	0.214443	-1.310372
N	1.178373	3.187562	-2.349998
N	-0.583030	-4.547262	1.349772
C	-3.039977	-1.174075	-2.487014
N	-0.117034	4.227500	-0.011011
N	1.783576	-4.062091	-0.136016
C	-3.297675	1.454081	-3.435834
O	-6.128844	0.007223	-1.098217
O	-3.026950	-2.177578	-3.056706
O	-3.101589	0.011994	1.583416
O	-3.534392	1.972819	-4.439775
C	-0.143834	1.264312	3.328234
H	0.510103	2.014214	2.892149
C	-1.516980	-4.411120	2.448994
C	-0.228040	-0.572030	-0.387093
C	-0.081819	1.926038	-4.710544
H	-0.605491	2.029061	-3.753893
C	-0.042644	-0.070909	2.924127
C	-0.310471	4.425501	1.412583
C	3.033896	-3.402663	-0.427794
C	1.837305	4.971191	-3.958571
H	1.383084	5.896447	-4.316978
H	1.892089	4.258198	-4.777819
H	2.865867	5.195478	-3.656671
C	-2.897704	-4.347415	2.195411
C	2.409834	-0.082033	2.271724
C	-1.607784	4.358267	1.950693
C	1.415757	1.845690	-4.404257
C	0.552219	0.076425	0.512442
C	-1.787434	4.789393	3.265271
H	-2.782658	4.773315	3.699660
C	2.282514	1.279043	-5.340995
H	1.885093	0.912799	-6.283222
C	1.710421	-5.369719	-0.375106
C	1.982336	2.347734	-3.214122
C	-0.415288	6.689541	-0.074820
H	0.444787	7.045612	0.502491
H	-1.256575	6.616391	0.614052
H	-0.637721	7.430636	-0.844241
C	1.082288	4.462693	-2.747379
C	4.062372	0.406156	3.983657
H	4.295777	0.590392	5.029541
C	2.745827	0.152453	3.606766
H	1.972417	0.133116	4.368073
C	0.798709	4.805443	2.189228
H	1.057609	-2.591466	-2.065142
C	0.618425	-6.160821	0.005798
H	0.654147	-7.202219	-0.288651
C	2.872166	-6.111698	-0.993320
H	3.464921	-5.492954	-1.666340
H	2.508837	-6.990459	-1.530306
H	3.532862	-6.460894	-0.192045
C	4.002523	-4.257534	1.799891
H	3.062471	-4.822034	1.801508
C	-1.003401	-4.383625	3.763275
C	0.990864	-0.524713	1.871028

C	3.433390	-0.086335	1.322540
H	3.197406	-0.300342	0.280672
C	-0.599531	0.648951	-5.387339
H	-0.161487	0.505897	-6.381076
H	-1.681344	0.717407	-5.523814
H	-0.391943	-0.245064	-4.793357
C	3.344714	2.174551	-2.903647
C	-1.046961	1.636068	4.320083
H	-1.083144	2.671532	4.640608
C	3.146159	-2.700036	-1.640904
C	-0.070897	5.367413	-0.721647
C	4.098980	-3.492950	0.484592
C	-1.806360	-0.630475	4.499576
H	-2.450181	-1.390047	4.936920
C	4.751859	0.151091	1.699159
H	5.537437	0.114303	0.950144
C	4.361440	-2.071903	-1.922074
H	4.477679	-1.511159	-2.845354
C	-3.475368	-4.404241	0.786946
H	-2.709428	-4.811755	0.118999
C	2.221105	4.816320	1.639154
H	2.164386	4.757015	0.546731
C	4.158948	1.576276	-3.868515
H	5.217228	1.439865	-3.669484
C	5.218752	1.879921	-1.210444
H	5.094689	0.794672	-1.292713
H	5.497851	2.112441	-0.178974
H	6.052837	2.185244	-1.853439
C	0.497576	-4.429110	4.044378
H	1.001122	-3.902793	3.227825
C	-0.390932	-5.797713	0.898128
C	-2.988678	0.211684	0.460932
C	-2.784735	3.840416	1.133778
H	-2.395559	3.075401	0.450987
C	-0.461083	3.132166	-5.584245
H	-0.322877	4.085963	-5.069371
H	-1.517935	3.064893	-5.860323
H	0.131295	3.149983	-6.507435
C	0.386694	5.448713	-2.036724
H	0.356614	6.430858	-2.491044
C	0.571559	5.179966	3.515424
H	1.412020	5.475127	4.138522
C	-0.886339	-1.001559	3.522397
H	-0.817202	-2.037761	3.228909
C	-3.762086	-4.231226	3.286545
H	-4.832769	-4.164705	3.112995
C	5.428608	-2.149531	-1.034257
H	6.369084	-1.657619	-1.272448
C	4.212522	4.136105	-1.500796
H	4.874111	4.440282	-2.321262
H	4.719123	4.377940	-0.559296
H	3.304547	4.742792	-1.543388
C	1.033567	-5.866736	4.083249
H	0.515596	-6.455670	4.849821
H	2.102472	-5.862872	4.327951
H	0.919814	-6.378902	3.122514
C	0.888893	-3.687943	5.326477
H	0.466583	-2.678379	5.347890
H	1.978754	-3.597476	5.375991
H	0.565042	-4.222219	6.227522
C	-1.266399	-6.923691	1.399959
H	-1.265056	-6.963466	2.492256

H	-0.913498	-7.879338	1.011123	H	-4.255901	-3.053766	-0.717589
H	-2.304194	-6.782074	1.087002	H	-2.881286	-2.407972	0.192500
C	-1.912380	-4.296136	4.818239	C	-4.705650	-5.314193	0.690093
H	-1.544766	-4.272260	5.839728	H	-4.524579	-6.304994	1.122280
C	-0.712808	5.210398	4.039016	H	-4.988530	-5.445195	-0.359184
H	-0.875666	5.538244	5.062650	H	-5.570067	-4.881285	1.204863
C	3.931314	2.626984	-1.565621	C	-3.279851	-4.215241	4.586075
H	3.197593	2.380109	-0.784973	H	-3.969835	-4.137375	5.422438
C	5.294238	-2.852060	0.154099	C	5.148021	-5.266427	1.958382
H	6.130552	-2.896813	0.847693	H	5.244369	-5.925986	1.089032
C	-3.438971	4.919313	0.261345	H	4.980186	-5.889664	2.843299
H	-3.790243	5.757497	0.876053	H	6.109673	-4.759672	2.094975
H	-4.300670	4.492518	-0.262157	C	2.979088	6.104584	1.979723
H	-2.758987	5.309398	-0.501323	H	2.421517	7.001523	1.687972
C	5.072798	0.410273	3.029177	H	3.941500	6.120015	1.456750
H	6.103632	0.600319	3.318337	H	3.191624	6.178059	3.051729
C	-1.886606	0.693469	4.908866	C	1.991003	-2.663670	-2.635499
H	-2.588481	0.992114	5.683688	C	1.917763	-3.936241	-3.493303
C	3.642332	1.166015	-5.089820	H	2.875820	-4.123098	-3.993690
H	4.298887	0.729355	-5.838150	H	1.151884	-3.817903	-4.267113
C	-3.851535	3.166532	2.000825	H	1.653755	-4.823048	-2.912906
H	-3.413724	2.441967	2.694942	C	2.052643	-1.451721	-3.555599
H	-4.560076	2.639917	1.352946	H	3.053070	3.563185	3.220231
H	-4.424570	3.901786	2.578286	H	4.025888	3.601056	1.741829
C	2.998049	3.585512	2.125431	H	2.527453	2.655466	1.792087
C	3.961653	-3.302940	2.999530	H	2.888168	-1.513871	-4.261419
H	4.854910	-2.668030	3.031208	H	2.150464	-0.515994	-2.996004
H	3.923984	-3.877530	3.933756	H	1.139351	-1.402085	-4.154432
H	3.086781	-2.649315	2.949732	H	-3.901890	2.174439	-1.243479
C	-3.796980	-2.999277	0.275919	C	-0.880951	0.112640	-1.551926
H	-4.492185	-2.484945	0.950250	H	-0.454802	-0.170558	-2.520702

42_TS6c.log

SCF (wB97x) = -3920.43353165

E(SCF)+ZPE(0 K)= -3918.851255

H(298 K)= -3918.755751

G(298 K)= -3918.977239

Lowest Frequency = -136.3632cm-1

W	-2.859735	1.273942	-1.496360
Al	-0.010165	2.638407	-0.675774
Al	0.328458	-2.907637	0.935892
O	-0.621161	-1.879490	-0.125554
O	-0.689176	1.331323	-1.766471
O	0.877741	-1.777099	2.118609
O	0.503479	1.550733	0.613920
C	-4.780232	1.728825	-1.010328
N	0.985805	3.405246	-2.166980
N	-0.598824	-4.434696	1.598326
C	-3.592054	-0.505140	-2.152247
N	-0.377603	4.324311	0.204387
N	1.751421	-3.895540	0.103340
C	-3.128475	1.933613	-3.441589
O	-5.843661	2.025207	-0.677605
O	-4.006415	-1.488982	-2.595214
O	-2.980059	0.668237	1.647067
O	-3.407623	2.362492	-4.472367
C	-0.371862	1.387419	3.608008
H	0.311313	2.139075	3.222183
C	-1.520385	-4.295879	2.704950
C	-0.371577	-0.533822	-0.184829
C	-0.006016	2.259700	-4.767213
H	-0.610200	2.731336	-3.986402
C	-0.287886	0.067166	3.154406
C	-0.480550	4.522510	1.643081
C	2.963460	-3.186829	-0.237015
C	1.463128	5.277921	-3.756061
H	0.711712	5.780822	-4.370941
H	2.010585	4.566416	-4.368472
H	2.164240	6.044425	-3.411369
C	-2.891212	-4.102726	2.464222
C	2.172817	0.118610	2.502149
C	-1.741436	4.533593	2.275356
C	1.423264	2.125446	-4.251538
C	0.300779	0.197385	0.742293
C	-1.803794	4.965743	3.600088
H	-2.767931	5.005431	4.098394
C	2.356256	1.513917	-5.091906
H	2.032951	1.153873	-6.065595
C	1.704352	-5.206160	-0.131734
C	1.869559	2.615890	-3.005976
C	-0.735102	6.790122	0.172893
H	0.169405	7.203383	0.631322
H	-1.482465	6.701861	0.958166
H	-1.082775	7.499627	-0.580481
C	0.785505	4.671115	-2.544243
C	3.823135	0.616877	4.214003
H	4.056765	0.794410	5.260933
C	2.510946	0.342584	3.839302
H	1.741074	0.301003	4.603083
C	0.689447	4.862653	2.346338
H	0.901848	-2.491931	-1.825843

C	0.629688	-6.020801	0.254455	H	1.963420	4.660339	0.642906
H	0.681176	-7.059454	-0.047650	C	4.091858	1.803237	-3.468477
C	2.877572	-5.916891	-0.763911	H	5.128356	1.668523	-3.177203
H	3.429901	-5.287071	-1.461113	C	5.012769	2.304404	-0.809379
H	2.537662	-6.818702	-1.277073	H	4.928452	1.213977	-0.859252
H	3.571446	-6.223937	0.026975	H	5.226443	2.580865	0.226986
C	4.049427	-4.015763	1.943109	H	5.871553	2.614160	-1.416647
H	3.161953	-4.659149	1.949334	C	0.482046	-4.525541	4.310004
C	-1.005377	-4.365973	4.014522	H	1.033817	-4.403911	3.371855
C	0.765140	-0.367909	2.112632	C	-0.399503	-5.676186	1.132378
C	3.192384	0.143035	1.549077	C	-2.832170	0.878306	0.527512
H	2.958758	-0.064620	0.504871	C	-3.018211	4.105046	1.561956
C	-0.609718	0.880774	-5.067015	H	-2.730165	3.340337	0.840021
H	0.024666	0.311150	-5.754796	C	-0.090406	3.124157	-6.034198
H	-1.590225	0.987743	-5.538789	H	0.352440	4.114028	-5.900395
H	-0.729904	0.297390	-4.151554	H	-1.139252	3.258479	-6.318635
C	3.207476	2.442662	-2.596132	H	0.422144	2.642139	-6.874679
C	-1.302924	1.742896	4.579238	C	0.011634	5.587412	-1.821954
H	-1.326905	2.766235	4.938963	H	-0.085620	6.570478	-2.267552
C	3.000608	-2.464909	-1.445212	C	0.576314	5.235237	3.688465
C	-0.393535	5.473332	-0.492051	H	1.474019	5.489850	4.246921
C	4.063733	-3.232724	0.636120	C	-1.182635	-0.864908	3.674456
C	-2.131800	-0.506415	4.628036	H	-1.130083	-1.892379	3.344798
H	-2.812776	-1.263600	5.008535	C	-3.744671	-3.998799	3.564523
C	4.508483	0.397638	1.925188	H	-4.807356	-3.839977	3.398761
H	5.292779	0.382830	1.174856	C	5.265850	-1.791445	-0.898527
C	4.168801	-1.763201	-1.751680	H	6.168198	-1.241551	-1.156866
H	4.222480	-1.181351	-2.667494	C	3.973918	4.505543	-1.339227
C	-3.467282	-3.975097	1.061909	H	4.639096	4.742521	-2.177971
H	-2.687182	-4.252478	0.344528	H	4.459787	4.848933	-0.418993
C	2.075104	4.866143	1.712309	H	3.052283	5.081143	-1.457370

C	0.793409	-5.927728	4.848460
H	0.262451	-6.115686	5.788855
H	1.866724	-6.032163	5.042384
H	0.503589	-6.709666	4.137761
C	0.986793	-3.442881	5.270419
H	0.822773	-2.446262	4.850353
H	2.060721	-3.566432	5.443781
H	0.486689	-3.498137	6.243649
C	-1.303433	-6.794401	1.590669
H	-1.293778	-6.887015	2.680021
H	-0.995611	-7.742952	1.149683
H	-2.337956	-6.588487	1.300446
C	-1.902904	-4.278603	5.079251
H	-1.528190	-4.341379	6.097927
C	-0.660658	5.319043	4.305637
H	-0.735988	5.643705	5.340370
C	3.725349	2.990973	-1.269702
H	2.965886	2.796591	-0.499633
C	5.210769	-2.521890	0.279169
H	6.070729	-2.531973	0.944415
C	-3.685354	5.224504	0.750975
H	-3.878798	6.106958	1.373941
H	-4.647090	4.870468	0.363918
H	-3.085757	5.526560	-0.111995
C	4.829868	0.648958	3.255838
H	5.858402	0.853735	3.542988
C	-2.190819	0.800569	5.091982
H	-2.915632	1.086076	5.850651
C	3.679658	1.357201	-4.715408
H	4.389448	0.883581	-5.388918
C	-4.047176	3.483910	2.512944
H	-3.596373	2.729846	3.165017
H	-4.836665	2.999394	1.928094
H	-4.533348	4.243712	3.136892
C	2.951124	3.759839	2.309976
C	3.937014	-3.075807	3.149132
H	4.772366	-2.366609	3.175351
H	3.956766	-3.656172	4.079600
H	3.008106	-2.498719	3.112682
C	-3.847065	-2.518365	0.777155
H	-4.578647	-2.152956	1.507623
H	-4.283393	-2.430469	-0.222872
H	-2.962448	-1.878227	0.820001
C	-4.667973	-4.901662	0.834972
H	-4.439705	-5.945545	1.078128
H	-4.980912	-4.857165	-0.213027
H	-5.527581	-4.601977	1.444413
C	-3.262555	-4.100456	4.861398
H	-3.945312	-4.030783	5.704419
C	5.276472	-4.926600	2.078624
H	5.413040	-5.568730	1.201346
H	5.172784	-5.568260	2.959903
H	6.195395	-4.344498	2.207707
C	2.764575	6.230410	1.846563
H	2.162082	7.041350	1.423969
H	3.727234	6.222047	1.324096
H	2.963905	6.476816	2.895041
C	1.824728	-2.486476	-2.415974
C	1.827046	-3.749405	-3.291028
H	2.783903	-3.856216	-3.816407
H	1.035452	-3.681623	-4.044898
H	1.647590	-4.660714	-2.715622

C	1.782390	-1.257891	-3.318589
H	3.078108	3.894277	3.390379
H	3.949863	3.772976	1.856745
H	2.513968	2.771628	2.141296
H	2.605305	-1.253048	-4.041546
H	1.828028	-0.323379	-2.748959
H	0.854436	-1.259275	-3.897907
H	-2.599219	3.056098	-1.278068
C	-0.960316	0.034111	-1.427349
H	-0.880275	-0.646807	-2.275775

43_Int7c.log

SCF (wB97x) = -3920.45365571
E(SCF)+ZPE(0 K)= -3918.870696
H(298 K)= -3918.773909
G(298 K)= -3919.000625
Lowest Frequency = 8.8956cm-1

W	-3.080447	1.537199	-1.703129
Al	-0.305630	2.413266	-0.918316
Al	0.629474	-3.253043	0.774978
O	-0.241708	-2.301561	-0.402525
O	-0.805951	0.837236	-1.954270
O	0.980065	-2.014810	1.934938
O	0.376984	1.209525	0.366199
C	-4.725883	2.478735	-1.201630
N	0.801309	3.053750	-2.402305
N	-0.318471	-4.773485	1.387498
C	-4.124244	-0.023670	-2.356841
N	-0.128998	4.092842	0.145495
N	2.117166	-4.206477	0.049810
C	-3.335945	2.384116	-3.556543
O	-5.686717	3.052147	-0.873681
O	-4.715075	-0.941280	-2.763047
O	-3.370076	0.333473	1.241383
O	-3.602102	2.863888	-4.573453
C	-0.317820	1.090465	3.455267
H	0.413532	1.841523	3.167560
C	-1.418125	-4.589101	2.309472
C	-0.171011	-0.942352	-0.474162
C	-0.340084	1.829873	-4.889290
H	-0.883235	2.309699	-4.072625
C	-0.274587	-0.191428	2.898338
C	-0.153552	4.277577	1.581889
C	3.288381	-3.447311	-0.322761
C	1.223712	4.900291	-4.040828
H	0.443414	5.472677	-4.550452
H	1.618237	4.150340	-4.721234
H	2.031304	5.597519	-3.797661
C	-2.705519	-4.319584	1.813422
C	2.217855	-0.076800	2.271649
C	-1.389246	4.283460	2.265417
C	1.119255	1.710029	-4.469986
C	0.344639	-0.079065	0.518901
C	-1.389533	4.645366	3.612424
H	-2.332638	4.680365	4.150061
C	2.003710	1.084912	-5.351710
H	1.620963	0.689423	-6.289760
C	2.141955	-5.530261	-0.121693
C	1.634130	2.244788	-3.269274
C	-0.312934	6.576544	0.115400

H	0.680493	6.969313	0.355715
H	-0.888873	6.540842	1.038350
H	-0.785882	7.276089	-0.577037
C	0.654178	4.338075	-2.753310
C	3.870275	0.457646	3.965579
H	4.110457	0.641112	5.009714
C	2.561389	0.152809	3.606272
H	1.801746	0.086348	4.377982
C	1.045525	4.589878	2.247208
H	1.216383	-3.026007	-1.977528
C	1.095422	-6.376918	0.266210
H	1.217418	-7.428456	0.038297
C	3.368278	-6.203606	-0.689748
H	3.880603	-5.589923	-1.430913
H	3.098877	-7.163228	-1.134647
H	4.077690	-6.397516	0.123046
C	4.388244	-4.122922	1.899303
H	3.578319	-4.860495	1.890459
C	-1.158805	-4.672195	3.691538
C	0.826223	-0.616978	1.901030
C	3.227491	-0.036920	1.308322
H	2.999888	-0.256184	0.264414
C	-0.970106	0.452082	-5.129394
H	-0.514695	-0.046678	-5.993082
H	-2.040513	0.556085	-5.334920
H	-0.852271	-0.200103	-4.261897
C	3.000424	2.106305	-2.945127
C	-1.270975	1.403447	4.419336
H	-1.260925	2.390148	4.869662
C	3.293916	-2.780665	-1.563683
C	-0.160087	5.227518	-0.556912

C	4.382192	-3.394152	0.559758
C	-2.226021	-0.787158	4.209067
H	-2.973587	-1.528461	4.475693
C	4.540918	0.250908	1.670313
H	5.315012	0.258452	0.910029
C	4.424425	-2.028438	-1.893039
H	4.452557	-1.489014	-2.836254
C	-3.013513	-4.211557	0.325907
H	-2.091876	-4.410842	-0.230438
C	2.392686	4.641135	1.540771
H	2.217197	4.501838	0.469544
C	3.839758	1.467142	-3.861597
H	4.897612	1.366347	-3.640203
C	4.886026	1.982115	-1.242691
H	4.781123	0.891756	-1.250142
H	5.161072	2.293189	-0.230986
H	5.718485	2.251730	-1.903196
C	0.234573	-4.926908	4.255979
H	0.909356	-5.146469	3.420643
C	-0.027523	-6.028489	1.020793
C	-3.170114	0.749277	0.180797
C	-2.706131	3.955057	1.568510
H	-2.490421	3.193099	0.816713
C	-0.522379	2.684878	-6.151687
H	-0.116894	3.693081	-6.037368
H	-1.588731	2.778021	-6.381070
H	-0.030904	2.222627	-7.015817
C	0.040470	5.298882	-1.944383
H	-0.057674	6.284643	-2.384633
C	0.995654	4.892031	3.610824
H	1.918379	5.122015	4.138819

C	-1.250964	-1.113325	3.271262
H	-1.245096	-2.106959	2.843968
C	-3.738265	-4.138970	2.736629
H	-4.740544	-3.922651	2.374922
C	5.513671	-1.958191	-1.032813
H	6.386255	-1.371187	-1.310336
C	3.864773	4.190483	-1.773577
H	4.487919	4.399779	-2.651181
H	4.405515	4.541529	-0.886725
H	2.948406	4.780382	-1.850809
C	0.257644	-6.139233	5.195327
H	-0.345719	-5.962515	6.092360
H	1.282084	-6.342983	5.524013
H	-0.127451	-7.042720	4.709812
C	0.775333	-3.678621	4.966373
H	0.841481	-2.832415	4.276100
H	1.775948	-3.875245	5.368403
H	0.125965	-3.391446	5.801561
C	-0.923455	-7.154296	1.470472
H	-0.892530	-7.254181	2.559786
H	-0.611636	-8.097680	1.021505
H	-1.964084	-6.953876	1.202717
C	-2.225788	-4.488794	4.570728
H	-2.048367	-4.542583	5.642333
C	-0.211793	4.941776	4.287912
H	-0.239034	5.209898	5.341151
C	3.590577	2.682587	-1.660164
H	2.858677	2.529775	-0.854970
C	5.490947	-2.638566	0.176917
H	6.347217	-2.573117	0.843437
C	-3.305222	5.155770	0.820627
H	-3.417778	6.017402	1.490645
H	-4.295736	4.891486	0.436944
H	-2.698199	5.457202	-0.037075
C	4.866534	0.512926	2.996815
H	5.891680	0.745222	3.273979
C	-2.227663	0.467921	4.801568
H	-2.971712	0.722130	5.552274
C	3.354431	0.974231	-5.064218
H	4.028848	0.498090	-5.771720
C	-3.764210	3.366497	2.506437
H	-3.372250	2.529812	3.091951
H	-4.605326	2.998569	1.909961
H	-4.164934	4.121878	3.193364
C	3.295974	3.502193	2.017175
C	4.103612	-3.160542	3.058657
H	4.868171	-2.377612	3.115630
H	4.103488	-3.705873	4.010024
H	3.132937	-2.670204	2.938541
C	-3.465565	-2.793029	-0.035496
H	-4.394826	-2.525477	0.480422
H	-3.648840	-2.709072	-1.111979
H	-2.702987	-2.062189	0.243242
C	-4.062597	-5.240911	-0.113464
H	-3.757223	-6.267955	0.115894
H	-4.227897	-5.171349	-1.193338
H	-5.025190	-5.063386	0.378465
C	-3.507368	-4.229528	4.101509
H	-4.326305	-4.090051	4.802675
C	5.696363	-4.887240	2.135594
H	5.947618	-5.542244	1.293809
H	5.612862	-5.503820	3.036447

H	6.539782	-4.205357	2.288314
C	3.100795	5.989004	1.730936
H	2.467128	6.836167	1.450614
H	4.008288	6.028588	1.117905
H	3.404545	6.136384	2.773309
C	2.140340	-2.920692	-2.552175
C	2.297268	-4.176189	-3.423070
H	3.262562	-4.170541	-3.943473
H	1.506963	-4.207719	-4.180601
H	2.227619	-5.099516	-2.841507
C	1.973866	-1.698530	-3.454042
H	3.492915	3.576703	3.093044
H	4.263570	3.533330	1.501629
H	2.837606	2.528383	1.828273
H	2.772026	-1.628393	-4.200482
H	1.966734	-0.759982	-2.888632
H	1.030493	-1.769702	-4.006740
H	-1.879409	2.972985	-1.326374
C	-0.739718	-0.407705	-1.624684
H	-1.188625	-1.123968	-2.321936

44_TS7c.log

SCF (wB97x) = -3920.40750919

E(SCF)+ZPE(0 K)= -3918.827153

H(298 K)= -3918.730653

G(298 K)= -3918.956048

Lowest Frequency = -623.5359cm-1

W	-2.853772	1.290514	-2.095333
Al	-0.001836	2.299566	-0.981522
Al	0.533609	-3.141289	0.882294
O	-0.454469	-2.157946	-0.178985
O	-0.512627	0.859804	-2.076948
O	1.099957	-1.948091	1.998777
O	0.620156	1.278913	0.359082
C	-4.663444	1.550938	-1.347690
N	0.963518	3.085302	-2.472440
N	-0.450628	-4.609602	1.557867
C	-3.300002	1.902823	-3.955760
N	-0.291703	3.938194	-0.027709
N	1.927073	-4.154845	0.060864
C	-2.758625	3.294031	-1.835696
O	-5.695129	1.712280	-0.841093
O	-3.567327	2.254337	-5.030486
O	-4.162756	-1.433737	-3.170845
O	-2.953251	4.440173	-1.798769
C	0.084829	1.150915	3.680557
H	0.877609	1.849685	3.428564
C	-1.451472	-4.382176	2.577786
C	-0.204486	-0.819496	-0.289701
C	-0.068673	1.888208	-5.072858
H	-0.662589	2.422314	-4.323833
C	-0.022304	-0.068309	3.006440
C	-0.355421	4.073749	1.419782
C	3.128198	-3.454806	-0.335578
C	1.374303	4.950541	-4.055377
H	0.705070	4.734800	-4.892484
H	2.350406	4.526293	-4.293305
H	1.464872	6.033850	-3.967030
C	-2.761574	-4.028043	2.209072
C	2.437571	-0.065762	2.275969

C	-1.570643	3.963644	2.119084
C	1.372341	1.807320	-4.581221
C	0.487976	-0.028690	0.587882
C	-1.598665	4.359722	3.457351
H	-2.538527	4.299953	4.001654
C	2.295331	1.186627	-5.427501
H	1.952668	0.799515	-6.383926
C	1.863767	-5.474598	-0.124802
C	1.835276	2.320953	-3.351161
C	-0.926663	6.329225	0.000716
H	-0.339350	6.609871	0.878246
H	-1.941618	6.117895	0.346275
H	-0.958784	7.171367	-0.691095
C	0.788770	4.376480	-2.787142
C	4.186378	0.312696	3.920596
H	4.481497	0.412884	4.961985
C	2.857796	0.052266	3.605385
H	2.140042	-0.069092	4.409855
C	0.825759	4.473395	2.077918
H	1.017965	-2.815205	-1.875034
C	0.790502	-6.264231	0.312209
H	0.836115	-7.316578	0.060590
C	3.016598	-6.210789	-0.763620
H	3.535228	-5.610854	-1.511780
H	2.665162	-7.138535	-1.219059
H	3.745462	-6.472696	0.011920
C	4.275366	-4.239947	1.827975
H	3.403941	-4.903608	1.858610
C	-1.074154	-4.506680	3.929088
C	1.010491	-0.539852	1.954856
C	3.398774	0.028066	1.269529
H	3.111474	-0.111605	0.227457
C	-0.666239	0.483770	-5.244116
H	-0.069579	-0.109111	-5.946950
H	-1.681397	0.550754	-5.645648
H	-0.702594	-0.047635	-4.291590
C	3.189495	2.178581	-2.974999
C	-0.791857	1.465759	4.715756
H	-0.656259	2.395895	5.257729
C	3.124996	-2.745092	-1.552004
C	-0.367093	5.107598	-0.686216
C	4.254758	-3.483417	0.505226
C	-1.999436	-0.568836	4.327858
H	-2.818513	-1.247070	4.550128
C	4.736270	0.258902	1.586493
H	5.475556	0.297419	0.793411
C	4.281189	-2.043945	-1.903508
H	4.303042	-1.473941	-2.828551
C	-3.188464	-3.815844	0.762815
H	-2.317960	-3.977615	0.120267
C	2.173424	4.573711	1.372627
H	2.053864	4.185508	0.357000
C	4.064319	1.539904	-3.856608
H	5.110236	1.430645	-3.589498
C	5.038298	2.083289	-1.228737
H	4.963064	0.990897	-1.256173
H	5.274616	2.384483	-0.204315
H	5.879921	2.387082	-1.862133
C	0.356383	-4.805239	4.362352
H	0.953099	-5.009674	3.466014
C	-0.254724	-5.871005	1.153659
C	-3.625611	-0.502554	-2.750262

C	-2.844099	3.424366	1.490912
H	-2.625653	3.201874	0.448920
C	-0.196350	2.628721	-6.413874
H	0.307627	3.599012	-6.420345
H	-1.254772	2.790469	-6.641821
H	0.232271	2.037473	-7.231095
C	0.075151	5.284725	-1.998089
H	-0.066035	6.271047	-2.421155
C	0.749324	4.837638	3.422943
H	1.649356	5.168532	3.936579
C	-1.095686	-0.902533	3.323918
H	-1.222206	-1.842789	2.803511
C	-3.702439	-3.847025	3.226210
H	-4.722641	-3.579426	2.961298
C	5.403782	-2.058469	-1.084723
H	6.295783	-1.509006	-1.377244
C	3.974882	4.267877	-1.762096
H	4.615573	4.498418	-2.621539
H	4.487915	4.620175	-0.860135
H	3.050920	4.844126	-1.856032
C	0.438996	-6.044354	5.262007
H	-0.089301	-5.888085	6.208891
H	1.483722	-6.268153	5.501947
H	0.005777	-6.929134	4.782599
C	0.973399	-3.583304	5.056262
H	0.984307	-2.719639	4.384432
H	2.004803	-3.798190	5.358390
H	0.405916	-3.315833	5.955147
C	-1.169778	-6.957944	1.658297
H	-1.039473	-7.093485	2.736511
H	-0.958273	-7.903131	1.157499
H	-2.217685	-6.691219	1.499817
C	-2.050442	-4.311680	4.905744
H	-1.778755	-4.400539	5.954991
C	-0.459979	4.815011	4.104036
H	-0.508944	5.132577	5.142949
C	3.735020	2.752695	-1.669879
H	2.994946	2.566698	-0.879273
C	5.387153	-2.773132	0.104334
H	6.266635	-2.769392	0.743384
C	-4.006563	4.422367	1.522666
H	-4.307095	4.657006	2.550529
H	-4.875143	3.992246	1.012358
H	-3.761366	5.363908	1.020739
C	5.134137	0.422085	2.908435
H	6.176986	0.614370	3.147745
C	-1.831822	0.606314	5.050537
H	-2.512611	0.857474	5.860157
C	3.628722	1.055792	-5.081458
H	4.328623	0.576280	-5.761057
C	-3.259168	2.100681	2.142540
H	-2.454259	1.360767	2.093440
H	-4.131652	1.689263	1.622832
H	-3.523070	2.239479	3.197357
C	3.236112	3.716878	2.069876
C	4.148434	-3.274140	3.011817
H	4.973913	-2.552898	3.020294
H	4.171586	-3.828684	3.957768
H	3.212998	-2.709835	2.958223
C	-3.654661	-2.371950	0.537188
H	-4.549680	-2.143966	1.127904
H	-3.905434	-2.219051	-0.517682

H	-2.874808	-1.652778	0.803324
C	-4.282183	-4.805223	0.341131
H	-3.963251	-5.847132	0.455804
H	-4.546590	-4.648919	-0.709741
H	-5.192861	-4.669424	0.935316
C	-3.358898	-3.998695	4.561801
H	-4.107796	-3.857822	5.337026
C	5.524520	-5.118050	1.971260
H	5.664796	-5.778686	1.108343
H	5.446011	-5.739601	2.869253
H	6.431496	-4.512790	2.074931
C	2.638974	6.031455	1.255643
H	1.941833	6.633783	0.663520
H	3.618985	6.084715	0.769016
H	2.731906	6.496764	2.243539
C	1.923300	-2.779910	-2.489687
C	1.934025	-4.037262	-3.372029
H	2.872589	-4.107727	-3.934801
H	1.111133	-3.996281	-4.093605
H	1.812678	-4.955944	-2.791986
C	1.822500	-1.542242	-3.378537
H	3.436932	4.070483	3.087558
H	4.182422	3.761827	1.517074
H	2.928852	2.668196	2.126520
H	2.607516	-1.519208	-4.142226
H	1.884917	-0.610224	-2.806229
H	0.866049	-1.548831	-3.910061
H	-2.217427	0.608117	-0.482122
C	-0.840066	-0.259316	-1.456683
H	-1.279940	-1.026689	-2.095657

45_Int8c.log

SCF (wB97x) = -3920.48900748

E(SCF)+ZPE(0 K)= -3918.902422

H(298 K)= -3918.805591

G(298 K)= -3919.033640

Lowest Frequency = 16.6080cm-1

W	-3.147444	1.474940	-2.192203
Al	0.083538	2.235007	-0.719923
Al	0.639149	-3.181551	0.949190
O	-0.227406	-2.176365	-0.195660
O	-1.144601	1.062903	-1.365896
O	1.198286	-2.030393	2.108348
O	0.949194	1.230700	0.423454
C	-4.986374	1.562335	-1.518077
N	0.916772	2.955731	-2.307778
N	-0.461866	-4.583634	1.594548
C	-3.830900	1.853228	-3.981045
N	-0.435540	3.858908	0.069325
N	1.994666	-4.289775	0.178853
C	-3.159079	3.500990	-1.984652
O	-6.049474	1.610700	-1.044799
O	-4.158652	2.069507	-5.080316
O	-3.879648	-1.536430	-3.000231
O	-3.358350	4.642440	-1.952433
C	0.359538	1.182118	3.696016
H	1.150018	1.845984	3.359226
C	-1.446515	-4.312075	2.618934
C	0.029970	-0.837517	-0.239285
C	-0.237425	1.641721	-4.721270

H	-0.791095	2.000905	-3.847468
C	0.218271	-0.084898	3.123702
C	-0.529139	4.057533	1.504039
C	3.256529	-3.679608	-0.176754
C	1.303475	4.812257	-3.929881
H	0.585880	5.463467	-4.433967
H	1.685914	4.080715	-4.637153
H	2.142616	5.438505	-3.608499
C	-2.707605	-3.798613	2.270395
C	2.643432	-0.206119	2.338490
C	-1.753971	3.880204	2.170060
C	1.245556	1.609128	-4.358479
C	0.705934	-0.103118	0.667643
C	-1.848051	4.308133	3.493250
H	-2.792471	4.203640	4.020242
C	2.137157	1.020272	-5.258391
H	1.753918	0.601927	-6.185811
C	1.840190	-5.600131	-0.017036
C	1.774760	2.174611	-3.178935
C	-1.241435	6.189591	-0.044286
H	-0.636450	6.591638	0.773498
H	-2.213463	5.916462	0.376270
H	-1.388555	6.965565	-0.795631
C	0.662913	4.211268	-2.696668
C	4.437950	0.140452	3.940086
H	4.758687	0.246286	4.973502
C	3.094014	-0.077059	3.656287
H	2.386686	-0.154158	4.475901
C	0.608189	4.549334	2.169048
H	1.267171	-2.873931	-1.816621
C	0.684757	-6.305860	0.351201
H	0.655736	-7.353543	0.078420
C	2.967625	-6.421772	-0.594056
H	3.560518	-5.869643	-1.323664
H	2.576330	-7.330809	-1.054517
H	3.639751	-6.719198	0.219069
C	4.257660	-4.477613	2.054091
H	3.324921	-5.052827	2.079899
C	-1.098295	-4.554532	3.962619
C	1.187873	-0.615665	2.044674
C	3.585849	-0.152045	1.311444
H	3.264779	-0.287156	0.279282
C	-0.772353	0.245886	-5.074310
H	-0.297867	-0.146128	-5.980806
H	-1.848470	0.300308	-5.267246
H	-0.608399	-0.477124	-4.270394
C	3.145948	2.079000	-2.866740
C	-0.465315	1.584772	4.742237
H	-0.299727	2.552149	5.203543
C	3.356304	-3.003782	-1.409516
C	-0.587699	4.979920	-0.662609
C	4.341311	-3.760059	0.713135
C	-1.674202	-0.480028	4.597145
H	-2.467336	-1.145487	4.928332
C	4.936088	0.042933	1.595061
H	5.657793	0.055596	0.783609
C	4.574244	-2.397215	-1.724937
H	4.679554	-1.861577	-2.664070
C	-3.087522	-3.432671	0.843147
H	-2.239230	-3.656562	0.190520
C	1.966748	4.682635	1.489219
H	1.845396	4.426757	0.431983

C	3.988605	1.470325	-3.799569
H	5.051524	1.396590	-3.593382
C	5.049534	1.988175	-1.183799
H	4.996637	0.895478	-1.241138
H	5.306899	2.260113	-0.156057
H	5.866470	2.328647	-1.831176
C	0.293652	-5.010819	4.382869
H	0.865024	-5.254950	3.480308
C	-0.360840	-5.844835	1.157222
C	-3.536230	-0.489434	-2.653993
C	-2.932433	3.213751	1.482873
H	-2.799147	3.348828	0.406981
C	-0.544028	2.593744	-5.886715
H	-0.266158	3.627456	-5.668645
H	-1.617154	2.575416	-6.103586
H	-0.009179	2.285440	-6.792819
C	-0.137838	5.108856	-1.973696
H	-0.332141	6.059680	-2.454102
C	0.469516	4.945845	3.501168
H	1.331688	5.344538	4.031389
C	-0.820468	-0.897023	3.579525
H	-0.949680	-1.885689	3.160358
C	-3.634821	-3.581502	3.293086
H	-4.617827	-3.190478	3.041323
C	5.656548	-2.462820	-0.856073
H	6.596078	-1.983829	-1.121430
C	3.910732	4.163316	-1.616619
H	4.520995	4.445378	-2.482905
H	4.432179	4.504343	-0.714907
H	2.964074	4.707089	-1.668580
C	0.254207	-6.270965	5.255269
H	-0.245287	-6.082882	6.211888
H	1.272100	-6.607469	5.478263
H	-0.273233	-7.094584	4.761396
C	1.029417	-3.869743	5.098801
H	1.126763	-2.997084	4.444905
H	2.033367	-4.191646	5.398058
H	0.488824	-3.564394	6.002268
C	-1.390123	-6.859578	1.587651
H	-1.306429	-7.057420	2.660874
H	-1.256046	-7.797003	1.046931
H	-2.401875	-6.483018	1.414610
C	-2.057157	-4.315328	4.946458
H	-1.805537	-4.492367	5.989478
C	-0.755496	4.862541	4.147734
H	-0.853194	5.205037	5.175133
C	3.718844	2.639877	-1.566710
H	3.009059	2.405378	-0.761180
C	5.536724	-3.138167	0.348673
H	6.383795	-3.174454	1.029200
C	-4.291063	3.818547	1.842324
H	-4.568509	3.611213	2.881966
H	-5.067511	3.381419	1.205471
H	-4.303579	4.904419	1.697171
C	5.367564	0.204749	2.907252
H	6.420851	0.366559	3.122898
C	-1.486988	0.758571	5.199097
H	-2.125992	1.076902	6.019269
C	3.496806	0.965730	-4.995768
H	4.174406	0.514199	-5.715772
C	-2.916896	1.704034	1.764783
H	-1.972726	1.237848	1.459279

H	-3.733467	1.206364	1.226594
H	-3.037162	1.505962	2.835982
C	2.979341	3.693000	2.080993
C	4.201830	-3.466866	3.205064
H	5.084541	-2.816784	3.200230
H	4.169180	-3.988642	4.168918
H	3.317387	-2.829414	3.120681
C	-3.350932	-1.925199	0.734566
H	-4.208241	-1.623015	1.348111
H	-3.574055	-1.657618	-0.302890
H	-2.480191	-1.345463	1.056685
C	-4.296076	-4.232721	0.343744
H	-4.115942	-5.312910	0.384691
H	-4.518665	-3.966998	-0.694989
H	-5.191442	-4.021833	0.939384
C	-3.322850	-3.847158	4.618024
H	-4.060382	-3.674805	5.397834
C	5.414072	-5.465953	2.250841
H	5.508895	-6.162928	1.410669
H	5.258664	-6.049859	3.164001
H	6.372146	-4.945857	2.357597
C	2.498153	6.119816	1.543334
H	1.786205	6.830111	1.108638
H	3.436351	6.198070	0.982824
H	2.701688	6.436579	2.572125
C	2.192560	-2.964390	-2.395445
C	2.099364	-4.252918	-3.226621
H	3.048443	-4.455179	-3.737765
H	1.321260	-4.149658	-3.990447
H	1.842825	-5.125080	-2.620228
C	2.258562	-1.765976	-3.340477
H	3.105664	3.847899	3.159006
H	3.962150	3.824692	1.611777
H	2.664344	2.657312	1.914861
H	3.066569	-1.864940	-4.074594
H	2.412823	-0.823629	-2.803027
H	1.325867	-1.689647	-3.909131
H	-1.242929	-0.937975	-1.906089
C	-0.508176	-0.233729	-1.514165
H	0.292167	-0.127672	-2.262368

46_TS10.log

SCF (wB97x) = -3920.43472476

E(SCF)+ZPE(0 K)= -3918.858733

H(298 K)= -3918.761006

G(298 K)= -3918.990921

Lowest Frequency = -221.7659cm-1

W	-2.454790	-0.738715	-2.730029
Al	-0.162384	2.934898	-0.489548
Al	0.451631	-2.605100	0.917278
O	-0.185247	-1.567967	-0.327136
O	-1.089451	1.711958	-1.370011
O	1.337813	-1.468839	1.869834
O	0.757228	1.841782	0.542608
C	-3.531495	-1.563993	-4.226067
N	0.914113	4.122708	-1.506069
N	-0.670238	-3.489954	2.174137
C	-4.066867	-0.910995	-1.572969
N	-1.298758	4.261129	0.265036
N	1.189829	-4.174046	0.142454

C	-1.926376	-2.601215	-2.048078
O	-4.151560	-2.020846	-5.095071
O	-5.031602	-1.066933	-0.940525
O	-3.440412	2.189640	-3.601125
O	-1.725655	-3.684688	-1.695152
C	2.981283	1.075328	3.336189
H	3.887897	1.059794	2.738586
C	-1.058148	-2.822710	3.398303
C	-0.173178	-0.205119	-0.317238
C	1.231714	3.029769	-4.227025
H	0.328374	3.465596	-3.786986
C	1.789580	0.590412	2.804788
C	-1.969332	3.929965	1.503189
C	2.524239	-4.215004	-0.416360
C	1.306914	6.204097	-2.780232
H	2.340581	6.285662	-2.433528
H	0.878815	7.203456	-2.860815
H	1.338465	5.755435	-3.776400
C	-2.282850	-2.140588	3.469244
C	3.071743	-0.274919	0.759029
C	-3.281797	3.430285	1.489933
C	2.405931	3.313472	-3.301345
C	0.675404	0.501150	0.514328
C	-3.818724	2.994474	2.703528
H	-4.826954	2.586494	2.720495
C	3.706656	3.097838	-3.763235
H	3.859672	2.753439	-4.782819
C	0.436038	-5.283703	0.068756
C	2.235489	3.761651	-1.980517
C	-2.506509	6.417176	0.252660
H	-3.407295	6.319458	-0.363824

H	-2.158510	7.448914	0.175598
H	-2.783737	6.202632	1.284916
C	0.487568	5.355736	-1.838241
C	5.209210	-1.419863	0.640616
H	5.889935	-2.171673	1.032597
C	3.978491	-1.221595	1.250172
H	3.689253	-1.809685	2.116700
C	-1.252010	4.117457	2.700013
H	0.760513	-3.156905	-2.053868
C	-0.685209	-5.511641	0.871900
H	-1.212519	-6.443294	0.708551
C	0.775151	-6.352864	-0.939479
H	1.849849	-6.506323	-1.046185
H	0.387864	-6.025462	-1.911694
H	0.295860	-7.298790	-0.683057
C	3.305985	-5.337155	1.764411
H	2.332857	-4.970680	2.105157
C	-0.180143	-2.857489	4.499352
C	1.705934	-0.172987	1.468807
C	3.438696	0.480109	-0.352472
H	2.764097	1.230034	-0.754565
C	1.007633	1.517914	-4.320809
H	1.886011	1.018244	-4.746485
H	0.145113	1.299982	-4.960643
H	0.822993	1.085027	-3.332545
C	3.334245	3.938768	-1.114114
C	3.035093	1.545284	4.650908
H	3.979701	1.903488	5.053091
C	2.786615	-3.719815	-1.704303
C	-1.457204	5.470496	-0.272119
C	3.553373	-4.781268	0.366800

C	0.691000	1.080079	4.910883
H	-0.211724	1.063022	5.517272
C	4.670372	0.272076	-0.976786
H	4.921985	0.857374	-1.856538
C	4.093332	-3.800651	-2.192955
H	4.308459	-3.418766	-3.188334
C	-3.179491	-1.935151	2.259782
H	-2.795057	-2.535542	1.427412
C	0.047664	4.914408	2.734146
H	0.409178	5.025415	1.706261
C	4.612971	3.733231	-1.631616
H	5.477514	3.872239	-0.989997
C	4.284134	3.830349	1.245334
H	4.492287	2.771891	1.059173
H	3.996231	3.938366	2.296058
H	5.210347	4.398069	1.097155
C	1.187434	-3.519042	4.422086
H	1.471889	-3.558127	3.367893
C	-1.131522	-4.720322	1.942181
C	-3.059851	1.141800	-3.299991
C	-4.147824	3.437855	0.236392
H	-3.568367	3.888244	-0.576453
C	1.407374	3.625671	-5.628913
H	1.638584	4.695902	-5.602576
H	0.487878	3.492983	-6.207526
H	2.212776	3.128247	-6.179857
C	-0.671765	5.943397	-1.331949
H	-0.918204	6.929264	-1.706094
C	-1.831293	3.663139	3.884497
H	-1.290953	3.777933	4.820994
C	0.640143	0.614458	3.604565
H	-0.295676	0.220457	3.213288
C	-2.647567	-1.554659	4.684672
H	-3.595128	-1.025287	4.753257
C	5.110122	-4.373495	-1.446871
H	6.116386	-4.440591	-1.852435
C	2.992679	5.857236	0.512912
H	3.864697	6.382232	0.104981
H	2.913659	6.115983	1.575248
H	2.097157	6.241688	0.014193
C	1.132422	-4.957556	4.951212
H	0.795577	-4.976655	5.994374
H	2.122187	-5.427140	4.909538
H	0.445744	-5.577335	4.363158
C	2.271908	-2.701532	5.130216
H	2.276258	-1.667592	4.769289
H	3.255931	-3.140765	4.932769
H	2.137668	-2.689445	6.217812
C	-2.168428	-5.319997	2.857133
H	-1.857958	-5.242149	3.902671
H	-2.341885	-6.367795	2.610007
H	-3.112480	-4.774850	2.764266
C	-0.593466	-2.269332	5.693859
H	0.065658	-2.297435	6.557116
C	-3.092676	3.079981	3.884125
H	-3.525349	2.721338	4.814544
C	3.152451	4.339639	0.347067
H	2.237307	3.854843	0.706029
C	4.834556	-4.862826	-0.177143
H	5.633753	-5.314703	0.405667
C	-5.407332	4.289267	0.451907
H	-6.073997	3.824924	1.186838

H	-5.965355	4.379901	-0.485873
H	-5.171606	5.296311	0.811681
C	5.556537	-0.677562	-0.486375
H	6.510746	-0.844297	-0.979769
C	1.896266	1.536682	5.445736
H	1.943155	1.885928	6.474389
C	4.802925	3.331998	-2.948375
H	5.809358	3.181078	-3.330480
C	-4.529268	2.033350	-0.223302
H	-3.636257	1.438414	-0.426779
H	-5.118362	2.080474	-1.145417
H	-5.128026	1.511015	0.531099
C	1.160752	4.243541	3.533388
C	4.359684	-4.860517	2.770303
H	5.351313	-5.267675	2.544339
H	4.097479	-5.194040	3.780360
H	4.437943	-3.768596	2.782458
C	-3.113154	-0.464428	1.838241
H	-3.450179	0.190131	2.650774
H	-3.755690	-0.295043	0.974358
H	-2.093107	-0.166455	1.571386
C	-4.637166	-2.343598	2.502037
H	-4.729258	-3.380871	2.842103
H	-5.207204	-2.234450	1.573940
H	-5.110313	-1.706810	3.258248
C	-1.823480	-1.632653	5.796105
H	-2.129194	-1.183213	6.737745
C	3.240272	-6.870408	1.756407
H	2.433230	-7.239889	1.116266
H	3.064246	-7.250697	2.768933
H	4.181441	-7.298883	1.392342
C	-0.226231	6.327170	3.271488
H	-0.987559	6.850511	2.683378
H	0.690179	6.927554	3.249375
H	-0.577375	6.285370	4.308856
C	1.705666	-3.138342	-2.600718
C	1.531273	-3.967043	-3.880076
H	2.418366	-3.904964	-4.521364
H	0.676068	-3.596094	-4.456538
H	1.356294	-5.025317	-3.660803
C	2.002777	-1.677085	-2.945616
H	0.892317	4.120774	4.587351
H	2.066768	4.860742	3.500287
H	1.398511	3.256596	3.129725
H	2.977216	-1.567492	-3.437021
H	2.007218	-1.053501	-2.047444
H	1.240008	-1.290558	-3.627951
H	-0.905434	-0.313239	-3.830777
C	-1.101804	0.391663	-1.290556
H	-1.021239	-1.101539	-3.982711

47_Int1prima.log

SCF (wB97x) = -3920.43497524
E(SCF)+ZPE(0 K)= -3918.858619
H(298 K)= -3918.760312
G(298 K)= -3918.991716
Lowest Frequency = 14.0235cm-1

W	-2.463967	-0.716267	-2.765334
Al	-0.168323	2.952876	-0.529373
Al	0.441541	-2.586134	0.880587

O	-0.194085	-1.548515	-0.363771
O	-1.092128	1.730613	-1.413221
O	1.324137	-1.449680	1.836218
O	0.750353	1.860700	0.504669
C	-3.490006	-1.566966	-4.283104
N	0.905324	4.139916	-1.548506
N	-0.680744	-3.475897	2.133950
C	-4.092748	-0.896539	-1.632485
N	-1.302779	4.278406	0.228080
N	1.182778	-4.151756	0.102401
C	-1.942273	-2.577931	-2.073408
O	-4.076049	-2.043632	-5.164836
O	-5.065777	-1.052172	-1.012429
O	-3.480321	2.209861	-3.605965
O	-1.744329	-3.660763	-1.718181
C	2.963134	1.088724	3.311161
H	3.873742	1.069292	2.719758
C	-1.074215	-2.809198	3.356717
C	-0.181382	-0.185465	-0.354342
C	1.203943	3.053144	-4.274730
H	0.305894	3.494815	-3.829699
C	1.772593	0.609943	2.771459
C	-1.967226	3.946163	1.469274
C	2.519058	-4.189885	-0.451836
C	1.298847	6.221839	-2.820601
H	2.335034	6.296220	-2.479988
H	0.875774	7.223845	-2.894731
H	1.321552	5.776912	-3.818695
C	-2.302783	-2.133606	3.424394
C	3.062283	-0.257943	0.730845
C	-3.277928	3.441823	1.462658
C	2.383441	3.322376	-3.351134
C	0.666843	0.520214	0.477867
C	-3.806645	3.002702	2.678742
H	-4.813449	2.591399	2.700657
C	3.680617	3.094663	-3.817391
H	3.826870	2.748125	-4.837254
C	0.432269	-5.263576	0.026659
C	2.222214	3.772352	-2.029701
C	-2.510911	6.433926	0.219244
H	-3.412217	6.335967	-0.396447
H	-2.163908	7.466060	0.142953
H	-2.786838	6.217941	1.251566
C	0.480422	5.374042	-1.877442
C	5.201040	-1.402091	0.626050
H	5.881297	-2.150726	1.024898
C	3.968460	-1.200807	1.230634
H	3.677156	-1.783647	2.100037
C	-1.244073	4.134875	2.662500
H	0.762509	-3.122711	-2.095688
C	-0.688512	-5.495990	0.828964
H	-1.212960	-6.429020	0.664214
C	0.775311	-6.330374	-0.982832
H	1.850546	-6.480650	-1.088689
H	0.388134	-6.002922	-1.955065
H	0.298518	-7.278044	-0.728179
C	3.292874	-5.316501	1.729773
H	2.317925	-4.951813	2.067254
C	-0.197628	-2.836986	4.459053
C	1.694092	-0.154036	1.435560
C	3.431723	0.489870	-0.384591
H	2.757796	1.236752	-0.793597

C	0.964011	1.544408	-4.378892
H	1.838189	1.038835	-4.806385
H	0.101268	1.341280	-5.023323
H	0.768595	1.105278	-3.395931
C	3.325807	3.941800	-1.167736
C	3.010236	1.557958	4.626389
H	3.953836	1.911210	5.035237
C	2.786546	-3.690974	-1.737290
C	-1.461716	5.488410	-0.307627
C	3.545225	-4.758321	0.333886
C	0.661760	1.105942	4.869566
H	-0.245507	1.094469	5.469302
C	4.665322	0.278460	-1.004142
H	4.918876	0.858016	-1.887131
C	4.095138	-3.772165	-2.221162
H	4.314415	-3.388472	-3.214925
C	-3.200660	-1.940079	2.213958
H	-2.815122	-2.546465	1.386489
C	0.053994	4.934774	2.690130
H	0.411311	5.044764	1.660640
C	4.600673	3.725218	-1.690059
H	5.468946	3.857904	-1.052133
C	4.284986	3.832892	1.188675
H	4.486081	2.772575	1.005466
H	4.002035	3.946358	2.240193
H	5.213957	4.394588	1.034835
C	1.171637	-3.495626	4.386742
H	1.459261	-3.536369	3.333457
C	-1.137944	-4.707344	1.900041
C	-3.091295	1.161865	-3.316965
C	-4.151665	3.449085	0.214523
H	-3.575471	3.894769	-0.603324
C	1.382213	3.656325	-5.673383
H	1.626408	4.723501	-5.641215
H	0.459533	3.538114	-6.250085
H	2.180028	3.152910	-6.229915
C	-0.677115	5.962542	-1.367512
H	-0.923097	6.949280	-1.739700
C	-1.815403	3.677548	3.849631
H	-1.269834	3.792198	4.783114
C	0.617623	0.640541	3.562921
H	-0.317848	0.252606	3.164581
C	-2.671042	-1.544145	4.636987
H	-3.621209	-1.019127	4.702814
C	5.108724	-4.347917	-1.473100
H	6.116254	-4.415137	-1.875471
C	3.001478	5.864964	0.457524
H	3.874237	6.384798	0.044601
H	2.928804	6.125831	1.519786
H	2.105572	6.253220	-0.037468
C	1.117443	-4.933167	4.918698
H	0.777281	-4.950602	5.960809
H	2.108103	-5.401169	4.881213
H	0.433646	-5.555341	4.329818
C	2.252680	-2.675039	5.096488
H	2.255723	-1.641455	4.734549
H	3.238082	-3.112465	4.901808
H	2.115966	-2.662167	6.183769
C	-2.174196	-5.311320	2.812906
H	-1.868545	-5.228370	3.859427
H	-2.339805	-6.360919	2.568031
H	-3.121559	-4.772864	2.714433

C	-0.614715	-2.245898	5.650827
H	0.043450	-2.268163	6.514967
C	-3.074553	3.089528	3.855463
H	-3.500729	2.728054	4.787789
C	3.152580	4.346249	0.293624
H	2.236323	3.867139	0.657281
C	4.828286	-4.839688	-0.205421
H	5.624996	-5.293383	0.379381
C	-5.406061	4.306538	0.436716
H	-6.069113	3.846529	1.177656
H	-5.970441	4.397166	-0.497266
H	-5.163890	5.313461	0.792402
C	5.550889	-0.667066	-0.504981
H	6.506542	-0.836387	-0.994688
C	1.865769	1.555483	5.413141
H	1.907290	1.904324	6.442168
C	4.781910	3.320024	-3.006908
H	5.785514	3.159780	-3.392675
C	-4.543617	2.045225	-0.238520
H	-3.656017	1.444341	-0.448286
H	-5.140459	2.093669	-1.155549
H	-5.138979	1.527875	0.522002
C	1.171775	4.267845	3.486159
C	4.342010	-4.839496	2.740268
H	5.335204	-5.244480	2.517276
H	4.076715	-5.174966	3.748863
H	4.418152	-3.747480	2.754228
C	-3.139018	-0.473095	1.779460
H	-3.477762	0.187454	2.586556
H	-3.782620	-0.313519	0.914434
H	-2.120025	-0.174262	1.509551
C	-4.657074	-2.350610	2.460312
H	-4.746207	-3.385107	2.809539
H	-5.228102	-2.250963	1.531680
H	-5.131929	-1.708537	3.210917
C	-1.847225	-1.613518	5.749209
H	-2.155502	-1.161261	6.688660
C	3.229472	-6.849834	1.719770
H	2.425612	-7.219861	1.075961
H	3.049911	-7.231441	2.731170
H	4.172752	-7.276542	1.359116
C	-0.220743	6.347817	3.226311
H	-0.985130	6.868888	2.640189
H	0.694431	6.949909	3.200163
H	-0.568202	6.306714	4.264950
C	1.710192	-3.107817	-2.638885
C	1.539875	-3.939188	-3.917238
H	2.429376	-3.878662	-4.555316
H	0.686857	-3.569277	-4.497500
H	1.364127	-4.996933	-3.696169
C	2.014481	-1.648195	-2.986157
H	0.908795	4.148503	4.541872
H	2.076874	4.886007	3.446335
H	1.408583	3.279772	3.084710
H	3.002833	-1.539855	-3.448885
H	1.990624	-1.017548	-2.093288
H	1.274344	-1.266733	-3.696548
H	-1.097088	-0.306381	-4.080789
C	-1.108293	0.410415	-1.329934
H	-0.842616	-1.020572	-3.785244

48_TS11.log

SCF (wB97x) = -3920.42527802

E(SCF)+ZPE(0 K)= -3918.847533

H(298 K)= -3918.750599

G(298 K)= -3918.977922

Lowest Frequency = -23.9830cm-1

W	-2.489624	-0.569049	-2.935212
Al	-0.134956	2.879970	-0.541731
Al	0.452137	-2.684240	0.725842
O	-0.244667	-1.636765	-0.491696
O	-1.102713	1.707940	-1.438082
O	1.356938	-1.573544	1.688067
O	0.772470	1.746693	0.438448
C	-3.570671	-1.186637	-4.524187
N	0.922460	4.024725	-1.621310
N	-0.703387	-3.590459	1.960226
C	-3.326257	-1.947787	-1.766337
N	-1.173812	4.226813	0.313412
N	1.292930	-4.245605	0.033611
C	-1.229589	-2.080427	-3.488280
O	-4.183390	-1.540645	-5.444798
O	-3.815958	-2.788125	-1.129500
O	-4.986759	1.410203	-2.640293
O	-0.685433	-3.021598	-3.882790
C	2.980541	0.893253	3.232798
H	3.906303	0.829549	2.668457
C	-1.177819	-2.917078	3.154830
C	-0.207411	-0.267749	-0.454954
C	1.189478	2.645160	-4.258467
H	0.260253	2.896480	-3.733333
C	1.782332	0.482131	2.653778
C	-1.717476	3.961946	1.631591
C	2.682646	-4.314705	-0.376140
C	1.215181	6.065059	-2.984027
H	2.244331	6.236912	-2.655228
H	0.725537	7.027610	-3.132834
H	1.273274	5.543593	-3.941951
C	-2.454701	-2.331707	3.172239
C	3.073481	-0.327318	0.592635
C	-3.026151	3.473549	1.784161
C	2.366849	3.146353	-3.433289
C	0.667500	0.410340	0.378080
C	-3.440673	3.108085	3.067398
H	-4.443902	2.711770	3.205880
C	3.653380	3.021008	-3.966450
H	3.774142	2.625813	-4.972082
C	0.551035	-5.362228	-0.095689
C	2.232925	3.676375	-2.139631
C	-2.334212	6.409425	0.327713
H	-3.288386	6.311937	-0.202195
H	-1.973358	7.429332	0.182868
H	-2.520370	6.236444	1.387850
C	0.463749	5.241577	-1.966887
C	5.238992	-1.411684	0.434564
H	5.932148	-2.168408	0.792318
C	3.994244	-1.282845	1.035905
H	3.708852	-1.930780	1.859463
C	-0.889280	4.206114	2.743907
H	1.088919	-3.560381	-2.323864
C	-0.624827	-5.590753	0.620452
H	-1.134382	-6.526259	0.424952

C	0.999561	-6.440333	-1.051344
H	2.040369	-6.725905	-0.884309
H	0.931799	-6.056532	-2.075413
H	0.366394	-7.324403	-0.967086
C	3.241254	-5.333865	1.925165
H	2.255056	-4.926454	2.164641
C	-0.336007	-2.843479	4.283430
C	1.708539	-0.269739	1.309689
C	3.433516	0.496230	-0.472278
H	2.746597	1.252495	-0.837753
C	1.281772	1.116584	-4.352089
H	2.253212	0.806904	-4.753289
H	0.510150	0.713287	-5.016598
H	1.166060	0.650545	-3.368180
C	3.365830	3.959523	-1.347193
C	3.011454	1.356368	4.550171
H	3.959528	1.655742	4.990409
C	3.082830	-3.903643	-1.657527
C	-1.357068	5.420110	-0.254034
C	3.624712	-4.841993	0.535128
C	0.633323	1.045525	4.713308
H	-0.293023	1.092158	5.281022
C	4.676372	0.355963	-1.091547
H	4.922933	0.997201	-1.933286
C	4.435267	-3.991906	-1.998017
H	4.752888	-3.658899	-2.983007
C	-3.370509	-2.306305	1.962563
H	-2.951157	-2.940579	1.177400
C	0.427245	4.961413	2.621639
H	0.694303	5.018480	1.561452
C	4.625579	3.831372	-1.930311
H	5.510216	4.062202	-1.344680
C	4.414275	3.867897	0.963135
H	4.594411	2.802786	0.785326
H	4.185750	3.998712	2.024937
H	5.341141	4.416095	0.758816
C	1.043552	-3.480629	4.306621
H	1.420741	-3.478738	3.281737
C	-1.125856	-4.824229	1.693596
C	-4.050338	0.730888	-2.647144
C	-4.035319	3.427475	0.645698
H	-3.537075	3.751296	-0.275249
C	1.125284	3.259426	-5.662179
H	1.091362	4.353512	-5.640848
H	0.227683	2.908714	-6.181678
H	1.989236	2.966208	-6.268279
C	-0.664091	5.839930	-1.399236
H	-0.941638	6.810564	-1.791252
C	-1.355069	3.836452	4.005178
H	-0.725207	4.004017	4.875040
C	0.606192	0.577829	3.406532
H	-0.337252	0.245269	2.975794
C	-2.883061	-1.694931	4.340398
H	-3.869660	-1.238152	4.359929
C	5.367536	-4.519094	-1.121527
H	6.411722	-4.600046	-1.412806
C	3.102469	5.901933	0.260100
H	3.957119	6.413829	-0.198000
H	3.072803	6.183697	1.318948
H	2.186773	6.279859	-0.207469
C	0.939570	-4.933595	4.790266
H	0.486133	-4.975388	5.787563

H	1.928065	-5.401785	4.852764	H	-5.016220	1.603834	1.314301
H	0.325930	-5.541711	4.115537	C	1.580173	4.274071	3.349118
C	2.055870	-2.687991	5.138227	C	4.225705	-4.847714	2.995087
H	2.078188	-1.637111	4.831295	H	5.216638	-5.296625	2.868810
H	3.057766	-3.106336	4.998861	H	3.873981	-5.128934	3.992766
H	1.837308	-2.733596	6.211316	H	4.343453	-3.759283	2.969902
C	-2.179400	-5.476015	2.552538	C	-3.433948	-0.881794	1.412038
H	-2.029664	-5.247853	3.610099	H	-3.759916	-0.180370	2.189066
H	-2.174359	-6.557309	2.409847	H	-4.140980	-0.824279	0.580414
H	-3.167406	-5.096474	2.272923	H	-2.454864	-0.550788	1.047724
C	-0.812508	-2.205061	5.427710	C	-4.785622	-2.811732	2.269204
H	-0.176121	-2.143208	6.305260	H	-4.781466	-3.783126	2.775444
C	-2.608897	3.262988	4.166283	H	-5.346697	-2.913052	1.334941
H	-2.950659	2.967306	5.154781	H	-5.332400	-2.111486	2.911096
C	3.246379	4.381329	0.112955	C	-2.077487	-1.635162	5.464750
H	2.342599	3.908400	0.514484	H	-2.429633	-1.142541	6.367735
C	4.955342	-4.950243	0.131698	C	3.127313	-6.863806	1.973347
H	5.686369	-5.376031	0.814287	H	2.348754	-7.235848	1.300336
C	-5.203741	4.385469	0.927293	H	2.875498	-7.195869	2.986939
H	-5.802741	4.027684	1.772133	H	4.075253	-7.334915	1.687996
H	-5.863866	4.441549	0.055486	C	0.250923	6.402806	3.121665
H	-4.866980	5.397657	1.171496	H	-0.545157	6.928370	2.583402
C	5.582682	-0.595246	-0.640536	H	1.179387	6.969756	2.989545
H	6.549332	-0.705817	-1.125556	H	-0.003106	6.415920	4.187672
C	1.842364	1.424635	5.297379	C	2.102607	-3.416198	-2.707133
H	1.869128	1.773406	6.327014	C	2.235839	-4.219611	-4.007979
C	4.772282	3.395036	-3.241339	H	3.188058	-4.014047	-4.510120
H	5.762612	3.313391	-3.681687	H	1.428898	-3.952008	-4.694632
C	-4.571848	2.019609	0.403796	H	2.187735	-5.299083	-3.826040
H	-3.774654	1.348547	0.073694	C	2.275997	-1.920762	-2.980028
H	-5.341924	2.034539	-0.370693	H	1.391749	4.187269	4.423737

H	2.499575	4.858653	3.229446
H	1.752138	3.267346	2.958555
H	3.306269	-1.685052	-3.274513
H	2.038467	-1.326681	-2.092119
H	1.607925	-1.612189	-3.790044
H	-1.934476	1.019810	-3.935477
C	-1.129712	0.384077	-1.402390
H	-1.296166	0.534794	-4.012993

49_TS12.log

SCF (wB97x) = -3920.43311639

E(SCF)+ZPE(0 K)= -3918.856982

H(298 K)= -3918.759214

G(298 K)= -3918.989230

Lowest Frequency = -122.9899cm-1

W	-2.440493	-0.788408	-2.719975
Al	-0.125397	2.929878	-0.552753
Al	0.407715	-2.592027	0.955664
O	-0.217483	-1.566411	-0.302402
O	-1.053959	1.703004	-1.437862
O	1.282332	-1.445661	1.907553
O	0.777339	1.844439	0.500615
C	-3.755579	-1.549038	-4.048877
N	0.965532	4.121402	-1.553694
N	-0.727514	-3.443845	2.222019
C	-1.895214	-2.618259	-2.172234
N	-1.279259	4.262062	0.176665
N	1.125964	-4.175946	0.193440
C	-1.108684	-0.925555	-4.270529
O	-4.515903	-1.974615	-4.816184
O	-1.613576	-3.708427	-1.881479
O	-4.917109	-1.197040	-0.740532
O	-0.447051	-1.119973	-5.200227
C	2.977144	1.110155	3.319581
H	3.878767	1.078815	2.714860
C	-1.081512	-2.780168	3.458033
C	-0.165705	-0.207143	-0.329692
C	1.316388	3.080586	-4.285590
H	0.426552	3.567743	-3.872195
C	1.780110	0.618368	2.806943
C	-1.962526	3.951143	1.413351
C	2.463994	-4.272088	-0.353546
C	1.386621	6.204220	-2.816816
H	2.414878	6.276310	-2.452992
H	0.965952	7.206966	-2.895030
H	1.431418	5.764592	-3.816529
C	-2.283816	-2.060999	3.552188
C	3.064948	-0.312123	0.801308
C	-3.281871	3.469060	1.397846
C	2.477702	3.312786	-3.329818
C	0.683205	0.503196	0.499632
C	-3.838866	3.067841	2.613827
H	-4.852788	2.674175	2.629216
C	3.782209	3.073377	-3.768450
H	3.947075	2.725608	-4.784743
C	0.322023	-5.247264	0.092318
C	2.293320	3.756126	-2.009359
C	-2.481305	6.423580	0.138295
H	-3.377340	6.320536	-0.484249
H	-2.132514	7.454351	0.053419

H	-2.767736	6.221305	1.170436
C	0.546981	5.352965	-1.895308
C	5.140047	-1.562892	0.652563
H	5.760961	-2.385969	0.997450
C	3.900149	-1.345602	1.239002
H	3.551733	-1.986198	2.043864
C	-1.255777	4.153056	2.614094
H	0.791349	-3.054280	-1.988051
C	-0.812544	-5.442355	0.889428
H	-1.375493	-6.349349	0.707304
C	0.613457	-6.307695	-0.938536
H	1.680345	-6.500629	-1.057241
H	0.230735	-5.938592	-1.897709
H	0.097816	-7.239156	-0.700187
C	3.163702	-5.476709	1.812608
H	2.202114	-5.075964	2.147021
C	-0.195880	-2.851245	4.550729
C	1.688795	-0.170215	1.485826
C	3.514228	0.513804	-0.227373
H	2.892280	1.328318	-0.585819
C	1.005690	1.586413	-4.394248
H	1.869876	1.031796	-4.778241
H	0.169230	1.419954	-5.079165
H	0.736682	1.158480	-3.423419
C	3.380317	3.928551	-1.127073
C	3.042508	1.603919	4.625013
H	3.991041	1.967327	5.013068
C	2.770354	-3.762641	-1.626184
C	-1.426328	5.471363	-0.367251
C	3.451881	-4.910756	0.427454
C	0.701061	1.141825	4.914821
H	-0.195167	1.132324	5.531042
C	4.756060	0.291678	-0.823914
H	5.072204	0.933415	-1.641054
C	4.076518	-3.908994	-2.101928
H	4.323846	-3.517274	-3.085733
C	-3.191507	-1.814051	2.358860
H	-2.846814	-2.424779	1.516762
C	0.051758	4.937053	2.648295
H	0.428391	5.021644	1.623504
C	4.664029	3.706132	-1.625165
H	5.520905	3.840225	-0.972321
C	4.314862	3.866036	1.243059
H	4.543511	2.808912	1.075849
H	4.016417	3.984828	2.289662
H	5.232211	4.447726	1.093701
C	1.150156	-3.552328	4.464458
H	1.386728	-3.683435	3.405832
C	-1.227169	-4.657385	1.975676
C	-3.991144	-0.975478	-1.400883
C	-4.133686	3.461012	0.134452
H	-3.542474	3.904795	-0.673662
C	1.564114	3.668781	-5.680578
H	1.866996	4.721096	-5.644855
H	0.652054	3.597674	-6.281369
H	2.345599	3.118806	-6.215393
C	-0.625325	5.940045	-1.415511
H	-0.862467	6.926483	-1.794139
C	-1.853616	3.730163	3.801245
H	-1.320944	3.855584	4.740689
C	0.638063	0.654939	3.616846
H	-0.299556	0.250820	3.241565

C	-2.617215	-1.476110	4.776893	H	-5.167899	5.324054	0.669656
H	-3.547832	-0.919673	4.861141	C	5.570010	-0.747568	-0.390963
C	5.050751	-4.555346	-1.359839	H	6.531458	-0.925711	-0.865890
H	6.055779	-4.672241	-1.757249	C	1.910843	1.609145	5.429997
C	3.002571	5.862033	0.477113	H	1.967296	1.975272	6.452294
H	3.867865	6.393198	0.062892	C	4.868042	3.291713	-2.935893
H	2.922094	6.131253	1.536718	H	5.877965	3.122886	-3.300965
H	2.101973	6.231147	-0.023600	C	-4.505343	2.049611	-0.313475
C	1.081680	-4.943680	5.105884	H	-3.608765	1.449078	-0.482854
H	0.803377	-4.872992	6.163907	H	-5.074535	2.080575	-1.249643
H	2.051925	-5.450148	5.047514	H	-5.123725	1.540419	0.434061
H	0.342290	-5.579754	4.605559	C	1.146794	4.273790	3.477911
C	2.277325	-2.706220	5.064968	C	4.225817	-5.054039	2.834007
H	2.299231	-1.712961	4.604725	H	5.200938	-5.502311	2.614392
H	3.243541	-3.191972	4.890398	H	3.939475	-5.382006	3.839249
H	2.168767	-2.583894	6.148402	H	4.352774	-3.966362	2.853874
C	-2.271591	-5.239940	2.892902	C	-3.070692	-0.343145	1.948039
H	-1.932279	-5.211594	3.932388	H	-3.325156	0.317298	2.784739
H	-2.497218	-6.270325	2.616661	H	-3.750522	-0.124108	1.124818
H	-3.191955	-4.650557	2.843268	H	-2.052667	-0.097044	1.625373
C	-0.577135	-2.261888	5.755595	C	-4.658592	-2.167975	2.629539
H	0.090092	-2.322806	6.611294	H	-4.778044	-3.196721	2.986564
C	-3.123978	3.167611	3.800193	H	-5.240114	-2.054962	1.709506
H	-3.571796	2.835540	4.733268	H	-5.097729	-1.505628	3.384345
C	3.182401	4.344477	0.328665	C	-1.784515	-1.588451	5.878893
H	2.271150	3.851170	0.687217	H	-2.066358	-1.139239	6.828017
C	4.733268	-5.053461	-0.103439	C	3.035678	-7.005472	1.782085
H	5.498065	-5.562513	0.478507	H	2.222417	-7.331230	1.125997
C	-5.397806	4.312055	0.320810	H	2.831248	-7.392635	2.786672
H	-6.073512	3.857043	1.053058	H	3.962786	-7.467417	1.423017
H	-5.944571	4.389590	-0.624939	C	-0.214826	6.364217	3.150396

H	-0.962377	6.882843	2.540873
H	0.708223	6.954233	3.127410
H	-0.579940	6.349092	4.183665
C	1.744132	-3.086645	-2.519605
C	1.532434	-3.868870	-3.822097
H	2.441897	-3.879534	-4.434587
H	0.738165	-3.404549	-4.415671
H	1.250748	-4.909483	-3.629539
C	2.149323	-1.640165	-2.817229
H	0.861779	4.174805	4.529993
H	2.057926	4.883296	3.446238
H	1.383168	3.277410	3.097804
H	3.122089	-1.589537	-3.321898
H	2.222138	-1.054125	-1.897376
H	1.410271	-1.170003	-3.470140
H	-2.755687	1.187954	-2.835921
C	-1.062269	0.378608	-1.334385
H	-3.046082	0.935762	-3.525406

50_Int1aprima.log

SCF (wB97x) = -3920.43645857

E(SCF)+ZPE(0 K)= -3918.858565

H(298 K)= -3918.760955

G(298 K)= -3918.987935

Lowest Frequency = 16.6896cm-1

W	-2.756003	-0.298725	-2.663006
Al	0.148213	2.940809	-0.564507
Al	0.214914	-2.655053	0.762999
O	-0.603490	-1.493716	-0.253808
O	-0.933619	1.855590	-1.449003
O	1.112212	-1.605695	1.814165
O	0.944865	1.741513	0.419113
C	-4.312939	-0.872646	-3.819323
N	1.197605	3.946036	-1.800238
N	-0.854683	-3.889191	1.760433
C	-2.694475	-2.124488	-1.864435
N	-0.643139	4.413167	0.297217
N	1.255449	-3.968672	-0.159379
C	-1.594976	-0.970698	-4.206536
O	-5.216756	-1.206600	-4.466744
O	-2.674824	-3.212245	-1.457217
O	-5.062982	0.765762	-0.711193
C	-1.135728	0.549629	-1.341121
O	-1.047063	-1.376417	-5.143036
C	1.465110	1.720254	3.330725
H	2.284976	2.147107	2.758443
C	-1.516346	-3.465433	2.977368
C	-0.330819	-0.157551	-0.330422
C	1.058486	2.920961	-4.582656
H	0.332277	3.565229	-4.077464
C	0.904221	0.507153	2.922191
C	-0.958290	4.502310	1.708011
C	2.600128	-3.690745	-0.617198
C	1.540632	5.912150	-3.268044
H	2.514352	5.478100	-3.494769
H	1.671319	6.949743	-2.951076
H	0.942758	5.921640	-4.185437
C	-2.878461	-3.116486	2.953876
C	2.887516	-0.130407	1.414425
C	-2.110952	3.896046	2.227288

C	2.273432	2.755217	-3.677356	C	0.357261	1.586893	-4.836738
C	0.602295	0.446232	0.488790	H	1.005894	0.891140	-5.380228
C	-2.416954	4.101763	3.574948	H	-0.546639	1.736907	-5.438010
H	-3.304830	3.633463	3.992498	H	0.063376	1.116352	-3.894995
C	3.378540	2.045575	-4.154254	C	3.522420	3.197792	-1.603025
H	3.333560	1.597363	-5.144087	C	0.995695	2.372582	4.465279
C	0.816739	-5.221494	-0.328716	H	1.448842	3.311812	4.772462
C	2.354688	3.304916	-2.385050	C	2.800127	-3.062470	-1.860051
C	-2.004955	6.456052	0.053524	C	-0.952099	5.497874	-0.445932
H	-2.937541	5.910582	0.230650	C	3.684833	-4.108356	0.175770
H	-2.186195	7.244695	-0.677827	C	-0.629750	0.635855	4.796588
H	-1.720722	6.910752	1.005214	H	-1.452028	0.195066	5.355699
C	0.800113	5.150105	-2.197790	C	4.773714	-0.291989	-0.099744
C	5.153084	-0.243265	2.272383	H	5.145745	-0.353729	-1.118908
H	5.830477	-0.263909	3.122628	C	4.111213	-2.923853	-2.319772
C	3.780576	-0.160539	2.488347	H	4.290942	-2.461877	-3.285929
H	3.397233	-0.132715	3.504882	C	-3.727502	-3.172699	1.693058
C	-0.107660	5.275948	2.525919	H	-3.167922	-3.693031	0.911245
H	0.826033	-2.261100	-2.043044	C	1.184560	5.907943	2.016608
C	-0.319502	-5.737805	0.304845	H	1.363803	5.549171	0.997610
H	-0.583993	-6.757671	0.054723	C	4.598210	2.485157	-2.129736
C	1.612306	-6.208600	-1.150669	H	5.504278	2.375005	-1.542318
H	2.138821	-5.738337	-1.980938	C	4.669127	3.309036	0.689051
H	0.951073	-6.988605	-1.533139	H	4.476015	2.247301	0.861122
H	2.363645	-6.687606	-0.513763	H	4.621800	3.827030	1.652735
C	3.513100	-4.732066	1.555113	H	5.694050	3.420072	0.316677
H	2.449441	-4.942569	1.714556	C	0.708311	-3.791254	4.217975
C	-0.767431	-3.411939	4.172510	H	1.148960	-3.508118	3.258841
C	1.370900	-0.237597	1.653795	C	-1.021923	-5.157816	1.366803
C	3.402637	-0.196009	0.119917	C	-4.186373	0.388152	-1.365607
H	2.725015	-0.180405	-0.731770	C	-3.028129	3.020664	1.391083

H	-2.689690	3.055193	0.348435
C	1.437397	3.584158	-5.914287
H	1.978237	4.525013	-5.771327
H	0.537299	3.790651	-6.502917
H	2.074994	2.925830	-6.514032
C	-0.326748	5.800679	-1.651689
H	-0.637668	6.712163	-2.148840
C	-0.473453	5.476947	3.855912
H	0.157138	6.090624	4.495684
C	-0.152799	-0.018522	3.662917
H	-0.604195	-0.954209	3.354747
C	-3.473290	-2.690034	4.144166
H	-4.521857	-2.402880	4.138106
C	5.192401	-3.355885	-1.564139
H	6.204658	-3.236266	-1.942439
C	3.972821	5.402502	-0.515995
H	4.911660	5.494475	-1.073940
H	4.090896	5.939049	0.431481
H	3.189883	5.908078	-1.090337
C	0.891621	-5.303804	4.399769
H	0.416774	-5.645560	5.327239
H	1.957585	-5.555560	4.454866
H	0.461594	-5.871924	3.568020
C	1.485725	-3.022380	5.289912
H	1.310042	-1.944926	5.206188
H	2.558253	-3.201589	5.168252
H	1.218518	-3.342254	6.303848
C	-1.986984	-6.057663	2.100005
H	-1.883518	-5.955761	3.182741
H	-1.822639	-7.098949	1.820873
H	-3.016827	-5.791551	1.845159
C	-1.416275	-3.002715	5.337891
H	-0.859735	-2.952484	6.268630
C	-1.622359	4.900647	4.380749
H	-1.890347	5.065986	5.421105
C	3.645887	3.922208	-0.267279
H	2.674720	3.870687	0.240098
C	4.976949	-3.922002	-0.317919
H	5.826967	-4.232824	0.284867
C	-4.481965	3.508078	1.415461
H	-4.913041	3.429027	2.419680
H	-5.090202	2.894829	0.743779
H	-4.567861	4.554179	1.100228
C	5.655693	-0.314422	0.975863
H	6.726929	-0.388511	0.805865
C	-0.054617	1.833384	5.203914
H	-0.421561	2.349049	6.087924
C	4.524726	1.899605	-3.388006
H	5.370511	1.337585	-3.775789
C	-2.937314	1.567019	1.865643
H	-1.921574	1.170359	1.762619
H	-3.616169	0.939453	1.286242
H	-3.212255	1.480350	2.923676
C	2.382460	5.479179	2.872491
C	3.956064	-3.739669	2.636273
H	4.997971	-3.434918	2.483134
H	3.880999	-4.198366	3.629530
H	3.337870	-2.836982	2.614953
C	-4.001680	-1.763308	1.169767
H	-4.511368	-1.155176	1.926890
H	-4.641540	-1.805971	0.282778
H	-3.067962	-1.264217	0.890160

C	-5.048895	-3.921748	1.911638
H	-4.901206	-4.903847	2.374210
H	-5.555118	-4.067980	0.952136
H	-5.730392	-3.355765	2.556397
C	-2.756266	-2.637198	5.328417
H	-3.239391	-2.313198	6.246881
C	4.271192	-6.057508	1.701065
H	4.017334	-6.769436	0.908539
H	4.030944	-6.522898	2.663227
H	5.355786	-5.905646	1.673368
C	1.095544	7.438359	1.955725
H	0.303411	7.774800	1.279338
H	2.040669	7.860398	1.596366
H	0.894362	7.861869	2.946272
C	1.636608	-2.572953	-2.713224
C	1.082115	-3.686647	-3.613976
H	1.881560	-4.126677	-4.223216
H	0.324216	-3.281906	-4.291061
H	0.610963	-4.484903	-3.033603
C	2.014312	-1.358702	-3.564886
H	2.274912	5.810962	3.911048
H	3.308261	5.919070	2.485906
H	2.495255	4.390518	2.876054
H	2.705369	-1.624756	-4.374196
H	2.480734	-0.563211	-2.971693
H	1.118061	-0.954268	-4.035091
H	-2.376482	1.587392	-3.160796
H	-2.958955	1.396616	-3.667312

7 REFERENCES

(1) Cui, C.; Roesky, H. W.; Schmidt, H.-G.; Noltemeyer, M.; Hao, H.; Cimpoesu, F. Synthesis and Structure of a Monomeric Aluminum(I) Compound [{HC(CMeNAr)$_2$}Al] (Ar=2,6–^{i}Pr$_2$C$_6$H$_3$): A Stable Aluminum Analogue of a Carbene. *Angew. Chem. Int. Ed.* **2000**, *39* (23), 4274–4276.

(2) Kong, R. Y.; Crimmin, M. R. Carbon Chain Growth by Sequential Reactions of CO and CO_2 with [W(CO)$_6$] and an Aluminum(I) Reductant. *J. Am. Chem. Soc.* **2018**, *140* (42), 13614–13617.

(3) Walz, F.; Moos, E.; Garnier, D.; Köppe, R.; Anson, C. E.; Breher, F. A Redox-Switchable Germylene and Its Ligating Properties in Selected Transition Metal Complexes. *Chem. - A Eur. J.* **2017**, *23* (5), 1173–1186.

(4) Szymańska-Buzar, T.; Kern, K. Photosubstitution of Carbon Monoxide in W(CO)$_6$ by Alkyne: NMR Detection of Thermally Unstable Alkyne Tungsten(0) Carbonyl Complexes. *J. Organomet. Chem.* **2001**, *622* (1–2), 74–83.

(5) SHELXTL v5.1, Bruker AXS, Madison, WI, 1998.

(6) G.M. Sheldrick. SHELX-2013. *Acta Cryst.* **2015**, *C71*, 3–8.

(7) A.L. Spek (2003, 2009) PLATON, A Multipurpose Crystallographic Tool, Utrecht University, Utrecht, The Netherlands. See Also A.L. Spek, Acta. Cryst., 2015, C71, 9-18.

(8) Frisch, M. J.; Trucks, G. W.; Schlegel, H. B.; Scuseria, G. E.; Robb, M. A.; Cheeseman, J. R.; Scalmani, G.; Barone, V.; Mennucci, B.; Petersson, G. A.; Nakatsuji, H.; Caricato, M.; Li, X.; Hratchian, H. P.; Izmaylov, A. F.; Bloino, J.; Zheng, G.; Sonnenb, D. J. Gaussian, Inc., Wallingford, CT 2009. 2009.

(9) Chai, J. Da; Head-Gordon, M. Systematic Optimization of Long-Range Corrected Hybrid Density Functionals. *J. Chem. Phys.* **2008**, *128* (8).

(10) Hratchian, H. P.; Schlegel, H. B. *Theory and Applications of Computational Chemistry*; Dykstra, C. E., Frenking, G., Kim, K. S., Scuseria, G. E., E., Ed.; Elsevier: Amsterdam, 2005.

(11) Gleadening, E. D.; Bakenhoop, J. K.; Reed, A. E.; Carpenter, J. E.; Bohmann, J. A.; Morales, C. M.; Landis, C. R.; Weinhold, F. Theoretical Chemistry Institute. University of Wisconsin: Madison 2013.

(12) Stephen, P. J.; Devlin, F. J.; Chabalowski, C. F.; Frisch, M. J. Ab Initio Calculation of Vibrational Absorption. *J. Phys. Chem.* **1994**, *98* (45), 11623–11627.

(13) Frost, A. A.; Musulin, B. Density-Functional Thermochemistry. III. The Role of Exact Exchange. *Hydrocarb. J. Chem. Phys.* **1953**, *21* (October 1992), 5648.

(14) Zhao, Y.; Truhlar, D. G. The M06 Suite of Density Functionals for Main Group Thermochemistry, Thermochemical Kinetics, Noncovalent Interactions, Excited States, and Transition Elements: Two New Functionals and Systematic Testing of Four M06-Class Functionals and 12 Other Function. *Theor. Chem. Acc.* **2008**, *120* (1–3), 215–241.

(15) Zhao, Y.; Truhlar, D. G. A New Local Density Functional for Main-Group Thermochemistry, Transition Metal Bonding, Thermochemical Kinetics, and Noncovalent Interactions. *J. Chem. Phys.* **2006**, *125* (19).
